# Highly Antiproliferative Latonduine and Indolo[2,3-*c*]quinoline Derivatives: Complex Formation with Copper(II) Markedly Changes the Kinase Inhibitory Profile

**DOI:** 10.1021/acs.jmedchem.1c01740

**Published:** 2022-02-01

**Authors:** Christopher Wittmann, Felix Bacher, Eva A. Enyedy, Orsolya Dömötör, Gabriella Spengler, Christian Madejski, Jóhannes Reynisson, Vladimir B. Arion

**Affiliations:** † Institute of Inorganic Chemistry of the University of Vienna, Währinger Strasse, 42, Vienna A1090, Austria; ‡ Department of Inorganic and Analytical Chemistry, Interdisciplinary Excellence Centre, University of Szeged, Dóm tér 7, Szeged H-6720, Hungary; § MTA-SZTE Lendület Functional Metal Complexes Research Group, University of Szeged, Dóm tér 7, Szeged H-6720, Hungary; ∥ Department of Medical Microbiology, Albert Szent-Györgyi Health Center and Albert Szent-Györgyi Medical School, University of Szeged, Semmelweis u. 6, Szeged H-6725, Hungary; ⊥ School of Pharmacy and Bioengineering, Keele University, Hornbeam Building, Staffordshire ST5 5BG, United Kingdom

## Abstract

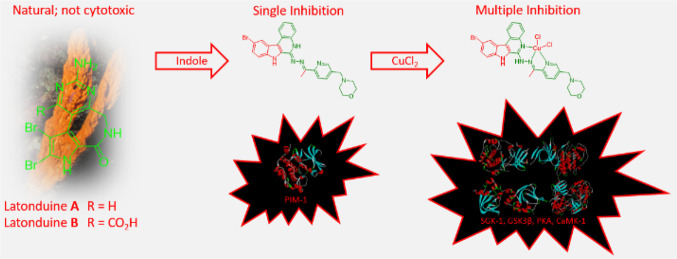

A series of latonduine and indoloquinoline derivatives **HL^1^**–**HL^8^** and their copper(II) complexes (**1–8**) were synthesized and comprehensively characterized. The structures of five compounds (**HL^6^**, **[CuCl(L^1^)(DMF)]·DMF**, **[CuCl(L^2^)(CH_3_OH)]**, **[CuCl(L^3^)]·0.5H_2_O**, and **[CuCl_2_(H_2_L^5^)]Cl·2DMF**) were elucidated by single crystal X-ray diffraction. The copper(II) complexes revealed low micro- to sub-micromolar IC_50_ values with promising selectivity toward human colon adenocarcinoma multidrug-resistant Colo320 cancer cells as compared to the doxorubicin-sensitive Colo205 cell line. The lead compounds **HL^4^** and **4** as well as **HL^8^** and **8** induced apoptosis efficiently in Colo320 cells. In addition, the copper(II) complexes had higher affinity to DNA than their metal-free ligands. **HL^8^** showed selective inhibition for the PIM-1 enzyme, while **8** revealed strong inhibition of five other enzymes, i.e., SGK-1, PKA, CaMK-1, GSK3β, and MSK1, from a panel of 50 kinases. Furthermore, molecular modeling of the ligands and complexes showed a good fit to the binding pockets of these targets.

## 1 Introduction

**Chart 1:**
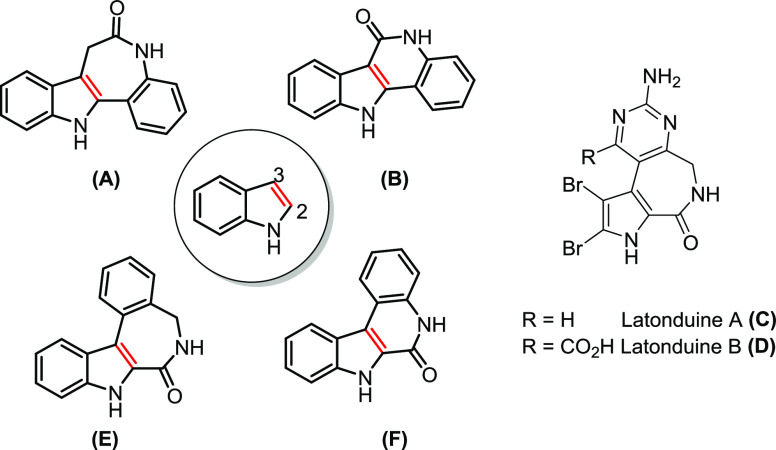
Indolo[3,2-*d*]benzazepine (**A**, Paullone), Indolo[3,2-*c*]quinoline (**B**), Naturally Occurring Latonduines **C** and **D**, Indolo[2,3-*d*]benzazepine (**E**), and Indolo[2,3-*c*]quinoline (**F**)

Indolobenzazepines and indoloquinolines are fused heterocyclic scaffolds, which have gained a considerable interest in the field of medicinal chemistry.^1−17^ Indolo[3,2-*d*]benzazepines or paullones (backbone **A** in Chart 1), first synthesized in 1992, were discovered as potential inhibitors of cyclin-dependent kinases (Cdks)^7,18,19^ with antiproliferative activity similar to that of flavopiridol, the first Cdk-inhibitor that reached clinical trials as an anticancer drug. Later, other possible targets have been identified, namely, sirtuins,^20,21^ GSK3β,^18,19,22,23^ and mitochondrial malate dehydrogenase.^23^ Indolo[3,2-*c*]quinolines (backbone **B** in Chart 1) are known to induce apoptosis in cancer cells. DNA intercalation and poisoning topoisomerase I/II (topo I/II) are considered the mechanisms of action.^24−26^ Furthermore, some of the indolo[3,2-*c*]quinolines are effective and selective KRAS-mutated oncogene G-quadruplex stabilizers causing cancer cell apoptosis.^27^ Indolo[3,2-*d*]benzazepines are non-planar heterocyclic systems due to the sp^3^-hybridized methylene carbon atom in the seven-membered azepine ring, whereas the indolo[3,2-*c*]quinolines are planar, making them effective DNA intercalators and/or topo I/II inhibitors.

Latonduines (backbones **C** and **D** in Chart 1) were first extracted from the Indonesian sponge *Stylissa carteri* and are not cytotoxic to cancer cells.^28,29^ However, substitution of their pyrrole ring by an indole unit made them cytotoxic.^30,31^ The resulting indolo[2,3-*d*]benzazepine (backbone **E** in Chart 1) is a microtubule destabilizing agent (MDA) targeting the colchicine binding site.^30^ The two isomeric backbones indolo[3,2-*d*]benzazepine **A** and indolo[2,3-*d*]benzazepine **E** are related structurally as shown in Chart 1. Nevertheless, by flipping the indole moiety and shifting the lactam unit in paullone **A**, one obtains not only increased cytotoxicity but also a different mode of action.^30^

Being intrigued by the activity of indolo[3,2-*d*]benzazepine- and indolo[3,2-*c*]quinoline-based molecules as potential anticancer drugs, we decided to extend our chemistry to other related isomeric systems, namely, indolo[2,3-*d*]benzazepine- and indolo[2,3-*c*]quinoline-derived species, with unexplored chemistry and biological effects. We envisioned exciting new results in the field of metal-based anticancer drugs and, in particular, new structure–activity relationships.

One of the major drawbacks of these isomeric scaffolds is their limited aqueous solubility and bioavailability. This issue was successfully addressed for many indolo[3,2-*d*]benzazepine and indolo[3,2-*c*]quinoline derivatives and several latonduines by creating metal binding sites at their backbones and metal complex formation. Werner-type coordination complexes of copper(II), ruthenium(II), osmium(II), gallium(III), and organometallic compounds were synthesized and investigated as potential anticancer drugs.^32−40^ The reported results revealed that the metal complexes did not only enhance the aqueous solubility and bioavailability but also augmented their antiproliferative activity both *in vitro* and *in vivo*. Nevertheless, bioavailability and aqueous solubility need further improvement, requiring other approaches to enhance their pharmacological profile.

Morpholine, as a known biologically active moiety, has been attached to the main scaffolds since it is considered to improve the necessary pharmacological parameters of drug candidates.^41,42^ In our recent paper,^43^ we reported that the Schiff base resulted from condensation of the 11-bromo-7-hydrazin-yl derivative of **E** (Chart 1) with 2-acetylpyridine and its copper(II) complex showed the highest cytotoxicity among the compounds tested. This prompted us to further develop this backbone and prepare 2-acetylpyridine with a morpholine unit. As a starting material, the respective aldehyde was used, which was recently reported by us.^44^

Protein kinases represent an excellent target for cancer therapy.^45−49^ It should be also stressed that the multitargeted kinase inhibitors have become a “hot topic”, accounting for about 25% of drug discovery research.^48,49^ Initial attempts to create highly selective mono-kinase inhibitors to avoid unexpected toxic effects have been steadily displaced by two anticancer therapies that target several kinases and block distinct kinase signaling pathways as they showed therapeutic benefits in the treatment of complex cancer diseases.^46^ The first therapy is based on using several selective mono-kinase inhibitors simultaneously, while the second is based on using a single drug as a multikinase inhibitor. Advantages and hurdles of both therapies have been discussed in the literature.^46,48,49^ The second therapy, which implies the use of a single drug as a multikinase inhibitor that revealed higher potency, permits avoiding the consequences of drug–drug interactions, which can affect absorption, metabolism, excretion, plasma level, and, finally, activities, as well as reducing side effects and is much easier to apply.^46^

**Chart 2:**
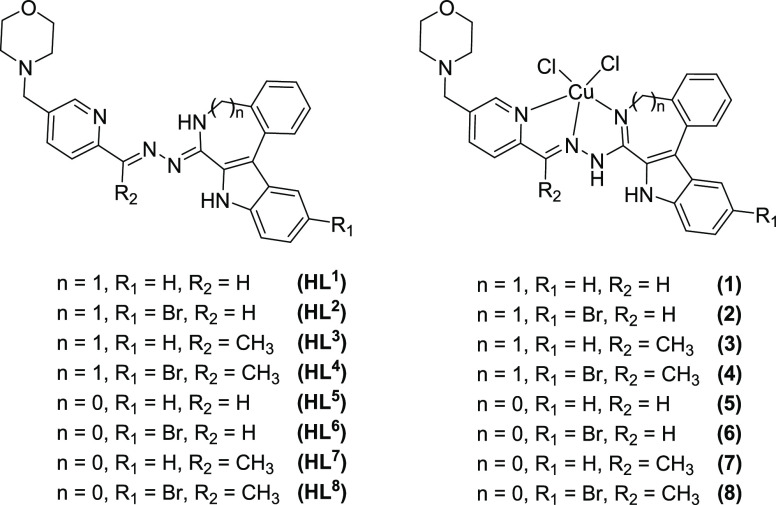
Copper(II) Complexes **1**–**8** and Their Metal-Free Ligands **HL^1^**–**HL^8^**

Herein, we report the synthesis and characterization of new chelating systems derived from indolo[2,3-*d*]benzazepine **E** and indolo[2,3-*c*]quinoline **F** and of their copper(II) complexes (Chart 2), speciation in aqueous solution, and antiproliferative activity. The inhibition ability of the lead compounds in a panel of 50 kinases was investigated *in vitro* and by molecular modeling, providing insights into the mode of action of the most potent copper(II) complex and its metal-free ligand. These modified molecules offer a broad spectrum of various effects on malign cells, while some of them show a marked increase in aqueous solubility, thus increasing the bioavailability and improving the pharmacological profile. Lead compound **8** and its proligand **HL^8^** do not only target cancer specific kinases but also offer an excellent pharmacological profile.

## 2 Results and Discussion

### 2.1 Synthesis and Characterization of Starting Building Blocks and Ligands

**Scheme 1:**
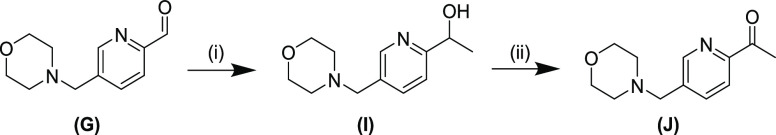
Synthesis of 2-Acetyl-5-(morpholinomethyl)-pyridine **J** Starting from 2-Formyl-5-(morpholinomethyl)-pyridine **G**(44) Reagents and conditions: (i) MeMgBr, THF_dry_, 0 °C; (ii) (COCl)_2_, DMSO_dry_, NEt_3_, DCM_dry_, −80 °C.

The aldehyde **G** prepared as reported elsewhere^44^ was converted into secondary alcohol **I** by reaction with the Grignard reagent (CH_3_MgBr) and workup in 92% yield. Swern oxidation of **I** resulted in ketone **J** (Scheme 1), which was purified chromatographically to give an easily crystallizable product in 69% yield.

The ^1^H NMR spectrum of **J** agreed with the expected structure, which, in addition, has been confirmed by SC-XRD (see Chart S1 for atom numbering scheme and Figure S1 in the Supporting Information).

**Scheme 2:**
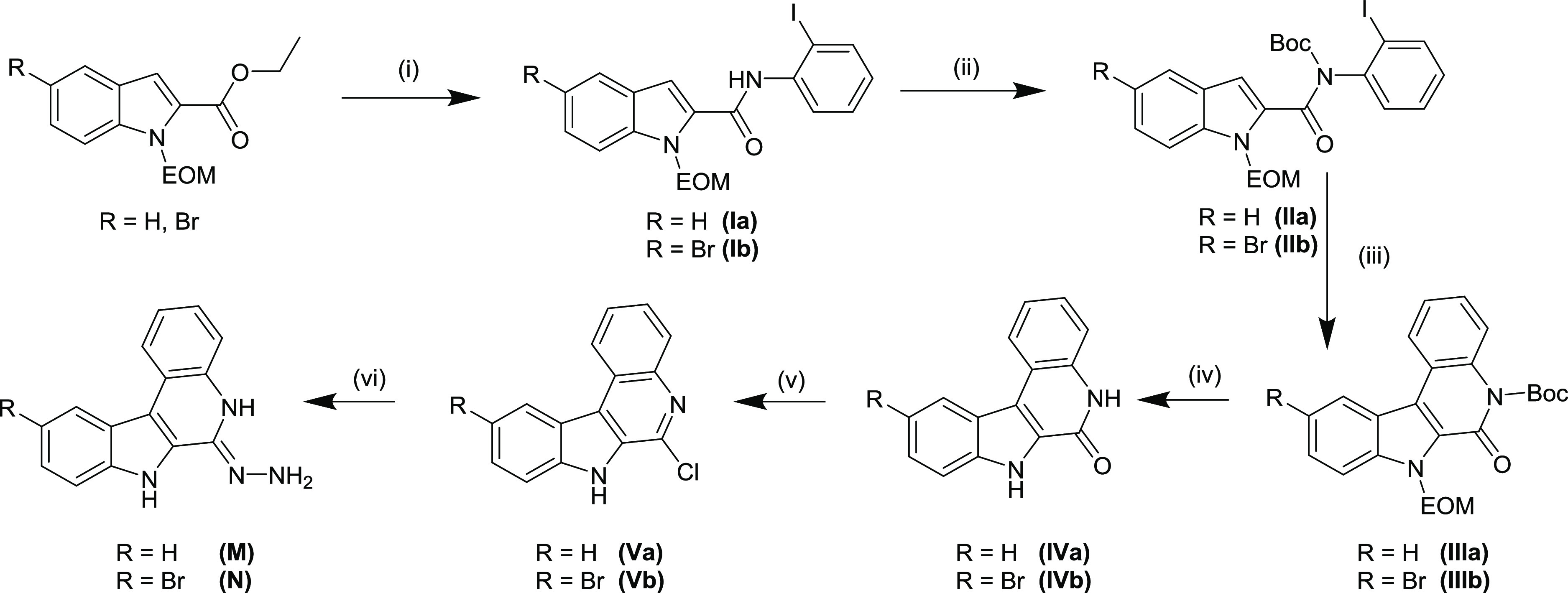
Synthesis of **M** and **N** Reagents and conditions: (i) AlMe_3_, 2-iodoaniline, CH_2_Cl_2dry_, −20 °C to RT, 19 h; (ii) Boc_2_O, DMAP, CH_3_CN_dry_, RT, 72 h; (iii) Pd(OAc)_2_, PPh_3_, Ag_2_CO_3_, DMF_dry_, 100 °C, 2 h; (iv) EtOH/HCl, 100 °C, 16 h; (v) POCl_3_, 120 °C, 16 h; (vi): N_2_H_4_·H_2_O 131 °C, 16 h.

The derivative **IVb** (Scheme 2) has not been reported previously. Its synthesis has been performed by following the procedures described in the literature for unsubstituted indolo[2,3-*c*]quinoline **IVa**(50) as shown in Scheme 2. In the first step, ethyl 5-bromo-1-ethoxymethyl-1*H*-indol-2-carboxylate was allowed to react with 2-iodoaniline in the presence of AlMe_3_ in dichloromethane (DCM) to give **Ib** in 84% yield. Protection of carboxamide nitrogen atom and isolation of **IIb** were realized in 99% yield by treatment of **Ib** with di-*tert-*butyl dicarbonate Boc_2_O in dry acetonitrile in the presence of catalytic amount of *N*,*N*-dimethyl-4-aminopyridine (DMAP). The intramolecular Heck cyclization reaction of **IIb** in the presence of Pd(OAc)_2_, PPh_3_, and Ag_2_CO_3_ followed by workup afforded **IIIb** in 19% yield. Full deprotection of **IIIb** and formation of **IVb** were accomplished in 79% yield by refluxing **IIIb** in EtOH:12 M HCl 4:1.

Then, compounds **IVa** and **IVb** were chlorinated with excess POCl_3_ at 120 °C to give rise to **Va**/**Vb** in >90% yield. Finally, the treatment of **Va**/**Vb** with excess hydrazine hydrate at reflux delivered the desired species **M** and **N** in >95% yield. This pathway to create chelating molecules with some modifications was also successful with core structures **A** and **B** (Chart 1).^51,52^

**Scheme 3:**
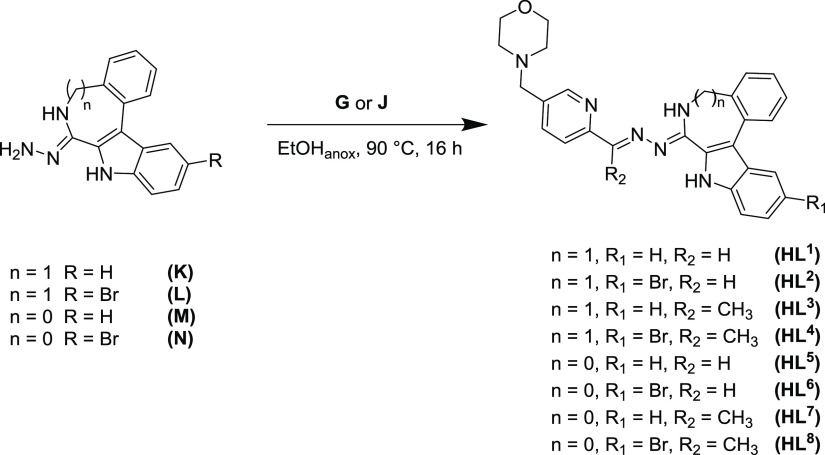
Synthesis of Proligands **HL^1^–HL^8^**

The potential ligands **HL^1^**–**HL^8^** were synthesized by Schiff base condensation reactions of hydrazin-yl derivatives **K**–**N** with aldehyde **G** or ketone **J** in anoxic ethanol (Scheme 3) in 57–98% yields by adapting literature protocols.^43,51,53^

^1^H NMR spectra of the potential ligands **HL^1^**–**HL^8^** show the typical peak pattern of the morpholine unit at around 2.35 and 3.55 ppm, as well as proton resonances of the linking methylene group between the morpholine unit and the pyridine ring at around 3.55 ppm sometimes overlapping with H^25^ for indolo[2,3-*d*]benzazepines or H^24^ for indolo[2,3-*c*]quinolines, respectively (for atom numbering scheme, see Chart S2). The additional methyl group as R_2_ in Scheme 3 is seen as a singlet at around 2.49 ppm. The 2D NMR spectra provided evidence that indolo[2,3-*d*]benzazepines **HL^1^**–**HL^4^** are solely present as tautomers with an exocyclic double bond between C^7^ and N^13^ as evidenced by weak coupling between H^6^ and C^5^ and triplet resonance in the ^1^H NMR spectra, suggesting the presence of two protons in the closest vicinity of H^6^.^43^ There were no other tautomeric forms identified. In contrast, NMR spectra of **HL^5^**–**HL^8^** indicate that these indolo[2,3-*c*]quinolines exist in two tautomeric forms in the solution. The major species possesses an exocyclic double bond with a hydrogen atom at N^5^, while the minor species contains an endocyclic double bond with a hydrogen atom at N^12^. The ratio between these two species is solvent- and concentration-dependent. At a concentration of about 10 mg/mL in DMSO-*d*_6_, the ratios between the major *vs* minor species are 1:0.02, 1:0.01, 1:0.80 and 1:0.15 for **HL^5^**–**HL^8^**, respectively. In most cases, a complete assignment of all ^1^H and ^13^C resonances was impeded by low signal intensity for minor species and signal overlapping. However, the high signal intensity of the minor species in the case of **HL^7^** made a complete assignment of all signals in both species possible. The chemical shift of N*H*^5^ for the species with an exocyclic double bond is 11.97 ppm, while that of N*H*^12^ for the species with an endocyclic double bond is 14.54 ppm. This was confirmed by a long-range ^1^H–^13^C HMBC 2D NMR experiment, where the proton N^5^*H* showed ^3^*J* couplings to quaternary carbons C^11c^ and C^6a^, while N^12^*H* revealed such couplings to quaternary carbon C^6a^ and ternary carbon C^14^, which is possible, if the hydrogen is bound to a hydrazinic nitrogen. The structural change in the molecule from an exocyclic to an endocyclic double bond leads to a shift of all ^1^H and ^13^C signals. While the ^1^H and ^13^C resonances of the morpholine moiety are only marginally affected, those near the hydrazinic moiety show major changes. In particular, the signals for the hydrogen C^14^*H* and imine carbon are upfield shifted from 8.52 to 7.57 ppm and from 152.19 to 131.14 ppm, respectively, when going from major to minor species (see Chart S2 for the NMR atom numbering scheme in the Supporting Information). At close inspection of the ^1^H NMR spectrum of **HL^7^**, two more sets of NMR signals with low intensities become apparent, which are presumably attributed to *E* and *Z* isomers of the previously described tautomers, leading to a total of four signal sets. However, low signal intensity, signal overlapping, and the absence of non-cross peaks in a two-dimensional ^1^H–^1^H NMR NOESY experiment made the identification of isomers difficult, if at all possible.

### 2.2 Synthesis and Characterization of Metal Complexes

Copper(II) complexes **1**–**8** were prepared by reactions of CuCl_2_·2H_2_O with **HL^1^–HL^8^** in a boiling mixture of methanol/isopropanol, a procedure used previously for the synthesis of copper(II) complexes with related Schiff bases without a morpholine moiety.^32,43^ The formation of copper(II) complexes **1**–**8** was confirmed by positive ion ESI mass spectra with peaks at *m*/*z* 512.14 and 548.11 attributed to [Cu^II^(**L^1^**)]^+^ and [Cu^II^Cl(**HL^1^**)]^+^, respectively (for **1**), *m*/*z* 592.07 assigned to [Cu^II^(**L^2^**)]^+^ (for **2**), at *m*/*z* 562.23 attributed to [Cu^II^Cl(**HL^3^**)]^+^ (for **3**), at *m*/*z* 498.27 assigned to [Cu^II^(**L^5^**)]^+^ (for **5**), at *m*/*z* 578.14 attributed to [Cu^II^(**L^6^**)]^+^ (for **6**), at *m*/*z* 512.20 assigned to [Cu^II^(**L^7^**)]^+^ (for **7**), and *m*/*z* 592.16 attributed to [Cu^II^(**L^8^**)]^+^ (for **8**). The negative ion ESI mass spectrum of **4** showed a strong peak at *m*/*z* 640.03, which could be easily assigned to [Cu^II^Cl(**L^4^**)–H^+^]^−^. The reaction of **HL^8^** with NiCl_2_·6H_2_O in methanol in a 1:1 molar ratio delivered a 1:2 nickel-to-ligand complex instead of the desired complex of 1:1 stoichiometry. Recrystallization of this product from DMF afforded crystals of the composition **[Ni(L^8^)(HL^8^)]Cl·2DMF**, the structure of which was determined by SC-XRD. Starting from **HL^7^** and NiCl_2_·6H_2_O in methanol in a 2:1 molar ratio, the complex **[Ni(HL^7^)_2_]Cl_2_·H_2_O** was synthesized. The ESI mass spectrum revealed a doubly charged peak at *m*/*z* 479.18 corresponding to [Ni(HL^7^)_2_]^2+^.

Elemental analyses were in good agreement with the composition proposed for all isolated complexes, attesting the purity (≥95%) required for biological assays. The coordination geometry was confirmed by SC-XRD measurements of complexes **[CuCl(L^1^)(DMF)]·DMF**, **[CuCl(L^2^)(CH_3_OH)]**, **[CuCl(L^3^)]·0.5H_2_O**, and **[CuCl_2_(H_2_L^5^)]Cl·2DMF**.

### 2.3 X-ray Crystallography

**Figure 1:**
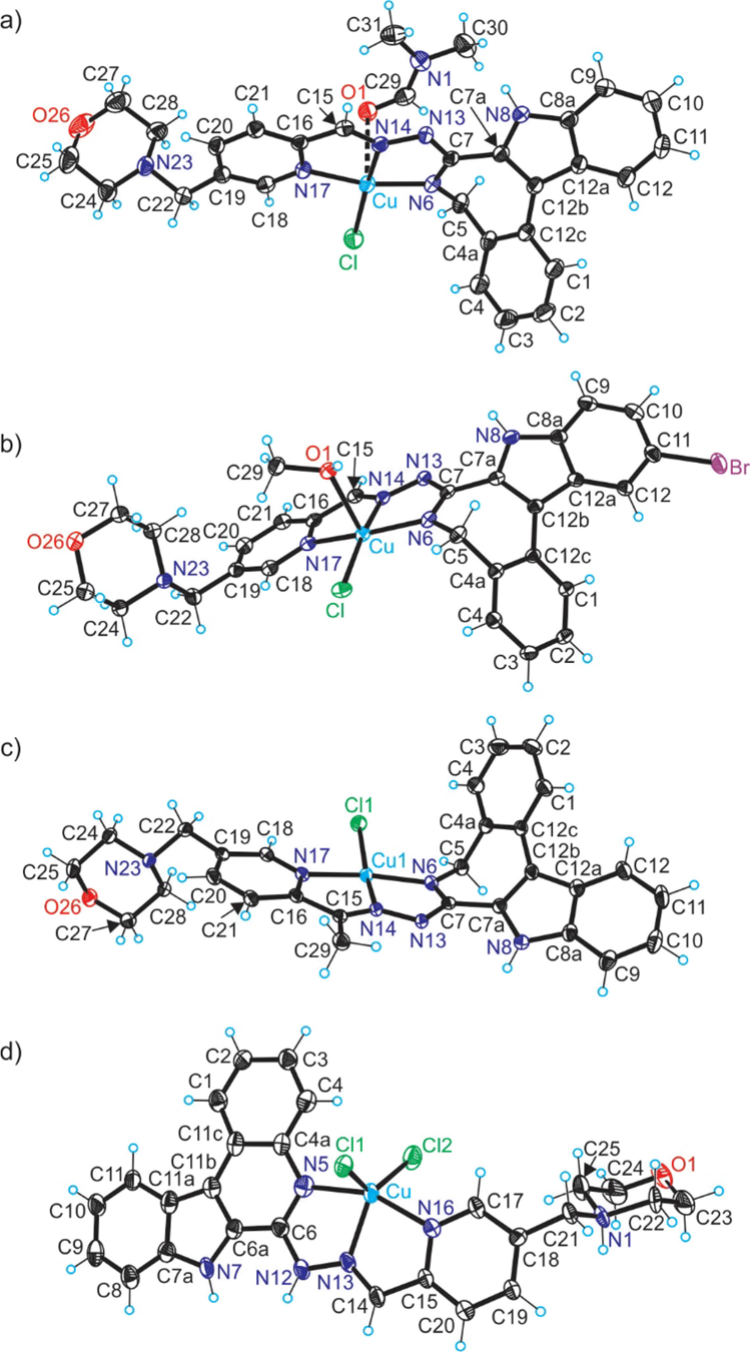
ORTEP views of (a) **[CuCl(L^1^)(DMF)]**, (b) **[CuCl(L^2^)(CH_3_OH)]**, (c) **[CuCl(L^3^)]**, and (d) **[CuCl_2_(H_2_L^5^)]^+^** with thermal ellipsoids at a 50% probability level. Selected bond distances (Å), bond angles (deg), and torsion angles (deg) in (a): Cu–N6 1.949(2), Cu–N14 1.963(2), Cu–N17 2.017(2), Cu–Cl 2.2355(9), Cu–O1 2.582(2); N6–C7 1.321(4), C7–N13 1.355(4), N13–N14 1.364(3), N14–C15 1.289(3), C15–C16 1.452(4), C16–N17 1.359(4), Θ_C4a–C5–N6–C7_–71.7(3); in (b): Cu–N6 1.9590(15), Cu–N14 1.9724(14), Cu–N17 2.0301(14), Cu–Cl 2.2555(4), Cu–O1 2.3493(12); N6–C7 1.322(2), C7–N13 1.366(2), N13–N14 1.354(2), N14–C15 1.290(2), C15–C16 1.455(2), C16–N17 1.366(2), Θ_C4a–C5–N6–C7_–72.84(19); in (c): Cu–N6 1.9407(19), Cu–N14 1.969(2), Cu–N17 2.0107(18), Cu–Cl 2.2475(7), N6–C7 1.305(3), C7–N13 1.374(3), N13–N14 1.364(3), N14–C15 1.302(3), C15–C16 1.479(3), C16–N17 1.353(3), Θ_C4a–C5–N6–C7_–73.6(3); and in (d): Cu–N5 2.013(8), Cu–N13 1.997(7), Cu–N16 2.024(7), Cu–Cl1 2.364(2), Cu–Cl2 2.281(2), N5–C6 1.334(12), C6–N12 1.383(11), N12–N13 1.350(9), N13–C14 1.281(11), C14–C15 1.458(12), C15–N16 1.366(11), Θ_C4a–N5–C6–C6a_ 1.4(13).

The results of SC-XRD studies of complexes **[CuCl(L^1^)(DMF)]·DMF**, **[CuCl(L^2^)(CH_3_OH)]**, **[CuCl(L^3^)]·0.5H_2_O**, and **[CuCl_2_(H_2_L^5^)]Cl·2DMF** are shown in Figure 1, while those for the metal-free indolo[2,3-*c*]quinoline **HL^6^** and **[Ni(L^8^)(HL^8^)]Cl·2DMF** are shown in Figures S3 and S4, respectively, with pertinent bond distances (Å), bond angles, and torsion angles (deg) quoted in the legends. Details of data collection and refinement are given in Table S1. The complexes crystallized in the monoclinic space groups *C*2/*c*, *P*2_1_/*c*, *P*2_1_/*c*, and non-centrosymmetric triclinic *P*1, respectively.

The coordination geometry of Cu(II) in **[CuCl(L^1^)(DMF)]** (Figure 1a) is four-coordinate square-planar, even though very weak coordination of DMF molecule can be considered, taking into account the apical position of oxygen atom O1 with respect to the basal plane determined by the metal ion, the coordinated three nitrogen donor atoms, and the chlorido co-ligand. In this latter case, the coordination geometry can be interpreted as 4 + 1 binding. Comparison with coordination geometry in **[CuCl(L^2^)(CH_3_OH)]** (Figure 1b), which is best described as five-coordinate square-pyramidal, and bond lengths around copper(II), which are significantly expanded when compared to those in **[CuCl(L^1^)(DMF)]**, provides further evidence for a more appropriate description of coordination geometry in **[CuCl(L^1^)(DMF)]** as four-coordinate. The increase in coordination number in **[CuCl(L^2^)(CH_3_OH)]** to five leads to a significant expanding of interatomic distances between Cu(II) and donor atoms due to increase in interatomic repulsions. The latonduine backbone in both complexes has almost identical folding due to the presence of an sp^3^-hybridized carbon atom in the seven-membered azepine ring. The torsion angle Θ_C4a–C5–N6–C7_ is almost the same in both complexes (see the values quoted in the legends to Figures 1a,b).

Complex **[CuCl(L^3^)]** forms a weak dimeric associate (Figure S2), in which the coordination environment of Cu1 can be described as slightly distorted square-planar (see also Figure 1c). The atom Cl1 acts as a bridging μ-chlorido co-ligand to Cu2 of the second half of the dimeric associate with formation of a long contact of 2.8970(8) Å. Therefore, the coordination geometry of Cu2 can be described as 4 + 1 as was the case for complex **[CuCl(L^1^)(DMF)]**. The latonduine derivatives adopt the same binding mode to both Cu(II) atoms Cu1 and Cu2, and each acts as a monoanionic tridentate ligand. The bond lengths in each chromophore of the two Cu(II) ions are very similar to those in **[CuCl(L^1^)(DMF)]**, in accordance with small structural difference between the two coordinated ligands (**L^1^**)^−^ and (**L^3^**)^−^.

In contrast to Cu(II) compounds with strongly folded indolo[2,3-*d*]benzazepine backbone **E** (**[CuCl(L^1^)(DMF)]·DMF**, **[CuCl(L^2^)(CH_3_OH)]**, and **[CuCl(L^3^)]·0.5H_2_O**), Cu(II) in **[CuCl_2_(H_2_L^5^)]Cl·2DMF** is coordinated by a flat indolo[2,3-*c*]quinoline-based tridentate ligand protonated at the nitrogen atom N1 of the morpholine moiety. The positive global charge of the complex cation (Figure 1d) is counterbalanced by a chloride anion. The brominated backbone in the metal-free ligand **HL^6^** is also flattened and stabilized by two intramolecular H-bonding interactions (Figure S3) N7–H···N13 [N7···N13 = 2.725(3) Å; ∠N7HN13 = 122(2)°] and N12–H···N16 [N12···N16 = 2.751(3) Å; ∠N12HN16 = 131(2)°]. The coordination geometry in **[CuCl_2_(H_2_L^5^)]**^+^ is five-coordinate and can be described as intermediate (τ_5_ = 0.40) between square-pyramidal (τ_5_ = 0) and trigonal bipyramidal (τ_5_ = 1).^54^

The indolo[2,3-*d*]benzazepine backbone in **[CuCl(L^1^)(DMF)]·DMF**, **[CuCl(L^2^)(CH_3_OH)]**, and **[CuCl(L^3^)]·0.5H_2_O** is folded due to the presence of one sp^3^-hybridized carbon atom in the seven-membered azepine ring. The dihedral angles between the mean plane through C1–C2–C3–C4–C4a–C12c and Cu–N6–C7–N13–N14 are of 121.5, 108.3, and 112.3° for **[CuCl(L^1^)(DMF)]·DMF**, **[CuCl(L^2^)(CH_3_OH)]**, and **[CuCl(L^3^)]·0.5H_2_O**, respectively. These are larger than those in copper(II) complexes with closely related Schiff bases based on indolo[3,2-*d*]benzazepine (paullone), reported previously of 99.6–102.2° (see Chart S3 in the Supporting Information).^33^ The presence of a six-membered pyridine-like ring in **[CuCl_2_(H_2_L^5^)]**^+^ and **HL^6^** instead of a seven-membered azepine ring makes these indoloquinoline systems flat, a premise to intercalate into DNA. The SC-XRD studies revealed that **HL^1^**–**HL^3^** and **HL^5^** act as tridentate ligands but adopt different protonation states depending on conditions. Therefore, it was of interest to study the solubility of metal-free ligands and Cu(II) complexes as well as their protonation state at physiological pH.

### 2.4 Solubility Studies

**Figure 2:**
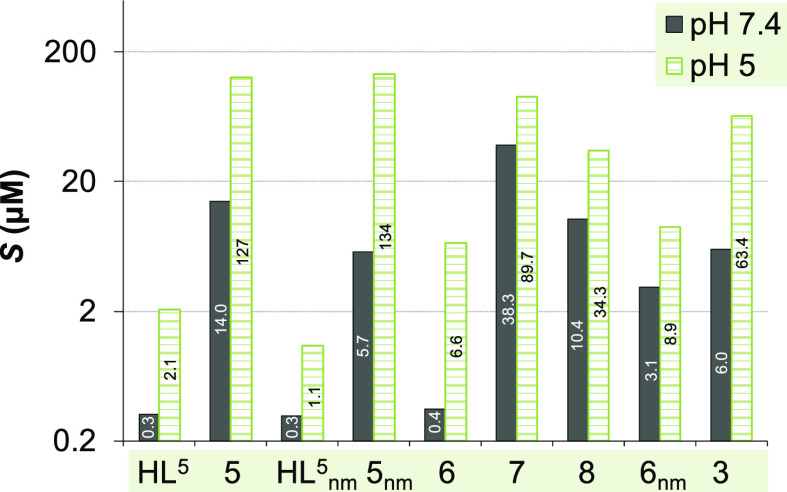
Thermodynamic solubility (*S*) determined for selected metal-free ligands and complexes using UV–vis spectral analysis at pH 5 (20 mM MES buffer) and 7.4 (20 mM HEPES) in water (*T* = 298 K).

The thermodynamic solubility of selected metal-free ligands and Cu(II) complexes was characterized in water at pH 7.4 and 5.0 using UV–vis spectrophotometry for the determination of the concentration of the saturated solutions. The determined solubility (*S*) values are shown in Figure 2.

The obtained solubility values indicate that the copper(II) complexes are commonly more soluble in water than their corresponding metal-free ligands. The positive effect of the morpholine moiety on the solubility is measurable but minor. Comparison of the *S* values for the **HL^5^** and **HL^5^_nm_**, **5** and **5_nm_**, and **6** and **6_nm_** pairs under the conditions used indicates that all compounds have significantly better solubility at pH 5 than at pH 7.4. For **HL^5^** and **HL^5^_nm_**, this can be explained by the partial protonation of the morpholine nitrogen and pyridine nitrogen with the decreasing pH. The morpholine-containing complexes (**3** and **5–8**) also can get partially protonated at the non-coordinating morpholine nitrogen when the pH is lowered. However, formation of the aqua complex from the mixed hydroxido species (*vide infra*) can also contribute to the increased solubility even for the non-morpholine complexes at pH 5. The presence of the bromo-substituent (**5***vs***6**, **5_nm_***vs***6_nm_**, and **7***vs***8**) results in a considerable decrease of the aqueous solubility at both tested pH values, while the effect of the methyl group at the Schiff base ketimine bond is not significant.

### 2.5 Solution Equilibrium Studies

**Chart 3:**
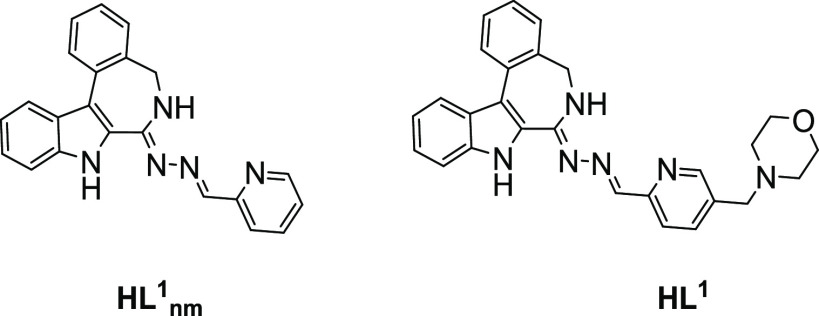
Metal-Free Ligands **HL^1^** and **HL^1^_nm_** Used for the Solution Equilibrium Studies

**Figure 3:**
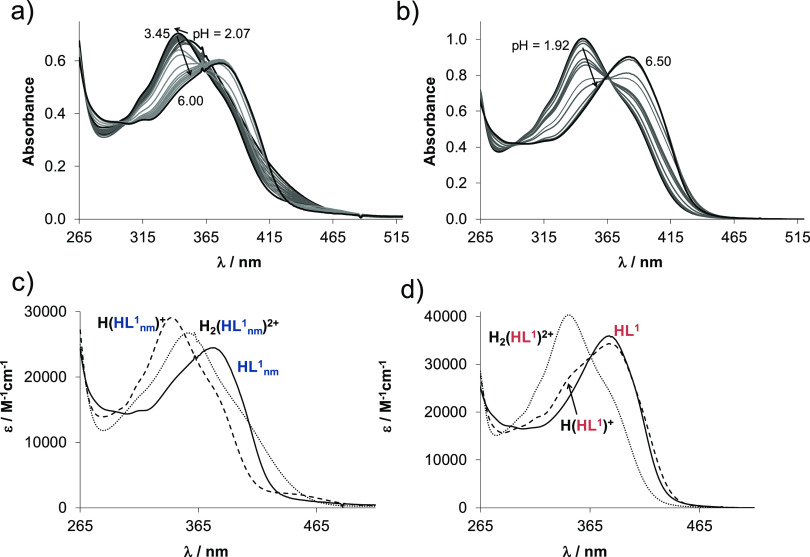
UV–vis spectra for (a) **HL^1^_nm_** and (b) **HL^1^** at various pH values in a 30–70% (w/w) DMSO–water solvent mixture in addition to the computed molar absorbance spectra of the proligand species in the different protonation states: (c) **HL^1^_nm_** and (d) **HL^1^**. (*c*_ligand_ = 12.5 μM (a), 50 μM (b); *T* = 298 K; *I* = 0.10 M (KCl); l = 2 cm (a), 0.5 cm (b)).

The protonation processes of **HL^1^_nm_** and its morpholine counterpart **HL^1^** (Chart 3) and the solution stability of their copper(II) complexes (**1_nm_** and **1**) were characterized by UV–vis spectrophotometry. Since the organic compounds and their copper(II) complexes possess limited aqueous solubility, the solution equilibrium studies were performed in 30% (w/w) DMSO at low concentrations (12.5 μM (**HL^1^_nm_**) or 50 μM (**HL^1^**)). Based on the characteristic changes in the UV–vis spectra for **HL^1^_nm_** in the pH range 2–6 (Figure 3a), two relatively well-separated proton dissociation processes were observed and their p*K*_a_ values were determined (Table 1).

**Table 1:** Proton Dissociation Constants (p*K*_a_) of **HL^1^_nm_** and **HL^1^**, along with Overall Stability Constants (log β) of Their Copper(II) Complexes Determined by UV–vis Titrations in a 30–70% (w/w) DMSO–Water Solvent Mixture (*T* = 298 K; *I* = 0.10 M (KCl))

	constant			constant
p*K*_a1_ H_2_(**HL^1^_nm_**)^2+^	2.32 ± 0.01		p*K*_a1_ H_3_(**HL^1^**)^3+^	<2
p*K*_a2_ H(**HL^1^_nm_**)^+^	4.75 ± 0.01		p*K*_a2_ H_2_(**HL^1^**)^2+^	4.53 ± 0.02
			p*K*_a3_ H(**HL^1^**)^+^	5.65 ± 0.02
log β [Cu(**HL^1^_nm_**)]^2+^	8.33 ± 0.01		log β [CuH(**HL^1^**)]^3+^	12.38 ± 0.01
log β [Cu(**L^1^_nm_**)]^+^	4.01 ± 0.01		log β [CuH(**L^1^**)]^2+^	8.57 ± 0.01
log β [Cu(**L^1^_nm_**)(OH)]	–3.1 ± 0.1		log β [Cu(**L^1^**)]^+^^a^	2.9 ± 0.1
p*K*_a_ [Cu(**HL^1^_nm_**)]^2+^	4.32		p*K*_a_ [CuH(**HL^1^**)]^3+^	3.81
p*K*_a_ [Cu(**L^1^_nm_**)]^+^	7.09		p*K*_a_ [CuH(**L^1^**)]^2+^^a^	5.71

a Formation of [Cu(**L^1^**)]^+^ and [CuH(**L^1^**)(OH)]^+^ is overlapping; the two species cannot be well distinguished.

**Scheme 4:**
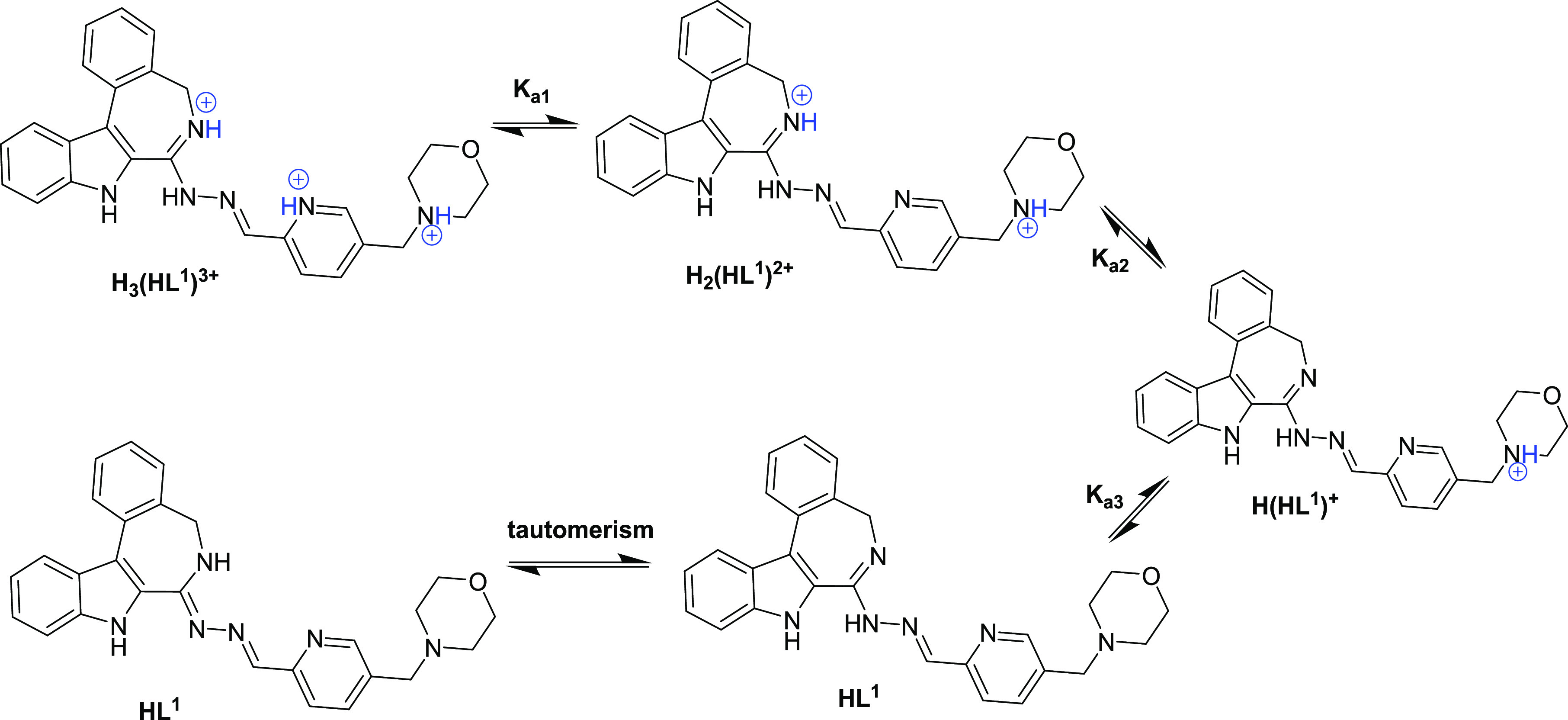
Stepwise Proton Dissociation Processes of **HL^1^** and Its Tautomeric Forms

Notably, upon increasing the pH to >∼6.6 precipitation occurred in the solution, leading to the elevation of the base line most probably due to the formation of the neutral metal-free ligand species. The first proton dissociation step was accompanied by a blueshift (λ_max_: 352 nm → 342 nm) in the pH range between 2 and 3.45, while the λ_max_ is redshifted (342 nm → 377 nm) upon the second step. These spectral changes and the spectra of the individual ligand species (Figure 3b) calculated by deconvolution of the recorded UV–vis spectra are fairly similar to those found for the ketimine derivative of **HL^1^_nm_** in our recent work.^43^ Thus, a similar deprotonation pattern is feasible for the non-substituted and the methyl-substituted ligands. The neutral species **HL^1^_nm_** can be present in two tautomeric forms (due to the rearrangement of the N=C–NH–N and NH–C=N–N bonds) and can be protonated at two sites (Scheme 4). Therefore, the first proton dissociation step (p*K*_a1_) is attributed to deprotonation of the pyridinium nitrogen, while the second process (p*K*_a2_) is attributed to the deprotonation of the benzazepinium nitrogen. The ligand **HL^1^_nm_** possesses somewhat lower p*K*_a_ values than its methyl derivative as a result of the electron-donating property of the methyl group.

Two p*K*_a_ values were determined for **HL^1^** as well (Table 1) by the deconvolution of the recorded spectra (Figure 3b), although the spectral changes were different. This ligand contains also the morpholinium group (Scheme 4). By the careful analysis of the spectral changes, it is suggested that the deprotonation of the pyridinium nitrogen takes place at fairly acidic pH, and its p*K*_a_ value is lower compared to that of **HL^1^_nm_** as a result of the electron-withdrawing effect of the protonated morpholinium moiety. Even though the deprotonation of the benzazepinium and morpholinium nitrogens is overlapping, the spectra of the individual species (Figure 3d) suggest that p*K*_a2_ mostly belongs to the benzazepinium moiety, and the deprotonation of the non-chromophoric morpholinium nitrogen is accompanied by a minor spectral change, as expected.

The p*K*_a_ values collected in Table 1 indicate that the neutral form (**HL**) predominates at physiological pH in solution in the case of both ligands, contributing to their strong lipophilic nature (log *D*_7.4_ values +4.75 (**HL^1^_nm_**) and +4.30 (**HL^1^**) estimated by the MarvinSketch program.^55^

**Figure 4:**
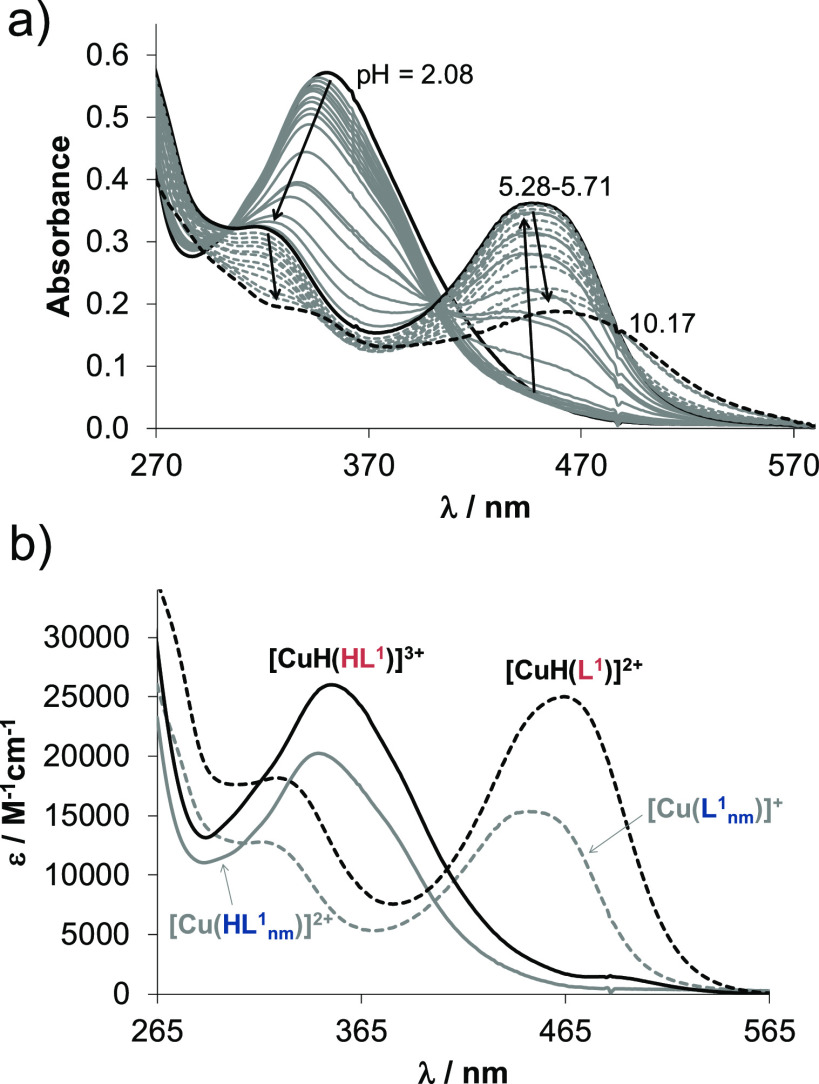
(a) UV–vis spectra recorded for complex **1_nm_** at various pH values. (b) Molar absorbance spectra computed for selected complex **1_nm_** (black lines) and **1** (gray lines) in the various protonation states. (*c*_complex_ = 12.5 μM; *T* = 298 K; *I* = 0.10 M (KCl); 30% (w/w) DMSO–H_2_O).

The UV–vis spectra recorded for **1_nm_** in the pH range 2–11 (Figure 4a) showed strong similarities to those of the methylated complex reported recently.^43^ The spectrum measured at pH 2 suggests a considerable extent of complex formation. The tridentate coordination via the N,N,N donor set is assumed in **[Cu(HL^1^_nm_)]^2+^** at such low pH. Increase in the pH is accompanied by λ_max_ shift to a lower wavelength (345 nm → 316 nm) parallel with the development of a new band at 449 nm, suggesting a significant rearrangement in the coordination sphere. The deprotonation of the non-coordinating hydrazonic nitrogen is likely, and complex **[Cu(L^1^_nm_)]^+^** is formed. Further increase in the pH (pH > 7) resulted in a bathochromic shift (λ_max_ ≈ 462 nm), indicating a new process, namely the formation of the neutral mixed hydroxido species **[Cu(L^1^_nm_)(OH)]**. However, the absorbance is decreased in the whole wavelength range due to precipitate formation. By the deconvolution of the recorded UV–vis spectra, overall stability constants for the complexes **[Cu(HL^1^_nm_)]^2+^** and **[Cu(L^1^_nm_)]^+^** were computed, and log β for **[Cu(L^1^_nm_)(OH)]** was only estimated (Table 1). In the case of the morpholine hybrid, a somewhat different speciation was obtained as the presence of the pendant morpholine moiety had to be taken into consideration. Log β values for **[CuH(HL^1^)]^3+^**, **[CuH(L^1^)]^2+^**, and **[Cu(L^1^)]^+^** were computed from the spectrophotometric titration data (Table 1). Since the molar absorbance spectra of **[CuH(HL^1^)]^3+^** and **[CuH(L^1^)]^2+^** complexes resemble those of **[Cu(HL^1^_nm_)]^2+^** and **[Cu(L^1^_nm_)]^+^** (Figure 4b), the same coordination modes are likely in these species in couples. Therefore, the deprotonation of **[CuH(HL^1^)]^3+^** takes place most probably at the hydrazinic nitrogen (characterized by a p*K*_a_ of 3.81) and the morpholinium nitrogen remains protonated. Increasing the pH (pH > 5), the absorbance is decreased in the whole wavelength range, no isosbestic points are found (indicating the formation of some precipitate), while the λ_max_ is increased. Formation of a mixed hydroxido species **[CuH(L^1^)(OH)]^+^** is also possible. However, the deprotonation of the morpholinium group can take place as well (formation of **[Cu(L^1^)]^+^**) in this pH range. As these two processes are overlapping, the two species could not be well distinguished, and the obtained stability constant is quite uncertain.

**Figure 5:**
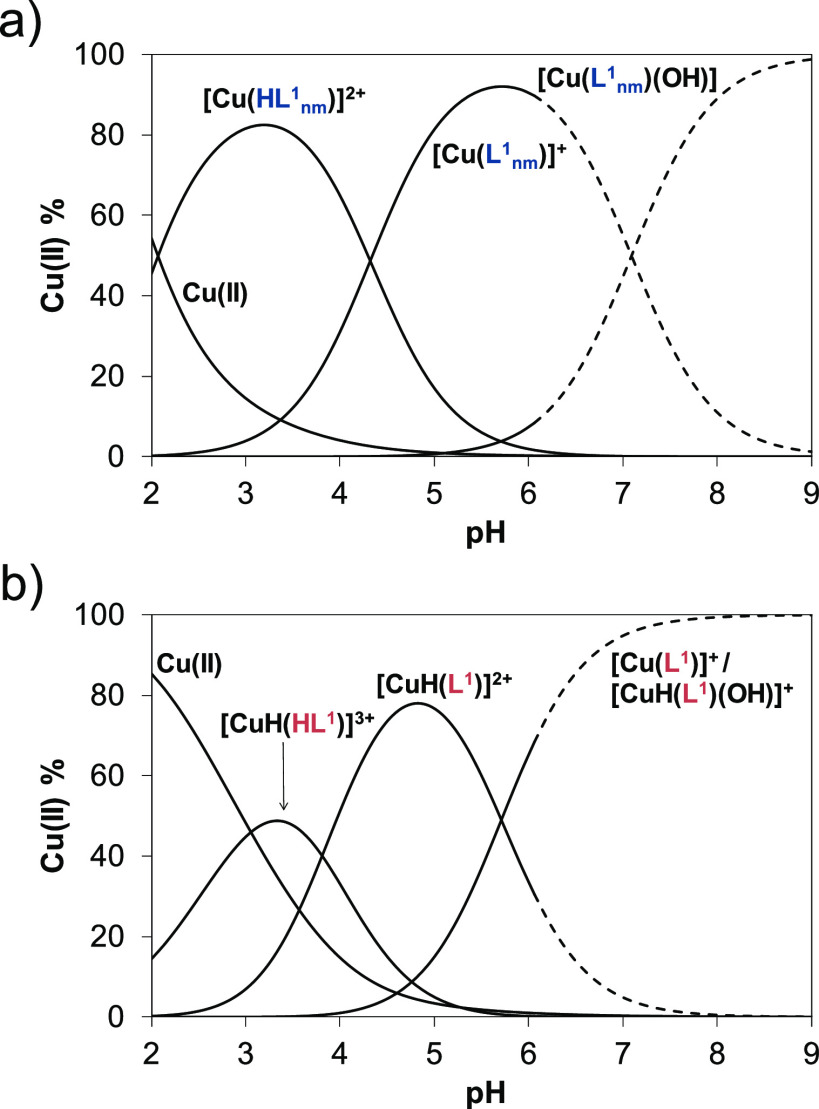
Concentration distribution curves for complexes (a) **1_nm_** and (b) **1** plotted against the pH. (Dashed lines show the region where precipitate appears.) (*c*_complex_ = 12.5 μM; *T* = 298 K; *I* = 0.10 M (KCl); 30% (w/w) DMSO–H_2_O).

Using the determined stability constants, concentration distribution curves were computed (Figure 5) for both copper(II)–ligand systems. The results imply that the complexes do not dissociate at the used 12.5 μM concertation at neutral pH (the fraction of the free metal ion is negligible). However, the concentration of the free metal ion is significantly higher in the acidic pH range than it was found for the methylated complex, suggesting the somewhat lower stability of **1_nm_** and **1**. Notably, while the ketimine derivative of **1_nm_** (or **3_nm_** in terms of nomenclature used herein) was identified as the predominant species at neutral pH,^43^ for **1_nm_** and **1**, formation of considerable amounts of mixed hydroxido species is also suggested (Figure 5).

### 2.6 Stability of Selected Compounds in a Buffered Medium and Blood Serum

Prior to the biological assays, the aqueous stability of selected compounds (**HL^4^**, **4**, **HL^8^**, and **8**) was measured as a function of time by UV–vis spectrophotometry at pH = 7.40 in 10 mM HEPES in PBS and in blood serum diluted by factor 3 (dilutions were made in HEPES and in PBS as well). In the buffer solutions, slow precipitation of the compounds was observed as shown for **4** in Figure S5 in the Supporting Information. This process took several hours, and it was in all cases less pronounced in the PBS buffer than in the HEPES medium.

Measurements with diluted serum show a more elaborate picture. As can be seen in Figure S6, complex **8** appears to react with serum components in a fast process and then slow precipitation and a second type of interaction become dominant. The first process (0.1–13 min) has only moderate effect on the spectral properties of the complex, and the *N*, *N*, *N* coordination sphere is not altered, while slow development of a new band at 400 nm indicates partial decomposition of the complex. The new band cannot be undoubtedly attributed to the liberation of ligand **HL^8^**. For complex **4**, a similar behavior was observed, even though changes were smaller. Interestingly, **HL^4^** itself interacts with serum components (Figure S7), and complex formation with metal ions (i.e., Zn(II), Cu(II), and Fe(III)) can be supposed as well.

### 2.7 Lead Morpholine-Indolo[2,3-*c*]quinolone and Latonduine Derivatives as well as Their Cu(II) Complexes Exhibit Antiproliferative Activity in a Sub-micromolar Concentration Range and Trigger Apoptosis

The *in vitro* antiproliferative activity of the compounds was tested in doxorubicin-sensitive Colo205 and multidrug-resistant Colo320 human colon adenocarcinoma cell lines as well as in normal human embryonal lung fibroblast cells (MRC-5) by MTT assay. As seen in Table 2, the morpholine-hybrid ligands **HL^1^**–**HL^8^** and their Cu(II) complexes **1**–**8** exhibit IC_50_ values in the low micromolar to the sub-micromolar range.

**Table 2:** IC_50_ Values for Morpholine-Indolo[2,3-*c*]quinoline and Latonduine Derivatives **HL^*1*^**–**HL^*8*^** and Their Copper(II) Complexes **1**–**8** as well as for the Ligand and Copper(II) Complex **HL^4^_nm_** and **4_nm_**. Selectivity factors (SF) for Colo205 and Colo320 cancer cell lines over non-cancerous MRC-5 cells. SF(Colo205) = IC_50_ MRC-5/IC_50_ Colo205, SF(Colo320) = IC_50_ MRC-5/IC_50_ Colo320

	IC_50_ (μM)							
compound	Colo320 resistant		Colo205 sensitive		MRC-5 benign		selectivity factor (SF)	
	mean	SD	mean	SD	mean	SD	Colo320	Colo205
HL^**1**^	8.32	0.81	2.545	0.058	3.02	0.23	0.36	1.19
1	2.29	0.19	1.425	0.047	1.90	0.16	0.83	1.33
HL^**2**^	4.80	0.48	2.83	0.46	4.86	0.32	1.01	1.72
2	3.19	0.45	2.74	0.12	5.52	0.38	1.73	2.01
HL^**3**^	1.41	0.20	0.372	0.099	1.13	0.17	0.80	3.04
3	0.335	0.053	0.23	0.042	0.413	0.03	1.23	1.80
HL^**4**^	1.34	0.38	0.267	0.01	0.374	0.025	0.28	1.40
4	0.109	0.008	0.22	0.01	0.397	0.029	3.64	1.80
HL^4^_nm_	8.19	0.60	0.091	0.004	0.111	0.009	0.01	1.22
4_nm_	0.350	0.037	0.083	0.010	0.19	0.04	0.54	2.29
HL^**5**^	2.08	0.13	1.40	0.19	0.615	0.054	0.30	0.44
5	2.64	0.17	2.09	0.13	2.30	0.13	0.87	1.10
HL^**6**^	1.04	0.12	1.03	0.12	0.043	0.010	0.04	0.04
6	0.851	0.056	1.087	0.034	0.221	0.014	0.26	0.20
HL^**7**^	0.392	0.025	0.123	0.012	2.83	0.52	7.22	23.0
7	0.149	0.007	0.098	0.007	0.652	0.063	4.38	6.65
[Ni(HL^**7**^)_2_]Cl_2_	40.4	2.1	72.6	4.7	52.80	0.78	1.31	0.73
HL^**8**^	4.07	0.07	0.016	0.002	1.066	0.051	0.26	68.8
8	0.547	0.003	0.038	0.002	0.165	0.013	0.30	4.38
doxorubicin	5.43	0.94	0.712	0.021	11.0	0.2	2.03	15.44

The summarized IC_50_ values indicate that the indolo[2,3-*d*]benzazepine derivatives (**HL^1^**–**HL^4^** and **1**–**4**) are generally less cytotoxic than the analogous indolo[2,3-*c*]quinoline compounds (**HL^5^**–**HL^8^** and **5**–**8**). This observation correlates well with the structural peculiarities of both families of compounds, i.e., better potential ability of flat indolo[2,3-*c*]quinolines and their metal complexes (**HL^5^**–**HL^8^** and **5**–**8**) to intercalate into DNA than that of the folded indolo[2,3-*d*]benzazepine derivatives (**HL^1^**–**HL^4^** and **1**–**4**). Another general trend was observed in our recent work,^43^ namely that methyl group at positions 15 and 14, respectively (see Chart S2), has a favorable effect on both the selectivity and cytotoxicity. Comparison of the IC_50_ values for the methylated and non-methylated pairs (**1** and **3**, and **5** and **7**) indicates little improvement of selectivity, while the cytotoxicity is enhanced markedly upon the methylation. Methylation of metal-free ligands can slightly improve the electron-donating abilities of the indolobenzazepines and indoloquinolines as ligands and increase their chelating ability with respect to copper(II). It has been established recently^56^ that higher copper(II) complex stability in the case of thiosemicarbazones correlates well with increased anticancer activity. Comparison of the stability constants of copper(II) complexes of **HL^1^_nm_** (log β [Cu(HL^1^_nm_)]^2+^ = 8.33; log β [Cu(L^1^_nm_)]^2+^ = 4.01) with copper(II) complexes of **HL^3^_nm_** (log β [Cu(HL^3^_nm_)]^2+^ = 10.96; log β [Cu(L^1^_nm_)]^2+^ = 6.39)^43^ indicates that methylation at the Schiff base azomethine bond increases markedly the stability constants and is in agreement with the enhancement of antiproliferative activity.

The bromo-substituent brings much smaller changes in the cytotoxicity but enhances selectivity for cancer cells in the case of methylated (ketimine) Schiff bases.

An additional trend can be seen when comparing the IC_50_ values of metal-free ligands **HL^1^**–**HL^8^** with their respective copper(II) complexes **1**–**8**. Upon complex formation, the IC_50_ values generally decrease, showing a positive effect on the cytotoxic behavior of the organic molecules in both cancer cell lines except for the **HL^5^**/**5** pair. The nickel(II) complex **[Ni(HL^7^)_2_]Cl_2_** showed inferior cytotoxicity when compared to compound **7** and other copper(II) complexes tested. Even though the stoichiometry of copper(II) complexes and nickel(II) complexes is different, we assume that not only the stoichiometry has an effect on cytotoxicity but also the metal ion identity. Most of the proligands as well as their copper(II) complexes (except **HL^5^**, **HL^6^**, and **6**) show selectivity toward the doxorubicin-sensitive Colo205 cells over the normal cells (selectivity factor (SF) > 1), while the SF values were smaller with regard to the resistant Colo320 cell line. Notably, the **HL^7^**/**7** pair in both cell lines and the **HL^8^**/**8** pair in the case of Colo205 display very good selectivity. An additional positive effect of complex formation with copper(II), besides the increase in cytotoxicity, is the generally increased selectivity of the complexes toward the cancer cells. Therefore, upon complex formation with copper(II), a marked enhancement of the pharmacological profile is noticed. In all, complexes **4** and **7** are not only characterized by lower IC_50_ values on both malign cell lines than their corresponding metal-free ligands and the reference compound doxorubicin but are also selective. Additionally, complex **4** is found to be somewhat more cytotoxic against the resistant Colo320 cells in comparison to the sensitive cells, which is another noteworthy feature.

**Chart 4:**
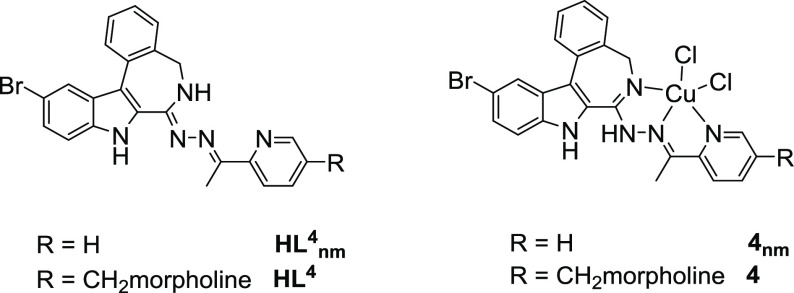
Structures of **HL^4^_nm_** and **4_nm_**(43) and **HL^4^** and **4**

The influence of the morpholine moiety on both cytotoxicity and selectivity was assessed by comparison of the cytotoxicity for the metal-free ligands **HL^4^** and **HL^4^_nm_** and complexes **4** and **4_nm_** (Chart 4).

While the cytotoxicity of **HL^4^_nm_** and **4_nm_** in Colo205 cells exceeds that of the respective morpholine-bearing molecules **HL^4^** and **4** by a factor of *ca.* 3, this trend is inverted for Colo320 cells. Interestingly, the increase in the cytotoxicity in the Colo205 cells upon the complex formation is much smaller for both ligands in comparison to the Colo320 cells. The morpholine-bearing complex **4** is more active than **4_nm_** in Colo320 cells.

**Figure 6:**
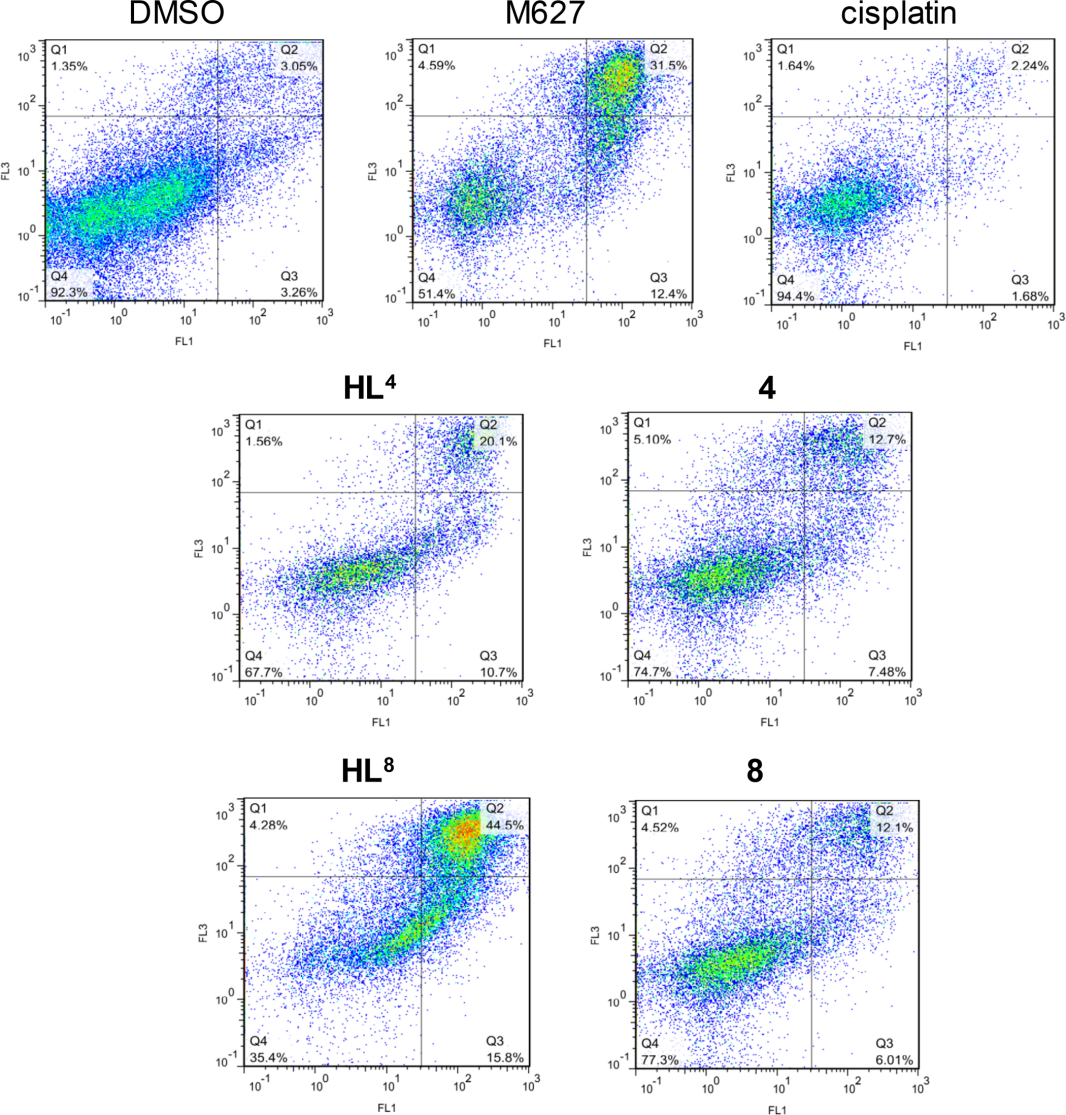
Quantification of apoptosis in Colo320 cells treated with **HL^4^**, **4**, **HL^8^**, **8**, M627, cisplatin (as positive controls), and DMSO (0.8% (v/v)) using the Annexin-V/PI double staining assay at 2 and 4 μM (**HL^8^** and **HL^4^**), 0.5 and 2 μM (**8**), and 0.25 and 0.5 μM (**4**) concentrations, respectively. Colo320 cells were treated at the indicated concentration of the compounds. The dual parametric dot plots combining Annexin-V (FL1) and PI (FL3) fluorescence show the viable cell population in the lower left quadrant Annexin-V–/PI– (**Q4**), the early apoptotic cells in the lower right quadrant Annexin-V+/PI– (**Q3**), and the late apoptotic and necrotic cells in the upper right quadrant Annexin-V+/PI+ (**Q2**). (Number of cells counted: 23,193 (M627), 20,262 (cisplatin), 13,391 (**HL^4^**), 22,309 (**4**), 27,966 (**HL^8^**), and 23,468 (**8**)).

The lead compound **8** and its metal-free ligand **HL^8^**, as well as their indolo[2,3-*d*]benzazepine analogues (**4** and **HL^4^**), were further investigated to elucidate the cytotoxic mechanism. An apoptosis assay was performed by flow cytometry via the analysis of multidrug-resistant Colo320 cells stained with Annexin-V-FITC and propidium iodide (PI). The compounds were tested at two concentrations in the range of their IC_50_ values, and 12*H*-benzophenothiazine (M627) and cisplatin were used as positive controls. Apoptosis is a form of programmed cell death, which is the preferred mode of action for an anticancer drug.^57^ The fluorescence of PI (FL3) was plotted versus Annexin-V fluorescence (FL1) as shown in Figure 6 for the positive controls, for DMSO, and for the tested compounds. The percentage of the gated events regarding the early apoptosis, the late apoptosis and necrosis, and cell death is quoted in Table S2. These data revealed that all four tested compounds (**HL^4^**, **4**, **HL^8^**, and **8**) could trigger apoptosis in Colo320 cells more efficiently than cisplatin. Of note is the high percentage of early apoptotic (10.7%) and late apoptotic and necrotic cells (20.1%) for **HL^4^** at 2 μM, which further increases for **HL^8^** to 15.8 and 44.5%, respectively. A high population of apoptotic and necrotic cells is also observed for complex **4** (12.7%) at 0.25 μM and for complex **8** (12.1%) at 0.5 μM.

The interaction of lead drug candidates with DNA was further studied to reveal peculiarities in their behavior.

### 2.8 Lead Cu(II) Complex **8** Interacts with Calf Thymus (ct)-DNA More Effectively than Complex 4

The interaction of **4** and **8** and **HL^4^** and **HL^8^** with ct-DNA was investigated by spectrofluorometry in ethidium bromide (EtBr) displacement studies. EtBr is a fluorescent probe, and its fluorescence intensity increases upon intercalation into the DNA helix. The ligands did not affect the fluorescence of the EtBr–ct-DNA system (Figure S8). Addition of complexes, however, decreased the emission intensity. Interestingly, the solubility of **8** (which was low) increased significantly in the presence of ct-DNA, and this complex reduced most significantly the fluorescence of EtBr. Decrease in the fluorescence may indicate (i) the displacement of EtBr or (ii) (partial) quenching of the fluorescence of the bound probe. In order to separate these processes, fluorescence lifetime measurements were carried out. These experiments indicated that the decrease in intensity is due to both EtBr displacement and alterations in the close environment of the intercalated EtBr (see Figure S9 in the Supporting Information). In addition, they provided evidence that the two copper(II) complexes bind to ct-DNA. However, complex **8** replaced EtBr more effectively than **4**. To get further insight into the mechanism of action of lead compounds, their antiproliferative activity in wild-type cells HCT116 and HCT116 cell subline with knocked out *p53* gene was investigated.

### 2.9 Is DNA a Crucial Target for Lead Drug Candidates **HL^4^**, **HL^8^**, **4**, and **8**?

The oncosuppressor protein *p53* controls the cellular response to DNA strand breaks induced by cytotoxic drugs or by radiation.^58^ The *p53* protein may enhance cell chemosensitivity by promoting apoptosis via different mechanisms including activation of proapoptotic genes such as *bax* and repression of antiapoptotic genes such as *bcl-2* or in contrast increase chemoresistance by promoting *p21*-mediated and *p21*-independent growth arrest and DNA repair and by activation of antiapoptotic genes such as *bcl-*x. There is strong evidence that the modulation of drug sensitivity by *p53* may be both drug- and cell type-specific.^59^ Targeted *p53* inactivation in human cancer cells was shown to enhance their chemosensitivity to the drugs able to induce DNA strand breaks such as doxorubicin but at the same time make them quite resistant to drugs with other nucleic-acid-related mechanisms of action, e.g., 5-fluorouracil (5-FU). Accordingly, essential differences in chemosensitivity were observed between cells with wild-type *p53* gene and cells with knocked out *p53* gene by homologous recombination.^60^ Based on this knowledge, we used two isogenic cell lines, namely the wild-type HCT116 and HCT116 cell line with knocked out *p53* gene, and treated them with our lead compounds as well as by cisplatin used as a DNA-damaging (positive control) drug. The results of these MTT assays summarized in Table 3 clearly show that the sensitivity of a *p53*-deficient HCT116 subline toward compounds **HL^4^**, **HL^8^**, **4**, and **8** remains intact when compared to wild-type cells with proficient *p53* gene. The sensitivity data are in strong contrast with the response of the cells to DNA cross-linking drug cisplatin, which showed lowered cytotoxicity in the subline with knocked out *p53* gene. The data obtained strongly suggest that DNA is not a crucial target for the lead drug candidates evaluated in this study.

**Table 3:** IC_50_ Values of Lead Drug Candidates in HCT-116 Colon Carcinoma Cells and an Isogenic p53-Knock-Out Subline

	IC_50_ [μM]			
compound	HCT-116		HCT-116p53ko	
	mean	SD	mean	SD
**HL^4^**	0.14	0.02	0.12	0.02
**4**	0.13	0.05	0.13	0.03
**HL^8^**	0.042	0.003	0.038	0.008
**8**	0.050	0.005	0.041	0.006
cisplatin	0.78	0.27	1.7	0.4

Among other possible mechanisms underlying the antiproliferative activity of the lead drug candidates, kinase inhibition was further considered.

### 2.10 Cu(II) Complex Formation Changes Dramatically the Kinase Inhibition Profile in a Panel of 50 Kinases

**Figure 7:**
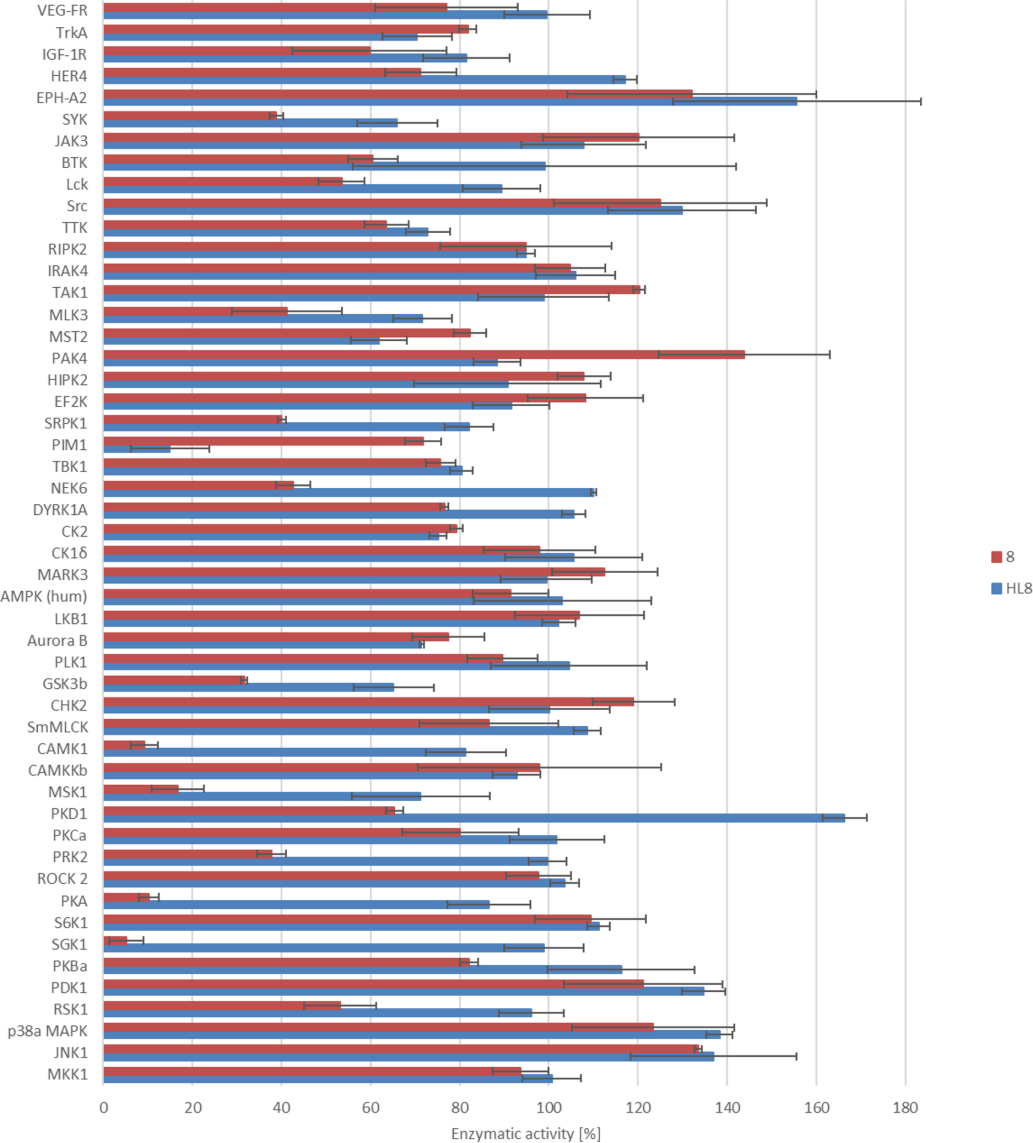
Screening of **HL^8^** and **8** against 50 enzymes: histogram with % remaining activity on the *x*-axis plotted against the compound identifier (blue trace: **HL^8^** and red trace: **8**) and enzyme identifier on the *x*-axis. The screen was carried out at a compound concentration of 10 μM, an enzyme concentration of 50 nM, and an ATP concentration of 100 μM.

The two lead compounds, **HL^8^** and **8**, showing the highest cytotoxicity with IC_50_ values in the sub-micromolar concentration range and selectivity for the Colo205 cell line (see Table 2), were submitted to the International Centre for Kinase Profiling at Dundee University and screened against 50 enzymes using an inhibitor concentration of 10 μM. Figure 7 (see also Table S4) summarizes the results of this assay as a histogram plotting the percentage of the remaining enzyme activity (*x*-axis) as a function of added lead compound (**HL^8^**: blue trace, **8**: red trace) for each of the 50 enzymes assayed (*y*-axis). These data revealed fully distinct enzyme inhibitory patterns and selectivity for **HL^8^** and its complex **8**. **HL^8^** showed good selectivity and notable potency for one of the 50 kinases assayed, namely for the serine and threonine protein kinase PIM-1, while **8** significantly inhibited (below 68% of original activity) the activity of five enzymes, namely of serum and glucocorticoid-regulated kinase SGK-1, cAMP-dependent protein kinase PKA, calcium/calmodulin-dependent protein kinase CaMK-1, mitogen stress-activated kinase MSK1, and glycogen synthase kinase GSK3β. Thus, the coordination to copper(II) completely changes the kinase inhibition profile of **HL^8^**.

A closer look at these proteins reveals common features. All of these are serine/threonine-protein kinases, which have at least one ATP binding site, indicating a competitive behavior with this molecule. They use ATP to phosphorylate protein residues, e.g., l-serine one.^61−66^ SGK-1 is closely related with cancer growth, survival, and metastasis in a variety of tumors; in these malign tissues, SGK-1 is upregulated.^67−71^ By downregulating this kinase, tumor growth and metastasis can be slowed down or even stopped. PKA is an important kinase often found in mitochondria that is able to modulate the energy household.^72,73^ Since cancer cells have a very high demand for energy, suppression of that enzyme can assist in starving them. MSK1 is related to cancer growth, metastasis, and increased aggressiveness with an overall poor survival for certain types of cancers.^74,75^ By downregulating this protein, the favorable effect of cancer therapy might be enhanced. CaMK-1 is expressed in all tissues but overexpressed in cancers.^76,77^ Furthermore, there is evidence that CaMK-1 impacts chemoresistance in ovarian cancer.^78^ This overexpressed kinase that affects cancer survival and growth by controlling the cell cycle^77^ offers a potent target for anticancer therapy. Upregulation of PIM-1 is directly connected with tumor progression, survival, and even transformation^79−81^ and, therefore, is a good target for chemotherapy. Due to its ability to initiate the transformation of healthy cells to malign cells, it is considered a proto-oncogene. Resveratrol inhibits PIM-1 activity via binding to the ATP pocket, reducing cancer cell proliferation and survival.^82^

Both compounds were screened against the respective enzymes, and IC_50_ values were determined. The IC_50_ values for **8** are in the range of 0.75 μM (CaMK-1), 1.64 μM (GSK3β), 2.97 μM (MSK1), 6.69 μM (PKA), and 8.45 μM (SGK-1), and for **HL^8^** the IC_50_ value against PIM-1 is 1.18 μM (see Table S5 and Figures S10–S15). GSK3β was chosen for determining IC_50_ values since the previously reported paullones showed both *in silico* and *in vitro* inhibition of this enzyme.^22,83,84^

Hence, we are prone to assume that mono-kinase and multi-kinase inhibition is a more plausible underlying mechanism of cytotoxicity for organic lead drug candidate **HL^8^**, and the copper(II) complex **8**. Even though the multikinase inhibitory activity of **HL^8^** is likely, it still has to be confirmed by increasing the panel of available kinases. At the same time inhibition of enzymes might not be the only and also not the main mode of action contributing to cell death for the selected molecules, and other underlying mechanisms might be responsible for the apoptosis. Among other possible targets, tubulin is worthy to be mentioned as some of the indolo[2,3-*d*]benzazepine derivatives were reported to effectively inhibit tubulin polymerization.^30^

To further provide evidence for the potential binding of copper(II) complexes and the respective ligands to PIM-1, CaMK-1, SGK-1, PKA, and GSK3β kinases and, in particular, for the lead drug candidates **HL^8^** and **8**, molecular docking calculations were conducted.

### 2.11 Lead Cu(II) Complexes are Located in the Binding Sites of Specific Kinases

Cu(II) complexes **1**–**8** and their ligands **HL^1^**–**HL^8^** were docked to the binding sites of PIM-1 (PDB ID: 1YXX, resolution: 2.00 Å),^85^ CaMK-1 (PDB ID: 2JAM, resolution: 1.70 Å),^86^ GSK3β (PDB ID: 3I4B, resolution: 2.30 Å),^87^ PKA (PDB ID: 3OF1, resolution: 2.21 Å),^88^ and SGK-1 kinase (PDB ID: 3HDM, resolution: 2.60 Å).^89^ The available X-ray diffraction structures in Protein Data Bank contain co-crystalized ligands, which were removed and redocked into the binding sites of the kinases to test the robustness of the scoring functions used, namely, GoldScore (GS),^90^ ChemScore (CS),^91,92^ ChemPLP (Piecewise Linear Potential),^93^ and ASP (Astex Statistical Potential)^94^ embedded in the GOLD (v2020.2.0) docking algorithm. The predicted poses were overlaid with the co-crystalized ligands, and the root-mean-square deviation (RMSD) was calculated for the heavy atoms. The results are shown in Tables S6–S11 in the Supporting Information. In general, good results were obtained.

Molecular docking with the ligands **HL^1^**–**HL^8^** and copper(II) complexes **1**–**8** showed reasonable scores for the five kinases (see Tables S6–S11 in the Supporting Information), implying potential binding to the respective kinase pockets. In the case of copper(II) complexes, the GS scoring function was used. The PIM-1 scores (Table S7) indicate that the metal complexes and their ligands bind with greater affinity than the co-crystalized ligand LI7. It should be also stressed that **HL^1^**–**HL^8^** showed better scores than the complexes **1**–**8**, i.e., for **8** and **HL^8^**, the metal-free ligand has a considerable better score. This is in line with the experimental IC_50_ value where the ligand **HL^8^** gave reasonable binding (1.18 μM), whereas the Cu(II) complex did not register binding. Table S8 contains the results for the CaMK-1 kinase, and the scores for both the copper(II) complexes and ligands are comparable to those of the co-crystalized ligand J60. Complex **8** has the best score of the Cu(II) complexes, which fits the experimental results in being the most active at least in the Colo205 cell-based assay (see Table 2). The scores for the GSK3β kinase (Table S9) have lower values than the co-crystallized ligand Z48, and similar scores were obtained for both the ligands and copper(II) complexes, but **8** has the best score of the complexes and a considerably better score than its **HL^8^** counterpart. For the PKA enzyme (Table S10), the ligands have better scores than the CMP co-crystallized ligand only for CS, similar to ChemPLP but worse scores for the other functions as well as for the complexes, which fits the modest IC_50_ value of 6.69 μM for **8**. Finally, the results for the SGK-1 kinase (Table S11), the ligands, and the complexes have better scores than the co-crystallized MMG ligand; the complex **8** has a better GS than **HL^8^**.

**Figure 8:**
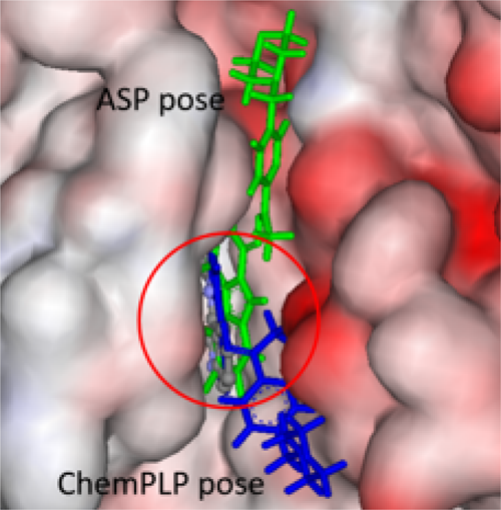
Docked poses of **HL^8^** in the PIM-1’s binding site (PDB ID: 1YXX); the co-crystalized ligand LI7 is shown in ball-and-stick format, and its hydrogens are not shown for clarity (see circled area). The configuration of the ASP prediction is shown in green, and the ChemPLP pose is blue, both are in the stick format. The protein surface is rendered; blue depicts regions with a partial positive charge on the surface, red depicts regions with a partial negative charge, and gray shows neutral areas.

The modeling for PIM-1 revealed that the ligands can adopt two plausible conformations in the binding pocket, whereas the copper(II) complexes are predicted to bind in an unfavorable pose, e.g., complex **8** has its morpholine ring buried within the binding pocket, while its bromine-containing moiety is pointing into the aqueous phase, which is in line with only the **HL^8^** ligand giving good binding results. It is important to note that the metal complexes were docked in their forms shown in Chart 2 (**CuCl_2_(HL)**); however, they are present in their **[Cu(L)]^+^** forms in aqueous solution at pH 7.4 based on the solution equilibrium studies, and the displacement of the coordinated chlorido ligands by the side chain donor atoms of the enzymes is also possible. Thus, the coordinative binding of the complexes to the proteins cannot be excluded. The predicted poses for **HL^8^** are shown in Figure 8, while those for complex **8** are shown in Figure S16. The tetracyclic motif of **HL^8^** for both predicted poses overlap with the co-crystalized ligand LI7 but with the morpholine-containing fragments occupying different clefts on the enzyme’s surface.

**Figure 9:**
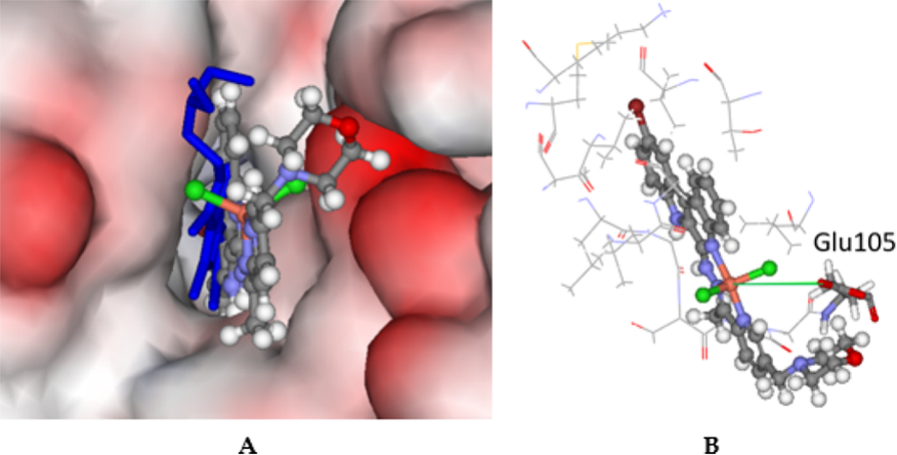
(A) Docked configuration of **8** (ball-and-stick format) in the CaMK-1 binding site (PDB ID: 2JAM) with the co-crystalized ligand J60 colored blue, stick format (hydrogen atoms are hidden for clarity). Extensive overlap is seen between the predicted and experimental configurations, indicating good binding. The protein surface is rendered. Blue depicts regions with a partial positive charge on the surface, red depicts regions with a partial negative charge, and gray shows neutral areas. (B) Predicted binding of complex **8** with amino acid residues within 5 Å shown in line format. The potential chelating amino acid residue Glu105 is shown as sticks. The distance between the proximal oxygen in the carboxylic acid moiety is depicted as a green solid line at 4.7 Å.

On the one hand, the modeling for CaMK-1 using the ligands resulted in several different predicted conformations, indicating poor binding reflected in the experimental binding results. On the other hand, the complexes had consistent pose prediction neatly overlapping the J60 co-crystalized ligand as shown in Figure 9A. The general binding of J60 and **8** is similar in that the chlorine atom of J60 and the bromine atom in **8** are in a similar position, as well as the solubilizing groups of both, i.e., they are pointing into the water environment. This is a strong indication that **8** is tightly bound to CaMK-1 as seen in the IC_50_ value of 0.75 μM. Furthermore, the copper(II) ion may potentially bind to the oxygen atoms in the carboxylic side chain of Glu105 with a 4.7 Å distance between copper(II) and the proximal oxygen atom. The binding is plausible since the side chain of Glu105 is quite flexible with three aliphatic carbon atoms (Figure 9B).

**Figure 10:**
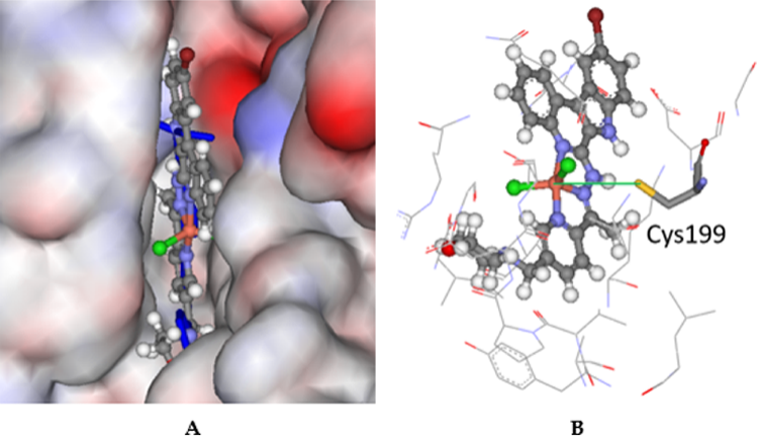
(A) Docked configuration of **8** (ball-and-stick format) in the GSK3β binding site (PDB ID: 3I4B) with the co-crystalized ligand Z48 colored blue, stick format (its hydrogen atoms are hidden for clarity). Extensive overlap is seen between the predicted and experimental configurations. The protein surface is rendered; blue depicts regions with a partial positive charge on the surface, red depicts regions with a partial negative charge, and gray shows neutral areas. (B) Predicted binding of complex **8**; amino acids within 5 Å are shown in line format. The potential chelating amino acid residue Cys199 is shown as sticks. The distance between the sulfur atom in the mercaptan moiety is shown as a green solid line at 4.5 Å.

The modeling for GSK3β showed that the ligands have two plausible binding modes, and the copper(II) complexes have a good fit in the pocket completely overlapping the co-crystallized Z48 ligand as shown in Figure 10A, indicating good binding. Cys199 is relatively close to the copper atom at 4.5 Å, but this amino acid residue is embedded deep within the protein, and it is therefore questionable how far the mercaptan moiety can reach (Figure 10B).

Docking studies of **8** in SGK-1 and PKA both show good overlap with the co-crystallized ligands. However, the binding can be characterized as weak. For both enzymes, the hydrophilic morpholine moiety is buried in the hydrophobic binding pocket and the bromine substituent pointing into the hydrophilic area. The results as well as the docking parameters are shown in Figures S17 and S18 and Tables S10 and S11 in the Supporting Information.

Thus, the molecular docking calculations are in agreement with the results of the kinase inhibition assays; good binding is seen for **HL^8^** to PIM-1 but not its copper(II) complex **8**. CaMK-1 and GSK3β both are predicted to have good binding to **8** in agreement with the kinase binding data.

### 2.12 Molecular Descriptors Indicate Drug-like Chemical Space for Lead Drug Candidates

The calculated molecular descriptors MW (molecular weight), log *P* (octanol–water partition coefficient), HD (hydrogen bond donors), HA (hydrogen bond acceptors), PSA (polar surface area), and RB (rotatable bonds) are given in Table S13 derived using the QikProp software.^95^ QikProp is not parameterized for copper(II) complexes. Therefore, Scigress^96^ was used instead with the available MW, log *P*, HD, and HA descriptors (Table S14). The values for the proligands’ descriptors lie mostly within drug-like chemical space with some exceptions. HD is in lead-like chemical space and for some proligands, MW and log *P* reach into the known drug space (KDS) (for the definition of lead-like, drug-like, and KDS regions see ref (97) and Table S12). The complexes obviously have higher MW and are all in the KDS, but the HD and HA remained intact (Table S14).

The known drug indexes (KDIs) for the proligands were calculated to gauge the balance of the molecular descriptors (MW, log *P*, HD, HA, PSA, and RB). This method is based on the analysis of drugs in clinical use, i.e., the statistical distribution of each descriptor is fitted to a Gaussian function and normalized to 1, resulting in a weighted index. Both the summation of the indexes (KDI_2A_) and multiplication (KDI_2B_) methods were used^98^ as shown for KDI_2A_ in eq 1 and for KDI_2B_ in eq 2; the numerical results are given in Table S13 in the Supporting Information.
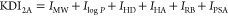
1
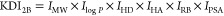
2

The KDI_2A_ values for the proligands range from 4.68 to 5.46 with a theoretical maximum of 6 and the average of 4.08 (±1.27) for known drugs. The KDI_2B_ range is from 0.14 to 0.55, with a theoretical maximum of 1 and with a KDS average of 0.18 (±0.20). This means that the molecular descriptors’ balance is reasonable with good biocompatibility. The low values can be explained by the high MW and log *P* values of some the compounds.

The proligands contain two imine groups linked by a N–N single bond. These groups are quite electron-rich, rendering them susceptible to an electrophilic attack. To test this, the ionization potential (one-electron oxidation) and electron affinity (one-electron reduction) were derived for **HL^1^** using DFT and compared to the statistical distribution of known drugs.^99^ The ionization potential is 6.6 eV, and 95% of drugs lie in the 6.0–9.0 eV range. The electron affinity is −1.3 eV with drugs in the −1.5 to 2.0 eV range.^99^ Thus, **HL^1^** is within the ranges of known drugs albeit with relatively low values. The bond dissociation energy (BDE) for the N–N single bond was also derived using DFT, resulting in 60.9 kcal/mol, which is substantially higher than the average for drugs (53.9 kcal/mol, *n* = 23).^100^

## 3 Conclusions

Two series of biologically active ligands, namely, latonduine derivatives (**HL^1^−HL^4^**), containing the indolo[2,3-*d*]benzazepine backbone **E**, and, indolo[2,3-*c*]quinoline (**C**) derivatives (**HL^5^−HL^8^**), were prepared via multistep syntheses. The new organic compounds **HL^1^–HL^4^** differ from the previously reported paullones, incorporating the indolo[3,2-*d*]benzazepine backbone **F**, by a flip of the indole moiety and a shift of the lactam unit in the seven-membered azepine ring, while **HL^5^–HL^8^** from the previously reported indolo[3,2-*c*]quinoline (**D**) derivatives by a flip of indole moiety solely. As previously described systems based on indolo[3,2-*d*]benzazepines (**F**) and indolo[3,2-*c*]quinolines (**D**), compounds **HL^1^–HL^8^** proved to be good chelators, enabling the preparation of a series of copper(II) complexes **1**–**8**, in which they act as tridentate neutral ligands. In addition, the organic compounds can act as tridentate monoanionic and monocationic chelating ligands with protonation of the morpholine nitrogen atom as supported by solution speciation studies and SC-XRD.

The determined p*K*_a_ values for **HL^1^** indicate that this morpholine-indolo[2,3-*d*]benzazepine hybrid is present in its neutral form at pH 7.4, and a similar behavior is suggested for the other ligands of the series. These are predicted to possess high lipophilic character, especially in the case of the bromo-substituted compounds (**HL^2^** and **HL^6^**). The thermodynamic solubility of copper(II) complexes was higher than that of the corresponding organic hybrids. The bromo-substituted derivatives showed lower aqueous solubility, unlike at the Schiff-base bond-methylated compound, where the effect was negligible. The determined stability constants of copper(II) complexes indicate high thermodynamic stability, and a low extent of dissociation is suggested at low micromolar concentrations at neutral pH. At this pH, the **[Cu(L)]^+^** species predominates, in which the non-coordinating hydrazonic nitrogen is most likely deprotonated.

Overall, the compounds studied in this work are highly antiproliferative in cancer cells and deserve further development as potential anticancer drugs. The indolo[2,3-*d*]benzazepine proligands **HL^1^**–**HL^4^** were found to be generally less cytotoxic than the analogous indolo[2,3-*c*]quinoline compounds **HL^5^**–**HL^8^** against human colon adenocarcinoma cell lines, and the presence of the bromo-substituent enhanced the selectivity for cancer cells. The copper(II) complexes are more cytotoxic than the corresponding metal-free hybrids. Some of the compounds (**HL^7^**, **7**, **HL^8^**, and **8**) displayed very good selectivity against cancer cells over the normal ones. Complex **4** was more cytotoxic against the resistant Colo320 cells in comparison to the sensitive cells, and it was more active than the **4_nm_**, which does not contain the morpholine moiety. Compounds **HL^4^**, **4**, **HL^8^**, and **8** could trigger apoptosis in the multidrug-resistant Colo320 cells more efficiently than cisplatin. Unlike **HL^4^** and **HL^8^**, complexes **4** and **8** were also able to replace the intercalative EtBr from ct-DNA. However, MTT assays with wild-type HCT116 cells with intact *p53* gene and its *p53*-deficient subline strongly suggest that DNA is not an effective target responsible for the inhibition of cell proliferation by lead drug candidates **HL^4^**, **4**, **HL^8^**, and **8**.

Enzyme inhibition assays against a panel of 50 related kinases revealed fully distinct inhibitory profiles for **HL^8^** and **8**, stressing again a special role of the metal on both the antiproliferative activity and the underlying mechanisms of cytotoxicity of this class of compounds at the molecular level. **HL^8^** proved to be selective and a potent inhibitor of PIM-1, while **8** strongly inhibited the activity of five other enzymes, namely, SGK-1, PKA, CaMK-1, MSK1, and GSK3β. The disclosed kinase inhibition was further supported by molecular docking calculations.

To further increase the affinity and selectivity of the lead drug candidates for particular enzymes, modification of lead structures by introduction of substituents and functional groups, which will allow for adjusting their electronic and steric properties, is needed. This work is going on in our laboratory and will be reported in due course.

## 4 Experimental Section

### 4.1 Starting Materials

2-Formyl-5-(morpholinomethyl)pyridine (**G** in Scheme 1) was synthesized in several steps as described elsewhere.^44^ The starting backbones **K** and **L** (see Scheme 3) were prepared as reported recently.^30,43^

### 4.2 Synthesis of Precursors and Metal-Free Ligands

The isolated yield and analytical data for **HL^1^**–**HL^8^** are summarized in Tables S15 and S16. The experimental CHN contents were within ±0.4% with those calculated, providing evidence for >95% purity.

#### 4.2.1 1-(5-(Morpholinomethyl)pyridin-2-yl)ethan-1-ol (Species **I** in Scheme 1)

A solution of **G** (2.03 g, 9.84 mmol) in dry THF (250 mL) was added dropwise to a solution of Grignard reagent (3 M in Et_2_O, 16.4 mL, 49.2 mmol) in dry THF (100 mL) under argon at room temperature. The resulting solution was stirred at room temperature for 2.5 h. The reaction was quenched with saturated aqueous solution of NH_4_Cl (160 mL) and water (200 mL). The product was extracted with DCM (2 × 100 mL). The combined organic phases were dried over MgSO_4_ and concentrated *in vacuo*. The raw product was purified on silica by using DCM/MeOH 94:6 as an eluent. After removal of the solvent, the product was isolated as a pale-yellow oil. Yield: 1.99 g, 91%. ^1^H NMR (500 MHz, DMSO-*d*_6_): δ 8.37 (d, *J* = 1.7 Hz, 1H, H^6^), 7.69 (dd, *J* = 8.0, 2.2 Hz, 1H, H^4^), 7.46 (d, *J* = 8.0 Hz, 1H, H^3^), 5.32 (d, *J* = 4.6 Hz, 1H, H^9^), 4.70 (dd, *J* = 6.5, 4.7 Hz, 1H, H^7^), 3.58–3.53 (m, 4H, H^13^), 3.46 (s, 2H, H^10^), 2.33 (s, 4H, H^12^), 1.34 (d, *J* = 6.5 Hz, 3H, H^8^). ^13^C NMR (151 MHz, DMSO-*d*_6_): δ 164.54 (Cq, C^2^), 148.69 (CH, C^6^), 137.23 (CH, C^4^), 131.23 (Cq, C^5^), 118.86 (CH, C^3^), 69.25 (CH, C^7^), 66.14 (CH_2_, C^13^), 59.36 (CH_2_, C^10^), 53.03 (CH_2_, C^12^), 24.25 (CH_3_, C^8^). For the atom numbering scheme, see Chart S1. ESI-MS (acetonitrile/methanol + 1% water), positive: *m*/*z* 223.18 [M + H]^+^ (calcd *m*/*z* for [C_12_H_18_N_2_O_2_ + H]^+^: 223.14).

#### 4.2.2 2-Acetyl-5-(morpholinomethyl)pyridine (**J** in Scheme 1)

Oxalylchloride (517 μL, 5.93 mmol) was diluted with dry DCM (9 mL) and cooled to −80 °C. Dry DMSO (890 μL, 12.5 mmol) was added dropwise. Five minutes later, to this solution, a solution of **I** (1.29 g, 5.80 mmol) in DCM (10 mL) was added. The reaction mixture was stirred at −80 °C for 15 min. Then, triethylamine (3.64 mL) was added dropwise (the solution turned turbid) and stirring was continued overnight while the reaction mixture slowly reached room temperature. The reaction was quenched with water (20 mL). The organic phase was separated, and the aqueous phase was extracted with DCM (3 × 20 mL). The combined organic phases were dried over MgSO_4_. The solvent was removed under reduced pressure, and the crude product was purified on silica by using DCM/MeOH 98:2 as an eluent to give the product as a pale-yellow solid. Yield: 878 mg, 69%. Anal. calcd for C_12_H_16_N_2_O_2_ (*M*_r_ = 220.27): C, 65.43; H, 7.32, N, 12.72. Found: C, 65.22; H, 7.01; N, 12.54. ^1^H NMR (500 MHz, DMSO-*d*_6_): δ 8.65 (s, 1H, H^6^), 7.93 (dd, *J* = 4.1, 1.4 Hz, 2H, H^3^, H^4^), 3.60 (s, 2H, H^10^), 3.59–3.55 (m, 4H, H^13^), 2.63 (s, 3H, H^8^), 2.37 (d, *J* = 3.8 Hz, 4H, H^12^). ^13^C NMR (151 MHz, DMSO-*d*_6_): δ 199.20 (Cq, C^7^), 152.07 (Cq, C^2^), 149.54 (CH, C^6^), 137.83 (Cq, C^5^), 137.67 (CH, C^4^), 120.90 (CH, C^3^), 66.13 (CH_2_, C^13^), 59.16 (CH_2_, C^10^), 53.07 (CH_2_, C^12^), 25.68 (CH_3_, C^8^). For the atom numbering scheme, see Chart S1. ESI-MS (acetonitrile/methanol + 1% water), positive: *m*/*z* 221.17 [M + H]^+^ (calcd *m*/*z* for [C_12_H_16_N_2_O_2_ + H]^+^: 221.13).

### 4.3 Synthesis of Precursors **Ib–IVb**, **Va**, and **Vb** and **N** and **M**

#### 4.3.1 5-Bromo-1-(ethoxymethyl)-*N*-(2-iodophenyl)-1*H*-indole-2-carboxamide (**I**)

Under an argon atmosphere, 2-iodoaniline (3.54 g, 16.16 mmol) was dissolved in dry DCM (10 mL). The solution was cooled to −20 °C, and AlMe_3_ (10.1 mL, 20.2 mmol) was added dropwise. The mixture was stirred at −20 °C for 45 min before slowly reaching 0 °C. Then, ethyl 5-bromo-1-ethoxymethyl-1*H*-indol-2-carboxylate^43^ (1.32 g, 4.04 mmol) in dry DCM (8 mL) was added dropwise. The mixture was stirred at room temperature for 18 h before being cooled to 0 °C again. Then, 1 M HCl (90 mL, 90 mmol) was added slowly. The solution was stirred for 10 min at room temperature until phase separation was clearly seen. The aqueous phase was extracted with DCM (3 × 100 mL). The combined organic phases were dried over MgSO_4_ and concentrated *in vacuo*. The crude product was refluxed in methanol (30 mL) and cooled to 4 °C. The product was collected by filtration as a white, cloudy solid. Yield: 1.74 g, 84%. ^1^H NMR (500 MHz, DMSO-*d*_6_): δ 10.25 (s, 1H), 7.98 (d, *J* = 1.8 Hz, 1H), 7.95 (d, *J* = 7.8 Hz, 1H), 7.66 (d, *J* = 8.9 Hz, 1H), 7.50–7.44 (m, 3H), 7.37 (s, 1H), 7.11–7.06 (m, 1H), 5.98 (s, 2H), 3.41 (q, *J* = 7.0 Hz, 2H), 1.01 (t, *J* = 7.0 Hz, 3H). ESI-MS (acetonitrile/methanol + 1% water), negative: 498.81 [M – H]^−^ (calcd *m*/*z* for [C_18_H_16_BrIN_2_O_2_ – H]^−^: 498.93).

#### 4.3.2 *tert*-Butyl-(5-bromo-1-(ethoxymethyl)-1*H*-indole-2-carbonyl)(2-iodophenyl)carbamate (**IIb**)

To **Ib** (1.65 g, 3.3 mmol) in dry acetonitrile (65 mL) diboc (1.15 g, 5.28 mmol) and a catalytic amount of 4-dimethylaminopyridine (DMAP) were added under an argon atmosphere. The suspension was stirred at room temperature for 72 h. The solvent was removed at reduced pressure. The resulting crude solid was extracted with a mixture of DCM and water 1:1 (60 mL). The aqueous layer was further extracted with DCM (2 × 30 mL), and the combined organic phases were dried over MgSO_4_. The crude product was purified on silica by using hexane:ethylacetate 95:5 as an eluent. The product was obtained as a white solid. Yield: 1.94 g, 99%. ^1^H NMR (500 MHz, DMSO-*d*_6_): δ 7.99 (dt, *J* = 4.0, 2.0 Hz, 1H), 7.97 (d, *J* = 1.9 Hz, 1H), 7.70 (d, *J* = 8.9 Hz, 1H), 7.48 (dddd, *J* = 19.4, 9.4, 7.8, 1.5 Hz, 3H), 7.21–7.15 (m, 2H), 5.81–5.73 (m, 2H), 3.54–3.46 (m, 1H), 3.46–3.37 (m, 1H), 1.24–1.18 (m, 9H), 1.07 (t, *J* = 7.0 Hz, 3H). ESI-MS (acetonitrile/methanol + 1% water), positive: *m*/*z* 623.05 [M + Na]^+^ (calcd *m*/*z* for [C_23_H_24_BrIN_2_O_4_ + Na]^+^: 622.98).

#### 4.3.3 *tert*-Butyl-10-bromo-7-(ethoxymethyl)-6-oxo-6,7-dihydroindolo[2,3-c]-quinoline-5-carboxylate (**IIIb**)

To **IIb** (1.94 g, 3.2 mmol) in dry DMF (75 mL) Pd(OAc)_2_ (182 mg, 0.81 mmol), PPh_3_ (425 mg, 1.62 mmol), and Ag_2_CO_3_ (1.78 g, 6.47 mmol) were added, and the suspension was stirred at 100 °C for 2 h under an argon atmosphere. The reaction mixture was cooled down to room temperature, and the solvent was removed by a rotary evaporator. The residue was taken up in DCM (20 mL) and filtered over celite. The solvent was evaporated, and the crude product was purified on silica by using hexane:ethylacetate 85:15 as an eluent. The solvent was evaporated, and the brownish solid was recrystallized from hexane (13 mL) to give the product as a white solid. Yield: 301 mg, 19%. ^1^H NMR (500 MHz, DMSO-*d*_6_): δ 8.81 (s, 1H), 8.67 (d, *J* = 8.0 Hz, 1H), 7.89 (d, *J* = 8.9 Hz, 1H), 7.76 (dd, *J* = 8.9, 1.5 Hz, 1H), 7.61 (t, *J* = 7.8 Hz, 1H), 7.54–7.45 (m, 1H), 7.24 (d, *J* = 8.3 Hz, 1H), 6.25 (s, 2H), 3.49 (p, *J* = 6.9 Hz, 2H), 1.67 (s, 9H), 1.04 (q, *J* = 6.8 Hz, 3H). ESI-MS (acetonitrile/methanol + 1% water), positive: *m*/*z* 965.16 [2 M + Na]^+^ (calcd *m*/*z* for [(C_23_H_23_BrN_2_O_4_)_2_ + Na]^+^: 965.16).

#### 4.3.4 10-Bromo-5,7-dihydro-6*H*-indolo-[2,3-*c*]-quinolin-6-one (**IVb**)

To **IIIb** (301 mg, 0.64 mmol) in ethanol (16 mL) 12 M HCl (4 mL) was added, and the solution was stirred at 100 °C overnight. The precipitate was filtered off, and the product was washed with ethanol (2 mL) to give a light-yellow solid. Yield: 159 mg, 79%. ^1^H NMR (500 MHz, DMSO-*d*_6_): δ 12.59 (s, 1H), 11.95 (s, 1H), 8.68 (s, 1H), 8.46 (d, *J* = 7.6 Hz, 1H), 7.60 (t, *J* = 4.2 Hz, 2H), 7.51 (dd, *J* = 8.1, 0.9 Hz, 1H), 7.45–7.40 (m, 1H), 7.37–7.32 (m, 1H). ESI-MS (acetonitrile/methanol +1% water), positive: *m*/*z* 312.99 [M + H]^+^, 648.94 [2 M + Na]^+^ (calcd *m*/*z* for [C_15_H_9_BrN_2_O + H]^+^: 313.00 and calcd *m*/*z* for [(C_15_H_9_BrN_2_O)_2_ + Na]^+^: 648.97).

#### 4.3.5 6-Chloro-7*H*-indolo[2,3-*c*]quinoline (**Va**)

5,7-Dihydro-6*H*-indolo[2,3-*c*]quinolin-6-one (**IVa**)^30^ (9.36 g, 40.0 mmol) and phosphorus oxychloride (140 mL) were mixed in a 250 mL nitrogen flask under an argon atmosphere. The mixture was refluxed overnight. The next day, it was cooled to room temperature and poured on ice water (800 mL), which was cooled additionally by an ice bath (strongly exothermic reaction). Solid sodium hydroxide was added in portions under cooling (strongly exothermic reaction) until a pH of about 12 was reached. Then, the reaction mixture was extracted with dichloromethane (3 × 500 mL). The organic phase was washed with water (3 × 200 mL) and brine (1 × 200 mL) and dried over magnesium sulfate. The solvent was removed under reduced pressure. Yield: 9.47 g, 94%. ^1^H NMR (500 MHz, DMSO-*d*_6_): δ 12.48 (s, 1H, N*H*), 8.84 (d, *J* = 8.1 Hz, 1H, *H*_(Ar)_), 8.71 (d, *J* = 8.2 Hz, 1H, *H*_(Ar)_), 8.12 (dd, *J* = 8.3, 0.9 Hz, 1H, *H*_(Ar)_), 7.80 (ddd, *J* = 8.2, 4.0, 1.3 Hz, 2H, *H*_(Ar)_), 7.74–7.69 (m, 1H, *H*_(Ar)_), 7.67–7.63 (m, 1H, *H*_(Ar)_), 7.47–7.41 (m, 1H, *H*_(Ar)_). ESI-MS (acetonitrile/methanol + 1% water), positive: *m*/*z* 253.04 [M + H]^+^.

#### 4.3.6 10-Bromo-6-chloro-7*H*-indolo[2,3-*c*]quinoline (**Vb**)

Species **IVb** (159 mg, 0.5 mmol) was suspended in POCl_3_ (5 mL), and the mixture was stirred at 120 °C overnight. The yellow suspension was cooled to room temperature and poured onto ice water (20 mL). The pH was set to ∼11 with NaOH pellets. The white suspension was extracted with DCM (3 × 30 mL). The combined organic phases were washed with brine (80 mL) and dried over MgSO_4_. The product was obtained after evaporation of the solvent as a light-beige solid. Yield: 153 mg, 94%. ^1^H NMR (500 MHz, DMSO-*d*_6_): δ 12.67 (s, 1H, N*H*), 8.90 (d, *J* = 1.7 Hz, 1H, *H*_(Ar)_), 8.85 (dd, *J* = 8.2, 1.4 Hz, 1H, *H*_(Ar)_), 8.11 (dd, *J* = 8.3, 1.3 Hz, 1H, *H*_(Ar)_), 7.83–7.69 (m, 4H, *H*_(Ar)_). ESI-MS (acetonitrile/methanol +1% water), positive: *m*/*z* 333.06 [M + H]^+^ (calcd *m*/*z* for [C_15_H_8_BrClN_2_ + H]^+^: 332.96).

#### 4.3.7 6-Hydrazineyl-7*H*-indolo[2,3-*c*]quinoline (**M**)

6-Chloro-7*H*-indolo[2,3-*c*]quinoline (**Va**) (9.46 g, 37.4 mmol) was mixed with hydrazine hydrate (140 mL) in a 500 mL nitrogen flask under an argon atmosphere. The reaction mixture was refluxed overnight. The next day, it was cooled to room temperature and stored at 4 °C overnight. The next day, the slightly orange product was filtered off, washed with water, and dried *in vacuo*. Yield: 9.10 g, 98%. ^1^H NMR (500 MHz, DMSO-*d*_6_): δ 11.54 (s, 1H, N*H*), 8.58–8.47 (m, 2H, *H*_(Ar)_), 8.11 (s, 1H, N*H*), 7.77 (dd, *J* = 13.2, 8.2 Hz, 2H, *H*_(Ar)_), 7.48 (dt, *J* = 24.8, 7.4 Hz, 2H, *H*_(Ar)_), 7.36 (dt, *J* = 24.1, 7.4 Hz, 2H, *H*_(Ar)_), 4.66 (s, 2H, N*H*).

#### 4.3.8 10-Bromo-6-hydrazineyl-7*H*-indolo[2,3-*c*]quinoline (**N**)

A mixture of **Vb** (0.96 g, 2.92 mmol) and hydrazine hydrate (37.0 mL) was refluxed at 131 °C overnight. Next day, the solution was cooled to room temperature and allowed to stand at 4 °C overnight. The product was filtered off, washed with water, and dried *in vacuo*. Yield: 0.91 g, 95%. ^1^H NMR (500 MHz, DMSO-*d*_6_): δ 11.61 (s, 1H), 8.70 (s, 1H), 8.51 (d, *J* = 6.9 Hz, 1H), 8.17 (s, 1H), 7.80–7.73 (m, 2H), 7.62 (d, *J* = 8.0 Hz, 1H), 7.48–7.36 (m, 2H), 4.68 (s, 2H). ESI-MS (acetonitrile/methanol + 1% water), positive: *m*/*z* 327.13 [M + H]^+^ (calcd *m*/*z* for [C_15_H_11_BrN_4_ + H]^+^: 327.02).

### 4.4 Synthesis of Proligands (**HL^1^−HL^8^**)

#### 4.4.1 **HL^1^**·H_2_O

Species **G** (209 mg, 1.01 mmol) was added to a solution of species **K** (241 mg, 0.92 mmol) in anoxic ethanol (3 mL), and the mixture was stirred at 90 °C overnight. The product was precipitated with water. Ethanol (10 mL) was added to the suspension to dissolve the precipitate. The solution was slowly concentrated by 1/2 under reduced pressure. The product was isolated by filtration, washed with cold ethanol (1 mL), and dried *in vacuo* overnight to give a yellow powder. Yield: 398 mg. ^1^H NMR (600 MHz, DMSO-*d*_6_): δ 11.92 (s, 1H, H^8^), 8.50 (d, *J* = 1.4 Hz, 1H, H^18^), 8.36 (s, 1H, H^15^), 8.32 (d, J = 8.1 Hz, 1H, H^21^), 8.27 (t, *J* = 5.3 Hz, 1H, H^6^), 7.99 (d, J = 8.1 Hz, 1H, H^1^), 7.96 (d, J = 7.1 Hz, 1H, H^12^), 7.78 (dd, *J* = 8.0, 1.9 Hz, 1H, H^20^), 7.59 (d, *J* = 8.2 Hz, 1H, H^9^), 77.51–7.48 (m 1H, H^2^), 7.47–7.44 (m, 1H, H^4^), 7.37–7.29 (m, 2H, H^3^, H^10^), 7.22–7.16 (m, 1H, H^11^), 4.23 (d, *J* = 95.0 Hz, 2H, 2H^5^), 3.62–3.55 (m, 4H, 2H^25^, 2H^27^), 3.53 (s, 2H, 2H^22^), 2.37 (d, *J* = 4.7 Hz, 4H, 2H^24^, 2H^28^). ^13^C NMR (151 MHz, DMSO-*d*_6_): δ 155.51 (Cq, C^7^), 153.51 (Cq, C^16^), 152.01 (CH, C^15^), 149.72 (CH, C^18^), 137.80 (Cq, C^8a^), 136.89 (CH, C^20^), 136.76 (Cq, C^4a^), 133.62 (Cq, C^12c^), 133.53 (Cq, C^19^), 129.03 (Cq, C^7a^), 128.09 (CH, C^4^), 128.05 (CH, C^2^), 127.41 CH, C^12^), 126.32 (CH, C^10^), 124.94 (Cq, C^12a^), 124.09 (CH, C^3^), 120.45 (CH, C^11^), 120.34 (CH, C^21^), 120.20 (CH, C^1^), 117.07 (Cq, C^12b^), 112.68 (CH, C^9^), 66.16 (2CH_2,_ C^25^, C^27^), 59.41 (CH_2_, C^22^), 53.09 (2CH_2_, C^24^, C^28^), 46.00 (CH_2_, C^5^).

#### 4.4.2 **HL^2^**·0.6H_2_O

A mixture of **L** (300 mg, 0.88 mmol) and **G** (181 mg, 0.88 mmol) was dissolved in anoxic ethanol (5 mL), and the solution was stirred at 85 °C overnight. The solvent was evaporated, and the resulting precipitate was taken up in water and diluted with ethanol (10 mL). The clear yellow solution was slowly concentrated and allowed to stand at 4 °C overnight. The bright-yellow precipitate was filtered off, washed with ethanol (1 mL), and dried *in vacuo* overnight. Yield: 150 mg. ^1^H NMR (600 MHz, DMSO-*d*_6_): δ 12.13 (s, 1H, H^8^), 8.50 (s, 1H, H^18^), 8.37–8.25 (m, 3H, H^6^, H^21^, H^15^), 8.10 (s, 1H, H^12^), 7.91 (d, *J* = 7.6 Hz, 1H, H^1^), 7.78 (d, *J* = 8.0 Hz, 1H, H^20^), 7.57–7.49 (m, 2H, H^9^, H^2^), 7.49–7.43 (m, 2H, H^4^, H^10^), 7.36 (t, *J* = 7.4 Hz, 1H, H^3^), 4.40–4.02 (m, 2H, H^5^), 3.58 (s, 4H, H^25^), 3.52 (s, 2H, H^22^), 2.37 (s, 4H, H^24^). ^13^C NMR (176 MHz, DMSO-*d*_6_): δ 155.12 (Cq, C^7^), 153.37 (Cq, C^16^), 152.37 (CH, C^15^), 149.70 (CH, C^18^), 137.86 (Cq, C^4a^), 136.86 (CH, C^20^), 135.36 (Cq, C^8a^), 133.61 (Cq, C^19^), 132.94 (Cq, C^12c^), 130.29 (Cq, C^7a^), 128.24 (CH, C^4^), 128.14 (CH, C^2^), 127.32 (CH, C^1^), 126.67 (CH, C^10^), 126.59 (CH, C^3^), 126.52 (Cq, C^12a^), 122.20 (CH, C^12^), 120.37 (CH, C^21^), 116.42 (Cq, C^12b^), 114.66 (CH, C^9^), 112.98 (Cq, C^11^), 66.13 (2CH_2_, C^25^), 59.37 (CH_2_, C^22^), 53.06 (2CH_2_, C^24^), 45.88 (CH_2_, C^5^).

#### 4.4.3 **HL^3^**·0.3C_2_H_5_OH

To a solution of **K** (320 mg, 1.22 mmol) in anoxic ethanol (5 mL) ketone **J** (295.6 mg, 1.34 mmol) was added, and the resulting solution was stirred at 85 °C overnight. The yellow suspension was cooled to room temperature and allowed to stand at 4 °C overnight. The yellow solid was filtered off, washed with diethyl ether (2 mL), and dried *in vacuo* overnight to give a yellow powder. Yield: 373 mg. ^1^H NMR (600 MHz, DMSO-*d*_6_): δ 11.76 (s, 1H, N^8^), 8.49 (d, *J* = 1.5 Hz, 1H, C^18^), 8.45 (d, *J* = 8.1 Hz, 1H, C^21^), 7.99 (d, *J* = 8.1 Hz, 1H, C^12^), 7.95 (d, *J* = 7.1 Hz, 1H, C^1^), 7.93 (t, *J* = 5.3 Hz, 1H, N^6^), 7.73 (dd, *J* = 8.2, 2.2 Hz, 1H, C^20^), 7.61 (d, *J* = 8.2 Hz, 1H, C^9^), 7.49 (td, *J* = 7.6, 1.3 Hz, 1H, C^2^), 7.45–7.42 (m, 1H, C^4^), 7.35–7.30 (m, 2H, C^3^, C^10^), 7.22–7.17 (m, 1H, C^11^), 4.46–3.99 (m, 2H, C^5^), 3.61–3.56 (m, 4H, C^25^), 3.53 (s, 2H, C^22^), 2.49 (s, 3H, C^27^), 2.41–2.35 (m, 4H, C^24^). ^13^C NMR (151 MHz, DMSO-*d*_6_): δ 158.13 (Cq, C^15^), 155.51 (Cq, C^16^), 153,27, (Cq, C^7^), 148.87 (CH, C^18^), 137.96 (Cq, C^4a^), 136.66 (Cq, C^8a^), 136.53 (CH, C^20^), 133.74 (Cq, C^12c^), 132.91 (Cq, C^19^), 129.76 (Cq, C^7a^), 128.07 (CH, C^4^), 127.97 (CH, C^2^), 127.33 (CH, C^1^), 126.17 (CH, C^3^), 125.05 (Cq, C^12a^), 123.91 (CH, C^10^), 120.42 (CH, C^21^), 120.36 (CH, C^11^), 120.12 (CH, C^12^), 116.47 (Cq, C^12b^), 112.61 (CH, C^9^), 66.18 (CH_2_, C^25^), 59.37 (CH_2_, C^22^), 53.08 (CH_2_, C^24^), 45.97 (CH_2_, C^5^), 13.14 (CH3, C^27^).

#### 4.4.4 HL^4^

To a solution of **L** (175.8 mg, 0.51 mmol) in anoxic ethanol (5 mL) ketone **J** (124.8 mg, 0.57 mmol) was added, and the resulting mixture was stirred at 85 °C overnight. The yellow suspension was cooled to room temperature and allowed to stand at 4 °C overnight. The yellow solid was filtered off, washed with diethyl ether (2 mL), and dried *in vacuo* overnight to give a yellow powder. Yield: 158 mg. ^1^H NMR (600 MHz, DMSO-*d*_6_): δ 11.98 (s, 1H, N^8^), 8.49 (s, 1H, C^18^), 8.45 (d, *J* = 8.2 Hz, 1H, C^21^), 8.09 (s, 1H, C^12^), 7.96 (t, *J* = 5.2 Hz, 1H, N^6^), 7.91 (d, *J* = 7.7 Hz, 1H, C^1^), 7.73 (dd, *J* = 8.2, 1.8 Hz, 1H, C^20^), 7.57 (d, *J* = 8.7 Hz, 1H, C^9^), 7.51 (t, *J* = 7.5 Hz, 1H, C^2^), 7.44 (dd, *J* = 11.9, 4.6 Hz, 2H, C^4^, C^10^), 7.34 (t, *J* = 7.4 Hz, 1H, C^3^), 4.41–4.03 (m, 2H, C^5^), 3.61–3.56 (m, 4H, C^25^), 3.53 (s, 2H, C^22^), 2.48 (s, 3H, C^27^), 2.37 (s, 4H, C^24^). ^13^C NMR (151 MHz, DMSO-*d*_6_): δ 158.51 (Cq, C^15^) 155.28 (Cq, C^16^), 152.86 (Cq, C^7^), 148.86 (CH, C^18^), 138.03 (Cq, C^4a^), 136.56 (CH, C^20^), 135.29 (Cq, C^8a^), 133.09 (Cq, C^19^), 133.07 (Cq, C^12c^), 131.02 (Cq, C^7a^), 128.18 (CH, C^4^), 128.14 (CH, C^2^), 127.26 (CH, C^1^), 126.67 (Cq, C^11^), 126.50 (CH, C^10^), 126.47 (CH, C^3^), 122.12 (CH, C^12^), 120.45 (CH, C^21^), 115.85 (Cq, C^12b^), 114.62 (CH, C^9^), 112.90 (Cq, C^12a^), 66.15 (CH_2_, C^25^), 59.33 (CH_2_, C^22^), 53.03 (CH_2_, C^24^), 45.86 (CH_2_, C^5^), 13.14 (CH_3_, C^27^).

#### 4.4.5 **HL^5^**·0.5H_2_O

A suspension of **G** (243 mg, 1.17 mmol) and **M** (290 mg, 1.17 mmol) in anoxic ethanol (6 mL) was stirred at 85 °C overnight. Next day, the reaction mixture was cooled to room temperature and allowed to stand at 4 °C for 2 h. The yellow precipitate was filtered off, washed with cold ethanol, and dried *in vacuo*. Yield: 466 mg. ^1^H NMR (600 MHz, DMSO-*d*_6_): δ 12.18 (s, 1H, H^7^), 11.05 (s, 1H, H^5^), 8.57 (d, *J* = 8.1 Hz, 1H, H^20^), 8.55–8.51 (m, 2H, H^17^, H^14^), 8.41 (d, *J* = 8.1 Hz, 1H, H^11^), 8.37 (d, *J* = 7.7 Hz, 1H, H^1^), 7.90 (d, *J* = 7.9 Hz, 1H, H^4^), 7.85–7.82 (m, 1H, H^19^), 7.66 (d, *J* = 8.2 Hz, 1H, H^8^), 7.43 (dd, *J* = 14.5, 6.6 Hz, 1H, H^9^), 7.39 (dd, *J* = 11.2, 4.1 Hz, 1H, H^3^), 7.32–7.27 (m, 1H, H^3^), 3.61–3.58 (m, 4H, H^24^), 3.56 (s, 2H, H^21^), 2.40 (s, 4H, H^23^). ^13^C NMR (151 MHz, DMSO-*d*_6_): δ 153.58 (Cq, C^15^), 151.77 (CH, C^14^), 149.73 (CH, C^17^), 146.57 (Cq, C^6^), 139.29 (Cq, C^7a^), 136.84 (CH, C^19^), 134.74 (Cq, C^4a^), 133.34 (Cq, C^18^), 126.63 (Cq, C^6a^), 125.95 (CH, C^3^), 125.29 (CH, C^9^), 122.89 (CH, C^1^), 122.33 (Cq, C^11a^), 122.22 (CH, C^10^), 121.67 (CH, C^11^), 120.77 (CH, C^20^), 120.67 (CH, C^2^), 118.84 (Cq, C^11c^), 116.50 (CH, C^4^), 116.10 (Cq, C^11b^), 112.93 (CH, C^8^), 66.18 (CH_2_, C^24^), 59.43 (CH_2_, C^21^), 53.09 (CH_2_, C^23^).

#### 4.4.6 **HL^6^**·H_2_O

A suspension of **G** (114 mg, 0.55 mmol) and **N** (180 mg, 0.55 mmol) in anoxic ethanol (6 mL) was stirred at 85 °C overnight. Next day, the reaction mixture was cooled to room temperature and allowed to stand at 4 °C for 2 h. The yellow-orange precipitate was filtered off, washed with cold ethanol, and dried *in vacuo*. Yield: 221 mg. ^1^H NMR (600 MHz, DMSO-*d*_6_) (species with an exocyclic double bond): δ 12.38 (s, 1H, H^7^), 11.07 (s, 1H, H^5^), 8.59 (d, *J* = 1.6 Hz, 1H, H^11^), 8.56 (d, *J* = 8.2 Hz, 1H, H^20^), 8.54 (d, *J* = 1.4 Hz, 1H, H^17^), 8.52 (s, 1H, H^14^), 8.36 (d, *J* = 7.5 Hz, 1H, H^1^), 7.90–7.86 (m, 1H, H^4^), 7.86–7.79 (m, 1H, H^19^), 7.61 (d, *J* = 8.7 Hz, 1H, H^8^), 7.59–7.55 (m, 1H, H^9^), 7.43–7.39 (m, 1H, H^3^), 7.31–7.27 (m, 1H, H^2^), 3.63–3.57 (m, 4H, H^24^), 3.56 (s, 2H, H^21^), 2.45–2.35 (m, 4H, H^23^). ^13^C NMR (151 MHz, DMSO-*d*_6_): δ 153.42 (Cq, C^15^), 152.19 (CH, C^14^), 149.73 (CH, C^17^), 146.31 (Cq, C^6^), 137.90 (Cq, C^7a^), 136.85 (CH, C^19^), 134.78 (Cq, C^4a^), 133.45 (Cq, C^18^), 127.90 (CH, C^9^), 127.65 (Cq, C^6a^), 126.25 (CH, C^3^), 123.85 (Cq, C^11a^), 123.69 (CH, C^11^), 123.12 (CH, C^1^), 122.34 (CH, C^2^), 120.84 (CH, C^20^), 118.24 (Cq, C^11c^), 116.47 (CH, C^4^), 115.41 (Cq, C^11b^), 114.81 (CH, C^8^), 113.13 (Cq, C^10^), 66.15 (2CH_2_, C^24^), 59.40 (CH_2_, C^21^), 53.07 (2CH_2_, C^23^). ^1^H NMR (600 MHz, DMSO-*d*_6_) (species with an endocyclic double bond): δ 14.54 (s, 1H, H^12^), 11.97 (s, 1H, H^7^), 8.82–8.78 (m, 2H, H^17^, H^11^), 8.67 (dd, *J* = 8.0, 1.0 Hz, 1H, H^1^), 8.01–7.98 (m, 1H, H^19^), 7.94–7.91 (m, 1H, H^8^), 7.90–7.86 (m, 1H, H^4^), 7.86–7.79 (m, 1H, H^19^), 7.71–7.68 (m, 1H, H^9^), 7.59–7.55 (m, 2H, H^3^, H^14^), 7.55–7.51 (m, 1H, H^2^), 3.63–3.57 (m, 6H, H^21^, H^24^), 2.45–2.35 (m, 4H, H^23^). ^13^C NMR (151 MHz, DMSO-*d*_6_): δ 151.64 (Cq, C^15^), 148.43 (CH, C^17^), 144.02 (Cq, C^6^), 141.44 (Cq, C^4a^), 138.29 (CH, C^19^), 137.90 (Cq, C^7a^), 133.60 (Cq, C^18^), 131.43 (CH, C^14^), 128.82 (CH, C^9^), 126.95 (CH, C^4^), 126.04 (CH, C^3^), 125.29 (CH, C^20^), 124.35 (CH, C^2^), 124.16 (CH, C^11^), 122.90 (CH, C^1^), 122.77 (Cq, C^6a^), 122.66 (Cq, C^11a^), 121.55 (Cq, C^11c^), 120.93 (Cq, C^11b^), 115.63 (CH, C^8^), 112.76 (Cq, C^10^), 66.17 (2CH_2_, C^24^), 59.19 (CH_2_, C^21^), 53.04 (2CH_2_, H^23^).

#### 4.4.7 **HL^7^**·0.5H_2_O

A suspension of **J** (175 mg, 0.79 mmol) and **M** (200 mg, 0.79 mmol) in anoxic ethanol (5 mL) was stirred at 85 °C overnight. Next day, the reaction mixture was cooled to room temperature and allowed to stand at 4 °C for 4 h. The yellow-orange precipitate was filtered off, washed with cold ethanol, and dried *in vacuo*. Yield: 333 mg. ^1^H NMR (600 MHz, DMSO-*d*_6_): δ 11.95 (s, 1H, H^7^), 10.74 (s, 1H, H^5^), 8.68 (d, *J* = 8.1 Hz, 1H, H^20^), 8.53 (s, 1H, H^17^), 8.40 (d, *J* = 8.1 Hz, 1H, H^11^), 8.34 (d, *J* = 7.7 Hz, 1H, H^1^), 7.86 (d, *J* = 8.1 Hz, 1H, H^4^), 7.79 (dd, *J* = 8.2, 1.7 Hz, 1H, H^19^), 7.70 (d, *J* = 8.2 Hz, 1H, H^8^), 7.44 (t, *J* = 7.6 Hz, 1H, H^9^), 7.36 (dd, *J* = 11.2, 4.1 Hz, 1H, H^3^), 7.32 (m, 2H, H^2^, H^10^), 3.60 (s, 4H, H^24^), 3.56 (s, 2H, H^21^), 2.68 (s, 3H, H^26^), 2.40 (s, 4H, H^23^). ^13^C NMR (151 MHz, DMSO-*d*_6_): δ 158.19 (Cq, C^14^), 155.53 Cq, C^15^), 148.89 (CH, C^17^), 144.68 (Cq, C^6^), 139.12 (Cq, C^7a^), 136.50 (CH, C^19^), 134.89 (Cq, C^4a^), 132.84 (Cq, C^18^), 127.39 (Cq, C^6a^), 125.78 (CH, C^3^), 125.05 (CH, C^9^), 122.78 (CH, C^1^), 122.51 (Cq, C^11a^), 121.93 (CH, C^2^), 121.56 (CH, C^11^), 120.88 (CH, C^20^), 120.58 (CH, C^10^), 118.79 (Cq, C^11c^), 116.46 (CH, C^4^), 115.40 (Cq, C^11b^), 112.86 (CH, C^8^), 66.17 (2 CH_2_, H^24^), 59.38 (CH_2_, H^21^), 53.06 (2 CH_2_, H^23^), 13.23 (CH_3_, C^26^).

#### 4.4.8 **HL^8^**·0.5H_2_O

A suspension of **J** (121 mg, 0.55 mmol) and **N** (180 mg, 0.79 mmol) in anoxic ethanol (6 mL) was stirred at 85 °C overnight. Next day, the reaction mixture was cooled to room temperature and allowed to stand at 4 °C for 2 h. The yellow-orange product was precipitated by addition of diethyl ether and filtered off, washed with cold ethanol, and dried *in vacuo*. Yield: 168 mg. ^1^H NMR (600 MHz, DMSO-*d*_6_): δ 12.14 (s, 1H, H^7^), 10.75 (s, 1H, H^5^), 8.67 (d, *J* = 8.1 Hz, 1H, H^20^), 8.57 (d, *J* = 1.7 Hz, 1H, H^11^), 8.53 (d, *J* = 1.6 Hz, 1H, H^17^), 8.33 (d, *J* = 7.6 Hz, 1H, H^1^), 7.85 (dd, *J* = 8.2, 0.8 Hz, 1H, H^4^), 7.79 (dd, *J* = 8.2, 2.1 Hz, 1H, H^19^), 7.65 (d, *J* = 8.7 Hz, 1H, H^8^), 7.56 (dd, *J* = 8.7, 1.8 Hz, 1H, H^9^), 7.39–7.34 (m, 1H, H^3^), 7.29–7.23 (m, 1H, H^2^), 3.61–3.59 (m, 4H, H^24^), 3.56 (s, 2H, H^21^), 2.67 (s, 3H, H^25^), 2.40 (s, 4H, H^23^). ^13^C NMR (151 MHz, DMSO-*d*_6_) *δ* 158.65 (Cq, C^14^), 155.41 (Cq, C^15^), 148.90 (CH, C^17^), 144.38 (Cq, C^6^), 137.77 (Cq, C^7a^), 136.52 (CH, C^19^), 134.96 (Cq, C^4a^), 132.96 (Cq, C^18^), 128.44 (Cq, C^4a^), 127.66 (CH, C^9^), 126.11 (CH, C^3^), 124.07 (Cq, C^11a^), 123.59 (CH, C^11^), 123.01 (CH, C^1^), 122.06 (CH, C^2^), 120.94 (CH, C^20^), 118.19 (Cq, C^11c^), 116.44 (CH, C^4^), 114.77 (CH, C^8^), 114.74 (Cq, C^11b^), 113.07 (Cq, C^10^), 66.17 (2CH_2_, C^24^), 59.37 (CH_2_, C^21^), 53.06 (2CH_2_, C^23^), 13.26 (CH_3_, C^25^).

### 4.5 Synthesis of Metal(II) Complexes

The isolated yields and analytical data for **1**–**8** and **[Ni(HL^7^)_2_]Cl_2_·H_2_O** are summarized in Tables S17 and S18. The experimental CHN contents were within ±0.4% with those calculated, providing evidence for >95% purity.

#### 4.5.1 CuCl_2_(HL^1^)·H_2_O (**1**)

To a solution of **HL^1^** (203 mg, 0.45 mmol) in isopropanol (10 mL) a solution of CuCl_2_·2H_2_O (77 mg, 0.45 mmol) in methanol (500 μL) was added. The mixture was heated at reflux for 1 h, cooled to room temperature, and allowed to stand at 4 °C overnight. The product was filtered off, washed with isopropanol (3 mL), and dried *in vacuo* overnight to give a red-brown powder. Yield: 245 mg. Solubility in water/DMSO 99/1: ≥11 mg mL^–1^.

#### 4.5.2 CuCl_2_(HL^2^)·2H_2_O (**2**)

To a solution of **HL^2^** (100 mg, 0.19 mmol) in isopropanol (20 mL) at 70 °C a solution of CuCl_2_·2H_2_O (32 mg, 0.19 mmol) in methanol (1 mL) was added. The brownish suspension was refluxed for 20 min, cooled to room temperature, and allowed to stand at 4 °C overnight. The precipitate was filtered off, washed with isopropanol (3 mL), and dried *in vacuo* overnight. Yield: 94 mg. Solubility in water/DMSO 99/1: ≥14 mg mL^–1^.

#### 4.5.3 CuCl_2_(HL^3^)·0.2C_3_H_7_OH·0.3H_2_O (**3**)

To a solution of **HL^3^** (73.2 mg, 0.16 mmol) in isopropanol (35 mL) at 70 °C a solution of CuCl_2_·2H_2_O (26.8 mg, 0.16 mmol) in methanol (500 μL) was added. The brown suspension was refluxed for 20 min, cooled to room temperature, and allowed to stand at 4 °C overnight. The resulting precipitate was filtered off, washed with isopropanol (3 mL), and dried *in vacuo* overnight to give a dark-brown solid. Yield: 89 mg. Solubility in water/DMSO 99/1: ≥10 mg mL^–1^.

#### 4.5.4 CuCl_2_(HL^4^)·0.5H_2_O (**4**)

To a solution of **HL^4^** (73.2 mg, 0.135 mmol) in isopropanol (40 mL) at 70 °C a solution of CuCl_2_·2H_2_O (23.0 mg, 0.135 mmol) in methanol (200 μL) was added. The brown suspension was refluxed for 20 min and cooled to 4 °C. After 3 h, the resulting precipitate was filtered off, washed with isopropanol (3 mL), and dried *in vacuo* overnight to give a light-brown solid. Yield: 83 mg.

#### 4.5.5 CuCl_2_(HL^5^)·1.5H_2_O (**5**)

To a solution of **HL^5^** (200 mg, 0.46 mmol) in isopropanol (105 mL) at 70 °C a solution of CuCl_2_·2H_2_O (78 mg, 0.46 mmol) in methanol (1 mL) was added. The dark-red suspension was refluxed for 15 min. After cooling to room temperature, the reaction mixture was allowed to stand at 4 °C overnight. The resulting precipitate was filtered off, washed with isopropanol (3 × 5 mL), and dried *in vacuo* to give a dark-purple solid. Yield: 238 mg.

#### 4.5.6 CuCl_2_(HL^6^)·1.5H_2_O (**6**)

To a solution of **HL^6^** (100 mg, 0.19 mmol) in boiling methanol (55 mL) a solution of CuCl_2_·2H_2_O (33 mg, 0.19 mmol) in methanol (5 mL) was added. The dark-red suspension was refluxed for 15 min. After cooling to room temperature, the solvent was removed under reduced pressure. The residue was suspended in isopropanol (50 mL) and refluxed under vigorous stirring for 10 min. The resulting suspension was allowed to stand at room temperature for 3 h. Then, the precipitate was filtered off, washed with isopropanol (3 × 5 mL), and dried *in vacuo* to give a dark-purple solid. Yield: 113 mg.

#### 4.5.7 CuCl_2_(HL^7^)·0.5H_2_O (**7**)

To a solution of **HL^7^** (150 mg, 0.33 mmol) in boiling methanol (50 mL) a solution of CuCl_2_·2H_2_O (57 mg, 0.33 mmol) in methanol (5 mL) was added. The red suspension was refluxed for 15 min. After cooling to room temperature, the reaction mixture was allowed to stand at room temperature overnight. The resulting precipitate was filtered off, washed with methanol (3 × 5 mL), and dried *in vacuo* to give a vermillion solid. Yield: 183 mg.

#### 4.5.8 [Ni(HL^7^)_2_]Cl_2_·H_2_O

To a boiling solution of **HL^7^** (35 mg, 77.6 μmol) in methanol (5 mL) a solution of NiCl_2_·6H_2_O (9.2 mg, 38.8 μmol) in methanol (0.15 mL) was added. The solution turned red immediately, and the product started to precipitate. The mixture was refluxed for further 30 min, cooled down to room temperature, and allowed to stand at 4 °C overnight. The red product was filtered off, washed with methanol (1 mL), and dried *in vacuo*. Yield: 23.8 mg.

#### 4.5.9 CuCl_2_(HL^8^)·H_2_O (**8**)

To a solution of **HL^8^** (70 mg, 0.13 mmol) in boiling methanol (25 mL) a solution of CuCl_2_·2H_2_O (22.5 mg, 0.13 mmol) in methanol (5 mL) was added. The cherry-red suspension was refluxed for 15 min. After cooling to room temperature, the reaction mixture was allowed to stand at room temperature overnight. The resulting precipitate was filtered off, washed with methanol (3 × 3 mL), and dried *in vacuo* to give a snuff-colored solid. Yield: 83 mg.

^1^H and ^13^C NMR as well as ESI mass spectra of metal free ligands **HL^1^**–**HL^8^**, copper(II)-complexes **1**–**8**, and nickel(II) complex **[Ni(HL^8^)_2_]Cl_2_** are collected in the Supporting Information (Figures S19–S73).

### 4.6 Crystallographic Structure Determination

X-ray diffraction measurements of **[CuCl(L^1^)(DMF)]·DMF**, **[CuCl(L^2^)(CH_3_OH)]**, **[CuCl(L^3^)]·0.5H_2_O**, **[CuCl_2_(H_2_L^5^)]Cl·2DMF**, **HL^6^**, and **[Ni(L^8^)(HL^8^)]Cl·2DMF** were performed on Bruker X8 APEX-II CCD and Bruker D8 Venture diffractometers. Single crystals were positioned at 40, 27, 35, 27, 27, and 30 mm from the detector, and 442, 2302, 1025, 1842, 2420, and 2596 frames were measured, each for 90, 8, 6, 60, 50, and 30 s over 2, 0.4, 0.360, 1, 0.360, and 0.360° scan widths, respectively. Crystal data, data collection parameters, and structure refinement details are given in Table S1. The structures were solved by direct methods and refined by full-matrix least-squares techniques. Non-H atoms were refined with anisotropic displacement parameters. H atoms were inserted in calculated positions and refined with a riding model. The following computer programs and hardware were used: structure solution, *SHELXS-2014* and refinement, *SHELXL-2014*;^101^ molecular diagrams, *ORTEP*; computer, Intel CoreDuo. CCDC 2113207 (**J**), 2113206 (**[CuCl(L^1^)(DMF)]·DMF**), 2113209 (**[CuCl(L^2^)(CH_3_OH)]**), 2113208 (**[CuCl(L^3^)]·0.5H_2_O**), 2113205 (**[CuCl_2_(H_2_L^5^)]Cl·2DMF**), 2113210 (**HL^6^**), and 2126320 **[Ni(L^8^)(HL^8^)]Cl·2DMF**.

### 4.7 Spectrophotometric Solution Equilibrium and Solubility Studies

An Agilent Carry 8454 diode array spectrophotometer was used to record the UV–visible (UV–vis) spectra in the interval 200–800 nm. The path length was 2 or 0.5 cm. Spectrophotometric titrations were performed on samples containing the proligands **HL^1^**, **HL^1^_nm_**, or their copper(II) complexes (**1** and **1_nm_**; see Chart 2) by a KOH solution in the presence of 0.1 M KCl in a DMSO:water 30:70 (w/w) mixture as a solvent at 25.0 ± 0.1 °C in the pH range from 2 to 11. The concentration of **HL^1^_nm_** and **1_nm_** was 12.5 μM in the samples, while **HL^1^** and **1** were titrated at 50 μM due to their somewhat better solubility. An Orion 710A pH meter equipped with a Metrohm combined electrode (type 6.0234.100) and a Metrohm 665 Dosimat burette were used for the pH measurements and titrations. The electrode system was calibrated to the pH = −log[H^+^] scale in the DMSO–water solvent mixture by means of blank titrations (HCl *vs* KOH) similarly to the method suggested by Irving *et al*. in pure aqueous solutions.^102^ The average water ionization constant (p*K*_w_) is 14.52 ± 0.05, which corresponds well to the literature data.^103^ Argon was passed over the solutions during the titrations. Proton dissociation constants (p*K*_a_) of the ligand, overall stability constants (log β) of the copper(II) complexes, and the individual spectra of the various species present in the solution were calculated by the computer program PSEQUAD.^104^ β (M_*p*_L_*q*_H_*r*_) is defined for the general equilibrium *p*M + *q*L + *r*H M_*p*_L_*q*_H_*r*_ as β (M_*p*_L_*q*_H_*r*_) = [M_*p*_L_*q*_H_*r*_]/[M]^*p*^[L]^*q*^[H]^*r*^ where M denotes the copper(II) ion and L the completely deprotonated ligand. The calculations were always made from the experimental titration data measured in the absence of any precipitate in the solution.

Thermodynamic solubility of proligands (**HL^5^** and **HL^5^_nm_**) and copper(II) complexes (**3**, **5**, **5_nm_**, **6**, **6_nm_**, **7**, and **8**) was measured for the saturated solutions in water at pH 5 (20 mM 2-(*N*-morpholino)ethanesulfonic acid (MES, Sigma Aldrich) buffer) and 7.4 (20 mM 4-(2-hydroxyethyl)-1-piperazineethanesulfonic acid (HEPES, Sigma Aldrich) buffer) at 25.0 ± 0.1 °C. The concentration of the compounds was determined by UV–vis spectrophotometry using stock solutions of the compounds with a known concentration dissolved in pure DMSO and 50% (v/v) DMSO/buffered aqueous solution for the calibration.

Aqueous stability of **4**, **8**, and **HL^4^** was investigated in phosphate-buffered saline (PBS) and 10 mM HEPES buffers and in three-times diluted blood serum (Sigma Aldrich, from human male AB plasma) at pH = 7.40. The concentration of the compounds was between 5 and 10 μM. Blood serum was filtered on a 1.25 μm polyethersulfone membrane syringe filter and diluted with 10 mM HEPES or PBS buffer.

### 4.8 DNA Binding Studies

Fluorescence measurements were carried out on a Fluoromax (Horiba Jobin Yvon) fluorometer. A stock solution of calf thymus DNA (ct-DNA, Sigma Aldrich) was prepared as described in our former work.^105^ Samples contained 10 μM ct-DNA expressed in base pairs, 5 μM ethidium bromide (EtBr, Sigma Aldrich), and different concentrations of complexes **4** or **8** or proligands **HL^4^** or **HL^8^** in 10 mM HEPES buffer (pH = 7.40). The excitation wavelengths were 510 or 455 nm, and the fluorescence emission was measured in the range 530–750 nm. Corrections for self-absorbance and inner filter effect were done according to our former work.^106^ Fluorescence lifetime was measured on the same fluorometer equipped with a DeltaHub time-correlated single photon counting (TCSPC) controller using a NanoLED light source N-455 (Horiba Jobin Yvon). Details on the instrument parameters are found in Table S3. The fluorescence intensity decay over time is described by a sum of exponentials,
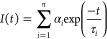
3where α_*i*_ and τ_*i*_ are the contribution to the total intensity (*I*) at time point 0 and lifetime of component *i*, respectively.^107^ The quality of the fit was judged from a χ^2^_R_ value close to 1.0 and a random distribution of weighted residuals. Fractional intensities were calculated as described in our former work.^105^

### 4.9 Cell Lines

Human colonic adenocarcinoma cell lines Colo 205 doxorubicin-sensitive (ATCC-CCL-222) and Colo 320/MDR-LRP multidrug resistant over-expressing ABCB1 (MDR1)-LRP (ATCC-CCL-220.1) were purchased from LGC Promochem, Teddington, UK. The cells were cultured in an RPMI 1640 medium supplemented with 10% heat-inactivated fetal bovine serum, 2 mM l-glutamine, 1 mM Na-pyruvate, and 10 mM HEPES. The semi-adherent human colon cancer cells were detached with Trypsin-Versene (EDTA) solution for 5 min at 37 °C. An MRC-5 human embryonal lung fibroblast cell line (ATCC CCL-171) was purchased from LGC Promochem, Teddington, UK. The cells were cultured in Eagle’s minimal essential medium (EMEM, containing 4.5 g L^–1^ glucose) supplemented with a non-essential amino acid mixture, a selection of vitamins, and 10% heat-inactivated fetal bovine serum. The HCT-116 colon carcinoma cells and isogenic p53-knock-out subline were a gift of Prof. Bert Vogelstein from The Johns Hopkins Oncology Center. Cells were cultured in McCoy’s 5A medium supplemented with 10% heat-inactivated fetal bovine serum and 4 mM l-glutamine. All cell lines were incubated at 37 °C, in a 5% CO_2_, 95% air atmosphere.

### 4.10 Assay for Cytotoxic Effect

MRC-5 non-cancerous human embryonic lung fibroblast and human colonic adeno-carcinoma cell lines (doxorubicin-sensitive Colo 205 and multidrug resistant Colo 320 colonic adenocarcinoma cells) were used to determine the effect of compounds on cell growth. The effects of increasing concentrations of compounds on cell growth were tested in 96-well flat-bottomed microtiter plates. The compounds were dissolved in DMSO and stock solutions of 10 mM were prepared. These were further diluted in the appropriate cell culture medium by twofold serial dilution starting from 100 or 10 μM for the compounds. The adherent human embryonal lung fibroblast cells were cultured in 96-well flat-bottomed microtiter plates using EMEM supplemented with 10% heat-inactivated fetal bovine serum. The density of the cells was adjusted to 1 × 10^4^ cells in 100 μL per well, the cells were seeded for 24 h at 37 °C, 5% CO_2_, then the medium was removed from the plates containing the cells, and the dilutions of compounds previously made in a separate plate were added to the cells in 200 μL. In the case of the colonic adenocarcinoma cells, the twofold serial dilutions of compounds were prepared in 100 μL of RPMI 1640, horizontally. The semi-adherent colonic adenocarcinoma cells were treated with Trypsin-Versene (EDTA) solution. They were adjusted to a density of 1 × 10^4^ cells in 100 μL of RPMI 1640 medium and were added to each well, with the exception of the medium control wells. The final volume of the wells containing compounds and cells was 200 μL. The culture plates were incubated at 37 °C for 72 h; at the end of the incubation period, 20 μL of MTT (thiazolyl blue tetrazolium bromide, Sigma) solution (from a stock solution of 5 mg mL^–1^) was added to each well. After incubation at 37 °C for 4 h, 100 μL of sodium dodecyl sulfate (SDS) (Sigma) solution (10% in 0.01 M HCI) was added to each well and the plates were further incubated at 37 °C overnight. Cell growth was determined by measuring the optical density (OD) at 540/630 nm with a Multiscan EX ELISA reader (Thermo Labsystems, Cheshire, WA, USA). Inhibition of the cell growth (expressed as IC_50_: inhibitory concentration that reduces by 50% the growth of the cells exposed to the tested compounds) was determined from the sigmoid curve where 100 – ((OD_sample_ – OD_medium control_)/(OD_cell control_ – OD_medium control_)) × 100 values were plotted against the logarithm of compound concentrations. Curves were fitted by GraphPad Prism software^57^ using the sigmoidal dose–response model (comparing variable and fixed slopes). The IC_50_ values were obtained from at least three independent experiments. Tests in adherent HCT-116 colon carcinoma cells and an isogenic *p53*-knock-out subline thereof were performed in a similar manner, with 1.5 × 10^3^ cells seeded in 100 μL of McCoy’s 5A medium (Sigma-Aldrich) per well. From fresh stock solutions in DMSO (10 mM **HL^4^** and **HL^8^** and 2 mM **4** and **8**), test compounds were diluted in a medium in adjusted concentration ranges and applied for 96 h. Upon removal of the medium containing the test compounds and 4 h of staining with 100 μL of MTT/medium mixture, the latter was exchanged for 150 μL of DMSO per well and photometric measurement performed immediately thereafter.

### 4.11 Mechanisms of Cell Death: Assay for Apoptosis Induction

The assay was carried out for selected compounds using an Annexin V-FITC Apoptosis Detection Kit (cat. no. APOAF-50TST) from Sigma according to the manufacturer’s instructions. The concentration of the Colo320 cell suspension was adjusted to approximately 0.5 × 10^6^ cells/mL in an RPMI 1640 medium, and the cell suspension was distributed in 1 mL of aliquots into a 24-well plate and then incubated overnight at 37 °C and 5% CO_2_. On the following day, the medium was removed and replaced by 1 mL of RPMI medium containing the compounds except the control samples. Colo320 cells were incubated in the presence of the compounds at 2 and 4 μM (**HL^8^** and **HL^4^**), or 0.5 and 2 μM (**8**), or 0.25 and 0.5 μM (**4**), in the 24-well plate at 37 °C for 3 h, and 12*H*-benzo[α]phenothiazine (M627, 20 μM)^108^ and cisplatin (Teva, 15 and 30 μM) were used as positive controls. After the incubation period, the samples were washed with PBS and fresh RPMI 1640 medium was added to the samples. The cells were incubated overnight at 37 °C and 5% CO_2_. The next day, 200 μL of 0.25% Trypsin (Trypsin-Versen) was added to the samples until cells appeared detached followed by the addition of 400 μL of RPMI 1640 medium supplemented with 10% bovine serum. The cells were collected in Eppendorf tubes and centrifuged at 2000*g* for 2 min. The harvested cells were resuspended in fresh serum-free RPMI 1640 culture medium. After this step, the apoptosis assay was carried out according to the instructions of the manufacturer. The fluorescence was analyzed immediately using a ParTec CyFlow flow cytometer (Partec GmbH, Münster, Germany).

### 4.12 Enzyme Inhibition Tests

All kinase assays were carried out using Multidrop 384’s at room temperature in a total assay volume of 25.5 μL. To plates containing 0.5 μL of **HL^8^** and **8**, DMSO control or acid blank (0.51 mM), 15 μL of an enzyme mix containing enzyme (0.51 mM), and excess peptide/protein substrate in buffer was added. Compounds were pre-incubated in the presence of the enzyme and peptide/protein substrate for 5 min before initiation of the reaction by addition of 10 μL of ATP (final concentration selected for each kinase at 5, 20, or 50 μM). Assays were carried out for 30 min at room temperature before termination by the addition of 5 μL of orthophosphoric acid. The assay plates were then harvested onto P81 Unifilter Plates by a PerkinElmer Harvester and air dried. The dry Unifilter plates were then sealed on the addition of MicroScint O and were counted in PerkinElmer Topcount scintillation counters.

### 4.13 Determination of IC_50_ Values against Protein Kinases

IC_50_ values were determined by the International Centre for Kinase Profiling at the University of Dundee according to standard protocols published elsewhere.^109,110^

### 4.14 Molecular Docking

The ligands and complexes were docked against the crystal structures of PIM-1 (PDB ID: 1YXX, resolution: 2.00 Å),^85^ calmodulin-dependent protein kinase I G (CaMK-1) (PDB ID: 2JAM, resolution: 1.70 Å), GSK3β (PDB ID: 3I4B, resolution: 2.30 Å),^87^ PKA (PDB ID: 3OF1, resolution: 2.21 Å),^88^ and SGK-1 (PDB ID: 3HDM, resolution: 2.60 Å)^89^ kinases, which were obtained from the Protein Data Bank (PDB).^111,112^ The GOLD (v2020.2.0) software suite was used to prepare the crystal structures for docking, i.e., the hydrogen atoms were added, water molecules deleted, and the co-crystallized ligands identified: PIM-1: LI7 ((3*E*)-3-[(4-hydroxyphenyl)imino]-1*H*-indol-2(3*H*)-one), CAMK-1: J60 (5-[(*E*)-(5-chloro-2-oxo-1,2-dihydro-3*H*-indol-3-ylidene)methyl]-*N*-[2-(diethylamino)ethyl]-2,4-dimethyl-1*H*-pyrrole-3-carboxamide), and GSK3β: Z48 (*N*-[(1*S*)-2-hydroxy-1-phenylethyl]-4-[5-methyl-2-(phenylamino)pyrimidin-4-yl]-1*H*-pyrrole-2-carboxamide), PKA: CMP (adenosine-3′,5′-cyclic-monophosphate), and SGK-1: MMG (4-(5-phenyl-1*H*-pyrrolo[2,3-*b*]pyridin-3-yl)benzoic acid). The docking center for the binding pockets was defined as the position of the co-crystallized ligands with a 10 Å radius. The GoldScore (GS)^90^ and ChemScore (CS)^91,92^ ChemPLP (Piecewise Linear Potential)^93^ and ASP (Astex Statistical Potential)^94^ scoring functions were implemented to predict the binding modes and relative energies of the ligands using the GOLD (v2020.2.0) software suite. The GOLD docking algorithm is reported to be an excellent modeling tool.^113,114^ The Scigress version FJ 2.6 program^96^ was used to build the ligands and complexes; the MM3^115−117^ force field was applied to identify the global minimum using the CONFLEX method^118^ followed by structural optimization. For other detail of molecular docking calculations, see the Supporting Information.

## Supporting Information Available

Numbering schemes for precursors **I** and **J** (Chart S1) and for latonduine- and indolo[2,3-*c*]quinoline derivatives (Chart S2), line drawings of previously reported Cu(II) complexes of paullones (Chart S3), ORTEP view of species **J** (Figure S1), association of molecules of **[CuCl(L^3^)]** into a dimer (Figure S2), ORTEP view of **HL^6^** (Figure S3), ORTEP view of the complex cation in **[Ni(L^8^)(HL^8^)]Cl·2DMF** (Figure S4), crystallographic data and details of data collection and refinement (Table S1), UV–vis absorption and emission spectra (Figures S5–S9), parameters for DNA-binding studies (Tables S2 and S3), enzyme inhibition assay data (Table S4), IC_50_ values of **HL^8^** and **8** against five different kinases (Table S5 and Figures S10–S15), molecular docking details (Figures S16–S18 and Tables S6–S14), yields and analytical data of **HL^1^**–**HL^8^** and Cu(II) complexes **1**–**8** (Tables S15–S18), ^1^H and ^13^C NMR data (Figures S19–S46), ESI mass spectra (Figures S47–S73), and HPLC-HR ESI mass spectra (Figures S74–S80) (PDF)
Molecular formula strings of proligands and copper(II) complexes (CSV)
Information for the 3D structure of CaMK-1 (PDB)
Information for the 3D structure of GSK3β (PDB)
Information for the 3D structure of PIM1 (PDB)
Docked poses of **HL^8^** in the PIM-1’s binding site (PDB)
Information for the 3D structure of PKA1 (PDB)
Information for the 3D structure of SGK1 (PDB)
Crystallographic Information File (CIF) for **[CuCl(L^1^)(DMF)]·DMF** (CIF)
CIF for species **J** (CIF)
CIF for **[CuCl(L^3^)]·0.5H_2_O** (CIF)
CIF for **[CuCl(L^2^)(CH_3_OH)]** (CIF)
CIF for **[CuCl_2_(H_2_L^5^)]Cl·2DMF** (CIF)
CIF for **HL^6^** (CIF)
CIF for **[Ni(L^8^)(HL^8^)] Cl·2DMF** (CIF)

## Supplementary Material

jm1c01740_si_002.csv

jm1c01740_si_003.pdb

jm1c01740_si_004.pdb

jm1c01740_si_005.pdb

jm1c01740_si_006.pdb

jm1c01740_si_007.pdb

jm1c01740_si_008.pdb

jm1c01740_si_010.cif

jm1c01740_si_012.cif

jm1c01740_si_014.cif

jm1c01740_si_016.cif

jm1c01740_si_018.cif

jm1c01740_si_020.cif

jm1c01740_si_021.cif

## ABBREVIATIONS

ASP: Astex Statistical Potential
Boc_2_O: di-*tert*-butyl dicarbonate
CAMK-1: calcium/calmodulin-dependent protein kinase
ChemPLP: Piecewise Linear Potential
CS: ChemScore
Cdk: cyclin dependent kinase
DNA: deoxyribonucleic acid
DCM: dichloromethane
DMAP: 4-dimethylaminopyridine
DMF: dimethylformamide
DMSO: dimethyl sulfoxide
GSK3β: glycogen synthase kinase-3 beta
GS: GoldScore
HEPES: 4-(2-hydroxyethyl)-1-piperazineethanesulfonic acid
HA: hydrogen bond acceptors
HD: hydrogen bond donors
KDIs: Known Drug Indexes
KDS: Known Drug Space
KRAS: Kirsten Rat Sarcoma
log *P*: octanol–water partition coefficient
MDA: microtubule destabilizing agent
MSK1: ribosomal protein S6 kinase alpha-5
MW: molecular weight
PBS: phosphate-buffered saline
PI: propidium iodide
PKA: cAMP-dependent protein kinase
PSA: polar surface area
RMSD: root-mean-square deviation
RB: rotatable bonds
SGK-1: serum and glucocortoid-regulated kinase
SC-XRD: single crystal X-ray diffraction
THF: tetrahydrofuran
topo I/II: topoisomerase I/II

## References

[ref1] Lee S.; Lee S.; Cheon C.-H. Concise Total Syntheses of Paullone and Kenpaullone via Cyanide-Catalyzed Intramolecular Imino-Stetter Reaction. Synthesis 2017, 49, 4247–4253. 10.1055/s-0036-1588749.

[ref2] Koutsandrea E. G.; Fousteris M. A.; Nikolaropoulos S. S. Synthesis of New Tetracyclic Paullone Derivatives as Potential CDK Inhibitors. Heterocycl. Commun. 2012, 18, 169–179. 10.1515/hc-2012-0121.

[ref3] Akunuri R.; Vadakattu M.; Bujji S.; Veerareddy V.; Madhavi Y. V.; Nanduri S. Fused-Azepinones: Emerging Scaffolds of Medicinal Importance. Eur. J. Med. Chem. 2021, 220, 113445 10.1016/j.ejmech.2021.113445. 33901899

[ref4] Singh A. K.; Raj V.; Saha S. Indole-Fused Azepines and Analogues as Anticancer Lead Molecules: Privileged Findings and Future Directions. Eur. J. Med. Chem. 2017, 142, 244–265. 10.1016/j.ejmech.2017.07.042. 28803677

[ref5] Wang N.; Świtalska M.; Wang L.; Shaban E.; Hossain M. I.; El Sayed I. E. T.; Wietrzyk J.; Inokuchi T. Structural Modifications of Nature-Inspired Indoloquinolines: A Mini Review of Their Potential Antiproliferative Activity. Molecules 2019, 24, 2121 10.3390/molecules24112121. PMC6600460 31195640

[ref6] Nadein O. N.; Aksenov D. A.; Abakarov G. M.; Aksenov N. A.; Voskressensky L. G.; Aksenov A. V. Methods of Synthesis of Natural Indoloquinolines Isolated from Cryptolepis Sanguinolenta. Chem. Heterocycl. Compd. 2019, 55, 905–932. 10.1007/s10593-019-02557-8.

[ref7] Schultz C.; Link A.; Leost M.; Zaharevitz D. W.; Gussio R.; Sausville E. A.; Meijer L.; Kunick C. Paullones, a Series of Cyclin-Dependent Kinase Inhibitors: Synthesis, Evaluation of CDK1/Cyclin B Inhibition, and in Vitro Antitumor Activity. J. Med. Chem. 1999, 42, 2909–2919. 10.1021/jm9900570. 10425100

[ref8] Singh A. K.; Bhadauria A. S.; Kumar U.; Raj V.; Rai A.; Kumar P.; Keshari A. K.; Kumar D.; Maity B.; Nath S.; Prakash A.; Saha S. Novel Indole-Fused Benzo-oxazepine (IFBOs) Inhibit Invasion of Hepatocellular Carcinoma by Targeting IL-6 Mediated JAK2/STAT3 Oncogenic Signals. Sci. Rep. 2018, 8, 5932 10.1038/s41598-018-24288-0. 29651140 PMC5897576

[ref9] Gentles R. G. Discovery of Beclabuvir: A Potent Allosteric Inhibitor of the Hepatitis C Virus Polymerase. In HCV: The Journey from Discovery to a Cure; Sofia M. J., Ed.; Springer International Publishing: Cham, 2019; Vol. 31, pp. 193–228.

[ref10] Funke A.; Weisz K. Comprehensive Thermodynamic Profiling for the Binding of a G-Quadruplex Selective Indoloquinoline. J. Phys. Chem. B 2017, 121, 5735–5743. 10.1021/acs.jpcb.7b02686. 28531353

[ref11] Thanetchaiyakup A.; Borwornpinyo S.; Rattanarat H.; Kanjanasirirat P.; Jearawuttanakul K.; Seemakhan S.; Chuanopparat N.; Ngernmeesri P. Copper-Catalyzed Synthesis and Anticancer Activity Evaluation of Indolo[1,2-*a*]quinoline Derivatives. Tetrahedron Lett. 2021, 82, 153365 10.1016/j.tetlet.2021.153365.

[ref12] Altwaijry N.; El-Ghlban S.; El Sayed I. E.-T.; El-Bahnsawye M.; Bayomi A. I.; Samaka R. M.; Shaban E.; Elmongy E. I.; El-Masry T. A.; Ahmed H. M. A.; Attallah N. G. M. In Vitro and In Vivo Antitumor Activity of Indolo[2,3-*b*]quinolines, Natural Product Analogs from Neocryptolepine Alkaloid. Molecules 2021, 26, 754 10.3390/molecules26030754. 33535575 PMC7867085

[ref13] Shang X.-F.; Dai L.-X.; Zhang Z.-J.; Yang C.-J.; Du S.-S.; Wu T.-L.; He Y.-H.; Zhu J.-K.; Liu Y.-Q.; Yan Y.-F.; Miao X.-L.; Zhang J.-Y. Integrated Proteomics and Transcriptomics Analyses Reveals the Possible Antifungal Mechanism of an Indoloquinoline Alkaloid Neocryptolepine against *Rhizoctonia Solani*. J. Agric. Food Chem. 2021, 69, 6455–6464. 10.1021/acs.jafc.1c01385. 34075744

[ref14] Chowdhury S.; Bhuiya S.; Das S. Comparative Binding Studies on the Interaction of the Indoloquinoline Alkaloid Cryptolepine with the B and the Non-Canonical Protonated Form of DNA: A Spectroscopic Insight. Biochim. Biophys. Acta 2021, 1865, 129993 10.1016/j.bbagen.2021.129993. 34453987

[ref15] Hsueh W.-Y.; Lee Y.-S. E.; Huang M.-S.; Lai C.-H.; Gao Y.-S.; Lin J.-C.; Chen Y.-F.; Chang C.-L.; Chou S.-Y.; Chen S.-F.; Lu Y.-Y.; Chang L.-H.; Lin S. F.; Lin Y.-H.; Hsu P.-C.; Wei W.-Y.; Huang Y.-C.; Kao Y.-F.; Teng L.-W.; Liu H.-H.; Chen Y.-C.; Yuan T.-T.; Chan Y.-W.; Huang P.-H.; Chao Y.-T.; Huang S.-Y.; Jian B.-H.; Huang H.-Y.; Yang S.-C.; Lo T.-H.; Huang G.-R.; Wang S.-Y.; Lin H.-S.; Chuang S.-H.; Huang J.-J. Copper(I)-Catalyzed Nitrile-Addition/*N*-Arylation Ring-Closure Cascade: Synthesis of 5,11-Dihydro-6*H*-indolo[3,2-*c*]quinolin-6-ones as Potent Topoisomerase-I Inhibitors. J. Med. Chem. 2021, 64, 1435–1453. 10.1021/acs.jmedchem.0c00727. 33492141

[ref16] Vianney Y. M.; Preckwinkel P.; Mohr S.; Weisz K. Quadruplex–Duplex Junction: A High-Affinity Binding Site for Indoloquinoline Ligands. Chem. – Eur. J. 2020, 26, 16910–16922. 10.1002/chem.202003540. 32975874 PMC7756412

[ref17] Håheim K. S.; Lindbäck E.; Tan K. N.; Albrigtsen M.; Urdal Helgeland I. T.; Lauga C.; Matringe T.; Kennedy E. K.; Andersen J. H.; Avery V. M.; Sydnes M. O. Synthesis and Evaluation of the Tetracyclic Ring-System of Isocryptolepine and Regioisomers for Antimalarial, Antiproliferative and Antimicrobial Activities. Molecules 2021, 26, 3268 10.3390/molecules26113268. 34070798 PMC8198049

[ref18] Leost M.; Schultz C.; Link A.; Wu Y.-Z.; Biernat J.; Mandelkow E.-M.; Bibb J. A.; Snyder G. L.; Greengard P.; Zaharevitz D. W.; Gussio R.; Senderowicz A. M.; Sausville E. A.; Kunick C.; Meijer L. Paullones Are Potent Inhibitors of Glycogen Synthase Kinase-3β and Cyclin-Dependent Kinase 5/P25: Paullones Inhibit GSK-3β and CDK5/P25. Eur. J. Biochem. 2000, 267, 5983–5994. 10.1046/j.1432-1327.2000.01673.x. 10998059

[ref19] Pinna L. A.; Cohen P. T. W. Inhibitors of Protein Kinases and Protein Phosphates; Springer-Verlag Berlin Heidelberg: Berlin, Heidelberg, 2005, 10.1007/b137900.

[ref20] Soto S.; Vaz E.; Dell’Aversana C.; Álvarez R.; Altucci L.; de Lera Á. R. New Synthetic Approach to Paullones and Characterization of Their SIRT1 Inhibitory Activity. Org. Biomol. Chem. 2012, 10, 2101–2112. 10.1039/c2ob06695e. 22286328

[ref21] Denis J. G.; Franci G.; Altucci L.; Aurrecoechea J. M.; de Lera Á. R.; Álvarez R. Synthesis of 7-Alkylidene-7,12-dihydroindolo[3,2-*d*]benzazepine-6-(5*H*)-ones (7-Alkylidene-Paullones) by N-Cyclization–Oxidative Heck Cascade and Characterization as Sirtuin Modulators. Org. Biomol. Chem. 2015, 13, 2800–2810. 10.1039/C4OB02493A. 25604354

[ref22] Chen Q.; Cui W.; Cheng Y.; Zhang F.; Ji M. Studying the Mechanism That Enables Paullones to Selectively Inhibit Glycogen Synthase Kinase 3 Rather than Cyclin-Dependent Kinase 5 by Molecular Dynamics Simulations and Free-Energy Calculations. J. Mol. Model. 2011, 17, 795–803. 10.1007/s00894-010-0762-0. 20559856

[ref23] Knockaert M.; Wieking K.; Schmitt S.; Leost M.; Grant K. M.; Mottram J. C.; Kunick C.; Meijer L. Intracellular Targets of Paullones. J. Biol. Chem. 2002, 277, 25493–25501. 10.1074/jbc.M202651200. 11964410

[ref24] Ibrahim E.-S.; Montgomerie A. M.; Sneddon A. H.; Proctor G. R.; Green B. Synthesis of Indolo[3,2-*c*]quinolines and Indolo[3,2-*d*]benzazepines and Their Interaction with DNA. Eur. J. Med. Chem. 1988, 23, 183–188. 10.1016/0223-5234(88)90192-4.

[ref25] Lu C.-M.; Chen Y.-L.; Chen H.-L.; Chen C.-A.; Lu P.-J.; Yang C.-N.; Tzeng C.-C. Synthesis and Antiproliferative Evaluation of Certain Indolo[3,2-*c*]quinoline Derivatives. Bioorg. Med. Chem. 2010, 18, 1948–1957. 10.1016/j.bmc.2010.01.033. 20171108

[ref26] Wang N.; Świtalska M.; Wu M.-Y.; Imai K.; Ngoc T. A.; Pang C.-Q.; Wang L.; Wietrzyk J.; Inokuchi T. Synthesis and in Vitro Cytotoxic Effect of 6-Amino-Substituted 11H- and 11Me-indolo[3,2-*c*]quinolines. Eur. J. Med. Chem. 2014, 78, 314–323. 10.1016/j.ejmech.2014.03.038. 24686018

[ref27] Lavrado J.; Brito H.; Borralho P. M.; Ohnmacht S. A.; Kim N.-S.; Leitão C.; Pisco S.; Gunaratnam M.; Rodrigues C. M. P.; Moreira R.; Neidle S.; Paulo A. KRAS Oncogene Repression in Colon Cancer Cell Lines by G-Quadruplex Binding Indolo[3,2-*c*]quinolines. Sci. Rep. 2015, 5, 9696 10.1038/srep09696. 25853628 PMC5382548

[ref28] Linington R. G.; Williams D. E.; Tahir A.; Van Soest R.; Andersen R. J. Latonduines A and B, New Alkaloids Isolated from the Marine Sponge Stylissa Carteri: Structure Elucidation, Synthesis, and Biogenetic Implications. Org. Lett. 2003, 5, 2735–2738. 10.1021/ol034950b. 12868902

[ref29] Fouad M. A.; Debbab A.; Wray V.; Müller W. E. G.; Proksch P. New Bioactive Alkaloids from the Marine Sponge *Stylissa Sp*. Tetrahedron 2012, 68, 10176–10179. 10.1016/j.tet.2012.09.097.

[ref30] Putey A.; Popowycz F.; Do Q.-T.; Bernard P.; Talapatra S. K.; Kozielski F.; Galmarini C. M.; Joseph B. Indolobenzazepin-7-ones and 6-, 8-, and 9-Membered Ring Derivatives as Tubulin Polymerization Inhibitors: Synthesis and Structure–Activity Relationship Studies. J. Med. Chem. 2009, 52, 5916–5925. 10.1021/jm900476c. 19743863

[ref31] Keller L.; Beaumont S.; Liu J.-M.; Thoret S.; Bignon J. S.; Wdzieczak-Bakala J.; Dauban P.; Dodd R. H. New C5-Alkylated Indolobenzazepinones Acting as Inhibitors of Tubulin Polymerization: Cytotoxic and Antitumor Activities. J. Med. Chem. 2008, 51, 3414–3421. 10.1021/jm701466p. 18503262

[ref32] Primik M. F.; Göschl S.; Jakupec M. A.; Roller A.; Keppler B. K.; Arion V. B. Structure–Activity Relationships of Highly Cytotoxic Copper(II) Complexes with Modified Indolo[3,2-*c*]quinoline Ligands. Inorg. Chem. 2010, 49, 11084–11095. 10.1021/ic101633z. 20979395

[ref33] Primik M. F.; Mühlgassner G.; Jakupec M. A.; Zava O.; Dyson P. J.; Arion V. B.; Keppler B. K. Highly Cytotoxic Copper(II) Complexes with Modified Paullone Ligands. Inorg. Chem. 2010, 49, 302–311. 10.1021/ic902042a. 19968251

[ref34] Dobrov A.; Göschl S.; Jakupec M. A.; Popović-Bijelić A.; Gräslund A.; Rapta P.; Arion V. B. A Highly Cytotoxic Modified Paullone Ligand Bearing a TEMPO Free-Radical Unit and Its Copper(II) Complex as Potential hR2 RNR Inhibitors. Chem. Commun. 2013, 49, 10007–10009. 10.1039/c3cc45743e. PMC4047831 24042148

[ref35] Filak L. K.; Göschl S.; Heffeter P.; Ghannadzadeh Samper K.; Egger A. E.; Jakupec M. A.; Keppler B. K.; Berger W.; Arion V. B. Metal–Arene Complexes with Indolo[3,2- *c* ]-Quinolines: Effects of Ruthenium vs Osmium and Modifications of the Lactam Unit on Intermolecular Interactions, Anticancer Activity, Cell Cycle, and Cellular Accumulation. Organometallics 2013, 32, 903–914. 10.1021/om3012272. 23431223 PMC3573711

[ref36] Filak L. K.; Mühlgassner G.; Jakupec M. A.; Heffeter P.; Berger W.; Arion V. B.; Keppler B. K. Organometallic Indolo[3,2-*c*]quinolines versus Indolo[3,2-*d*]benzazepines: Synthesis, Structural and Spectroscopic Characterization, and Biological Efficacy. J. Biol. Inorg. Chem. 2010, 15, 903–918. 10.1007/s00775-010-0653-y. 20369265 PMC2908761

[ref37] Filak L. K.; Mühlgassner G.; Bacher F.; Roller A.; Galanski M.; Jakupec M. A.; Keppler B. K.; Arion V. B. Ruthenium– and Osmium–Arene Complexes of 2-Substituted Indolo[3,2-*c*]quinolines: Synthesis, Structure, Spectroscopic Properties, and Antiproliferative Activity. Organometallics 2011, 30, 273–283. 10.1021/om101004z. 21253447 PMC3022494

[ref38] Stepanenko I. N.; Casini A.; Edafe F.; Novak M. S.; Arion V. B.; Dyson P. J.; Jakupec M. A.; Keppler B. K. Conjugation of Organoruthenium(II) 3-(1*H*-Benzimidazol-2-yl)pyrazolo[3,4-*b*]pyridines and Indolo[3,2-*d*]benzazepines to Recombinant Human Serum Albumin: A Strategy To Enhance Cytotoxicity in Cancer Cells. Inorg. Chem. 2011, 50, 12669–12679. 10.1021/ic201801e. 22111668 PMC3255472

[ref39] Arion V. B.; Dobrov A.; Göschl S.; Jakupec M. A.; Keppler B. K.; Rapta P. Ruthenium- and Osmium-Arene-Based Paullones Bearing a TEMPO Free-Radical Unit as Potential Anticancer Drugs. Chem. Commun. 2012, 48, 8559–8561. 10.1039/c2cc33786j. 22797078

[ref40] Schmid W. F.; John R. O.; Mühlgassner G.; Heffeter P.; Jakupec M. A.; Galanski M.; Berger W.; Arion V. B.; Keppler B. K. Metal-Based Paullones as Putative CDK Inhibitors for Antitumor Chemotherapy. J. Med. Chem. 2007, 50, 6343–6355. 10.1021/jm701042w. 17997519

[ref41] Pennington L. D.; Moustakas D. T. The Necessary Nitrogen Atom: A Versatile High-Impact Design Element for Multiparameter Optimization. J. Med. Chem. 2017, 60, 3552–3579. 10.1021/acs.jmedchem.6b01807. 28177632

[ref42] Bacher F.; Dömötör O.; Chugunova A.; Nagy N. V.; Filipović L.; Radulović S.; Enyedy É. A.; Arion V. B. Strong Effect of Copper(II) Coordination on Antiproliferative Activity of Thiosemicarbazone–Piperazine and Thiosemicarbazone–Morpholine Hybrids. Dalton Trans. 2015, 44, 9071–9090. 10.1039/C5DT01076D. 25896351

[ref43] Bacher F.; Wittmann C.; Nové M.; Spengler G.; Marć M. A.; Enyedy E. A.; Darvasiová D.; Rapta P.; Reiner T.; Arion V. B. Novel Latonduine Derived Proligands and Their Copper(II) Complexes Show Cytotoxicity in the Nanomolar Range in Human Colon Adenocarcinoma Cells and *in Vitro* Cancer Selectivity. Dalton Trans. 2019, 48, 10464–10478. 10.1039/C9DT01238A. 31125040 PMC6635014

[ref44] Ohui K.; Afanasenko E.; Bacher F.; Ting R. L. X.; Zafar A.; Blanco-Cabra N.; Torrents E.; Dömötör O.; May N. V.; Darvasiova D.; Enyedy É. A.; Popović-Bijelić A.; Reynisson J.; Rapta P.; Babak M. V.; Pastorin G.; Arion V. B. New Water-Soluble Copper(II) Complexes with Morpholine–Thiosemicarbazone Hybrids: Insights into the Anticancer and Antibacterial Mode of Action. J. Med. Chem. 2019, 62, 512–530. 10.1021/acs.jmedchem.8b01031. 30507173 PMC6348444

[ref45] Faivre S.; Djelloul S.; Raymond E. New Paradigms in Anticancer Therapy: Targeting Multiple Signaling Pathways With Kinase Inhibitors. Semin. Oncol. 2006, 33, 407–420. 10.1053/j.seminoncol.2006.04.005. 16890796

[ref46] Krug M.; Hilgeroth A. Recent Advances in the Development of Multi-Kinase Inhibitors. Mini-Rev. Med. Chem. 2008, 8, 1312–1327. 10.2174/138955708786369591. 18991750

[ref47] Garuti L.; Roberti M.; Bottegoni G. Multi-Kinase Inhibitors. Curr. Med. Chem. 2015, 22, 695–712. 10.2174/0929867321666141216125528. 25511779

[ref48] Bhullar K. S.; Lagarón N. O.; McGowan E. M.; Parmar I.; Jha A.; Hubbard B. P.; Rupasinghe H. P. V. Kinase-Targeted Cancer Therapies: Progress, Challenges and Future Directions. Mol. Cancer 2018, 17, 48 10.1186/s12943-018-0804-2. 29455673 PMC5817855

[ref49] Guo T.; Ma S. Recent Advances in the Discovery of Multitargeted Tyrosine Kinase Inhibitors as Anticancer Agents. ChemMedChem 2021, 16, 600–620. 10.1002/cmdc.202000658. 33179854

[ref50] Putey A.; Joucla L.; Picot L.; Besson T.; Joseph B. Synthesis of Latonduine Derivatives via Intramolecular Heck Reaction. Tetrahedron 2007, 63, 867–879. 10.1016/j.tet.2006.11.042.

[ref51] Primik M. F.; Filak L. K.; Arion V. B. Metal-Based Indolobenzazepines and Indoloquinolines: From Moderate Cdk Inhibitors to Potential Antitumor Drugs. In Advances in Organometallic Chemistry and Catalysis: The Silver/Gold Jubilee International Conference on Organometallic Chemistry Celebratory Book; John Wiley & Sons, Inc., 2014.

[ref52] Filak L. K.; Kalinowski D. S.; Bauer T. J.; Richardson D. R.; Arion V. B. Effect of the Piperazine Unit and Metal-Binding Site Position on the Solubility and Anti-Proliferative Activity of Ruthenium(II)- and Osmium(II)-Arene Complexes of Isomeric Indolo[3,2-*c*]quinoline-Piperazine Hybrids. Inorg. Chem. 2014, 53, 6934–6943. 10.1021/ic500825j. 24927493 PMC4087041

[ref53] Primik M. F.; Göschl S.; Meier S. M.; Eberherr N.; Jakupec M. A.; Enyedy É. A.; Novitchi G.; Arion V. B. Dicopper(II) and Dizinc(II) Complexes with Nonsymmetric Dinucleating Ligands Based on Indolo[3,2-*c*]quinolines: Synthesis, Structure, Cytotoxicity, and Intracellular Distribution. Inorg. Chem. 2013, 52, 10137–10146. 10.1021/ic401573d. 23952332 PMC3763518

[ref54] Addison A. W.; Rao T. N.; Reedijk J.; van Rijn J.; Verschoor G. C. Synthesis, Structure, and Spectroscopic Properties of Copper(II) Compounds Containing Nitrogen–Sulphur Donor Ligands; the Crystal and Molecular Structure of Aqua[1,7-bis(*N*-methylbenzimi- dazol-2′-yl]-2,6-dithiaheptane]copper(II) Perchlorate. J. Chem. Soc., Dalton Trans. 1984, 1349–1356. 10.1039/DT9840001349.

[ref55] MarvinSketch; ChemAxon Ltd: Budapest, Hungary, 2012.

[ref56] Hager S.; Pape V. F. S.; Pósa V.; Montsch B.; Uhlik L.; Szakács G.; Tóth S.; Jabronka N.; Keppler B. K.; Kowol C. R.; Enyedy É. A.; Heffeter P. High Copper Complex Stability and Slow Reduction Kinetics as Key Parameters for Improved Activity, Paraptosis Induction, and Impact on Drug-Resistant Cells of Anticancer Thiosemicarbazones. Antioxid. Redox Signaling 2020, 33, 395–414. 10.1089/ars.2019.7854. 32336116

[ref57] Elmore S. Apoptosis: A Review of Programmed Cell Death. Toxicol. Pathol. 2007, 35, 495–516. 10.1080/01926230701320337. 17562483 PMC2117903

[ref58] Vogelstein B.; Lane D.; Levine A. J. Surfing the P53 Network. Nature 2000, 408, 307–310. 10.1038/35042675. 11099028

[ref59] Weller M. Predicting Response to Cancer Chemotherapy: The Role of P53. Cell Tissue Res. 1998, 292, 435–445. 10.1007/s004410051072. 9582400

[ref60] Bunz F.; Hwang P. M.; Torrance C.; Waldman T.; Zhang Y.; Dillehay L.; Williams J.; Lengauer C.; Kinzler K. W.; Vogelstein B. Disruption of P53 in Human Cancer Cells Alters the Responses to Therapeutic Agents. J. Clin. Invest. 1999, 104, 263–269. 10.1172/JCI6863. 10430607 PMC408422

[ref61] UniProtKB - P17612 (KAPCA_HUMAN) https://www.uniprot.org/uniprot/P17612 (accessed 2021-05-15).

[ref62] UniProtKB - Q14012 (KCC1A_HUMAN) https://www.uniprot.org/uniprot/Q14012 (accessed 2021-05-15).

[ref63] UniProtKB - P11309 (PIM1_HUMAN) https://www.uniprot.org/uniprot/P11309 (accessed 2021-05-15).

[ref64] UniProtKB - O00141 (SGK1_HUMAN) https://www.uniprot.org/uniprot/O00141 (accessed 2021-05-15).

[ref65] UniProtKB - P49841 (GSK3B_HUMAN) https://www.uniprot.org/uniprot/P49841 (accessed 2021-07-12).

[ref66] UniProtKB - O75582 (KS6A5_HUMAN) https://www.uniprot.org/uniprot/O75582 (accessed 2021-05-15).

[ref67] Sang Y.; Kong P.; Zhang S.; Zhang L.; Cao Y.; Duan X.; Sun T.; Tao Z.; Liu W. SGK1 in Human Cancer: Emerging Roles and Mechanisms. Front. Oncol. 2021, 10, 608722 10.3389/fonc.2020.608722. 33542904 PMC7851074

[ref68] Guerriero I.; Monaco G.; Coppola V.; Orlacchio A. Serum and Glucocorticoid-Inducible Kinase 1 (SGK1) in NSCLC Therapy. Pharmaceuticals 2020, 13, 413 10.3390/ph13110413. PMC7700219 33266470

[ref69] Lang F.; Perrotti N.; Stournaras C. Colorectal Carcinoma Cells – Regulation of Survival and Growth by SGK1. Int. J. Biochem. Cell Biol. 2010, 42, 1571–1575. 10.1016/j.biocel.2010.05.016. 20541034

[ref70] Talarico C.; Dattilo V.; D’Antona L.; Menniti M.; Bianco C.; Ortuso F.; Alcaro S.; Schenone S.; Perrotti N.; Amato R. SGK1, the New Player in the Game of Resistance: Chemo-Radio Molecular Target and Strategy for Inhibition. Cell. Physiol. Biochem. 2016, 39, 1863–1876. 10.1159/000447885. 27771704

[ref71] Catalogna G.; Talarico C.; Dattilo V.; Gangemi V.; Calabria F.; D’Antona L.; Schenone S.; Musumeci F.; Bianco C.; Perrotti N.; Amato R.; Cascini G. L. The SGK1 Kinase Inhibitor SI113 Sensitizes Theranostic Effects of the ^64^CuCl_2_ in Human Glioblastoma Multiforme Cells. Cell. Physiol. Biochem. 2017, 43, 108–119. 10.1159/000480328. 28848088

[ref72] Rinaldi L.; Delle Donne R.; Borzacchiello D.; Insabato L.; Feliciello A. The Role of Compartmentalized Signaling Pathways in the Control of Mitochondrial Activities in Cancer Cells. Biochim. Biophys. Acta, Rev. Cancer 2018, 1869, 293–302. 10.1016/j.bbcan.2018.04.004. 29673970

[ref73] Chiaradonna F.; Balestrieri C.; Gaglio D.; Vanoni M. RAS and PKA Pathways in Cancer: New Insight from Transcriptional Analysis. Front. Biosci. 2008, 13, 5257. 18508585 10.2741/3079

[ref74] Fu X.; Fan X.; Hu J.; Zou H.; Chen Z.; Liu Q.; Ni B.; Tan X.; Su Q.; Wang J.; Wang L.; Wang J. Overexpression of MSK1 Is Associated with Tumor Aggressiveness and Poor Prognosis in Colorectal Cancer. Dig. Liver Dis. 2017, 49, 683–691. 10.1016/j.dld.2017.02.009. 28314603

[ref75] Li J.; Liu X.; Wang W.; Li C.; Li X. MSK1 Promotes Cell Proliferation and Metastasis in Uveal Melanoma by Phosphorylating CREB. Arch. Med. Sci. 2020, 16, 1176–1188. 10.5114/aoms.2019.85810. 32864007 PMC7444723

[ref76] Lei Y.; Yu T.; Li C.; Li J.; Liang Y.; Wang X.; Chen Y.; Wang X. Expression of CAMK1 and Its Association with Clinicopathologic Characteristics in Pancreatic Cancer. J. Cell Mol. Med. 2021, 25, 1198–1206. 10.1111/jcmm.16188. 33342045 PMC7812292

[ref77] Rodriguez-Mora O.; LaHair M. M.; Howe C. J.; McCubrey J. A.; Franklin R. A. Calcium/Calmodulin-Dependent Protein Kinases as Potential Targets in Cancer Therapy. Expert Opin. Ther. Targets 2005, 9, 791–808. 10.1517/14728222.9.4.791. 16083343

[ref78] Tsuyoshi H.; Orisaka M.; Fujita Y.; Asare-Werehene M.; Tsang B. K.; Yoshida Y. Prognostic Impact of Dynamin Related Protein 1 (Drp1) in Epithelial Ovarian Cancer. BMC Cancer 2020, 20, 467 10.1186/s12885-020-06965-4. 32448194 PMC7247242

[ref79] Li Q.; Chen L.; Luo C.; ChenYan; Ge J.; Zhu Z.; Wang K.; Yu X.; Lei J.; Liu T.; Peng X.; Liu X.; Yuan R. TAB3 Upregulates PIM1 Expression by Directly Activating the TAK1-STAT3 Complex to Promote Colorectal Cancer Growth. Exp. Cell Res. 2020, 391, 111975 10.1016/j.yexcr.2020.111975. 32229191

[ref80] Herzog S.; Fink M. A.; Weitmann K.; Friedel C.; Hadlich S.; Langner S.; Kindermann K.; Holm T.; Böhm A.; Eskilsson E.; Miletic H.; Hildner M.; Fritsch M.; Vogelgesang S.; Havemann C.; Ritter C. A.; Meyerzu Schwabedissen H. E.; Rauch B.; Hoffmann W.; Kroemer H. K.; Schroeder H.; Bien-Möller S. Pim1 Kinase Is Upregulated in Glioblastoma Multiforme and Mediates Tumor Cell Survival. Neuro-Oncol. 2015, 17, 223–242. 10.1093/neuonc/nou216. 25155357 PMC4288523

[ref81] Zhang C.; Qie Y.; Yang T.; Wang L.; Du E.; Liu Y.; Xu Y.; Qiao B.; Zhang Z. Kinase PIM1 Promotes Prostate Cancer Cell Growth via C-Myc-RPS7-Driven Ribosomal Stress. Carcinogenesis 2019, 40, 52–60. 10.1093/carcin/bgy126. 30247545

[ref82] Kim S.; Kim W.; Kim D.-H.; Jang J.-H.; Kim S.-J.; Park S.-A.; Hahn H.; Han B. W.; Na H.-K.; Chun K.-S.; Choi B. Y.; Surh Y.-J. Resveratrol Suppresses Gastric Cancer Cell Proliferation and Survival through Inhibition of PIM-1 Kinase Activity. Arch. Biochem. Biophys. 2020, 689, 108413 10.1016/j.abb.2020.108413. 32473133

[ref83] Stukenbrock H.; Mussmann R.; Geese M.; Ferandin Y.; Lozach O.; Lemcke T.; Kegel S.; Lomow A.; Burk U.; Dohrmann C.; Meijer L.; Austen M.; Kunick C. 9-Cyano-1-azapaullone (Cazpaullone), a Glycogen Synthase Kinase-3 (GSK-3) Inhibitor Activating Pancreatic β Cell Protection and Replication. J. Med. Chem. 2008, 51, 2196–2207. 10.1021/jm701582f. 18345612

[ref84] Kunick C.; Lauenroth K.; Wieking K.; Xie X.; Schultz C.; Gussio R.; Zaharevitz D.; Leost M.; Meijer L.; Weber A.; Jørgensen F. S.; Lemcke T. Evaluation and Comparison of 3D-QSAR CoMSIA Models for CDK1, CDK5, and GSK-3 Inhibition by Paullones. J. Med. Chem. 2004, 47, 22–36. 10.1021/jm0308904. 14695817

[ref85] Kumar A.; Mandiyan V.; Suzuki Y.; Zhang C.; Rice J.; Tsai J.; Artis D. R.; Ibrahim P.; Bremer R. Crystal Structures of Proto-Oncogene Kinase Pim1: A Target of Aberrant Somatic Hypermutations in Diffuse Large Cell Lymphoma. J. Mol. Biol. 2005, 348, 183–193. 10.1016/j.jmb.2005.02.039. 15808862

[ref86] Debreczeni J. E.; Bullock A.; Keates T.; Niesen F. H.; Salah E.; Shrestha L.; Smee C.; Sobott F.; Pike A. C. W.; Bunkoczi G.; von Delft F.; Turnbull A.; Weigelt J.; Arrowsmith C. H.; Edwards A.; Sundstrom M.; Knapp S. Crystal Structure of Human Calmodulin-Dependent Protein Kinase I G. PDB entry 2JC6.

[ref87] Aronov A. M.; Tang Q.; Martinez-Botella G.; Bemis G. W.; Cao J.; Chen G.; Ewing N. P.; Ford P. J.; Germann U. A.; Green J.; Hale M. R.; Jacobs M.; Janetka J. W.; Maltais F.; Markland W.; Namchuk M. N.; Nanthakumar S.; Poondru S.; Straub J.; ter Haar E.; Xie X. Structure-Guided Design of Potent and Selective Pyrimidylpyrrole Inhibitors of Extracellular Signal-Regulated Kinase (ERK) Using Conformational Control. J. Med. Chem. 2009, 52, 6362–6368. 10.1021/jm900630q. 19827834

[ref88] Rinaldi J.; Wu J.; Yang J.; Ralston C. Y.; Sankaran B.; Moreno S.; Taylor S. S. Structure of Yeast Regulatory Subunit: A Glimpse into the Evolution of PKA Signaling. Structure 2010, 18, 1471–1482. 10.1016/j.str.2010.08.013. 21070946 PMC3435106

[ref89] Hammond M.; Washburn D. G.; Hoang T. H.; Manns S.; Frazee J. S.; Nakamura H.; Patterson J. R.; Trizna W.; Wu C.; Azzarano L. M.; Nagilla R.; Nord M.; Trejo R.; Head M. S.; Zhao B.; Smallwood A. M.; Hightower K.; Laping N. J.; Schnackenberg C. G.; Thompson S. K. Design and Synthesis of Orally Bioavailable Serum and Glucocorticoid-Regulated Kinase 1 (SGK1) Inhibitors. Bioorg. Med. Chem. Lett. 2009, 19, 4441–4445. 10.1016/j.bmcl.2009.05.051. 19497745

[ref90] Jones G.; Willett P.; Glen R. C.; Leach A. R.; Taylor R. Development and Validation of a Genetic Algorithm for Flexible Docking. J. Mol. Biol. 1997, 267, 727–748. 10.1006/jmbi.1996.0897. 9126849

[ref91] Verdonk M. L.; Cole J. C.; Hartshorn M. J.; Murray C. W.; Taylor R. D. Improved Protein-Ligand Docking Using GOLD. Proteins 2003, 52, 609–623. 10.1002/prot.10465. 12910460

[ref92] Eldridge M. D.; Murray C. W.; Auton T. R.; Paolini G. V.; Mee R. P. Empirical Scoring Functions: I. The Development of a Fast Empirical Scoring Function to Estimate the Binding Affinity of Ligands in Receptor Complexes. J. Comput.-Aided Mol. Des. 1997, 11, 425–445. 9385547 10.1023/a:1007996124545

[ref93] Korb O.; Stützle T.; Exner T. E. Empirical Scoring Functions for Advanced Protein–Ligand Docking with PLANTS. J. Chem. Inf. Model. 2009, 49, 84–96. 10.1021/ci800298z. 19125657

[ref94] Mooij W. T. M.; Verdonk M. L. General and Targeted Statistical Potentials for Protein-Ligand Interactions. Proteins 2005, 61, 272–287. 10.1002/prot.20588. 16106379

[ref95] QikProp; 2009.

[ref96] Scigress Ultra V F.J 2.6.

[ref97] Zhu F.; Logan G.; Reynisson J. Wine Compounds as a Source for HTS Screening Collections. A Feasibility Study. Mol. Inf. 2012, 31, 847–855. 10.1002/minf.201200103. 27476738

[ref98] Eurtivong C.; Reynisson J. The Development of a Weighted Index to Optimise Compound Libraries for High Throughput Screening. Mol. Inf. 2019, 38, 1800068 10.1002/minf.201800068. 30345657

[ref99] Matuszek A. M.; Reynisson J. Defining Known Drug Space Using DFT. Mol. Inf. 2016, 35, 46–53. 10.1002/minf.201500105. 27491789

[ref100] Yu B.; Reynisson J. Bond Stability of the “Undesirable” Heteroatom–Heteroatom Molecular Moieties for High-Throughput Screening Libraries. Eur. J. Med. Chem. 2011, 46, 5833–5837. 10.1016/j.ejmech.2011.09.044. 22000918

[ref101] Sheldrick G. M. A Short History of SHELX. Acta Crystallogr., Sect. A: Found. Crystallogr. 2008, 64, 112–122. 10.1107/S0108767307043930. 18156677

[ref102] Irving H. M.; Miles M. G.; Pettit L. D. A Study of Some Problems in Determining the Stoichiometric Proton Dissociation Constrants of Complexes by Potentiometric Titrations Using a Glass Electrode. Anal. Chim. Acta 1967, 38, 475–488. 10.1016/S0003-2670(01)80616-4.

[ref103] Enyedy É. A.; Nagy N. V.; Zsigó É.; Kowol C. R.; Arion V. B.; Keppler B. K.; Kiss T. Comparative Solution Equilibrium Study of the Interactions of Copper(II), Iron(II) and Zinc(II) with Triapine (3-Aminopyridine-2-carbaldehyde Thiosemicarbazone) and Related Ligands. Eur. J. Inorg. Chem. 2010, 1717–1728. 10.1002/ejic.200901174.

[ref104] Zekany L.; Nagypal I. PSEQUAD A Comprehensive Program for the Evaluation of Potentiometric and or Spectrophotometric Equilibrium Data Using Analytical Derivatives; Plenum Press: New York, 1985.

[ref105] Dömötör O.; de Almeida R. F. M.; Côrte-Real L.; Matos C. P.; Marques F.; Matos A.; Real C.; Kiss T.; Enyedy É. A.; Helena Garcia M.; Tomaz A. I. Studies on the Mechanism of Action of Antitumor Bis(Aminophenolate) Ruthenium(III) Complexes. J. Inorg. Biochem. 2017, 168, 27–37. 10.1016/j.jinorgbio.2016.12.008. 28006663

[ref106] Dömötör O.; Enyedy É. A. Binding Mechanisms of Half-Sandwich Rh(III) and Ru(II) Arene Complexes on Human Serum Albumin: A Comparative Study. J. Biol. Inorg. Chem. 2019, 24, 703–719. 10.1007/s00775-019-01683-0. 31300922 PMC6682546

[ref107] Lakowicz J. R. Principles of Fluorescence Spectroscopy, 3rd ed. (corr. at 4. print.).; Springer: New York, 2010.

[ref108] Mucsi I.; Varga A.; Kawase M.; Motohashi N.; Molnar J. Interaction between Various Resistance Modifiers and Apoptosis Inducer 12*H*-Benzo[alpha]phenothiazine. Anticancer Res. 2002, 22, 2833–2836. 12530005

[ref109] Bain J.; Plater L.; Elliott M.; Shpiro N.; Hastie C. J.; Mclauchlan H.; Klevernic I.; Arthur J. S. C.; Alessi D. R.; Cohen P. The Selectivity of Protein Kinase Inhibitors: A Further Update. Biochem. J. 2007, 408, 297–315. 10.1042/BJ20070797. 17850214 PMC2267365

[ref110] Hastie C. J.; McLauchlan H. J.; Cohen P. Assay of Protein Kinases Using Radiolabeled ATP: A Protocol. Nat. Protoc. 2006, 1, 968–971. 10.1038/nprot.2006.149. 17406331

[ref111] Berman H.; Henrick K.; Nakamura H. Announcing the Worldwide Protein Data Bank. Nat. Struct. Mol. Biol. 2003, 10, 980–980. 10.1038/nsb1203-980. 14634627

[ref112] Berman H. M.; Westbrook J.; Feng Z.; Gilliland G.; Bhat T. N.; Weissig H.; Shindyalov I. N.; Bourne P. E. The Protein Data Bank. Nucleic Acids Res. 2000, 28, 235–242. 10.1093/nar/28.1.235. 10592235 PMC102472

[ref113] Wang Z.; Sun H.; Yao X.; Li D.; Xu L.; Li Y.; Tian S.; Hou T. Comprehensive Evaluation of Ten Docking Programs on a Diverse Set of Protein–Ligand Complexes: The Prediction Accuracy of Sampling Power and Scoring Power. Phys. Chem. Chem. Phys. 2016, 18, 12964–12975. 10.1039/C6CP01555G. 27108770

[ref114] Bissantz C.; Folkers G.; Rognan D. Protein-Based Virtual Screening of Chemical Databases. 1. Evaluation of Different Docking/Scoring Combinations. J. Med. Chem. 2000, 43, 4759–4767. 10.1021/jm001044l. 11123984

[ref115] Allinger N. L.; Yuh Y. H.; Lii J. H. Molecular Mechanics. The MM3 Force Field for Hydrocarbons. 1. J. Am. Chem. Soc. 1989, 111, 8551–8566. 10.1021/ja00205a001.

[ref116] Lii J. H.; Allinger N. L. Molecular Mechanics. The MM3 Force Field for Hydrocarbons. 2. Vibrational Frequencies and Thermodynamics. J. Am. Chem. Soc. 1989, 111, 8566–8575. 10.1021/ja00205a002.

[ref117] Lii J. H.; Allinger N. L. Molecular Mechanics. The MM3 Force Field for Hydrocarbons. 3. The van Der Waals’ Potentials and Crystal Data for Aliphatic and Aromatic Hydrocarbons. J. Am. Chem. Soc. 1989, 111, 8576–8582. 10.1021/ja00205a003.

[ref118] Goto̅ H.; O̅sawa E. An Efficient Algorithm for Searching Low-Energy Conformers of Cyclic and Acyclic Molecules. J. Chem. Soc., Perkin Trans. 2 1993, 187–198. 10.1039/P29930000187.

