# Supplementary material for: Highly Antiproliferative Latonduine and Indolo[2,3-c]quinoline Derivatives: Complex Formation with Copper(II) Markedly Changes the Kinase Inhibitory Profile
Source: J Med Chem. 2022 Feb 1;65(3):2238–61. doi: 10.1021/acs.jmedchem.1c01740 (PMC8842277; doi:10.1021/acs.jmedchem.1c01740)
Supplement: Supplementary file 1 — jm1c01740_si_001.pdf [file jm1c01740_si_001.pdf]

Supporting Information

for

Highly Antiproliferative Latonduine and Indolo[2,3-c]quinoline Derivatives: Complex Formation with Copper(II) Markedly Changes the Kinase Inhibitory Profile

Christopher Wittmann,[†] Felix Bacher,[†] Eva A. Enyedy,[‡,§] Orsolya Dömötör,[‡,§] Gabriella Spengler,[§,Δ] Christian Madejski,[†] Jóhannes Reynisson,[⊥] and Vladimir B. Arion*[,†]

[†]Institute of Inorganic Chemistry of the University of Vienna, Währinger Strasse 42, A1090 Vienna, Austria

[‡]Department of Inorganic and Analytical Chemistry, Interdisciplinary Excellence Centre, University of Szeged, Dóm tér 7, H-6720 Szeged, Hungary

[§]MTA-SZTE Lendület Functional Metal Complexes Research Group, University of Szeged, Dóm tér 7, H-6720 Szeged, Hungary

[Δ]Department of Medical Microbiology, Albert Szent-Györgyi Health Center and Albert Szent-Györgyi Medical School, University of Szeged, Semmelweis u. 6, H-6725 Szeged, Hungary

[⊥]School of Pharmacy and Bioengineering, Keele University, Hornbeam Building, Staffordshire, ST5 5BG, United Kingdom

Table of Contents

- Numbering Schemes S3
- Previously reported Paullones S3
- Additional X-ray crystallography results S4
- Crystallographic data collection S6
- UV–vis and fluorescence data S7
- Apoptosis and DNA binding studies S9
- Enzyme inhibition assays S11
- IC_{50} determination for **HL^8^** and **8** against 6 different kinases S12
- Molecular docking with PIM-1, PKA and SGK-1 .... S16
- Other molecular docking details S19
- Yields and analytical data of isolated proligands and Cu(II) complexes S24
- NMR spectra S28
  - ^{1}H NMR spectra S28
  - ^{13}C NMR spectra S37
- ESI mass spectra S42
- HPLC-HR-mass spectra S69
- References S76

- **Numbering Schemes**

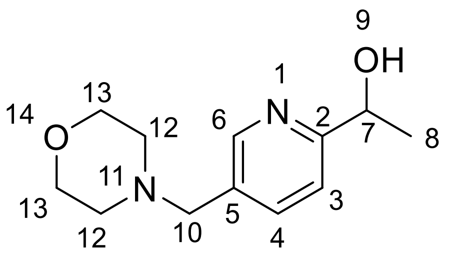

I

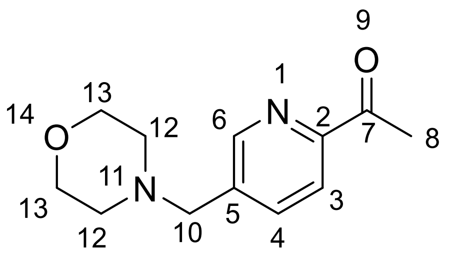

J

Chart S1. Numbering scheme of 1-(5-(morpholinomethyl)pyridin-2-yl)ethan-1-ol (**I**) and 2-acetyl-5-(morpholinomethyl)pyridine (**J**).

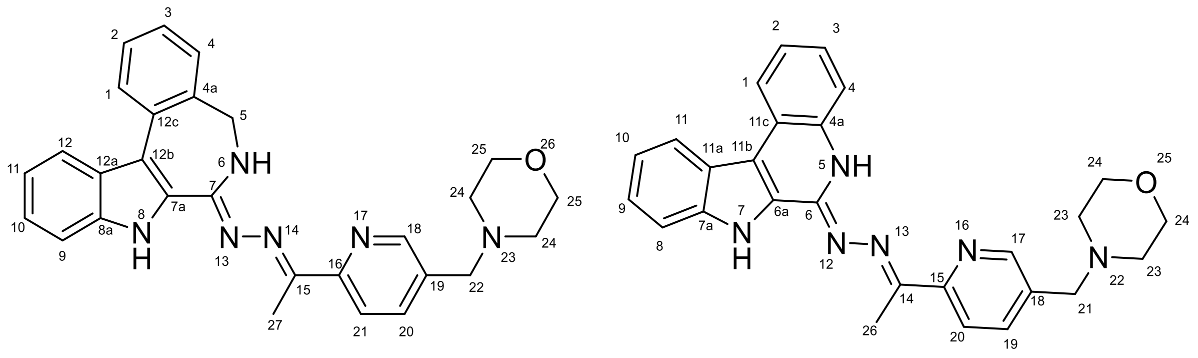

Chart S2. Numbering scheme of the novel morpholine-bearing latonduine- and indolo[2,3-c]quinoline derivatives.

- **Previously reported Paullones**

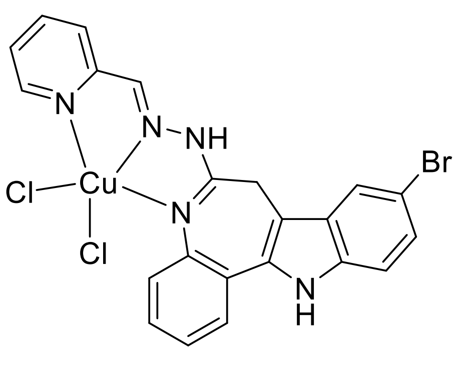

[Cu(H^{P}L^3)Cl$_2$]

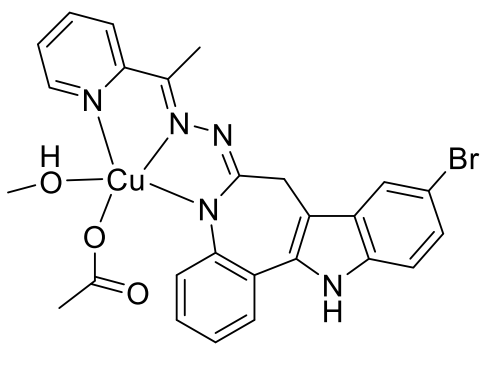

[Cu(^{P}L^4)(CH$_3$COO)(CH$_3$OH)]

Chart S3. Related copper(II) complexes. Superscript ^{P} in the chemical formula of the ligand indicates that the latter is derived from a Paullone core.

- **Additional X-ray crystallography results**

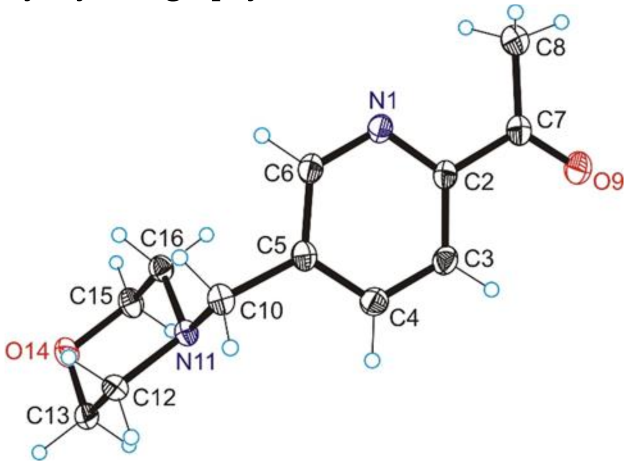

Figure S1. ORTEP plot of the ketone **J** with thermal elipsoids at 50% probability level and atom labeling scheme.

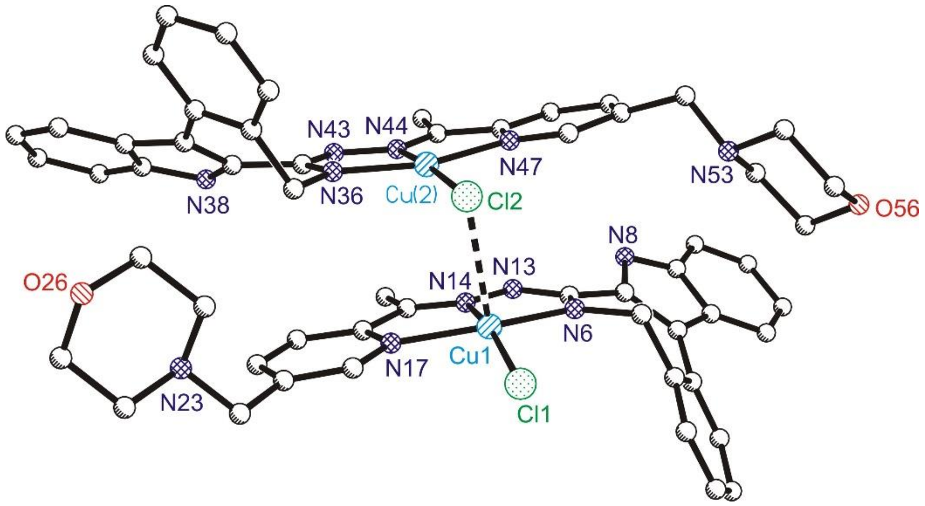

Figure S2. Association of molecules of **3** into dimer.

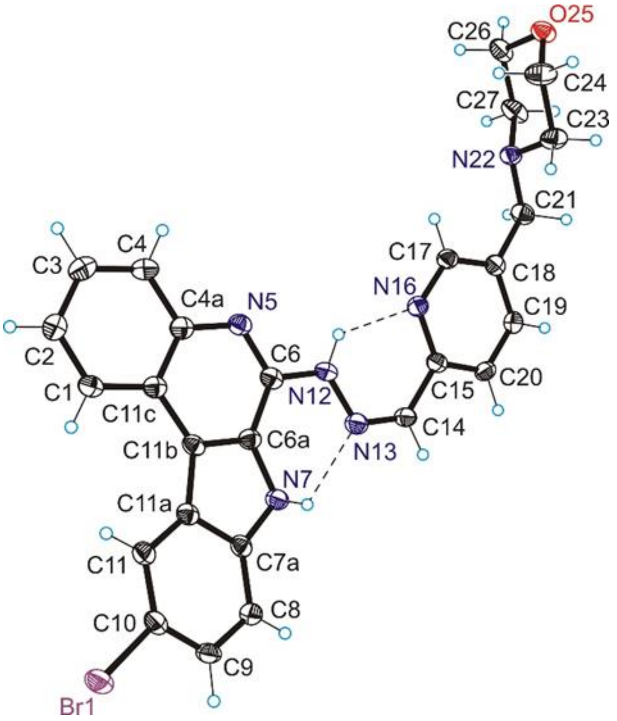

Figure S3. ORTEP view of the ligand **HL[6]** with thermal ellipsoids at 50% probability level. Selected bond distances (Å), bond angles (deg) and torsion angles (deg): N5–C6 1.320(3), C6–N12 1.374(3), N12–N13 1.361(3), N13–C14 1.290(3), C14–C15 1.459(3), C15–N16 1.349(3), C6–C6a 1.422(3), C6a–N7 1.383(3), $\Theta_{C4a–N5–C6–C6a}$ –1.9(3), $\Theta_{N13–C14–C15–N16}$ 2.1(3).

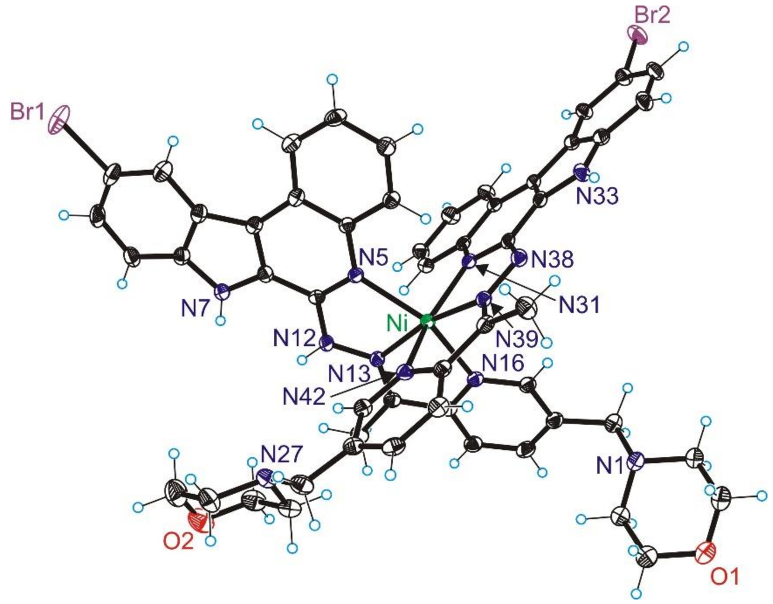

Figure S4. ORTEP view of the complex cation in **[Ni(L^8)(HL^8)]Cl·2DMF**.

- **Crystallographic data collection**

Table S1. Crystal Data and Details of Data Collection and Refinement for **[CuCl(L[1])(DMF)]·DMF** (**1·DMF**) and **[CuCl(L[2])(MeOH)] (2), [CuCl$_2$(H$_2$L[5])]Cl·2DMF** (**[5+H]Cl·2DMF**), **HL[6]** and **[Ni(L[8])(HL[8])]Cl·2DMF**.

compound	ketone **J**	**1·DMF**	**2**	**3·0.5H$_2$O**	**[5+H]Cl·2DMF**	**HL[6]**	**[Ni(L[8])(HL[8])]Cl·2DMF**
emp. formula	$C_{12}H_{16}N_2O_2$	$C_{33}H_{39}ClCuN_8O_3$	$C_{28}H_{28}BrClCuN_6O_2$	$C_{28}H_{28}ClCuN_6O_{1.5}$	$C_{32}H_{39}Cl_3CuN_8O_3$	$C_{26}H_{23}BrN_6O$	$C_{60}H_{63}Br_2ClN_{14}NiO_4$
fw	220.27	694.71	659.46	571.55	753.60	515.41	1298.22
space group	*P*-1	*C*2/*c*	*P*2$_1$/*c*	*P*2$_1$/*c*	*P*1	*P*-1	*P*-1
a, Å	7.7366(8)	33.667(6)	16.1127(6)	15.0147(7)	7.2313(10)	12.220(2)	9.7632(13)
b, Å	7.9072(8)	10.5159(16)	8.9758(3)	34.2629(15)	9.2604(13)	12.7328(19)	15.6735(15)
c, Å	10.7395(11)	19.220(3)	19.2463(7)	10.2999(4)	14.1201(19)	16.4240(15)	19.622(2)
α, °	99.872(4)				78.160(6)	76.485(5)	87.172(3)
β, °	102.594(4)	118.948(4)	103.8245(13)	107.565(3)	77.214(6)	82.598(5)	75.925(5)
γ, °	113.640(4)				70.423(6)	67.698(8)	85.319(4)
V [Å^3]	562.21(10)	6662.0(18)	2702.85(17)	5051.7(4)	859.9(2)	2296.6(6)	2901.4(6)
Z	2	8	4	8	1	4	2
λ [Å]	0.71073	0.71073	0.71073	0.71073	0.71073	0.71073	0.71073
ρ_{calcd}, g cm^{-3}	1.301	1.385	1.621	1.503	1.455	1.491	1.486
cryst size, mm^3	0.21 × 0.05× 0.02	0.15 × 0.15 × 0.08	0.18 × 0.13 × 0.05	0.29 × 0.09 × 0.01	0.05 × 0.02 × 0.01	0.10 × 0.02 × 0.01	0.12 × 0.04 × 0.01
T [K]	100(2)	200(2)	100(2)	125(2)	100(2)	100(2)	100(2)
μ, mm^{-1}	0.090	0.782	2.424	1.008	0.914	1.822	1.819
R_1^{a}	0.0436	0.0457	0.0287	0.0472	0.0753	0.0373	0.0331
wR_2^{b}	0.1232	0.1086	0.0709	0.1203	0.1779	0.0928	0.0833
GOF^c	1.083	1.011	1.016	1.019	1.057	1.043	1.022

^{a} $R_1 = \Sigma||F_o| - |F_c||/\Sigma|F_o|$. ^{b} $wR_2 = \{\Sigma[w(F_o^2 - F_c^2)^2]/\Sigma[w(F_o^2)^2]\}^{1/2}$. ^{c} GOF = $\{\Sigma[w(F_o^2 - F_c^2)^2]/(n - p)\}^{1/2}$, where n is the number of reflections and p is the total number of parameters refined.

- **UV–vis and fluorescence data**

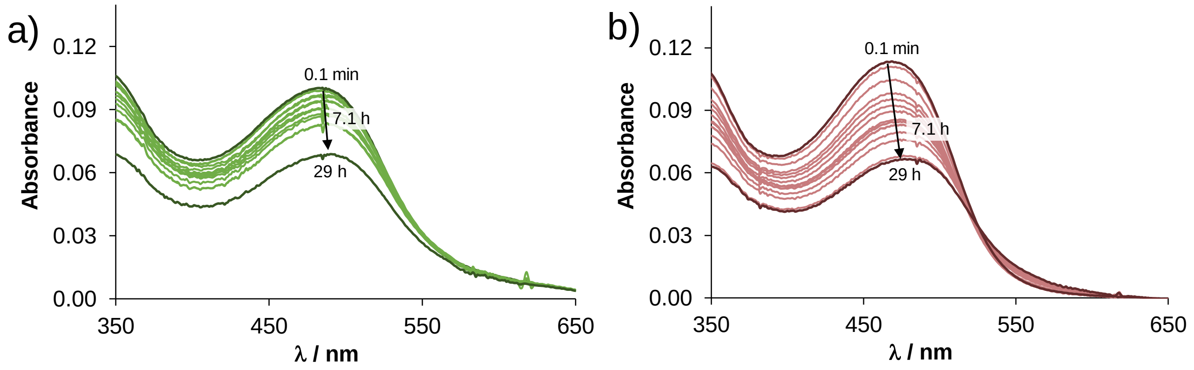

Figure S5. a) UV–vis absorbance spectrum of **4** followed in PBS, and b) in HEPES buffer. {$c_{complex}$ = 6.3 μM, l = 1 cm; pH = 7.40, PBS (a), 10 mM HEPES (b)}.

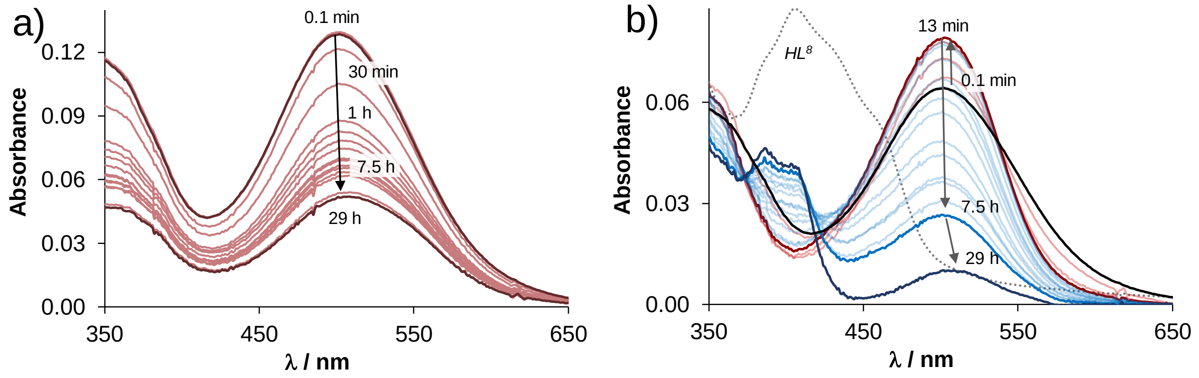

Figure S6. a) UV–vis absorbance spectrum of **8** followed in HEPES buffer and b) in diluted blood serum; spectrum of metal-free ligand **HL^8** is shown as well in Figure 8b). {$c_{complex}$ = 6.1 μM, c_{ligand} = 6.1 μM; l = 1 cm (a), 0.5 cm (b); pH = 7.40, 10 mM HEPES (a), diluted by factor 3 blood serum in 10 mM HEPES (b)}.

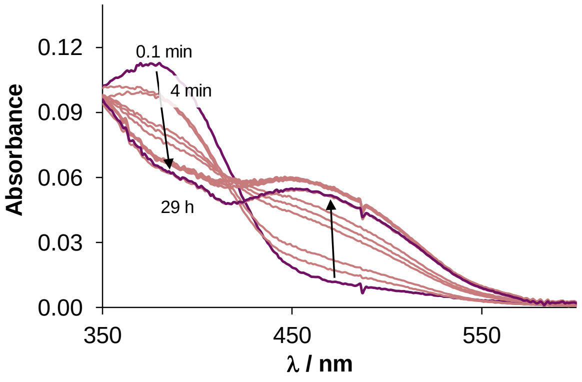

Figure S7. UV–vis absorbance spectrum of **HL^4** followed in diluted serum. {c_{ligand} = 6.3 μM, l = 0.5 cm; pH = 7.40, three-times diluted blood serum in 10 mM HEPES}.

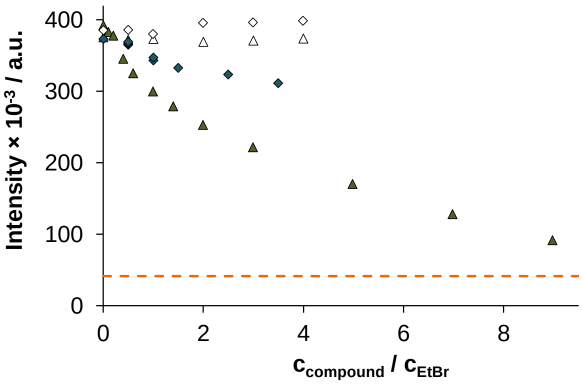

Figure S8. Fluorescence intensity values of the ct-DNA–EtBr system in the presence of increasing amount of **HL⁴** (◊), **HL⁸** (Δ), **4** (♦) and **8** (▲), orange dashed line denotes the emission signal of free EtBr. At the indicated ratios precipitate formation was not observed {c_{DNA} = 10.0 µM, c_{EtBr} = 5.0 µM; λ_{EX} = 510 nm, λ_{EM} = 610 nm; T = 298 K; 10 mM HEPES, pH = 7.40}.

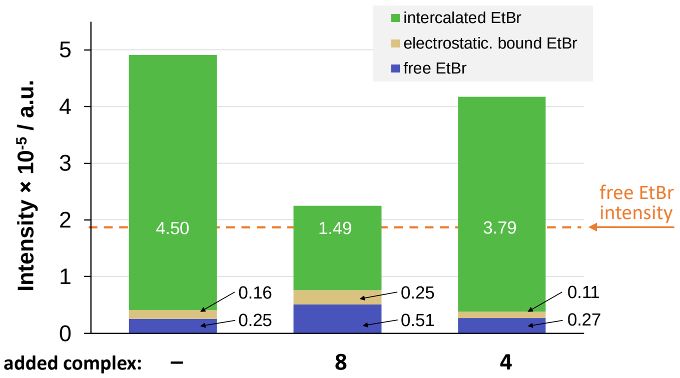

τ_1 / ns	1.65	1.65	1.65
τ_2 / ns	6.4 ± 0.5	6.6 ± 0.3	5.8 ± 0.6
τ_3 / ns	21.27 ± 0.04	18.83 ± 0.06	21.21 ± 0.04

Figure S9. Deconvoluted fluorescence intensities and lifetime values (τ) of the ct-DNA–EtBr–indicated complex systems at 1:5:12.5 ratio based on steady-state and fluorescence lifetime measurements. τ_3 can be attributed to the intercalated EtBr; in the presence of **8** it was decreased significantly, this indicates changes in the close environment of the intercalated form. τ_2 denotes most probably EtBr bound electrostatically at the phosphate backbone of DNA, its relatively large uncertainty is due to the small quantity of this form at these conditions. τ_1 was kept constant in the calculations. {c_{DNA} = 10.0 µM; λ_{EX} = 455 nm, λ_{EM} = 610 nm; T = 298 K; 10 mM HEPES, pH = 7.40}.

- **Apoptosis and DNA binding studies**

Table S2. Gated events (%) in Colo320 colon adenocarcinoma cells measured by flow cytometry with 3 h induction time using M627 and cisplatin as positive control.

**Colo 320**		**Gated events %**		
A+: annexin positive<br>I+: propidium iodide positive	Concentration	**Early apoptosis % Q3**	**Late apoptosis and necrosis % Q2**	**Cell death % Q1**
Control A-I+	-	0	0	0.074
Control A+I-	-	0.032	0.010	5.24
Control A+I+	-	3.70	5.34	3.44
Control A+I+	-	3.25	9.99	6.93
DMSO	0.8%	3.26	3.05	1.35
M627	10 µM	12.4	31.5	4.59
M627	20 µM	11.1	26.1	7.85
cisplatin	15 µM	1.68	2.24	1.64
cisplatin	30 µM	4.90	4.07	0.79
**HL^4**	2 µM	10.7	20.1	1.56
**HL^4**	4 µM	5.38	9.06	3.53
**4**	0.25 µM	7.48	12.7	5.10
**4**	0.5 µM	5.18	11.6	4.76
**HL^8**	2 µM	15.8	44.5	4.28
**HL^8**	4 µM	6	6.55	3.22
**8**	0.5 µM	6.01	12.1	4.52
**8**	2 µM	6.91	6.97	1.76
Control A+I+	-	4.73	7.18	2.89

Table S3. Instrument parameters for TCSPC measurements.

LED source λ_{EX} (nm)	455
λ_{EM} (nm)	610
Slit width on emission side (nm)	7
Count no. at peak channel	10 000
Approx. no. of channels used for analysis [a]	2000
Time window	100 ns (EtBr only)
	200 ns
Time calibration (ns/ch)	0.02532 (100 ns)
	0.05065 (200 ns)
Background correction	not needed
Instrument response function	Ludox®

[a] total number of channels = 4096

- **Enzyme inhibition assays**

Table S4. Enzyme inhibition data for **HL[8]** and **8** in a panel of 50 cancer related proteins

	**HL[8]**		**8**			**HL[8]**		**8**	
**c (μM)**	10	SD	10	SD	**c (μM)**	10	SD	10	SD
**MKK1**	101	7	94	6	**CK2**	75	2	79	1
**JNK1**	137	19	134	1	**DYRK1A**	106	3	77	1
**p38a MAPK**	138	3	123	18	**NEK6**	110	1	43	4
**RSK1**	96	7	53	8	**TBK1**	80	3	76	3
**PDK1**	135	5	121	18	**PIM1**	15	9	72	4
**PKBa**	116	16	82	2	**SRPK1**	82	5	40	1
**SGK-1**	99	9	5	4	**EF2K**	92	9	108	13
**S6K1**	111	2	109	12	**HIPK2**	91	21	108	6
**PKA**	87	9	10	2	**PAK4**	88	5	144	19
**ROCK 2**	104	3	98	7	**MST2**	62	6	82	4
**PRK2**	100	4	38	3	**MLK3**	72	7	41	12
**PKCa**	102	11	80	13	**TAK1**	99	15	120	1
**PKD1**	166	5	65	2	**IRAK4**	106	9	105	8
**MSK1**	71	15	17	6	**RIPK2**	95	2	95	19
**CAMKKb**	93	5	98	27	**TTK**	73	5	64	5
**CaMK-1**	81	9	9	3	**Src**	130	17	125	24
**SmMLCK**	109	3	87	16	**Lck**	89	9	54	5
**CHK2**	100	14	119	9	**BTK**	99	43	61	6
**GSK3β**	65	9	32	1	**JAK3**	108	14	120	21
**PLK1**	105	17	90	8	**SYK**	66	9	39	2
**Aurora B**	71	1	78	8	**EPH-A2**	156	28	132	28
**LKB1**	102	4	107	14	**HER4**	117	3	71	8
**AMPK (hum)**	103	20	91	8	**IGF-1R**	81	10	60	17
**MARK3**	99	10	112	12	**TrkA**	70	8	82	2
**CK1δ**	106	15	98	13	**VEG-FR**	100	10	77	16

- **IC_{50} determination for HL^8 and 8 against 6 different kinases**

Table S5. Summary IC_{50} values of **8** and **HL^8** for selected cancer related enzymes.

	**c(8) [μM]**	**c(HL^8) [μM]**
**SGK-1**	8.45	
**PKA**	6.69	
**CaMK-1**	0.75	
**GSK3β**	1.64	
**MSK1**	2.97	
**PIM1**		1.18

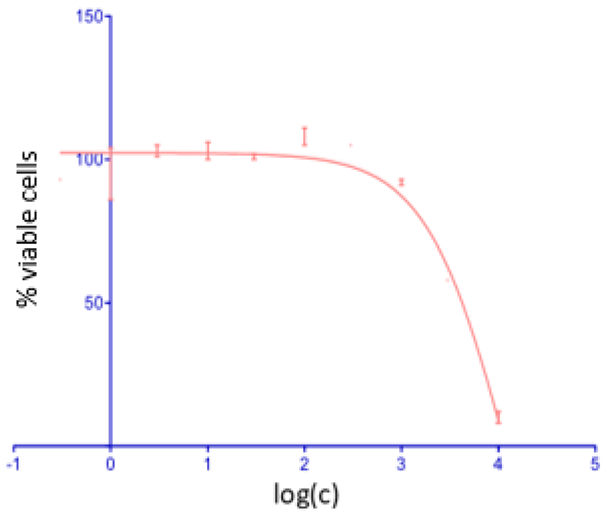

Figure S10. Determination of IC_{50} value of **8** against SGK-1.

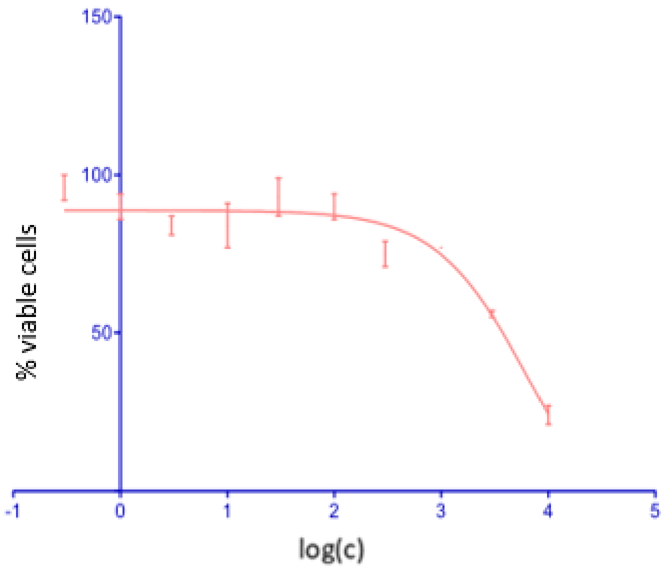

Figure S11. Determination of IC_{50} value of **8** against PKA.

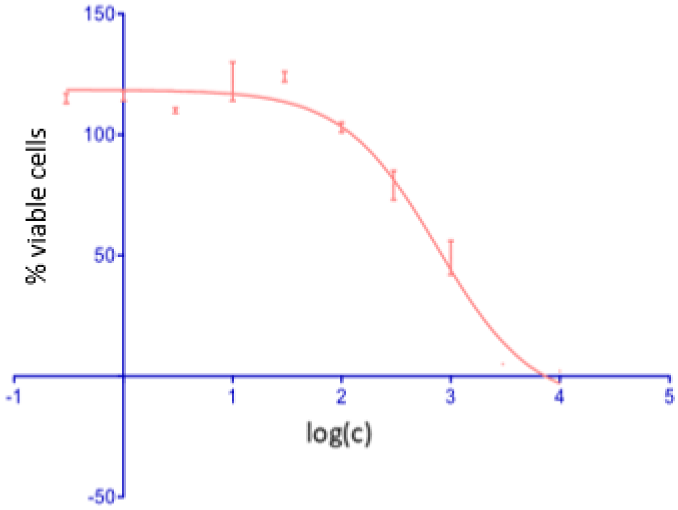

Figure S12. Determination of IC_{50} value of **8** against CaMK-1.

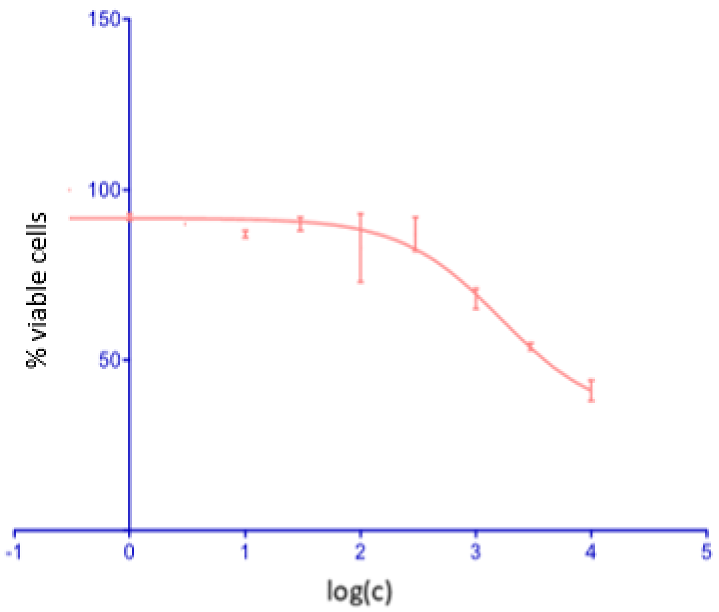

Figure S13. Determination of IC_{50} value of **8** against GSK3β.

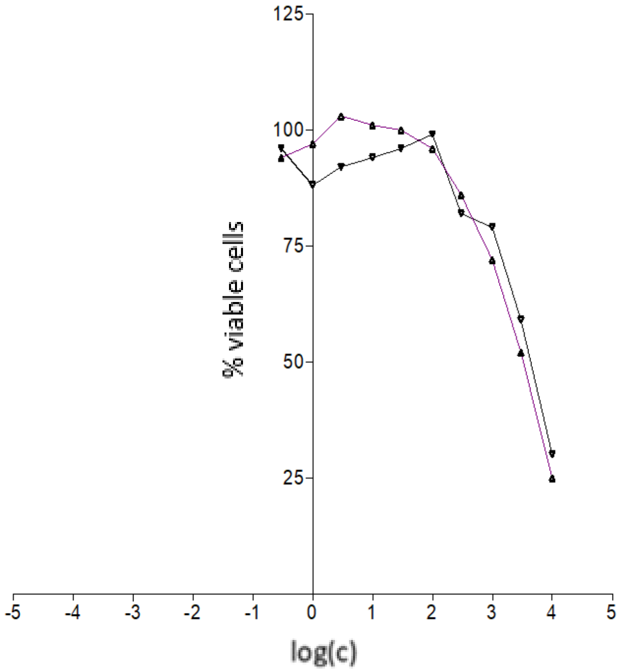

Figure S14. Determination of IC_{50} value of **8** against MSK1

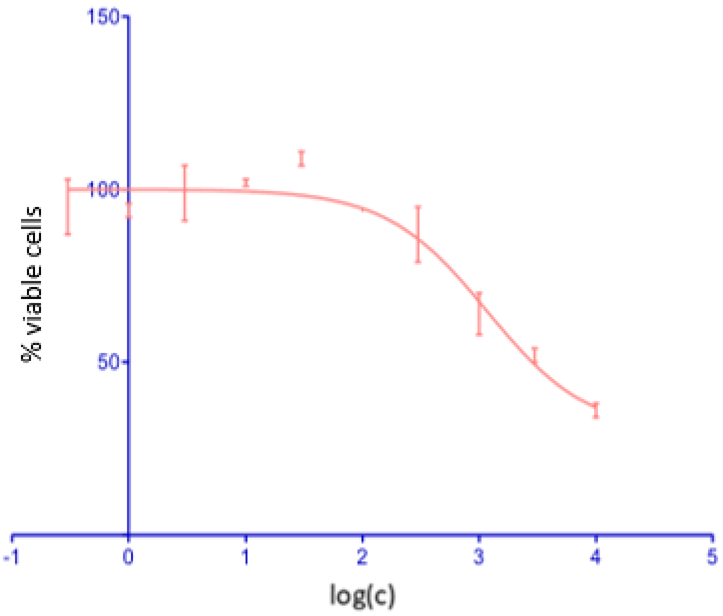

Figure S15. Determination of IC_{50} value of $\mathbf{HL^8}$ against PIM-1.

- **Molecular docking with PIM-1, PKA and SGK-1**

The QikProp v6.2[1] software package was used to calculate the molecular descriptors of the ligands. The reliability of it QikProp established for the calculated descriptors.[2] Furthermore, the Scigress version FJ 2.6 program[3] was used to calculate the molecular descriptors for the complexes. The Known Drug Indexes (KDI) were calculated from the molecular descriptors as described by Eurtivong and Reynisson.[4] For application in Excel, columns for each property were created and the following equations used do derive the KDI numbers for each descriptor: KDI MW: =EXP(-((*MW*-371.76) ^2)/(2×(112.76^2))), KDI log*P*: =EXP(-((*logP*-2.82) ^2)/(2×(2.21^2))), KDI HD: =EXP(-((*HD*-1.88) ^2)/(2×(1.7^2))), KDI HA: =EXP(-((*HA*-5.72) ^2)/(2×(2.86^2))), KDI RB =EXP(-((*RB*-4.44) ^2)/(2×(3.55^2))), and KDI PSA: =EXP(-((*PSA*-79.4)^2)/(2*(54.16^2))). These equations could simply be copied into Excel and the descriptor name (e.g., *molecular weight (MW)*) substituted with the value in the relevant column. To derive KDI_{2A}, this equation was used: = (KDI MW + KDI log*P* + KDI HD + KDI HA + KDI RB + KDI PSA) and for KDI_{2B}: = (KDI MW × KDI log*P* × KDI HD × KDI HA × KDI RB × KDI PSA). The Gaussian 16 software suite[5] was used with unrestricted Density Functional Theory (DFT). The B3LYP functional hybrid approach was employed[6–8] and standard 6-31+G(d,p) diffused basis set[9,10] was used for geometry optimization and frequency analysis (keywords: opt freq). The zero-point vibrational energies (ZPE) were scaled according to Wong (0.9804).[11] In all cases, normal modes revealed no imaginary frequencies indicating that they represent minima on the potential energy surface. The subsequent energy calculations were then performed with the larger 6-311+G(2df, p) basis set. Adiabatic ionization potentials (IP) and electron affinities (EA) were calculated as described in Forseman and Frisch.[12] The bond dissociation energies were calculated as in Yu and Reynisson.[13] The energies and ZPE are given in Table S6.

RMSD values < 1 Å were considered as excellent. The binding of *N*-[(1*S*)-2-hydroxy-1-phenylethyl]-4-[5-methyl-2-(phenylamino)pyrimidin-4-yl]-1*H*-pyrrole-2-carboxamide (Z48) in GSK3β, 4-(5-phenyl-1H-pyrrolo[2,3-b]pyridin-3-yl)benzoic acid (MMG) in SGK-1 and adenosine-3',5'-cyclic-monophosphate (CMP) in PKA was < 1 Å for all the scoring functions, while that of (3*E*)-3-[(4-hydroxyphenyl)imino]-1*H*-indol-2(3*H*)-one (LI7) to PIM1 was < 1 Å for GS and ChemPLP, but for CS and ASP were larger. CS predicted the phenol moiety to be in the same conformation as in the X-ray structure of the adduct, but the indole-2-one ring system adopted a twisted conformation as compared to its crystallographic counterpart albeit in the same space. Re-docking of 5-[(*E*)-(5-chloro-2-oxo-1,2-dihydro-3*H*-indol-3-ylidene)methyl]-*N*-[2-(diethylamino)ethyl]-2,4-dimethyl-1*H*-pyrrole-3-carboxamide (J60) into

the binding site of CaMK-1 was > 1 Å for all the scoring functions, in all cases the indole-2-one moiety was predicted to be in the same conformation as in the X-ray structure, ChemPLP and GS placed the pyrrole ring correctly, whereas all the scoring functions did not reproduce the pose of the chain containing the tertiary amine leading to relatively poor RMSD values.

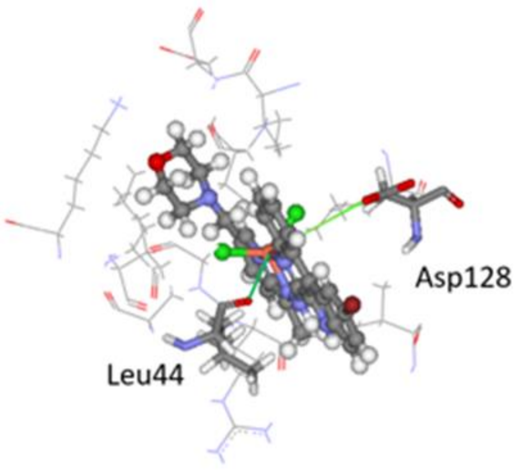

Figure S16. The predicted binding of complex **8** to PIM-1 (PDB ID: 1YXX), amino acids within 5 Å are shown in line format. The potential chelating amino acid residues Leu44 and Asp128 are shown as lines. The distance between the oxygen atom in the backbone of Leu44 is 4.7 Å and 5.5 Å (green solid lines) for the oxygen in the Asp128 side group who can both potentially replace the chlorido co-ligands.

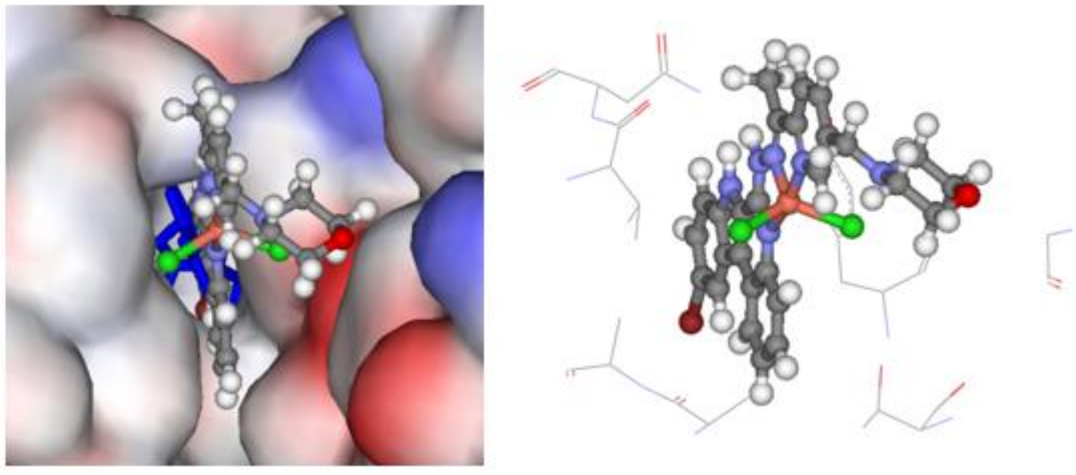

A **B**

Figure S17. (A) The docked configuration of **8** (ball-and stick format) in the PKA1 binding site (PDB ID: 3OF1) with the co-crystalized ligand CMP colored blue, stick format (its hydrogen atoms are hidden for clarity). The predicted conformation overlaps the co-crystalised ligand. The protein surface is rendered; blue depicts regions with a partial positive charge on the surface; red depicts regions with a partial negative charge and grey shows neutral areas. (B)

The predicted binding of complex **8**, amino acids within 5 Å are shown in line format. No potential chelating amino acid residues are within range of the copper atom.

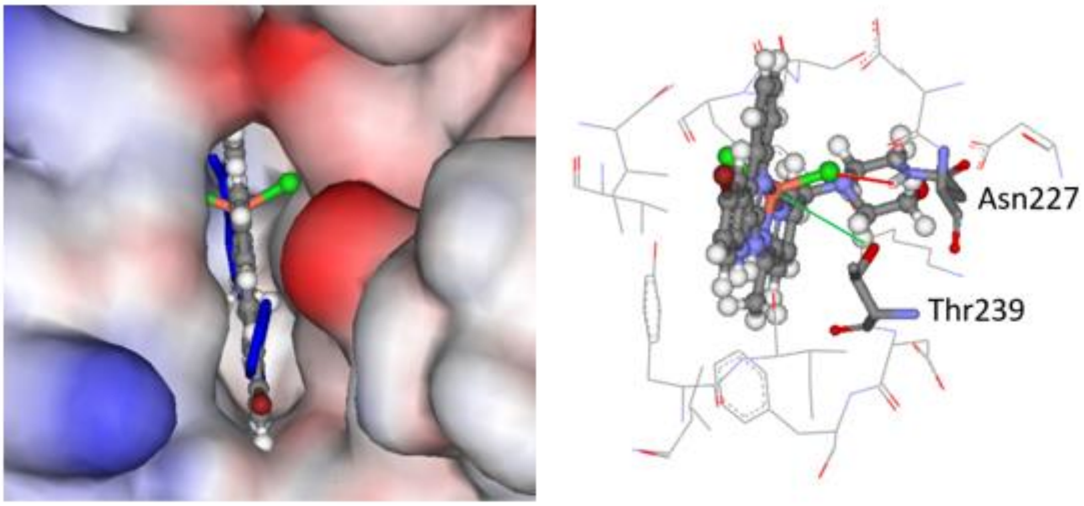

A B

Figure S18. The docked configuration of **8** (ball-and stick format) in the SGK-1 binding site (PDB ID: 3HDM) with the co-crystalized ligand MMG colored blue, stick format (its hydrogen atoms are hidden for clarity). The predicted pose completely overlaps the co-crystalized ligand. The protein surface is rendered; blue depicts regions with a partial positive charge on the surface; red depicts regions with a partial negative charge and grey shows neutral areas. (B) The predicted binding of complex **8**, amino acids within 5 Å are shown in line format. Amino acid residues Asn227 and Thr239 are shown as sticks. Asn227 is predicted to interact with one of the chlorido co-ligands of **8** (red line, 2.5 Å) and Thr239 can potentially chelate via its hydroxy group with the copper(II) (green line) albeit the distance is 4.2 Å.

- **Other molecular docking details**

Table S6. The single point and corrected zero-point vibrational energies (ZPE) of **HL[1]** in hartrees (a.u.).

Systems	Energy/a.u.	ZPE/a.u. [a]
**HL[1]**	–1448.684961	0.481717579
**HL**$^{\mathbf{1}\bullet+}$	–1448.442854	0.481310713
**HL**$^{\mathbf{1}\bullet-}$	–1448.728769	0.477730292
Fragment 1	–667.1981693	0.235283255
Fragment 2	–781.3834136	0.240069568

[a] According to Ref. 11

Table S7. The binding affinities as predicted by the scoring functions for the PIM1 kinase. LI7 is the co-crystalized ligand. Root-mean-square deviation – RMSD from the co-crystalized ligand (heavy atoms) in Å.

**Complexes**	**GS**	**Ligands**	**GS**	**CS**	**ChemPLP**	**ASP**
**1**	58.6	**HL[1]**	72.6	37.6	79.6	45.5
**2**	60.0	**HL[2]**	75.7	37.2	74.5	45.0
**3**	60.9	**HL[3]**	64.8	36.7	75.9	44.6
**4**	61.1	**HL[4]**	72.4	34.3	65.2	40.8
**5**	58.5	**HL[5]**	69.1	35.6	79.9	43.4
**6**	60.3	**HL[6]**	72.9	37.4	81.6	42.8
**7**	58.4	**HL[7]**	72.1	36.8	72.0	41.9
**8**	60.2	**HL[8]**	75.7	36.9	79.1	45.1
		**LI7**	50.5	31.4	58.0	28.4
		**RMSD:**	0.9020	3.1517	0.5348	6.7059

Table S8. The binding affinities as predicted by the scoring functions for the CaMK-1 kinase. J60 is the co-crystalized ligand. Root-mean-square deviation – RMSD from the co-crystalized ligand (heavy atoms) in Å.

**Complexes**	**GS**	**Ligands**	**GS**	**CS**	**ChemPLP**	**ASP**
**1**	57.3	**HL^1**	61.9	33.6	71.9	33.9
**2**	57.3	**HL^2**	62.3	33.1	69.7	34.4
**3**	58.1	**HL^3**	60.0	33.1	71.3	35.7
**4**	59.7	**HL^4**	58.6	29.0	67.2	28.6
**5**	54.8	**HL^5**	64.0	31.6	69.9	33.9
**6**	58.3	**HL^6**	63.3	31.6	65.3	31.7
**7**	55.5	**HL^7**	61.3	31.2	70.8	35.4
**8**	61.7	**HL^8**	62.3	30.0	63.3	29.8
		**J60**	60.9	31.9	70.5	31.7
		**RMSD:**	2.2669	4.6535	1.4495	3.2900

Table S9. The binding affinities as predicted by the scoring functions for the GSK3β kinase. Z48 is the co-crystalized ligand. Root-mean-square deviation – RMSD from the co-crystalized ligand (heavy atoms) in Å.

**Complexes**	**GS**	**Ligands**	**GS**	**CS**	**ChemPLP**	**ASP**
**1**	63.4	**HL^1**	67.6	34.1	67.3	36.8
**2**	67.7	**HL^2**	70.1	34.2	72.3	33.9
**3**	64.5	**HL^3**	64.4	34.0	73.3	36.8
**4**	66.8	**HL^4**	66.4	32.5	70.6	33.5
**5**	64.7	**HL^5**	70.0	33.8	77.3	38.1
**6**	66.6	**HL^6**	66.4	34.5	75.0	36.0
**7**	69.7	**HL^7**	68.6	34.4	76.0	37.1
**8**	70.9	**HL^8**	64.3	34.6	75.9	35.9
		**Z48**	93.3	41.7	102.6	44.2
		**RMSD:**	0.4811	0.8887	0.5860	0.5547

Table S10. The binding affinities as predicted by the scoring functions for the PKA. CMP is the co-crystalised ligand. Root-mean-square deviation – RMSD from the co-crystalised ligand (heavy atoms) in Å.

**Complexes**	**GS**		**GS**	**CS**	**ChemPLP**	**ASP**
**1**	56.1	**HL^1**	68.0	40.5	81.0	45.0
**2**	53.4	**HL^2**	62.9	38.5	76.0	39.1
**3**	42.8	**HL^3**	70.1	41.0	87.8	45.5
**4**	35.9	**HL^4**	67.2	38.3	81.4	42.0
**5**	55.4	**HL^5**	64.1	38.6	84.0	45.3
**6**	59.0	**HL^6**	67.1	38.8	81.4	45.4
**7**	60.7	**HL^7**	61.7	37.2	81.0	46.9
**8**	59.9	**HL^8**	65.8	38.1	81.2	48.0
		**CMP**	82.2	26.8	82.5	54.0
		**RMSD:**	0.3329	0.2472	0.5882	0.4436

Table S11. The binding affinities as predicted by the scoring functions for the SGK-1. MMG is the co-crystalised ligand. Root-mean-square deviation – RMSD from the co-crystalised ligand (heavy atoms) in Å.

**Complexes**	**GS**		**GS**	**CS**	**ChemPLP**	**ASP**
**1**	72.4	**HL^1**	76.8	39.0	68.3	37.6
**2**	73.7	**HL^2**	73.2	40.4	67.5	37.9
**3**	75.2	**HL^3**	68.8	37.4	74.4	37.1
**4**	75.5	**HL^4**	67.2	38.3	68.9	37.3
**5**	75.6	**HL^5**	71.0	39.8	75.5	39.5
**6**	78.1	**HL^6**	71.9	41.7	73.3	39.2
**7**	78.0	**HL^7**	73.6	42.3	76.9	39.3
**8**	78.6	**HL^8**	74.3	40.9	75.5	38.7
		**MMG**	62.7	39.4	76.8	34.1
		**RMSD:**	0.9197	0.6643	0.4187	0.4686

Table S12. Definition of lead-like, drug-like and Known Drug Space (KDS) in terms of molecular descriptors. The values given are the maxima for each descriptor for the volumes of chemical space used.

	**Lead-like Space**	**Drug-like Space**	**Known Drug Space**
Molecular weight (g/mol)	300	500	800
Lipophilicity (log*P*)	3	5	6.5
Hydrogen bond donors (HD)	3	5	7
Hydrogen bond acceptors (HA)	3	10	15
Polar surface area ($Å^2$) (PSA)	60	140	180
Rotatable bonds (RB)	3	10	17

Table S13. The molecular descriptors as calculated by QikProp and their corresponding Known Drug Indexes 2A and 2B ($KDI_{2A/2B}$).

	**RB**	**MW(g/mol)**	**HD**	**HA**	**Log*P***	**PSA ($Å^2$)**	**KDI_{2A}**	**KDI_{2B}**
**HL^1**	5	450.5	2	7.7	4.3	77.4	5.36	0.49
**HL^2**	5	529.4	2	7.7	4.8	77.5	4.81	0.19
**HL^3**	5	464.6	2	7.2	4.8	73.9	5.23	0.40
**HL^4**	5	543.5	2	7.2	5.4	73.8	4.68	0.14
**HL^5**	5	436.5	2	7.7	4.0	69.2	5.46	0.55
**HL^6**	5	515.4	2	7.7	4.6	69.2	4.92	0.25
**HL^7**	5	450.5	2	7.2	4.6	65.8	5.34	0.48
**HL^8**	5	529.4	2	7.2	5.1	65.7	4.78	0.18

Table S14. The molecular descriptors as calculated by Scigress sofware.

	**MW(g/mol)**	**HD**	**HA**	**Log*P***
**HL^1**	450.5	2	7	4.4
**HL^2**	529.4	2	7	5.2
**HL^3**	464.6	2	7	3.7
**HL^4**	543.5	2	7	4.5
**HL^5**	436.5	2	7	4.3
**HL^6**	515.4	2	7	5.1
**HL^7**	450.5	2	7	3.6
**HL^8**	529.4	2	7	4.4
**1**	585.0	2	7	n.d.
**2**	663.9	2	7	n.d.
**3**	599.0	2	7	n.d.
**4**	677.9	2	7	n.d.
**5**	571.0	2	7	n.d.
**6**	649.9	2	7	n.d.
**7**	585.0	2	7	n.d.
**8**	663.9	2	7	n.d.

- **Yields and analytical data of isolated proligands and Cu(II) complexes**

Table S15. Yields and analytical data for **HL^1^–HL^4^**.

		**HL^1^**	**HL^2^**	**HL^3^**	**HL^4^**
**Yield (%)**		98	32	67	57
**Brutto formula**		$C_{27}H_{26}N_6O\cdot H_2O$	$C_{27}H_{25}BrN_6O\cdot 0.6H_2O$	$C_{28}H_{28}N_6O\cdot 0.2H_2O$	$C_{28}H_{27}BrN_6O$
$\boldsymbol{M_r}$		468.55	540.24	468.16	545.15
**C (%)**	**calcd**	68.93	60.02	71.82	61.88
	**found**	69.21	59.97	71.83	61.63
**H (%)**	**calcd**	6.07	4.88	6.11	5.01
	**found**	6.02	4.93	6.00	4.99
**N (%)**	**calcd**	17.81	15.55	17.95	15.46
	**found**	17.93	15.21	17.84	15.33
**O (%)**	**calcd**				
	**found**				
**ESI-MS**	**$[M + Na]^+$**				
**(methanol)**	**$[M + H]^+$**	451	531	465	545
**X-ray structure**		no	no	no	no

Table S16. Yields and analytical data for **HL5–HL8**.

		**HL^5**	**HL^6**	**HL^7**	**HL^8**
**Yield (%)**		91	78	94	58
**Brutto formula**		$C_{26}H_{24}N_6O$ $0.5H_2O$	$C_{26}H_{23}BrN_6O·H_2O$	$C_{27}H_{26}N_6O·0.5H_2O$	$C_{26}H_{25}BrN_6O·0.5H_2O$
M_r		445.52	532.12	459.54	538.44
**C (%)**	**calcd**	70.09	58.54	70.57	60.22
	**found**	69.79	58.69	70.46	59.94
**H (%)**	**calcd**	5.66	4.72	5.92	4.87
	**found**	5.68	4.70	5.76	4.92
**N (%)**	**calcd**	18.86	15.75	18.28	15.61
	**found**	18.68	15.71	18.14	15.78
**O (%)**	**calcd**				
	**found**				
**ESI-MS**	**$[M + Na]^+$**				
**(methanol)**	**$[M + H]^+$**	437	517	451	531
**X-ray structure**		no	yes	no	no

Table S17. Yields and analytical data fo1 **1–4**.

		**1**	**2**	**3**	**4**
**Yield (%)**		99	75	82	34.9
**Brutto formula**		$C_{27}H_{26}Cl_2CuN_6O$ $\cdot H_2O$	$C_{27}H_{25}BrCl_2CuN_6O$	$C_{28}H_{28}Cl_2CuN_6O$ $\cdot 0.5CH_4O$	$C_{28}H_{27}Cl_2CuN_6O_2$ $\cdot 0.5H_2O$
***M*r**		603.00	699.91	615.03	675.01
**C (%)**	**calcd**	53.90	46.33	55.65	49.12
	**found**	53.98	46.52	55.55	49.51
**H (%)**	**calcd**	4.69	4.18	4.91	4.12
	**found**	4.67	3.96	4.83	4.18
**N (%)**	**calcd**	13.98	12.01	13.66	12.28
	**found**	13.79	11.99	13.27	11.98
**O (%)**	**calcd**				
	**found**				
**ESI-MS**	**$[Cu(L^n)]^+$**	512, 553	592, 633	553	633
**(methanol HCN/CH_3CN[14])**	**$[CuCl(HL^n)]^+$**	548		562	
**X-ray structure**		yes	yes	yes	no

Table S18. Yields and analytical data for **5**–**8** and **[Ni(HL^{7})$_2$]Cl$_2$·H$_2$O**.

		**5**	**6**	**7**	**8**	**[Ni(HL^{7})$_2$]Cl$_2$·H$_2$O**
**Yield (%)**		99	75	82	34.9	60
**Brutto formula**		$C_{26}H_{24}Cl_2CuN_6O$ ·1.5H_2O	$C_{26}H_{23}BrCl_2CuN_6O$ ·1.5H_2O	$C_{27}H_{26}Cl_2CuN_6O$ ·0.5H_2O	$C_{27}H_{25}BrCl_2CuN_6O_2$ ·H_2O	$C_{54}H_{52}N_{12}O_2NiCl_2$ ·H_2O
***M*$_r$**		597.98	676.88	593.99	681.90	1148.32
**C (%)**	**calcd**	52.22	46.14	54.59	47.56	61.85
	**found**	52.06	46.18	54.60	47.49	61.82
**H (%)**	**calcd**	4.55	3.87	4.58	3.99	5.19
	**found**	4.20	3.53	4.40	3.64	5.13
**N (%)**	**calcd**	14.05	12.41	14.14	12.32	16.03
	**found**	13.76	12.15	14.03	12.17	16.18
**O (%)**	**calcd**					
	**found**					
**ESI-MS**	**[Cu(L^{n})]$^{+}$**	498	578	512	592	
**(methanol)**	**[CuCl(HL^{n})]$^{+}$**					
	**[Ni(HL^{7})$_2$]$^{2+}$**					479.18
**X-ray structure**		yes	no	no	no	no

- **NMR spectra**
    - **^{1}H NMR spectra**

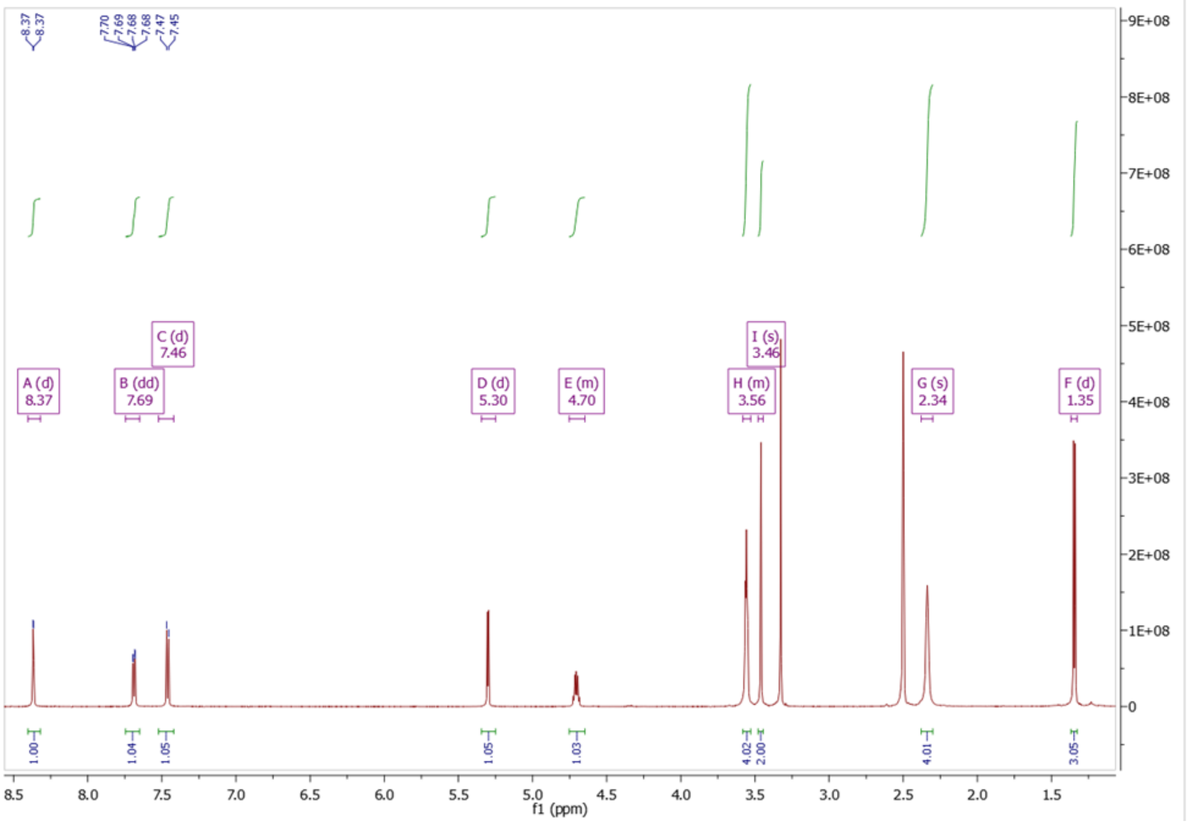

Figure S19. ^{1}H NMR spectrum of **I**.

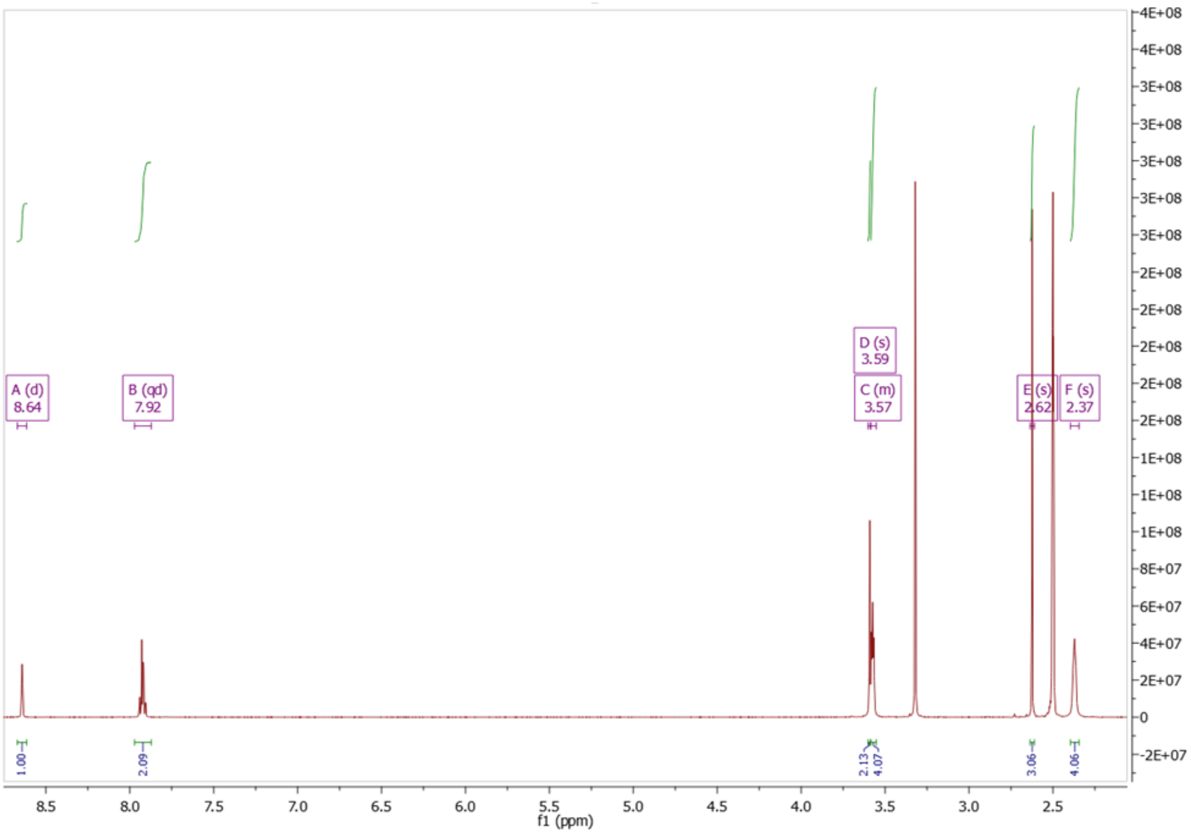

Figure S20. ^{1}H NMR spectrum of **J**.

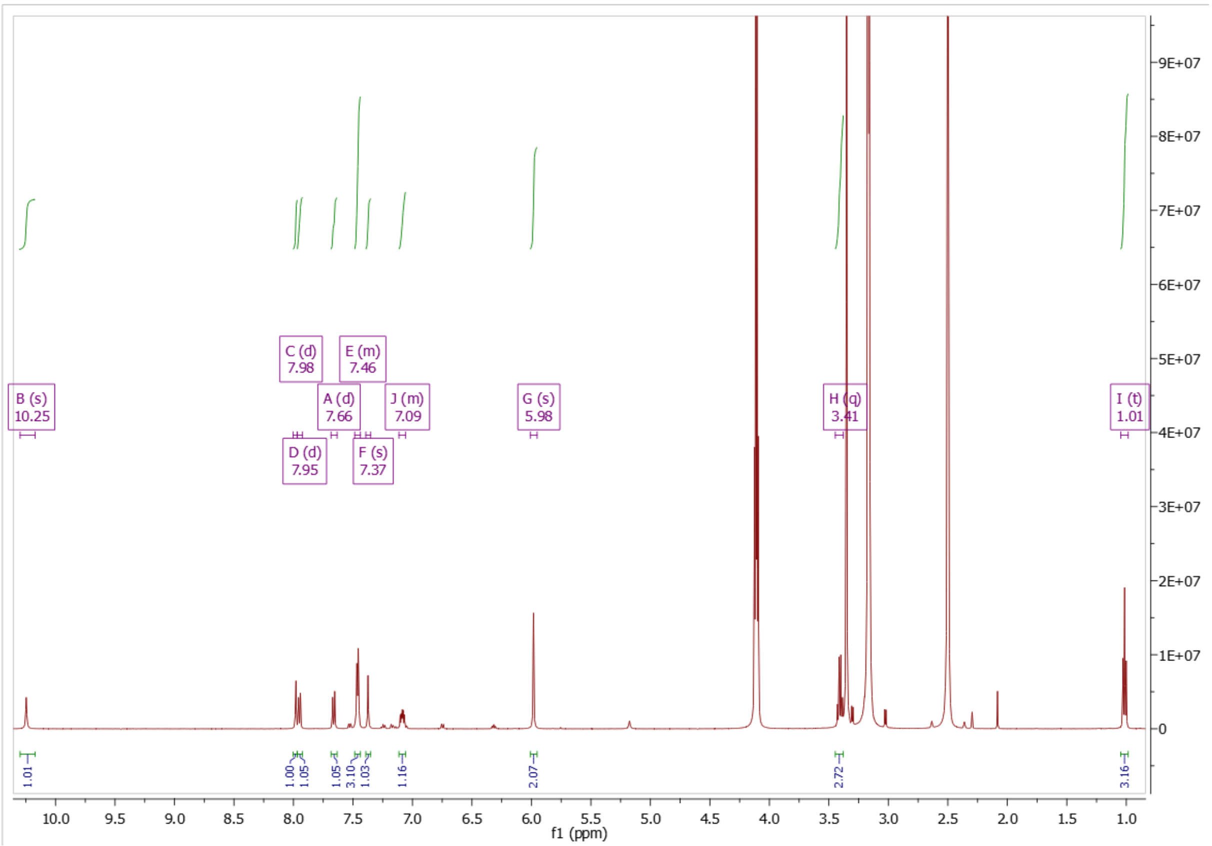

Figure S21. ^{1}H NMR spectrum of **Ib**.

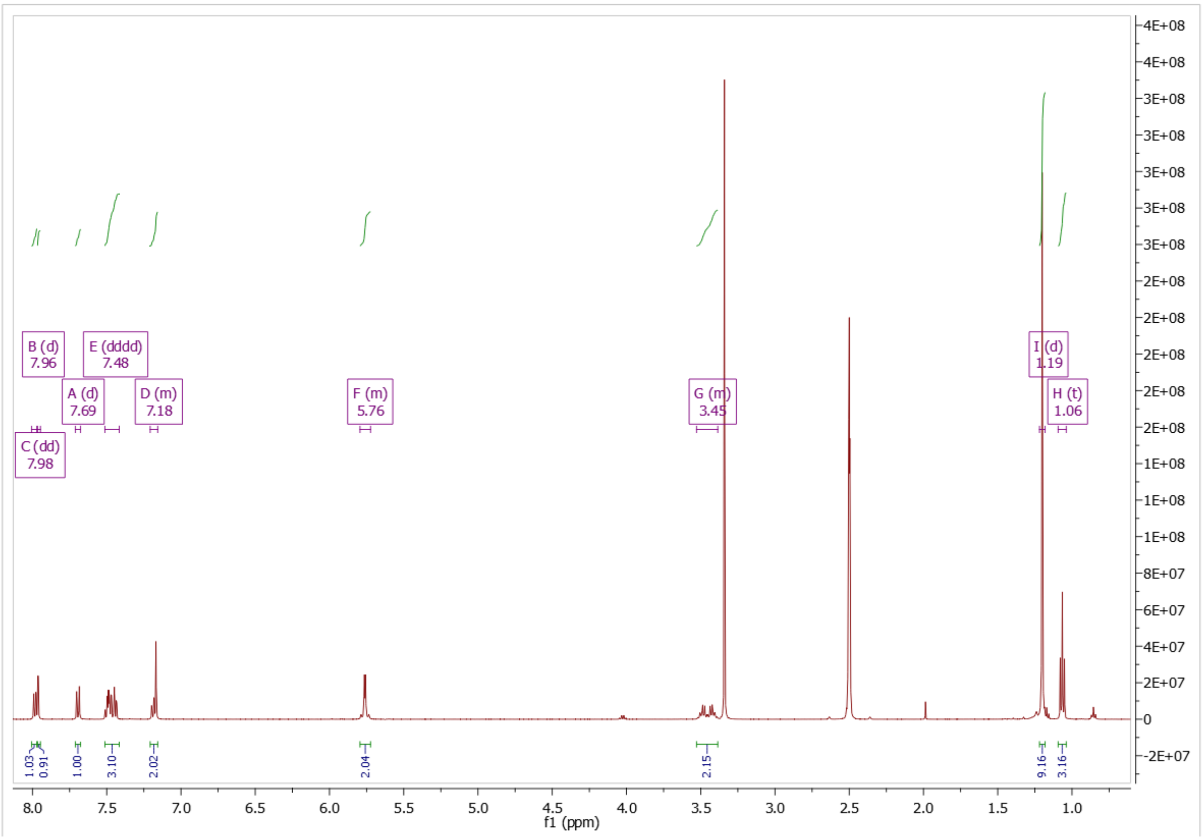

Figure S22. ^{1}H NMR spectrum of **IIb**.

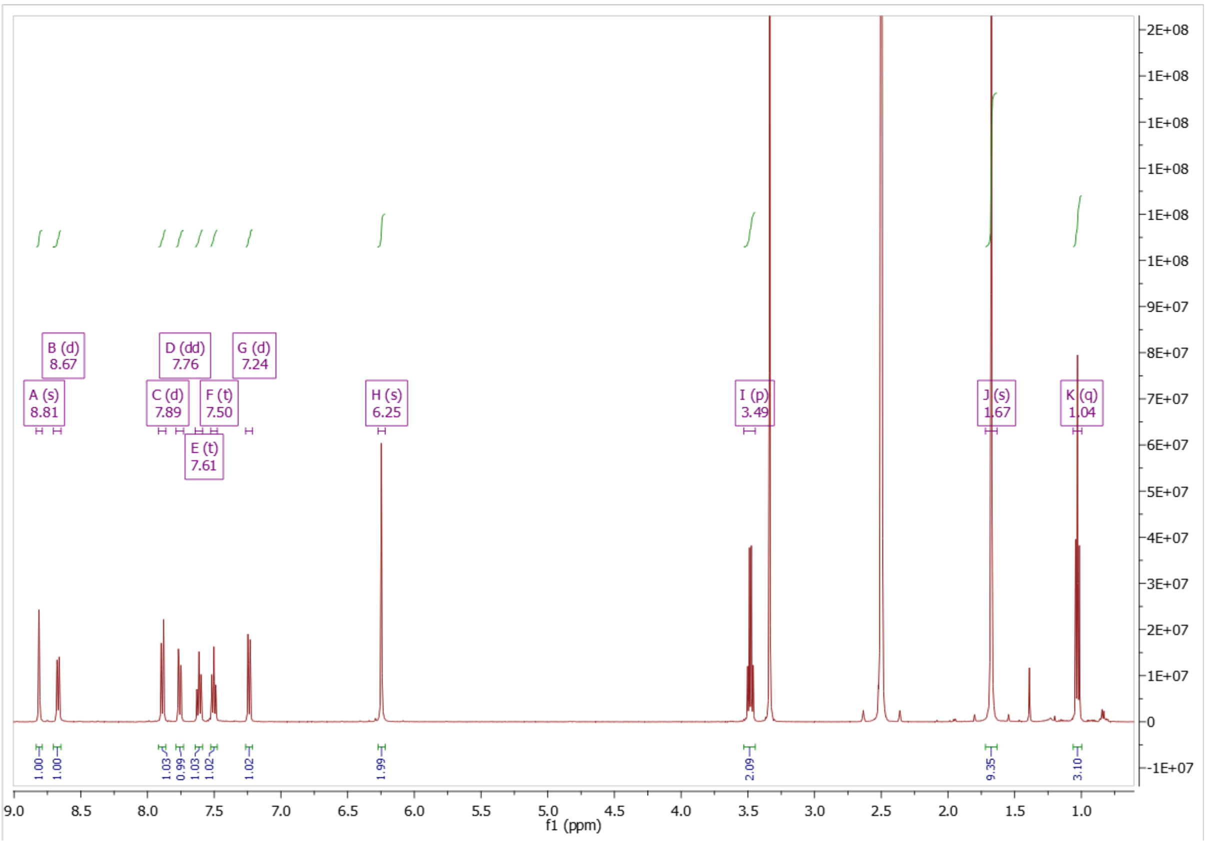

Figure S23. ^{1}H NMR spectrum of **IIIb**.

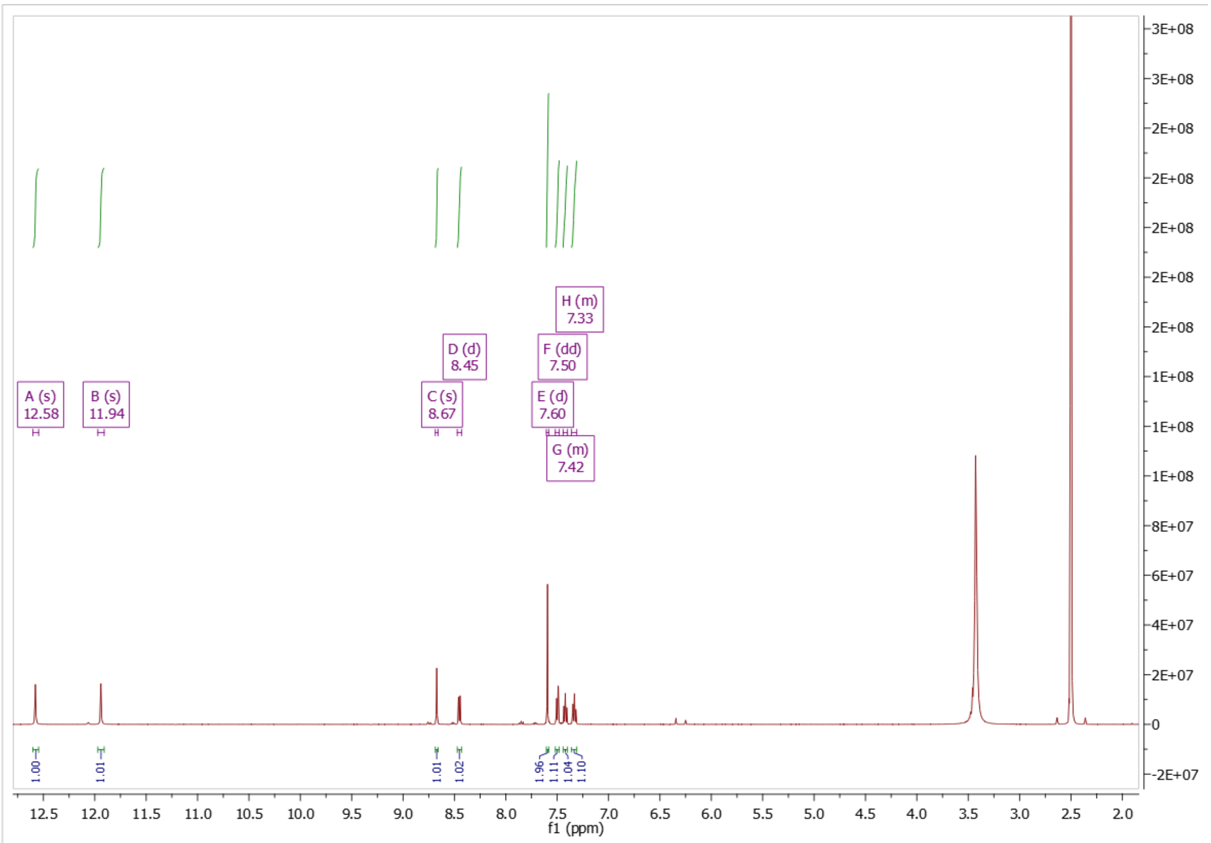

Figure S24. ^{1}H NMR spectrum of **IVb**.

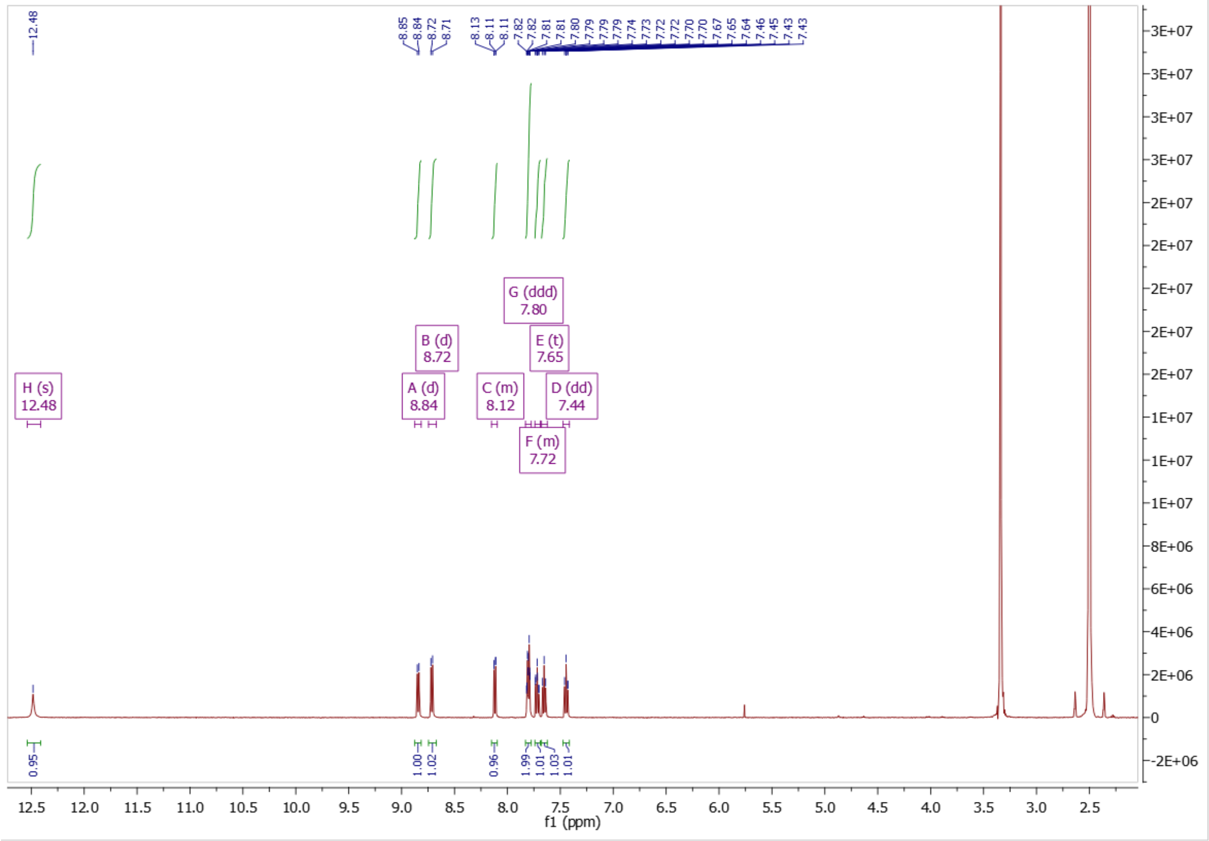

Figure S25. ^{1}H NMR spectrum of **Va**.

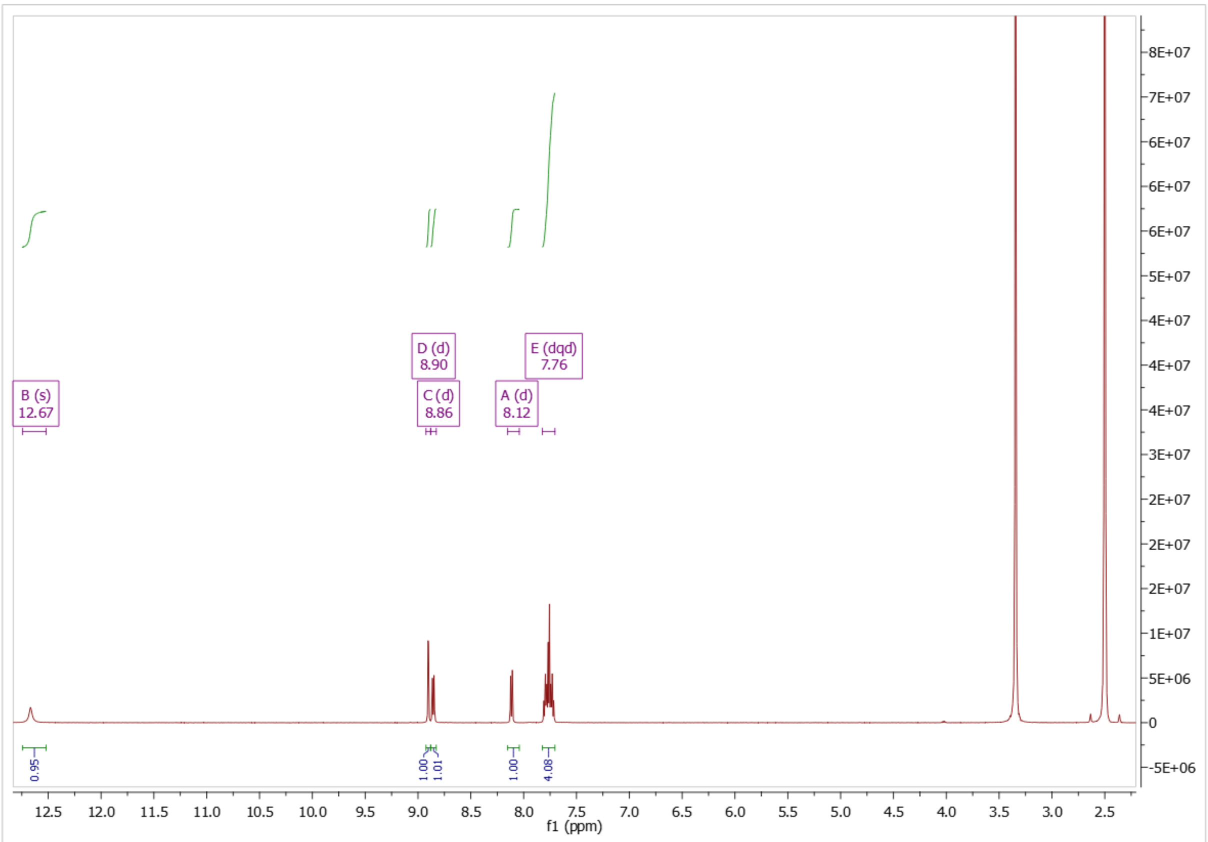

Figure S26. ^{1}H NMR spectrum of **Vb**.

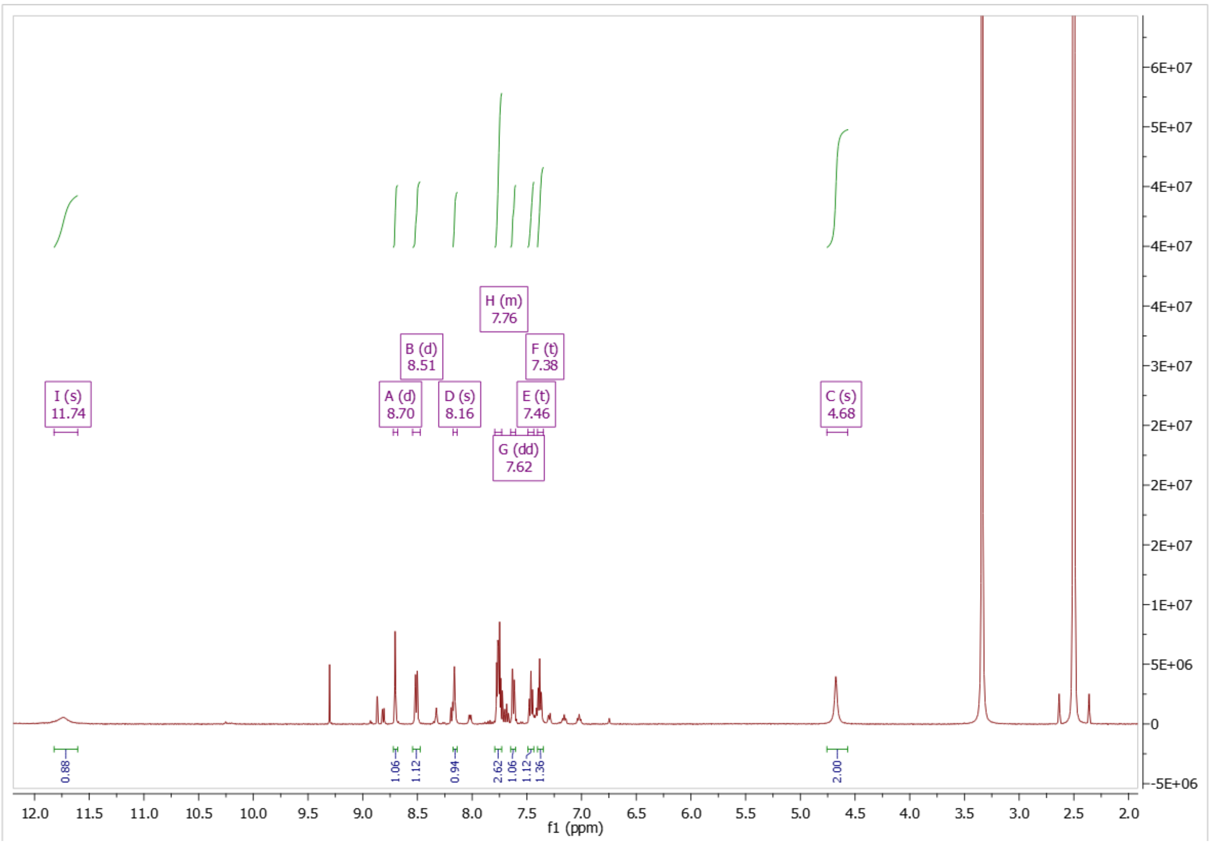

Figure S27. ¹H NMR spectrum of **M**.

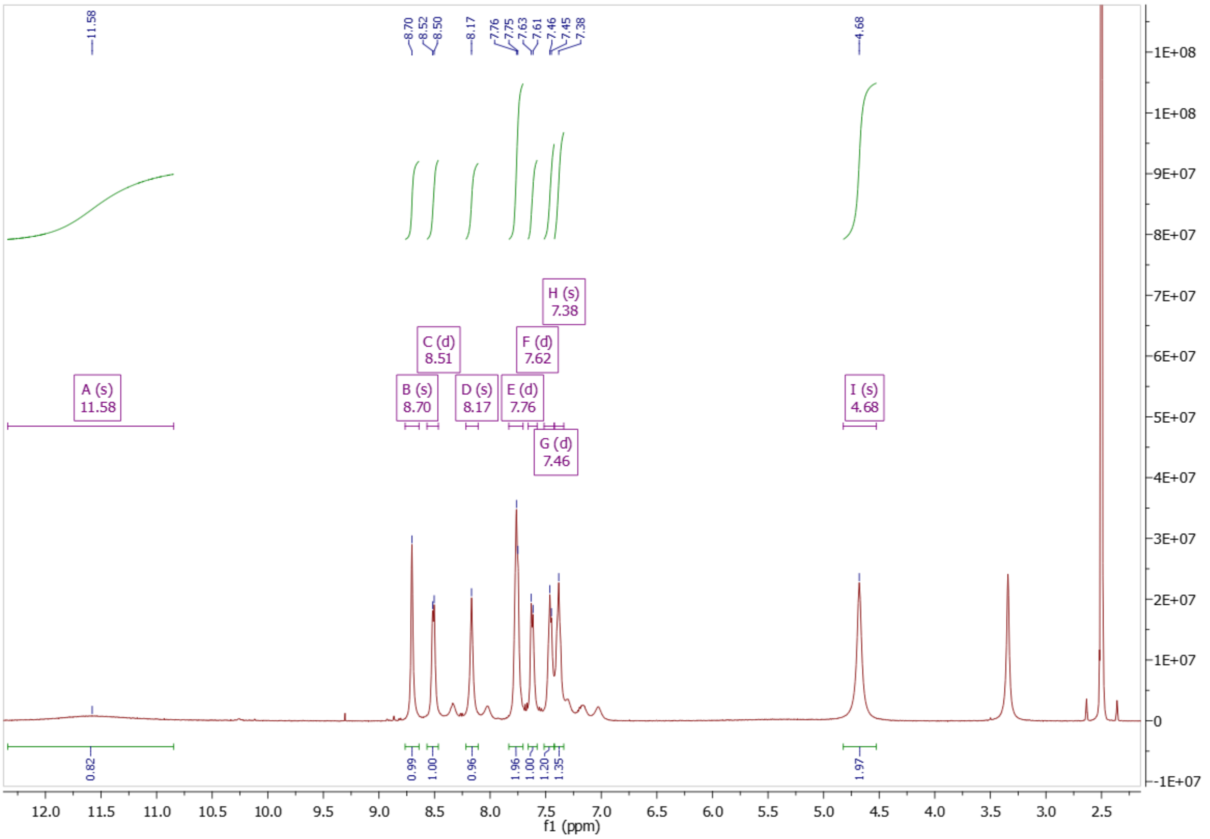

Figure S28. ¹H NMR spectrum of **N**.

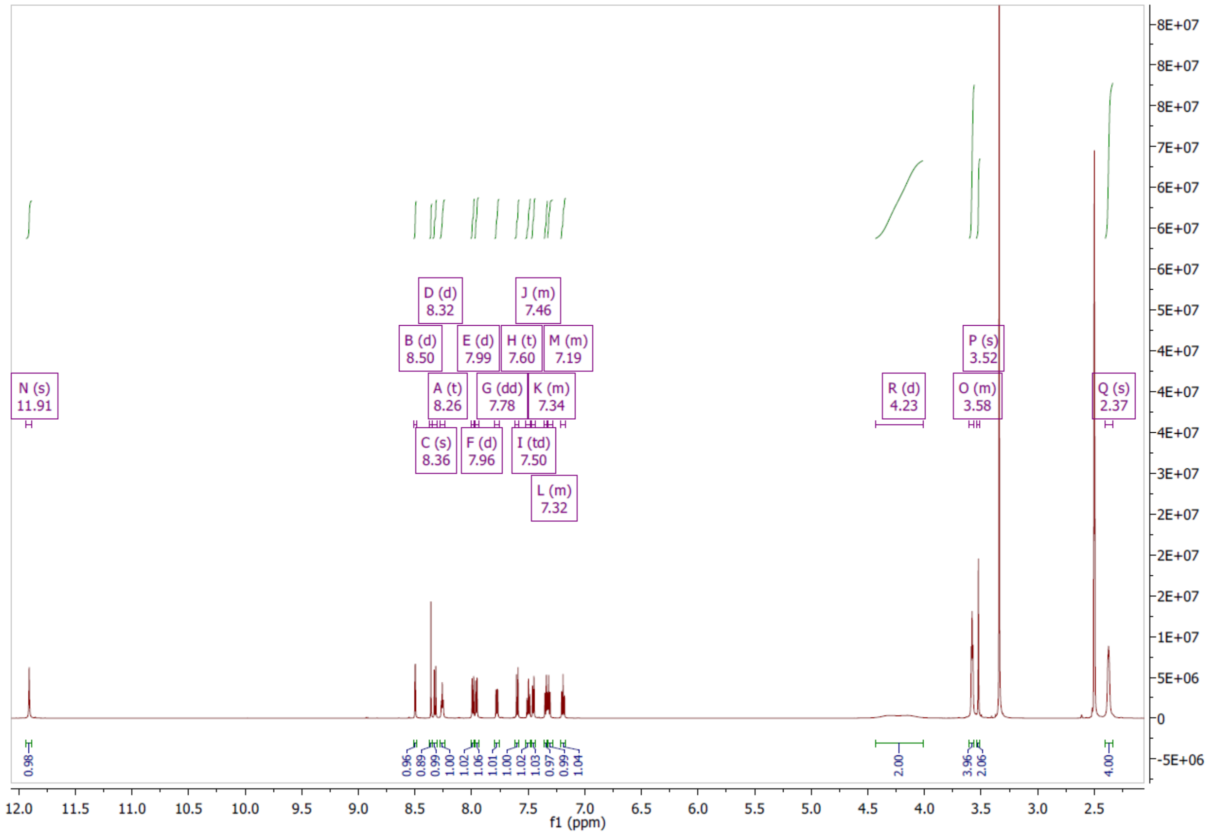

Figure S29. ^{1}H NMR spectrum of **HL^{1}**.

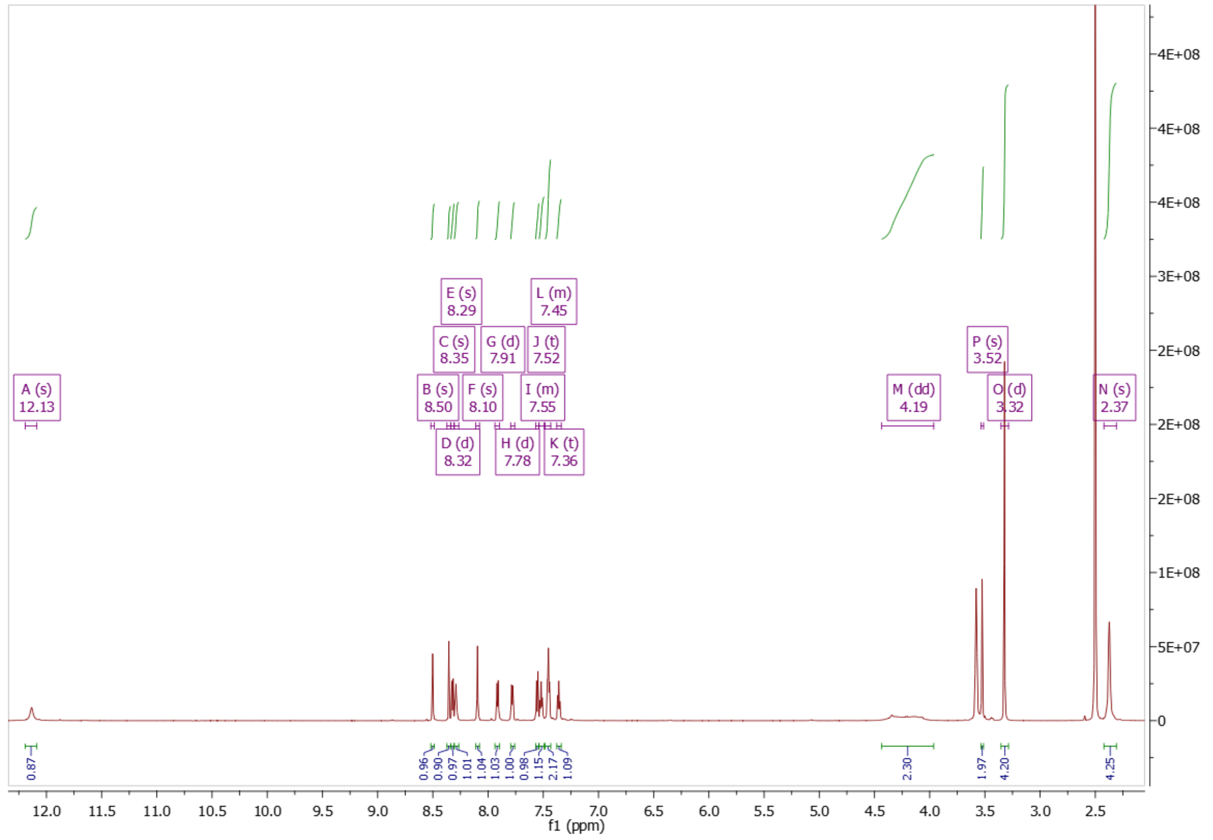

Figure S30. ^{1}H NMR spectrum of **HL^{2}**.

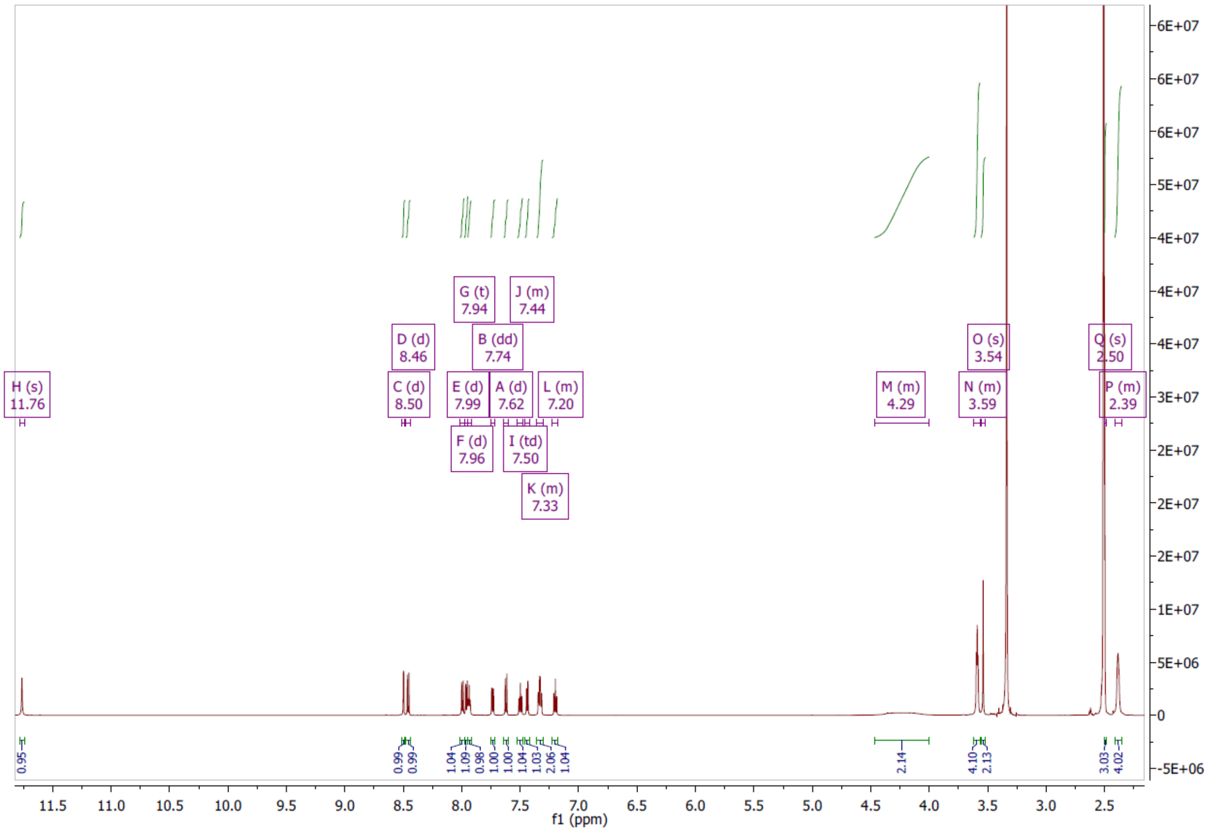

Figure S31. ^{1}H NMR spectrum of **HL^{3}**.

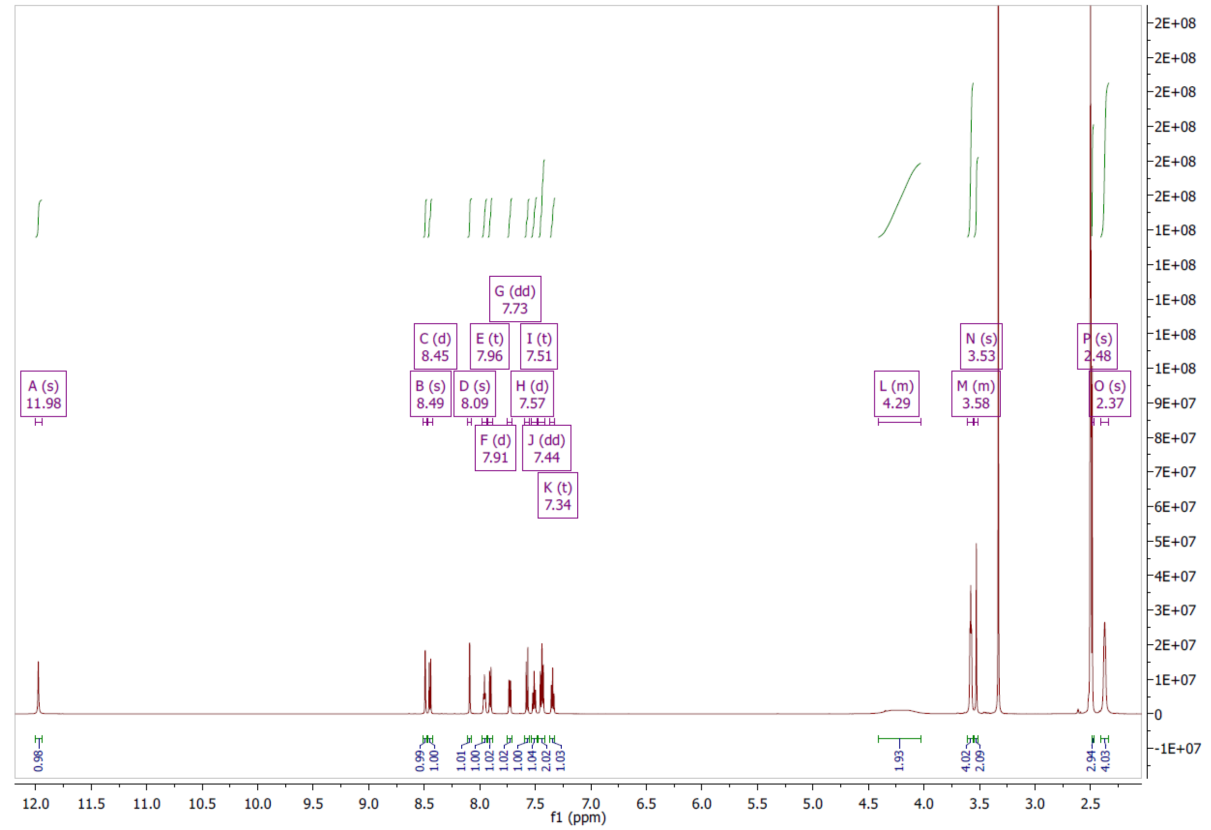

Figure S32. ^{1}H NMR spectrum of **HL^{4}**.

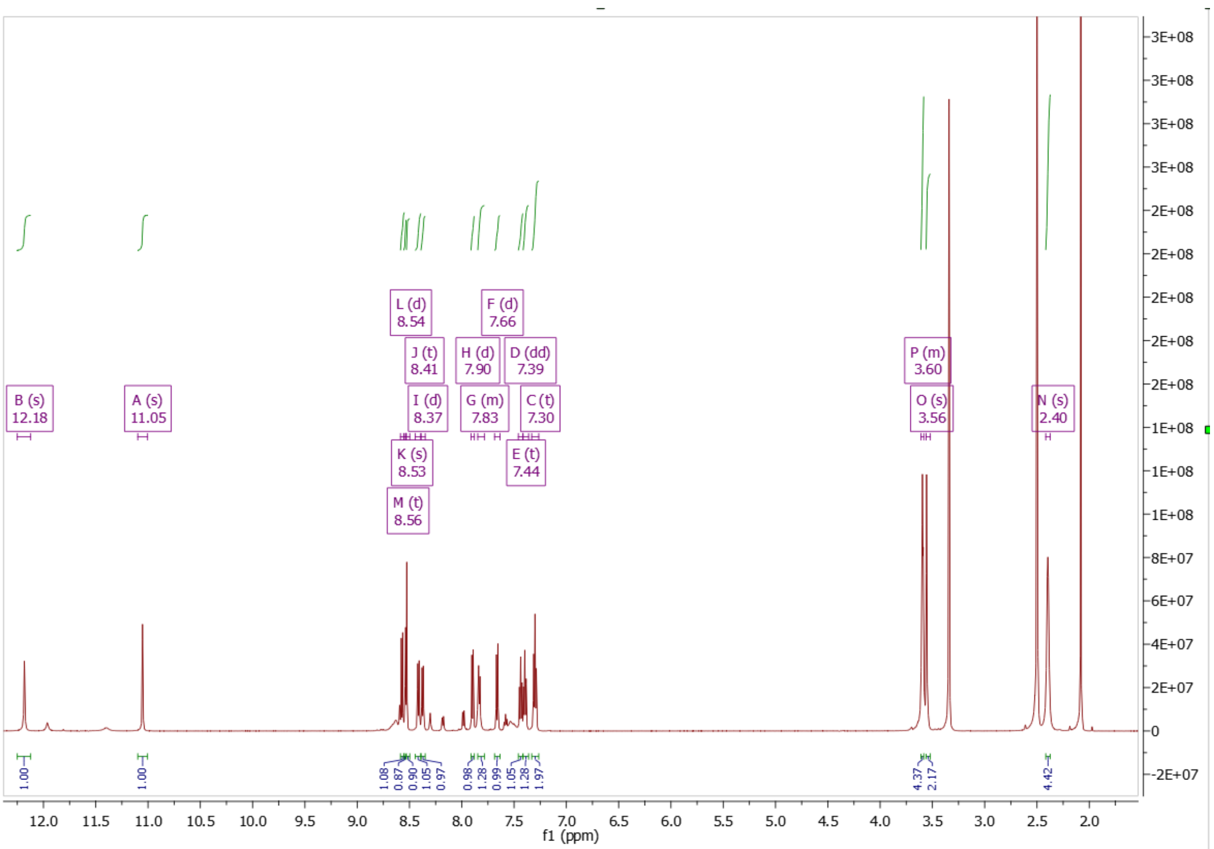

Figure S33. ¹H NMR spectrum of **HL⁵**.

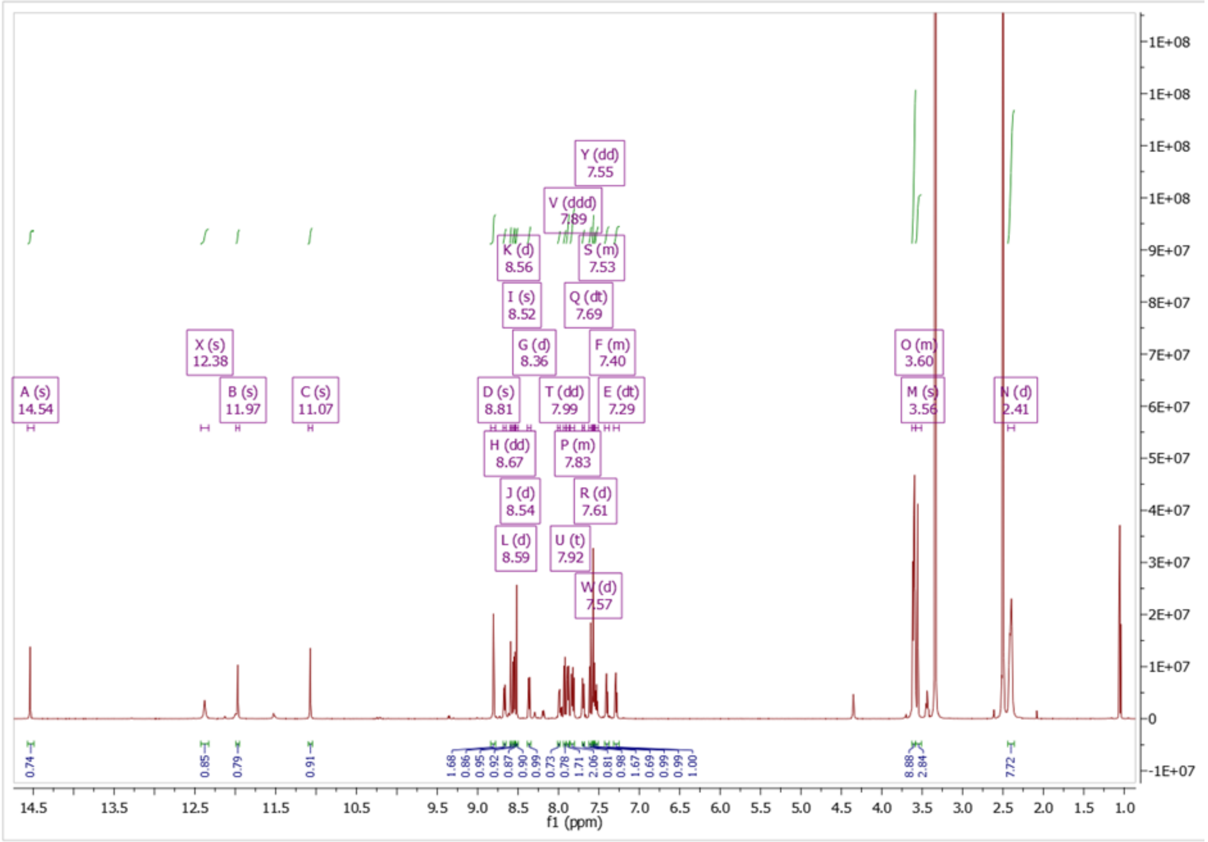

Figure S34. ¹H NMR spectrum of **HL⁶**.

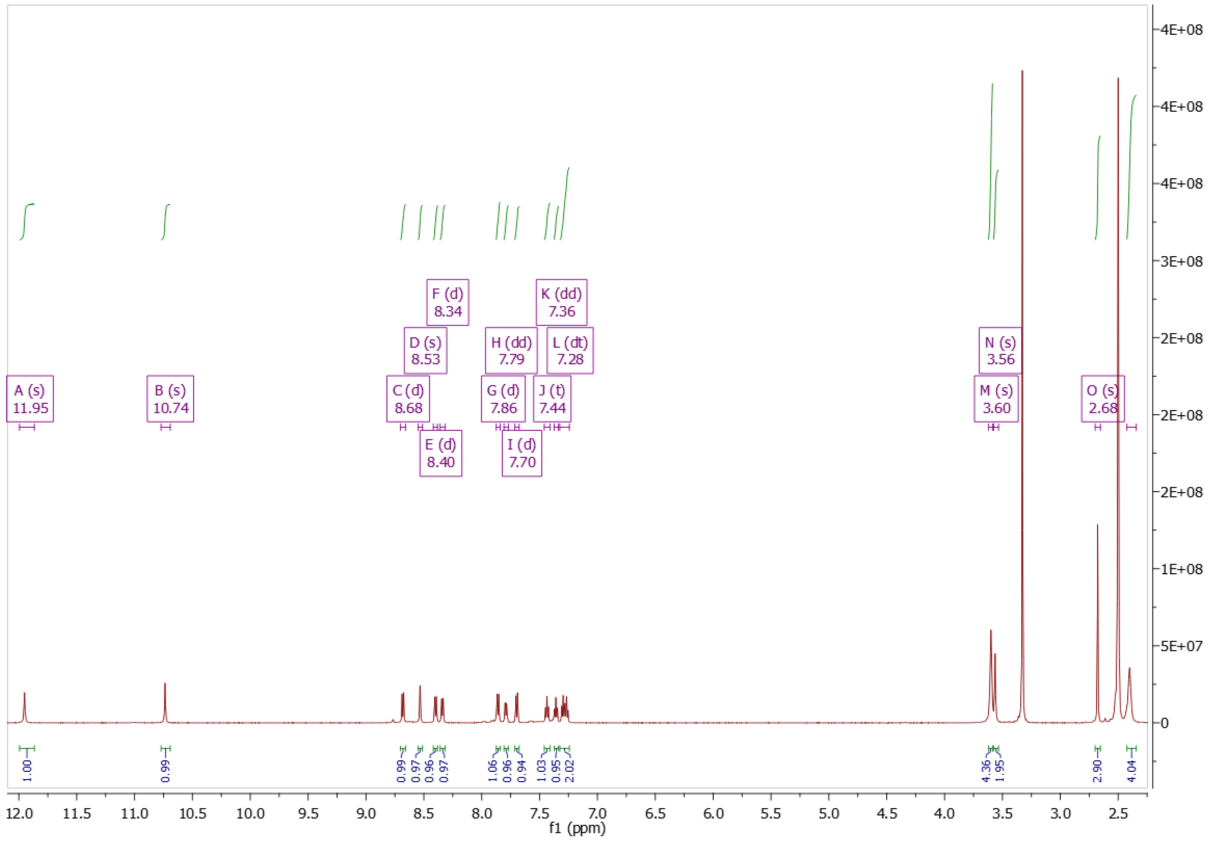

Figure S35. ^{1}H NMR spectrum of **HL^{7}**.

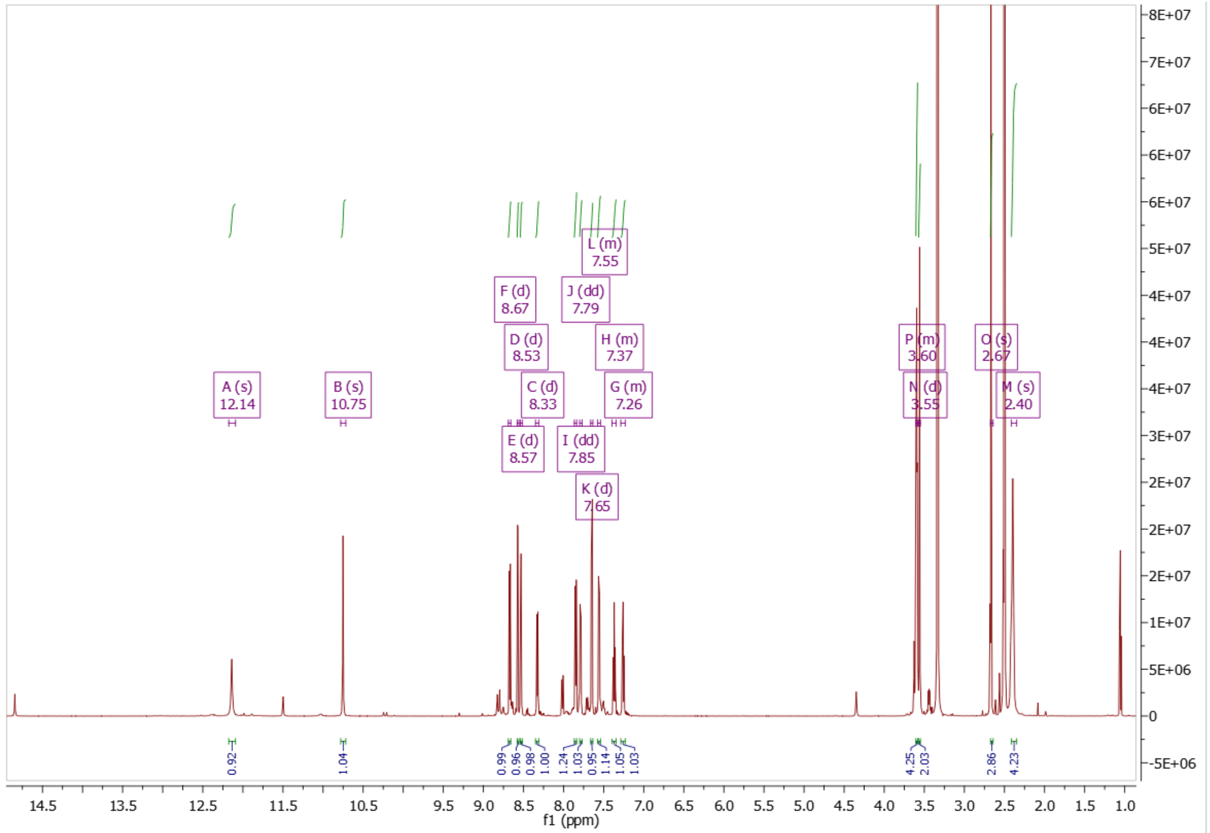

Figure S36. ^{1}H NMR spectrum of **HL^{8}**.

○ ^{13}C NMR spectra

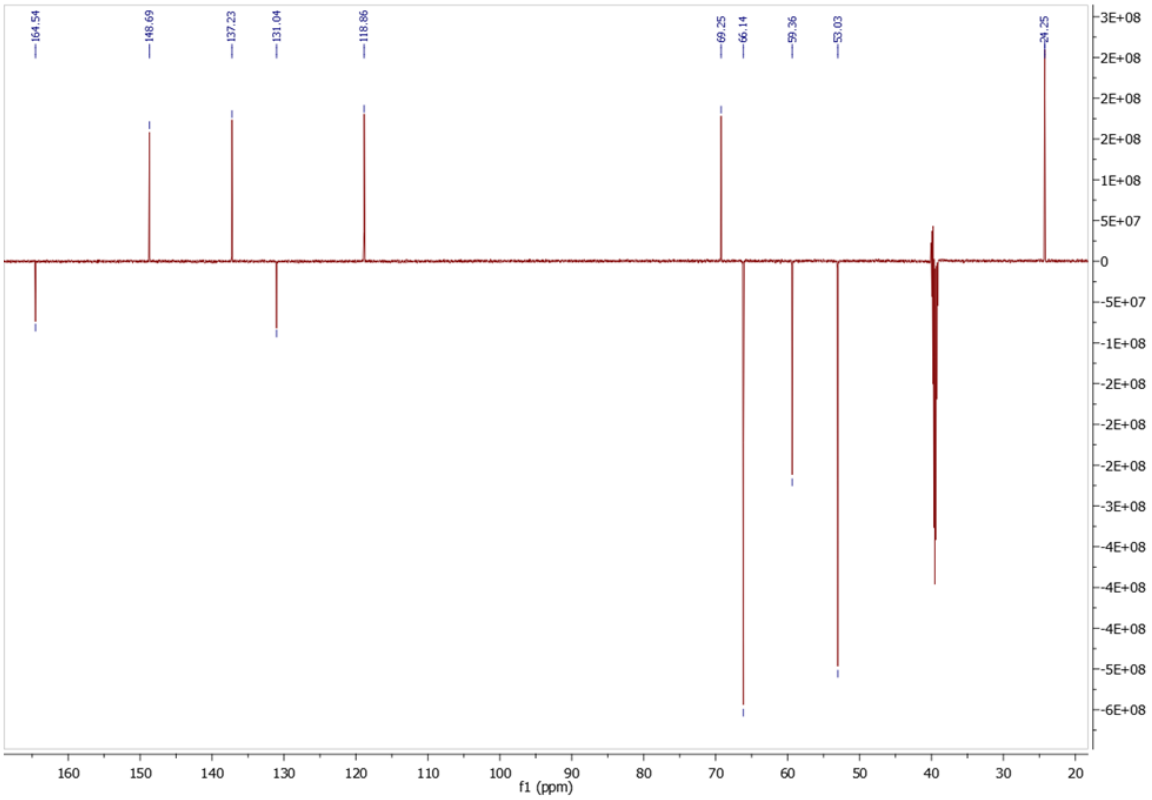

Figure S37. ^{13}C NMR spectrum of **I**.

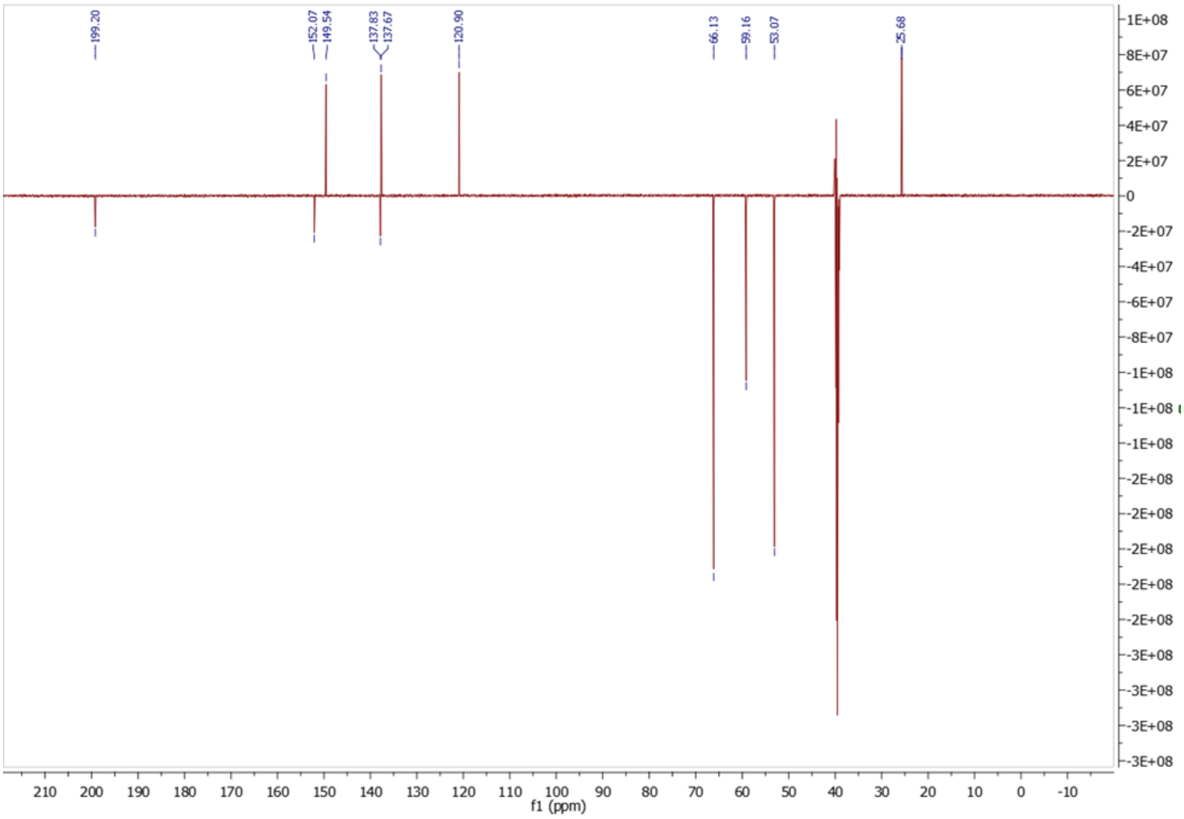

Figure S38. ^{13}C NMR spectrum of **J**.

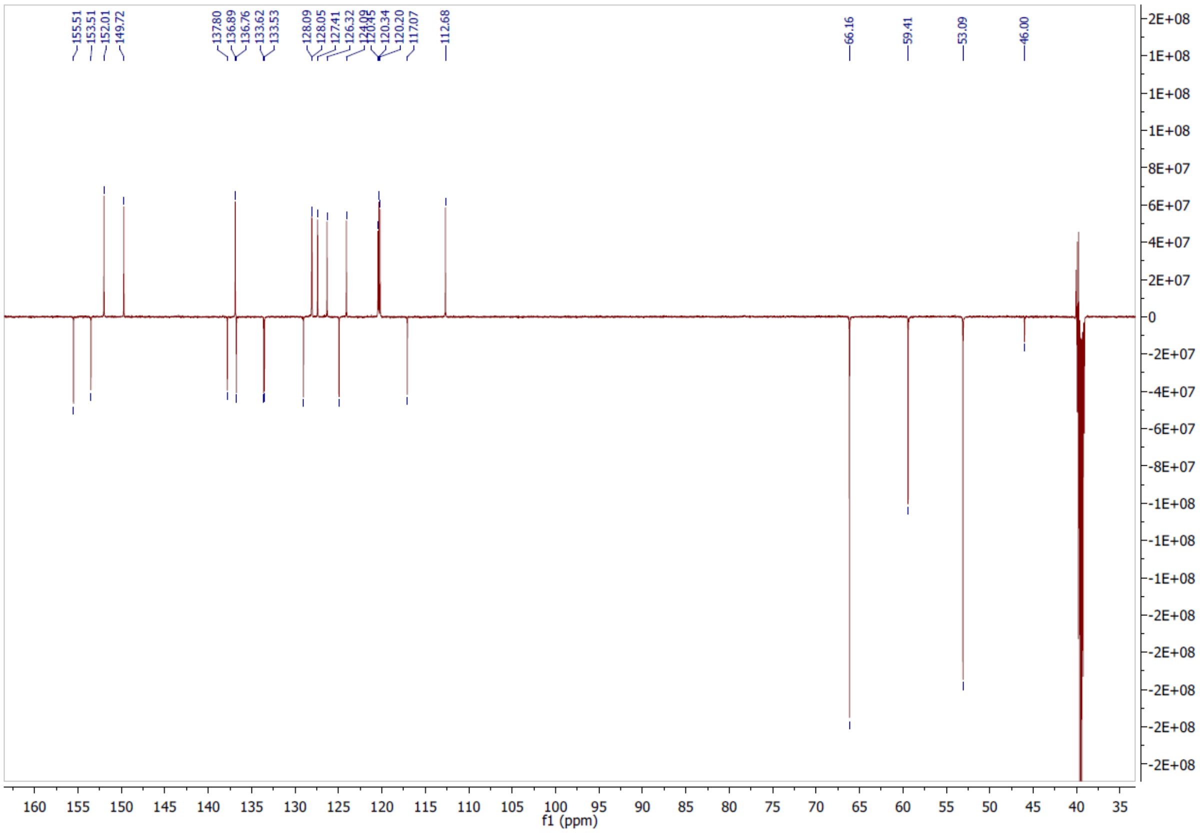

Figure S39. ^{13}C NMR spectrum of **HL^{1}**.

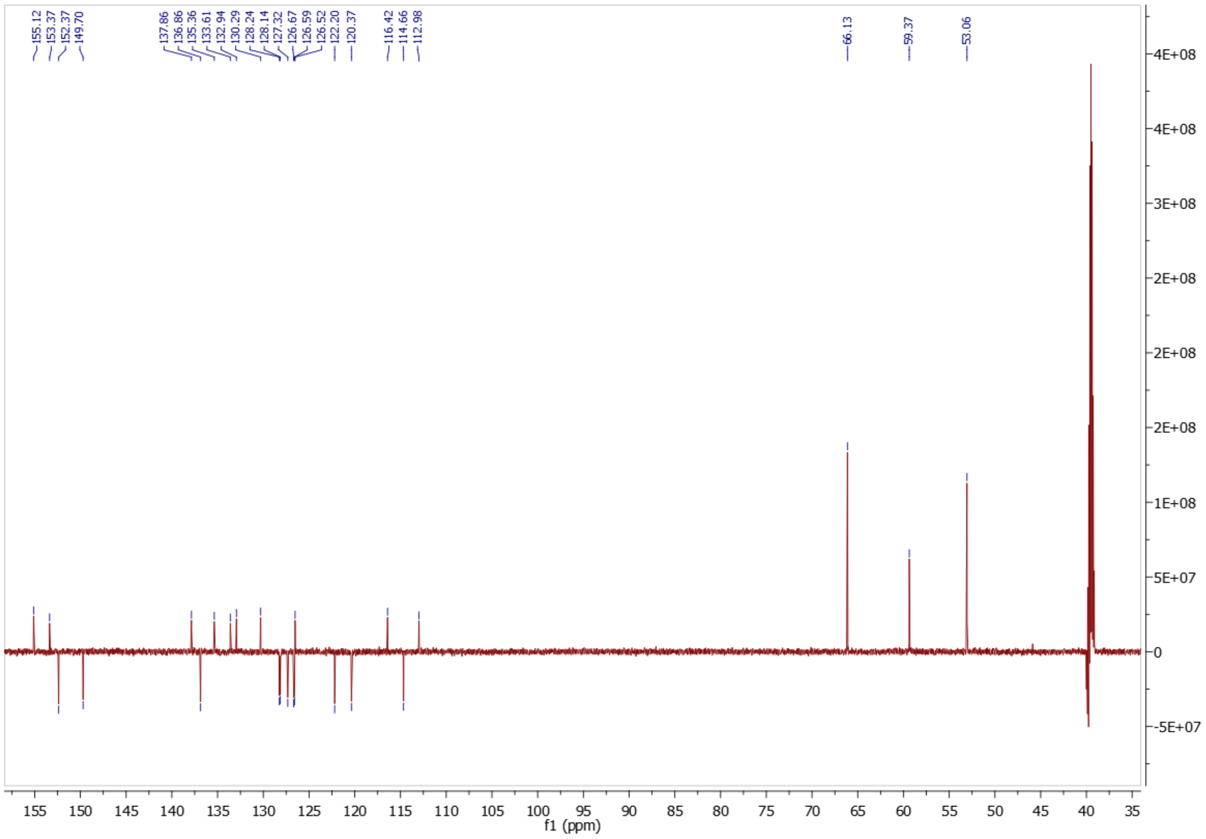

Figure S40. ^{13}C NMR spectrum of **HL^{2}**.

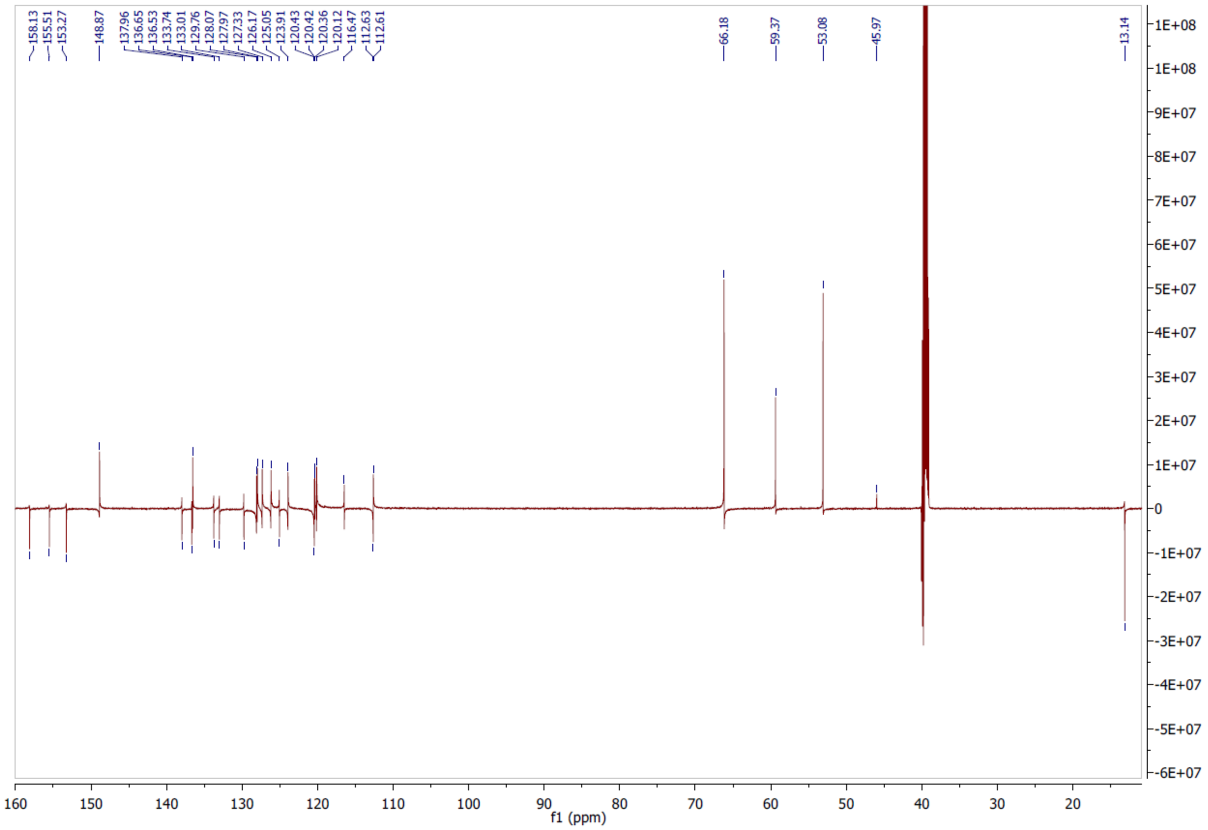

Figure S41. ^{13}C NMR spectrum of **HL^3**.

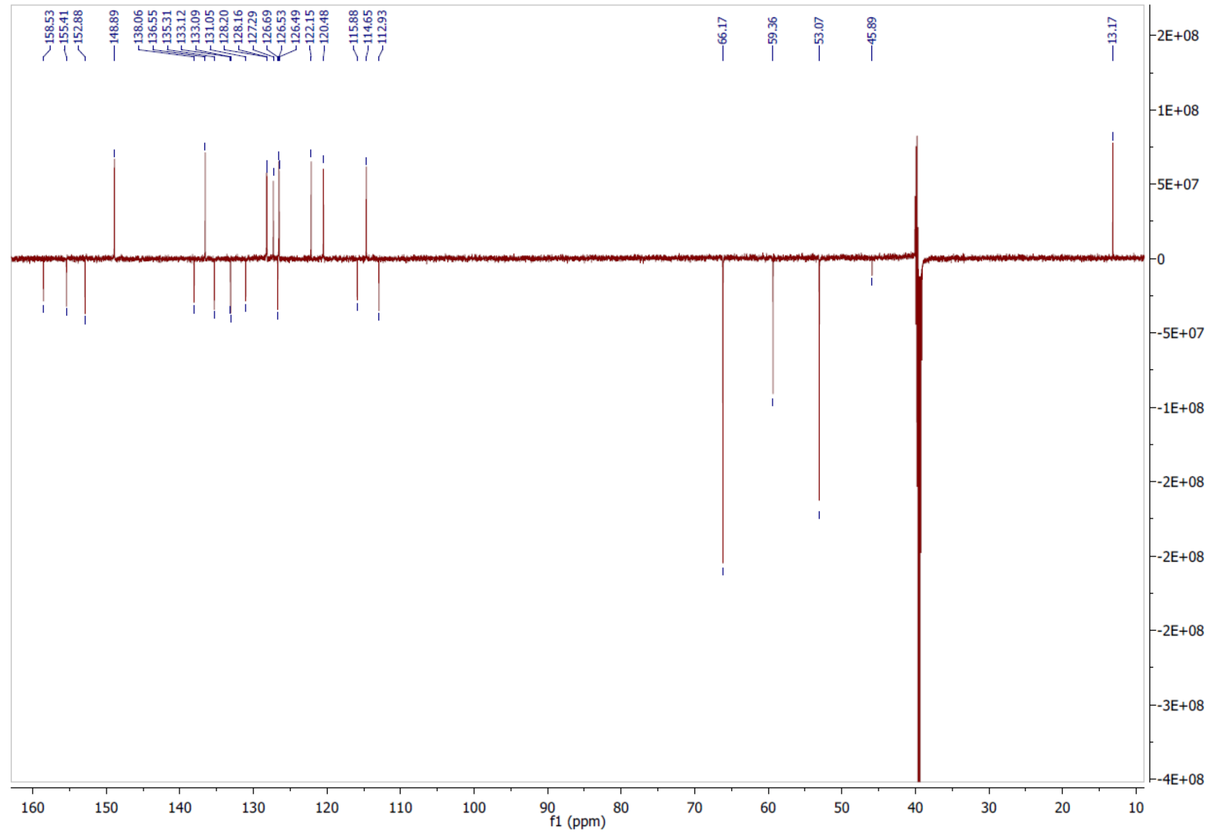

Figure S42. ^{13}C NMR spectrum of **HL^4**.

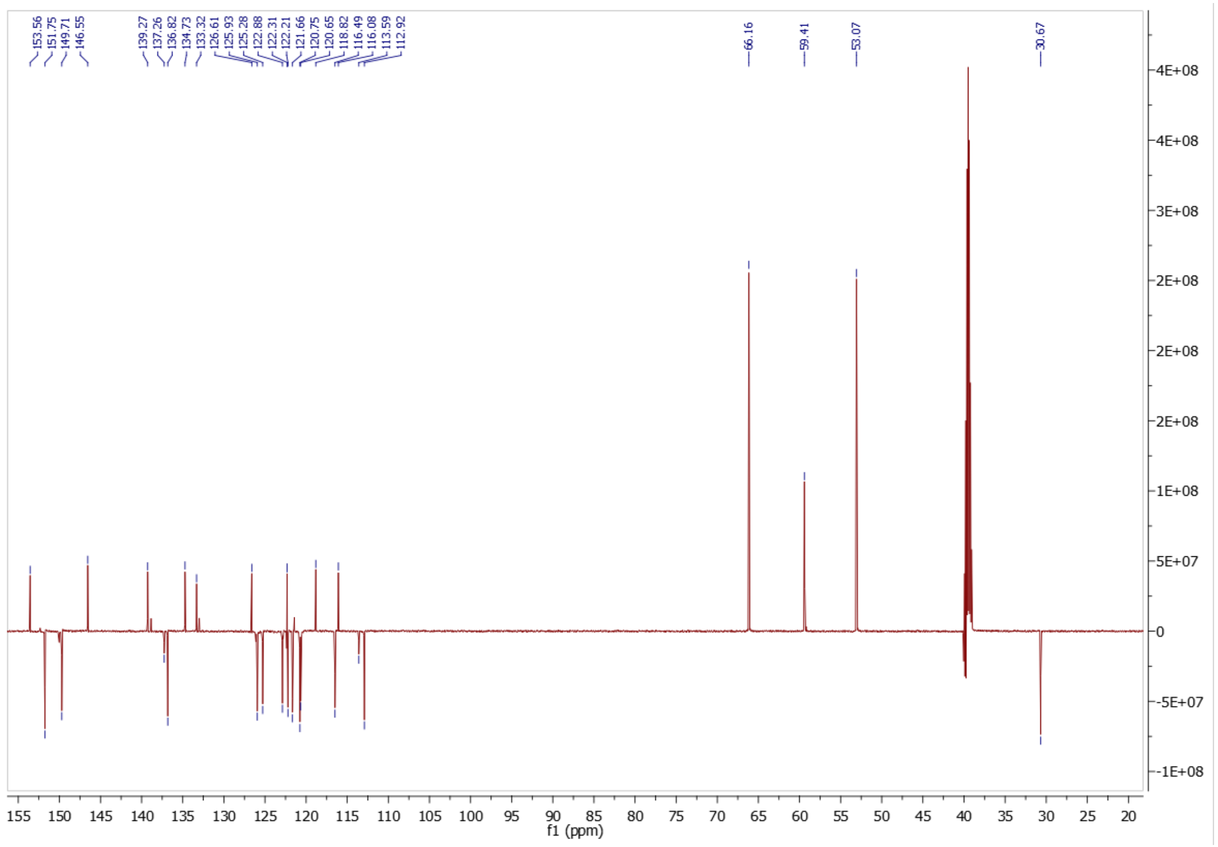

Figure S43. ^{13}C NMR spectrum of **HL^{5}**.

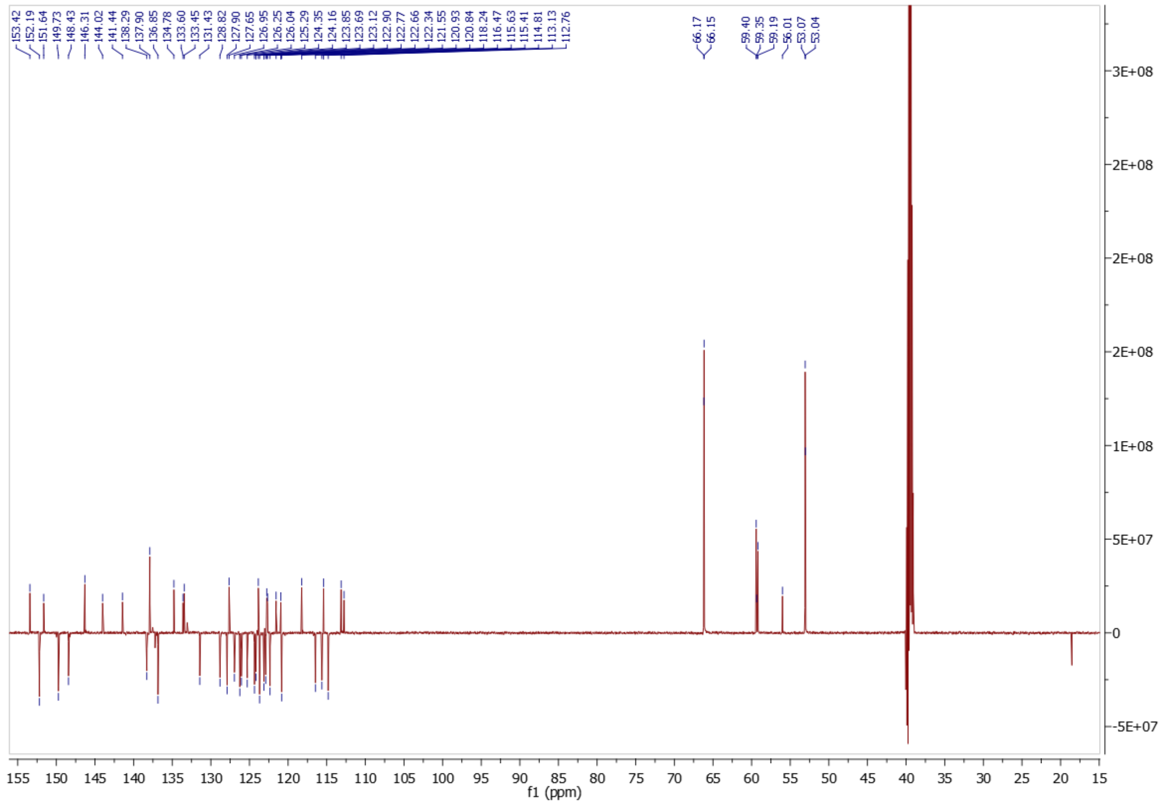

Figure S44. ^{13}C NMR spectrum of **HL^{6}**.

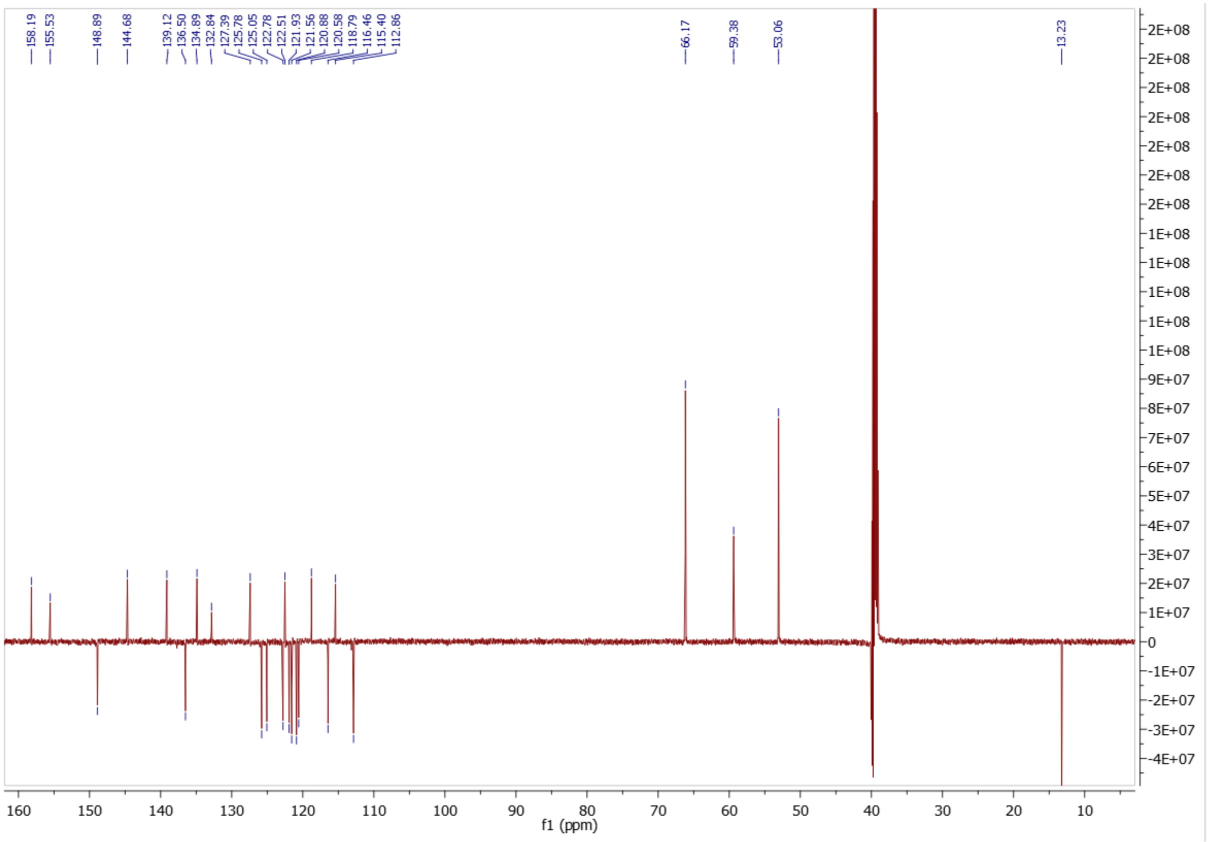

Figure S45. ^{13}C NMR spectrum of **HL^{7}**.

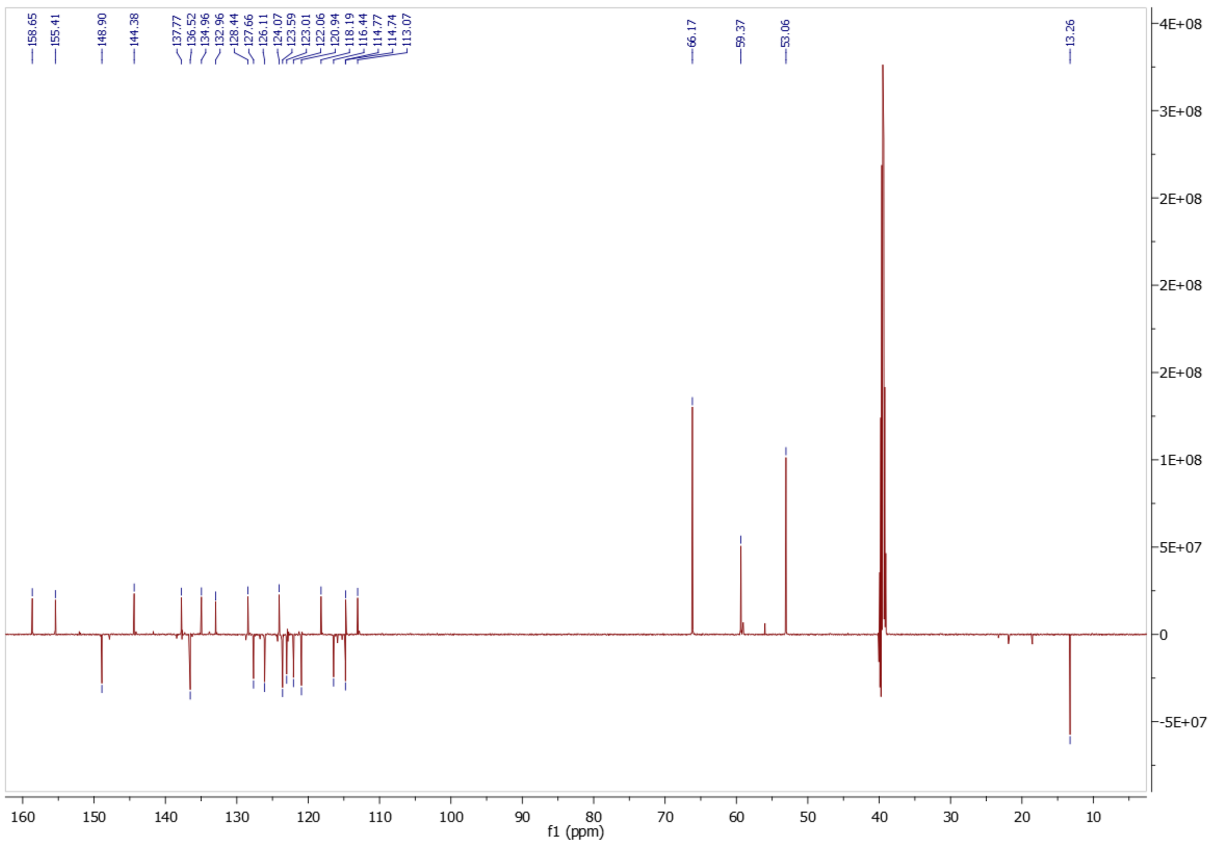

Figure S46. ^{13}C NMR spectrum of **HL^{8}**.

- **ESI Mass spectra**

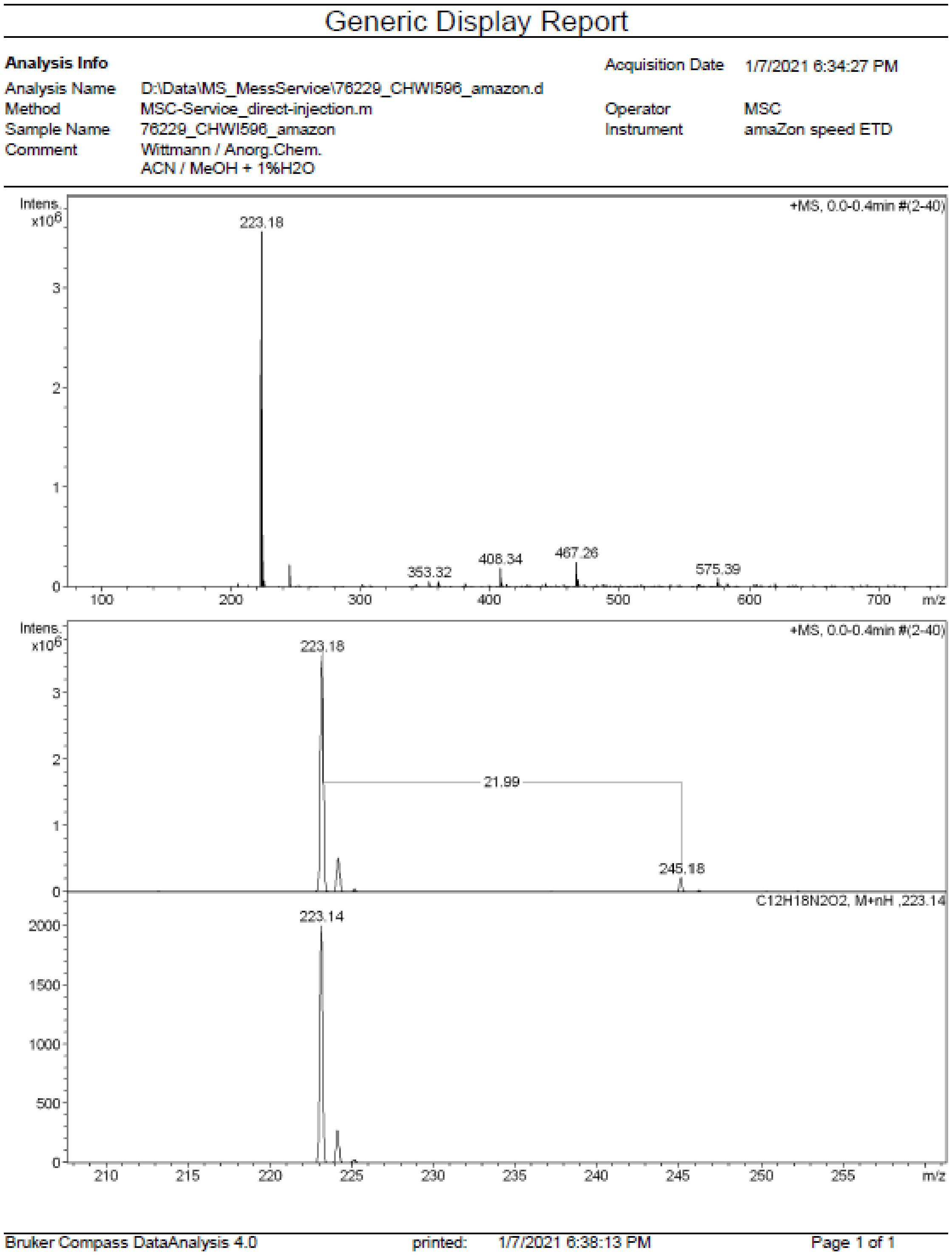

Figure S47. ESI mass spectrum of **I** in positive mode.

Generic Display Report

**Analysis Info**		Acquisition Date	1/7/2021 5:32:15 PM
Analysis Name	D:\Data\MS_MessService\76222_CHWI363_amazon.d		
Method	MSC-Service_direct-injection.m	Operator	MSC
Sample Name	76222_CHWI363_amazon	Instrument	amaZon speed ETD
Comment	Wittmann / Anorg.Chem.<br>ACN / MeOH + 1%H2O		

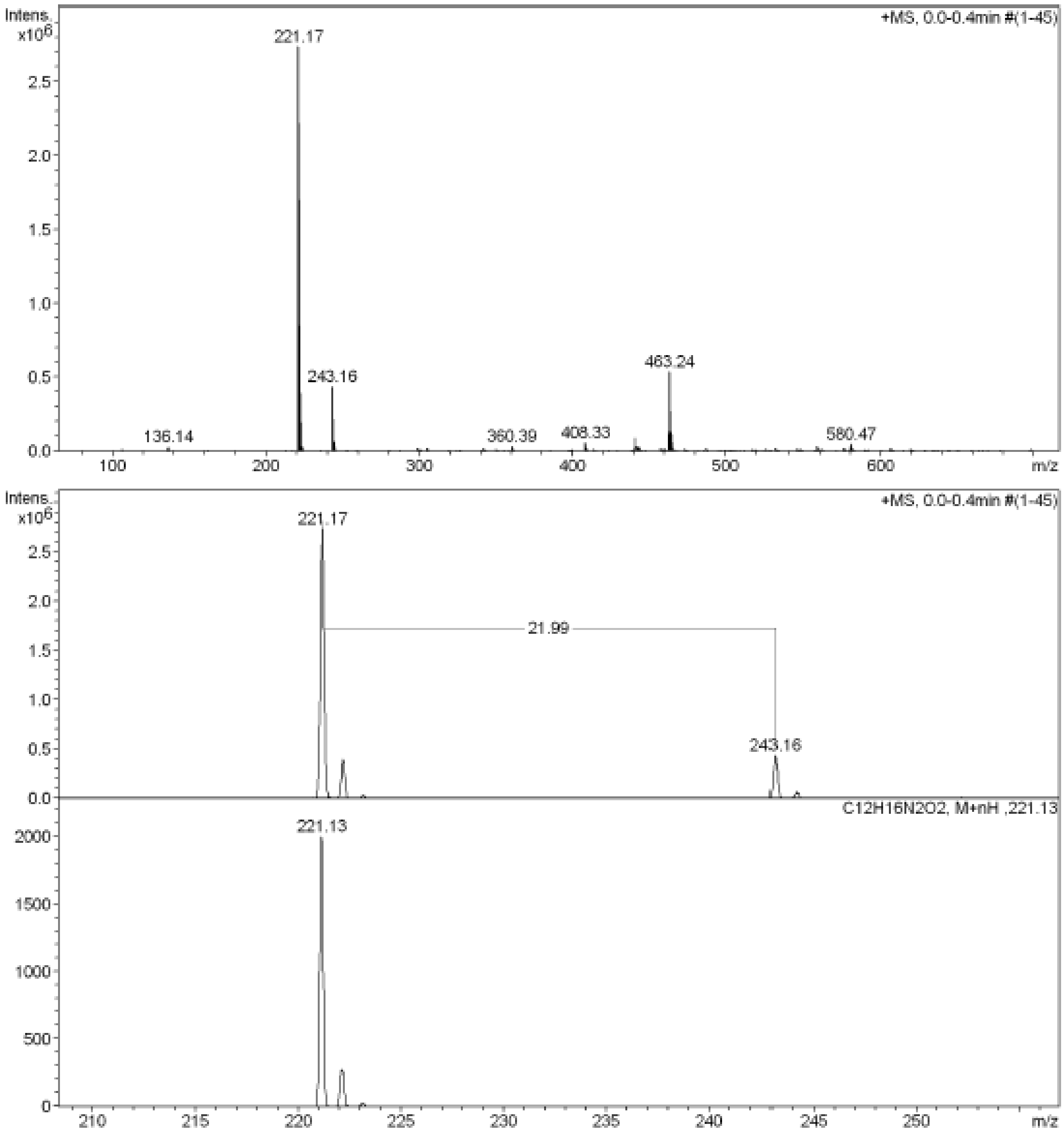

Bruker Compass DataAnalysis 4.0 printed: 1/7/2021 5:35:44 PM Page 1 of 1

Figure S48. ESI mass spectrum of **J** in positive ion mode.

Generic Display Report

Analysis Info

Analysis Name	D:\Data\MS_MessService\76336_CHWI598_amazon	Acquisition Date	1/13/2021 3:57:54 PM
Method	MSC-Service_direct-injection.m	Operator	MSC
Sample Name	76336_CHWI598_amazon	Instrument	amaZon speed ETD
Comment	Wittmann / AOC<br>ACN / MeOH + 1%H2O		

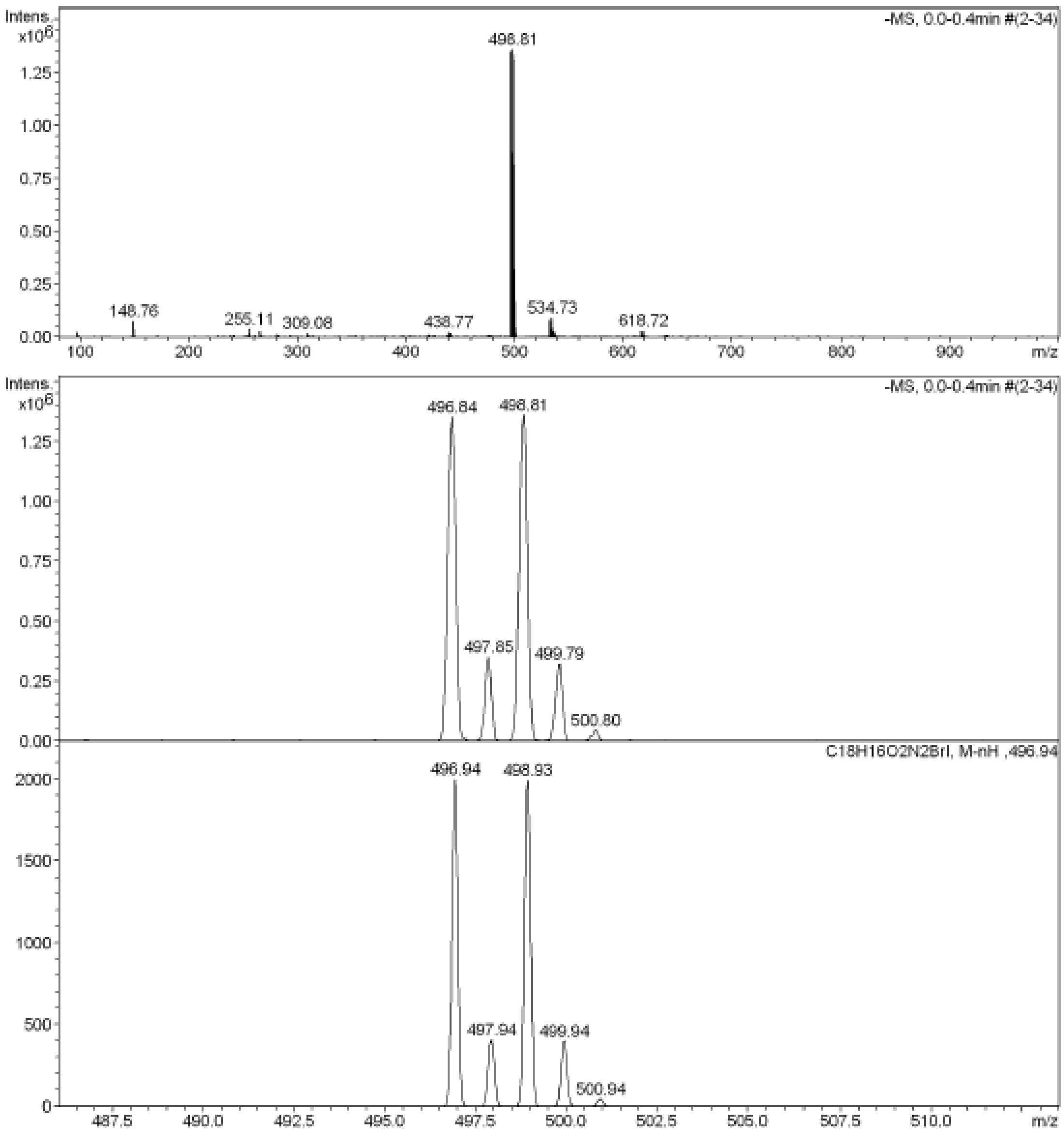

Bruker Compass DataAnalysis 4.0 printed: 1/13/2021 4:02:36 PM Page 1 of 1

Figure S49. ESI mass spectrum of **Ib** in negative ion mode.

Generic Display Report

Analysis Info		Acquisition Date	1/13/2021 4:10:23 PM
Analysis Name	D:\Data\MS_MessService\76337_CHWI599_amazon.d		
Method	MSC-Service_direct-injection.m	Operator	MSC
Sample Name	76337_CHWI599_amazon	Instrument	amaZon speed ETD
Comment	Wittmann / AOC		
	ACN / MeOH + 1%H2O		

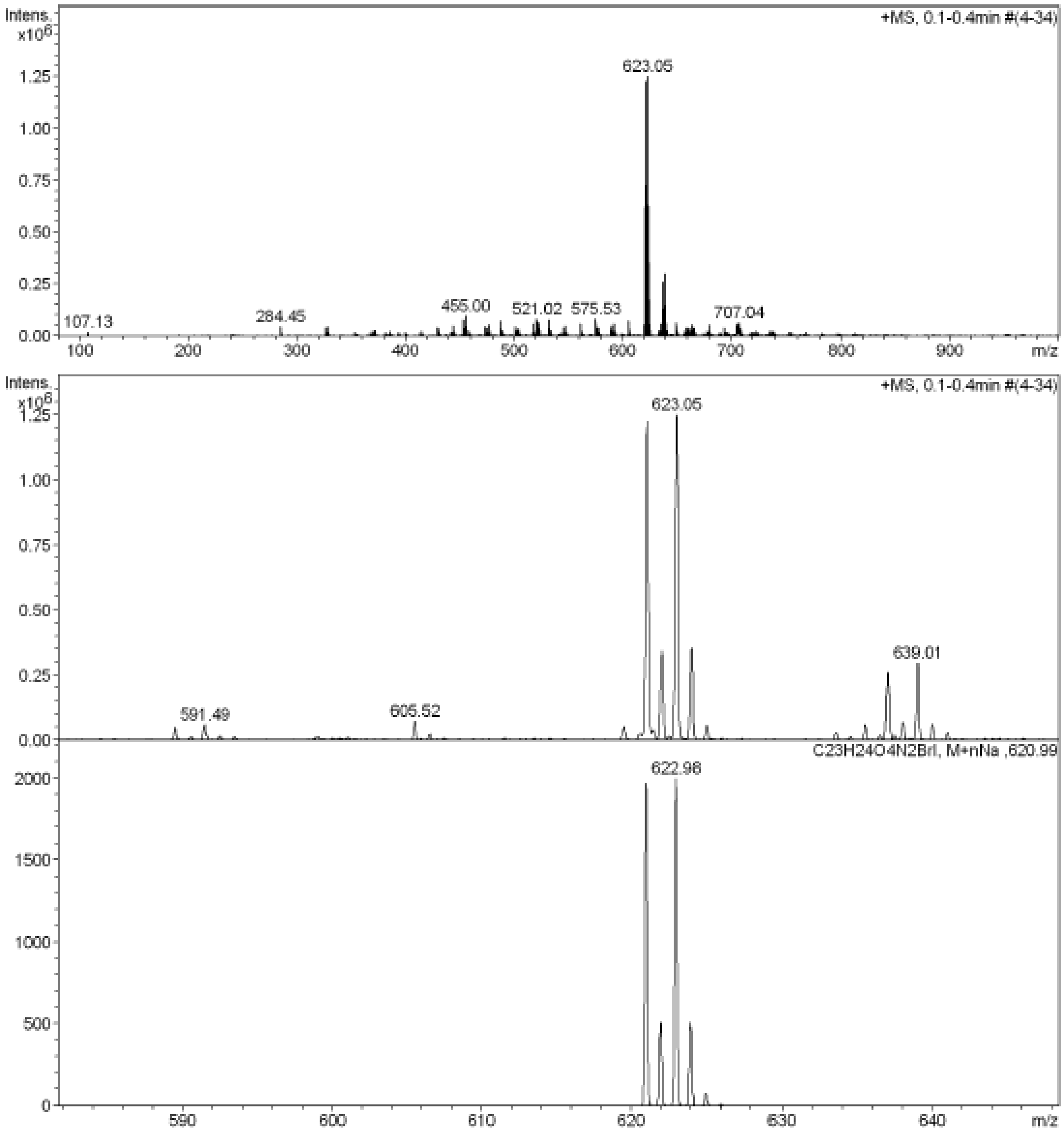

Bruker Compass DataAnalysis 4.0 printed: 1/13/2021 4:15:27 PM Page 1 of 1

Figure S50. ESI mass spectrum of **IIb** in positive ion mode.

Generic Display Report

Analysis Info

Analysis Name	D:\Data\MS_MessService\76383_CHWI603_amazon.d	Acquisition Date	1/15/2021 11:21:50 AM
Method	MSC-Service_direct-injection.m	Operator	MSC
Sample Name	76383_CHWI603_amazon	Instrument	amaZon speed ETD
Comment	Wittmann / AOC<br>ACN / MeOH + 1%H2O		

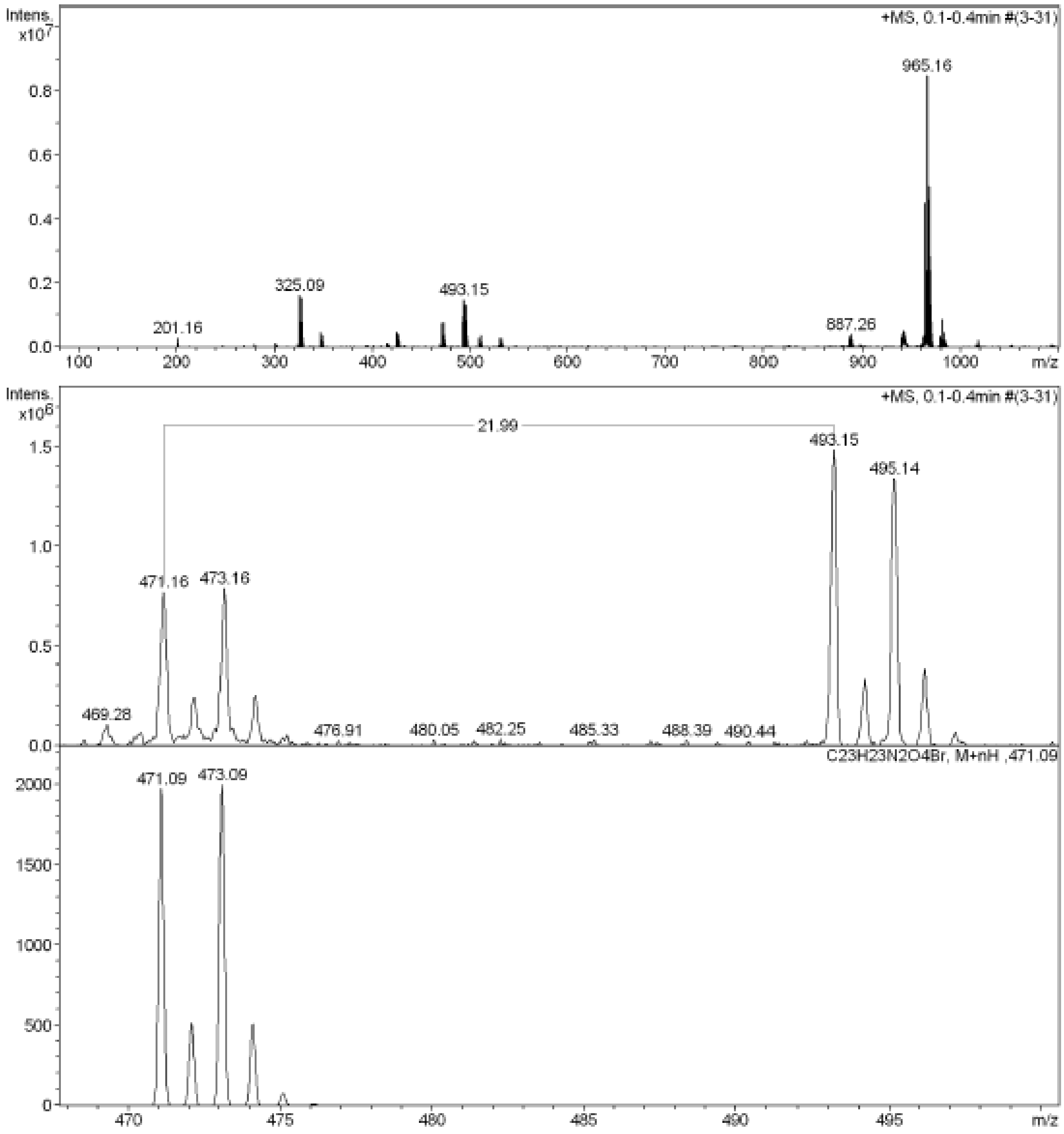

Bruker Compass DataAnalysis 4.0 printed: 1/15/2021 11:39:59 AM Page 1 of 1

Figure S51. ESI mass spectrum of **IIIb** in positive ion mode.

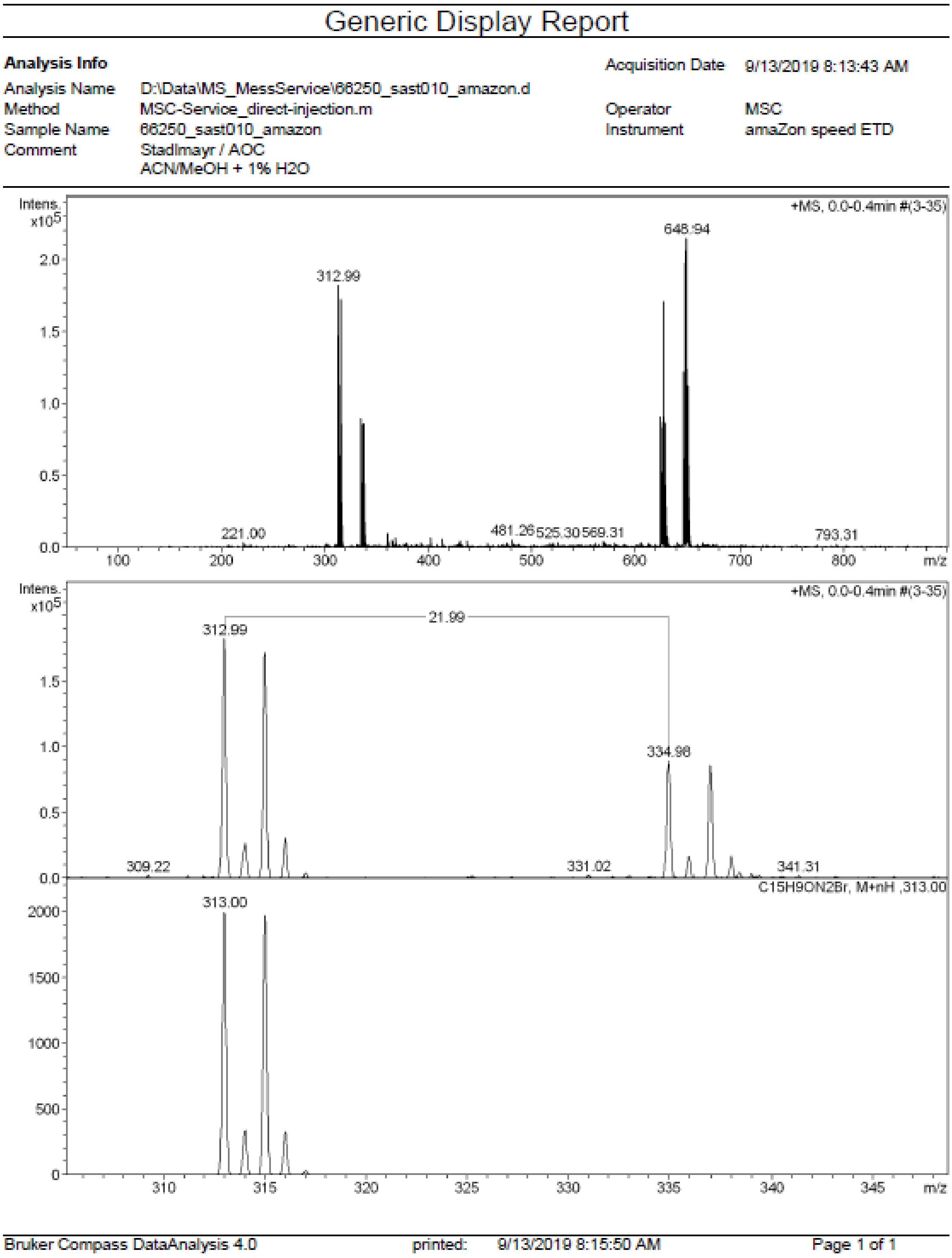

Figure S52. ESI mass spectrum of **IVb** in positive ion mode.

Generic Display Report

Analysis Info

Analysis Name	D:\Data\MS_MessService\61947_feba590_amazon.d	Acquisition Date	30.01.2019 08:40:08
Method	MSC-Service_direct-injection.m	Operator	MSC
Sample Name	61947_feba590_amazon	Instrument	amaZon speed ETD
Comment	Bacher / Anorg.Chem. ACN / MeOH + 1% H2O		

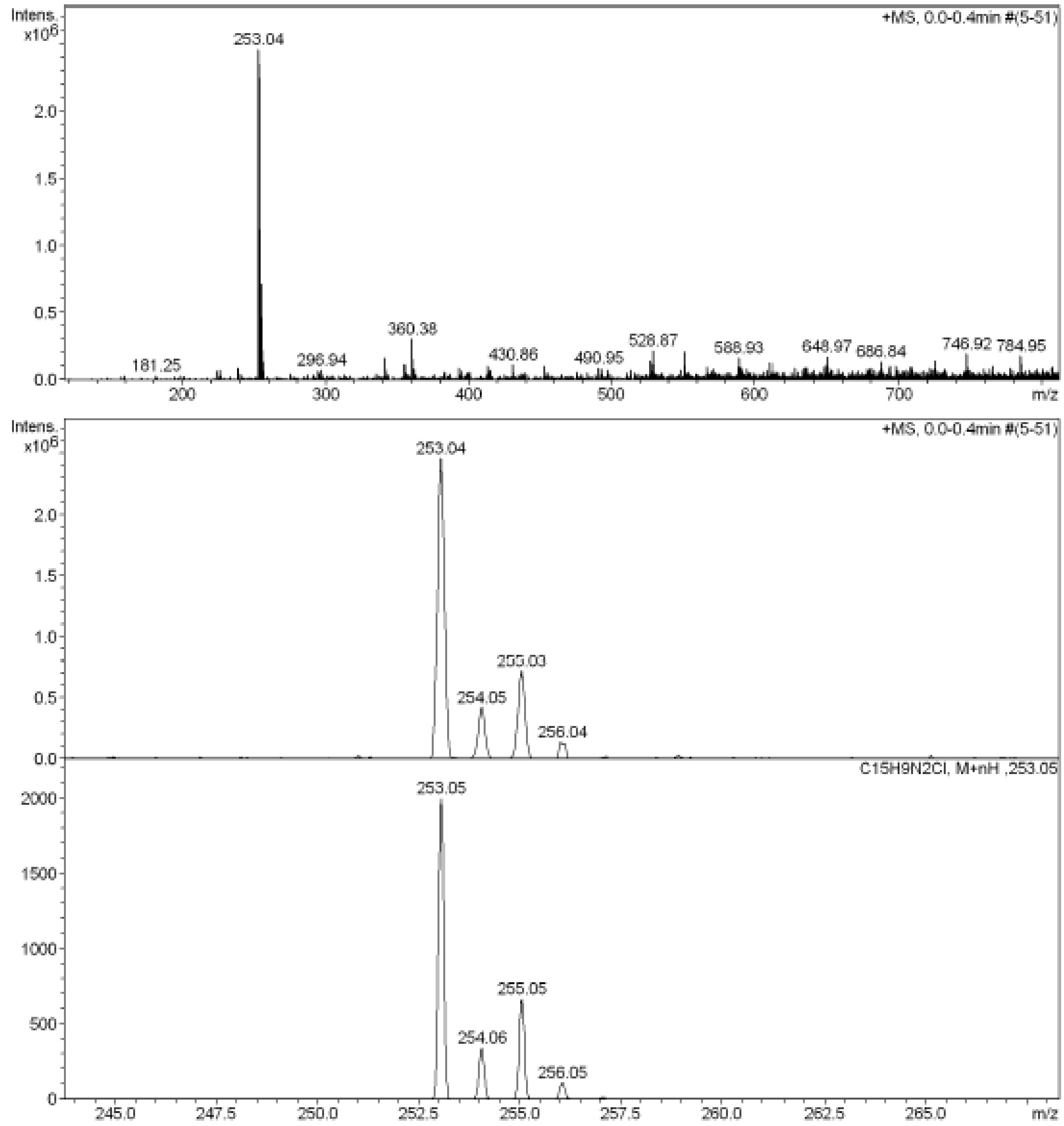

Bruker Compass DataAnalysis 4.0 printed: 30.01.2019 08:42:55 Page 1 of 1

Figure S53. ESI mass spectrum of **Va** in positive ion mode.

Generic Display Report

**Analysis Info**		Acquisition Date	10/2/2019 2:09:07 PM
Analysis Name	D:\Data\MS_MessService\66562_feba644_amazon.d		
Method	MSC-Service_direct-injection.m	Operator	MSC
Sample Name	66562_feba644_amazon	Instrument	amaZon speed ETD
Comment	Bacher / AOC<br>ACN / MeOH + 1% H2O		

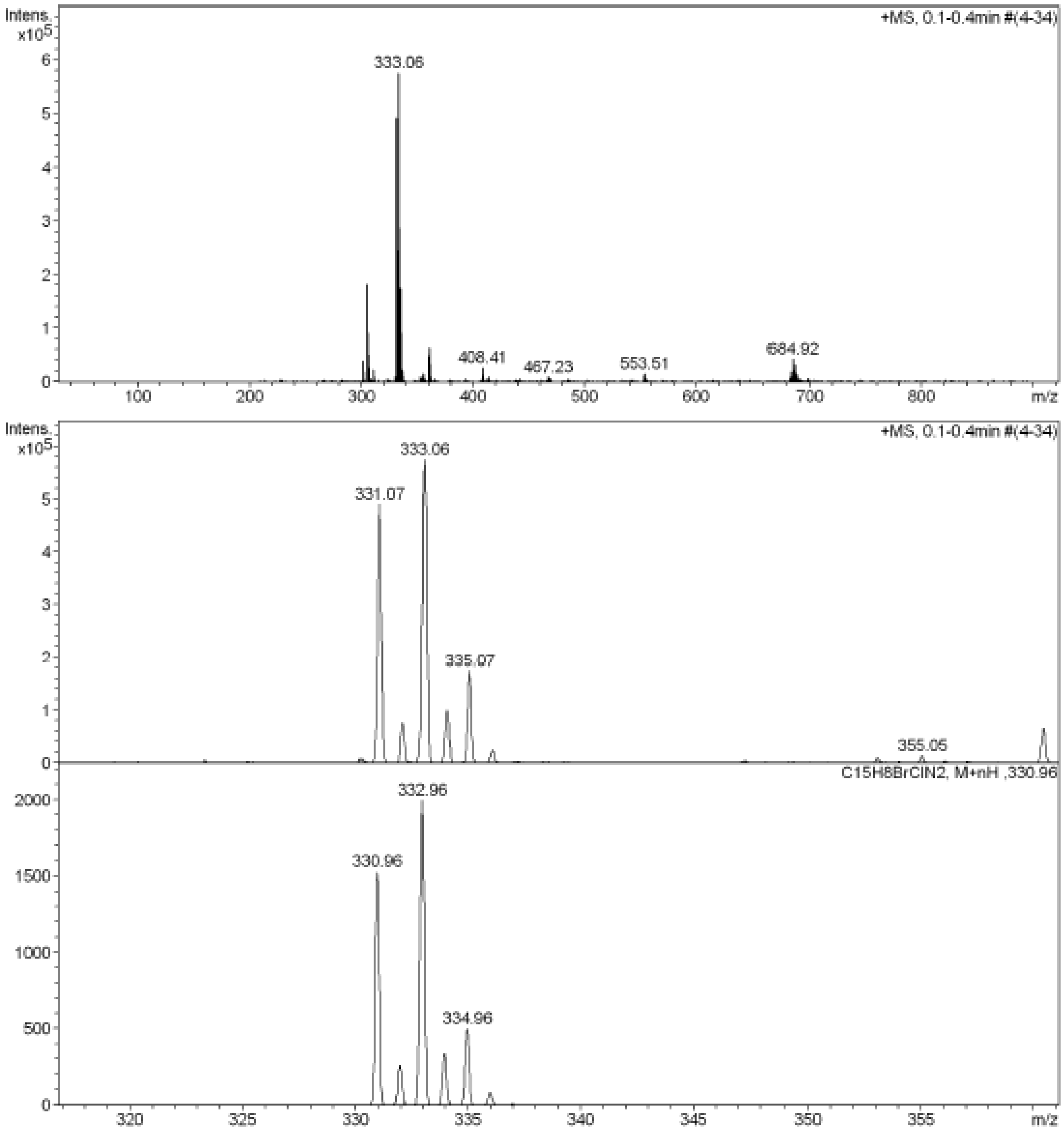

Bruker Compass DataAnalysis 4.0 printed: 10/2/2019 2:11:05 PM Page 1 of 1

Figure S54. ESI mass spectrum of **Vb** in positive ion mode.

Generic Display Report

Analysis Info

Analysis Name	D:\Data\MS_MessService\83182_feba604_amazon.d	Acquisition Date	8/31/2021 11:49:22 AM
Method	MSC-Service_direct-injection.m	Operator	MSC
Sample Name	83182_feba604_amazon	Instrument	amaZon speed ETD
Comment	Wittmann / Anorg. Chem.		
	ACN / MeOH + 1% H2O		

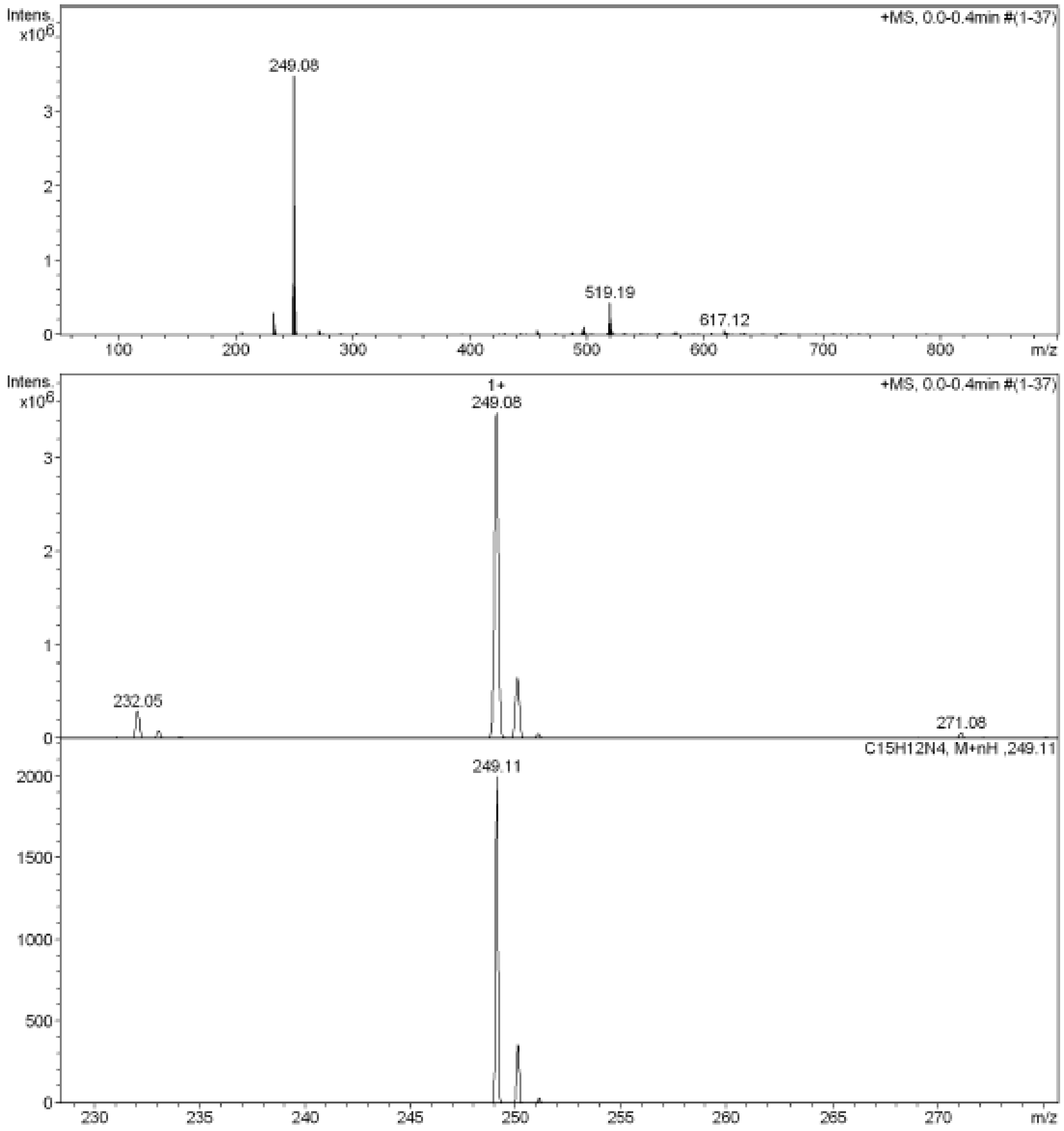

Bruker Compass DataAnalysis 4.0 printed: 8/31/2021 11:51:25 AM Page 1 of 1

Figure S55. ESI mass spectrum of **M** in positive ion mode.

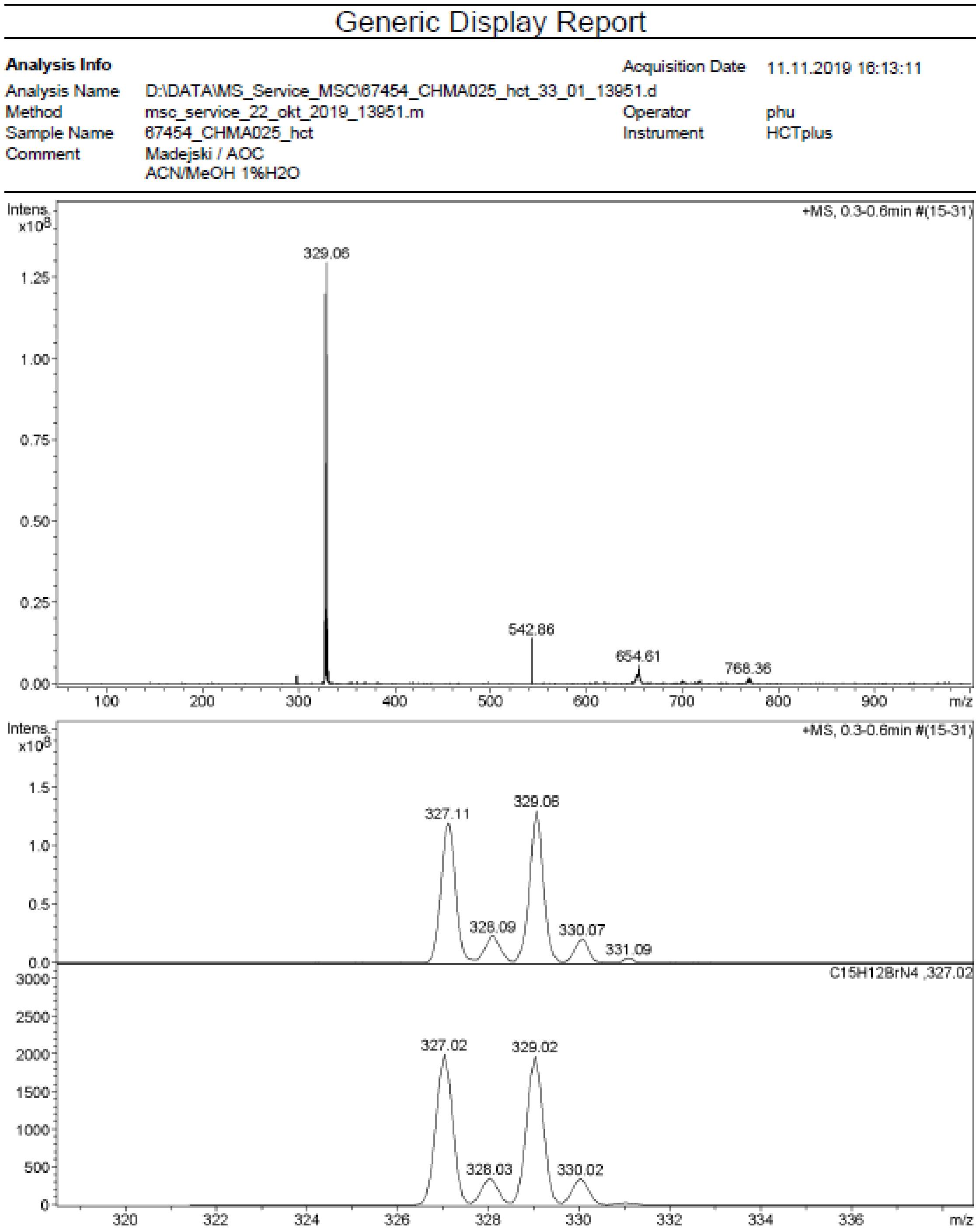

Figure S56. ESI mass spectrum of **N** in positive ion mode.

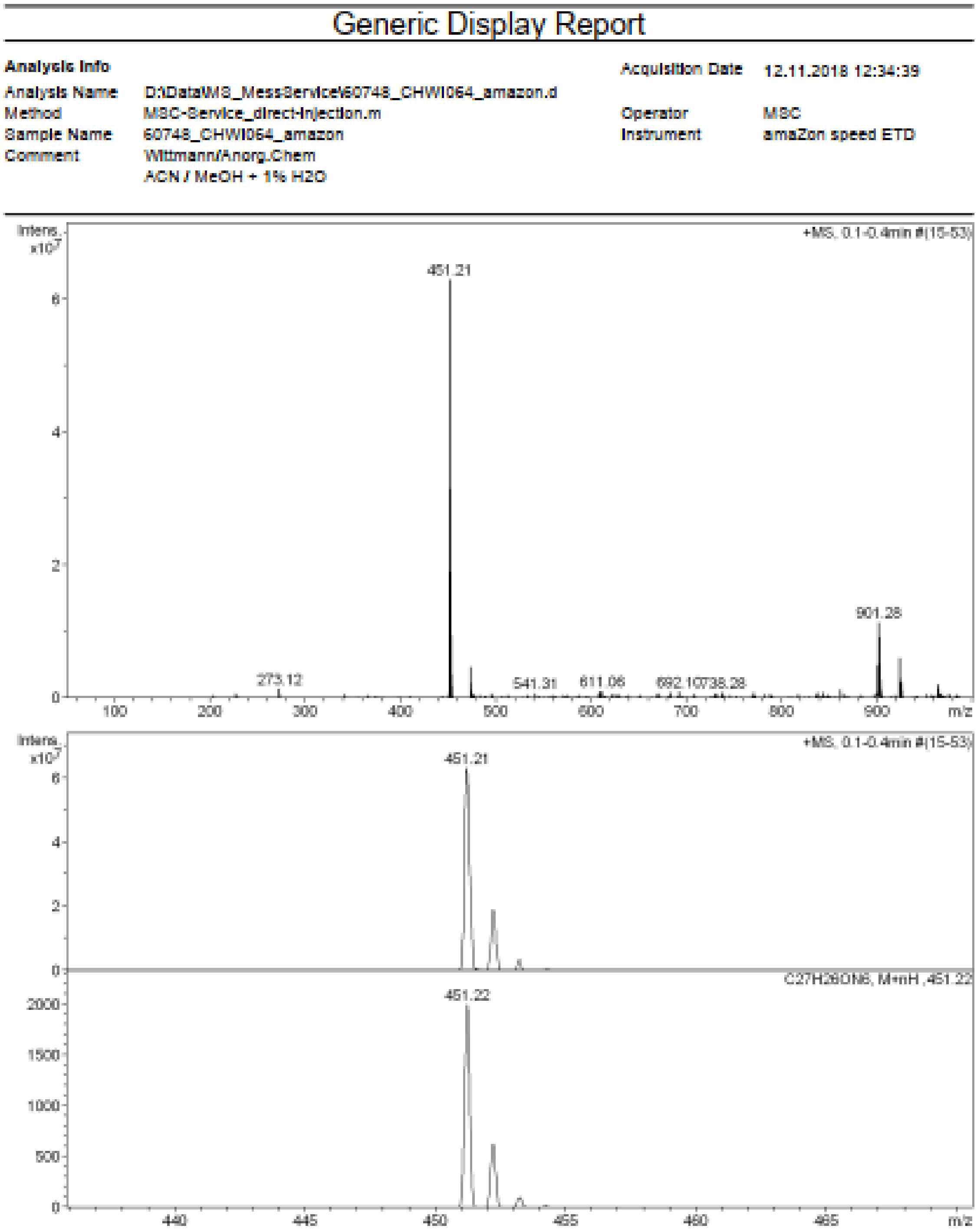

Figure S57. ESI mass spectrum of **HL¹** in positive ion mode.

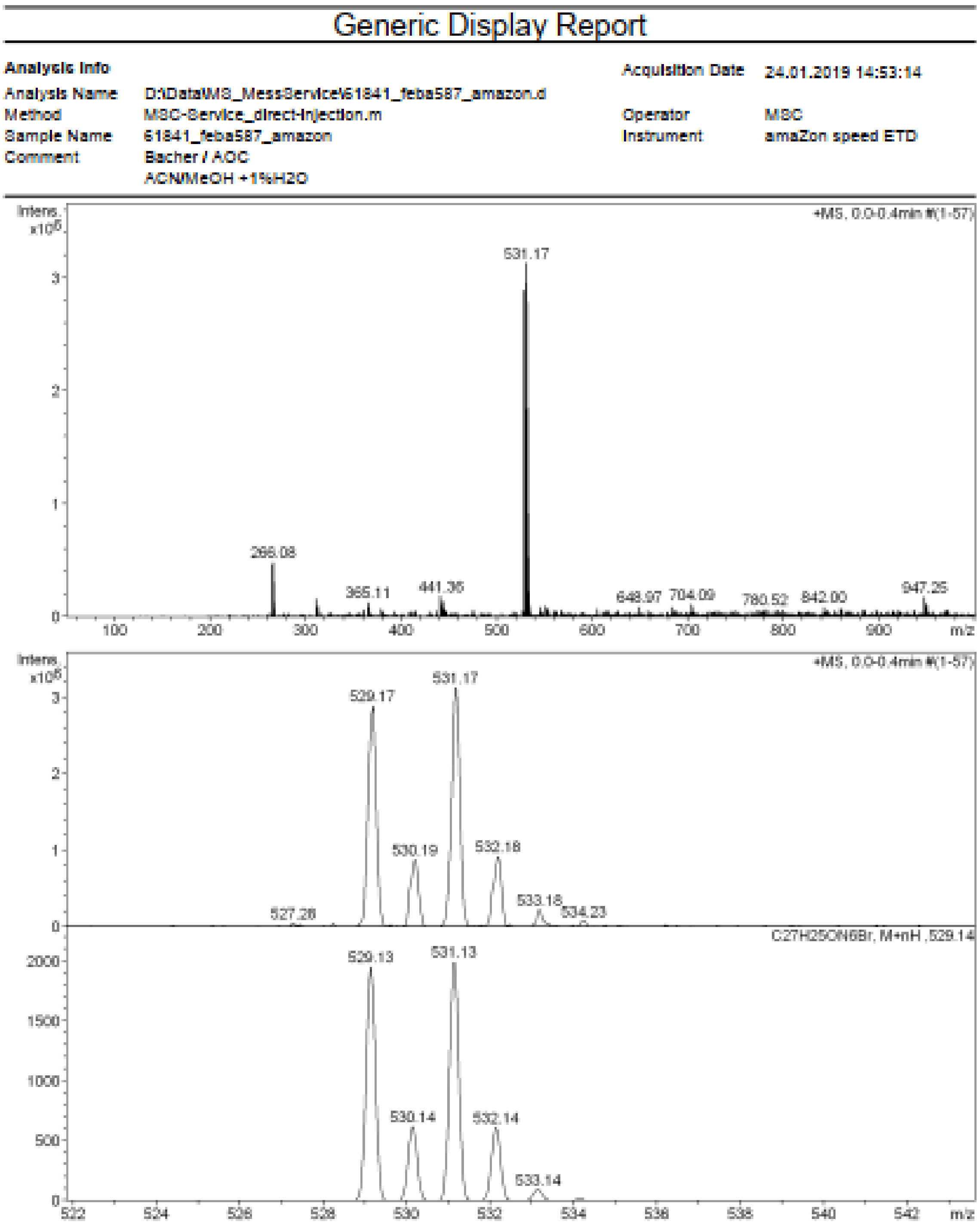

Figure S58. ESI mass spectrum of **HL^2** in positive ion mode.

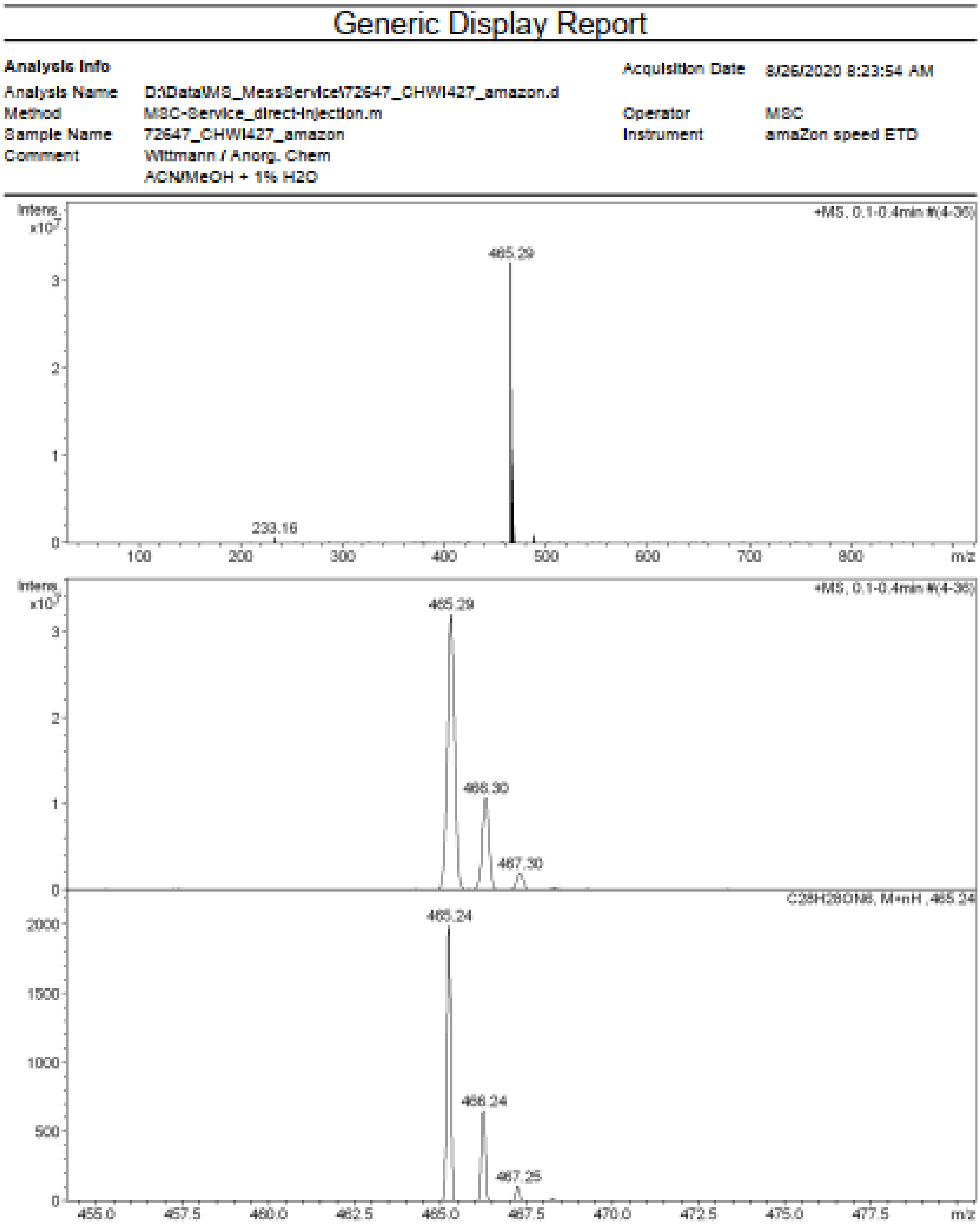

Figure S59. ESI mass spectrum of **HL^3** in positive ion mode.

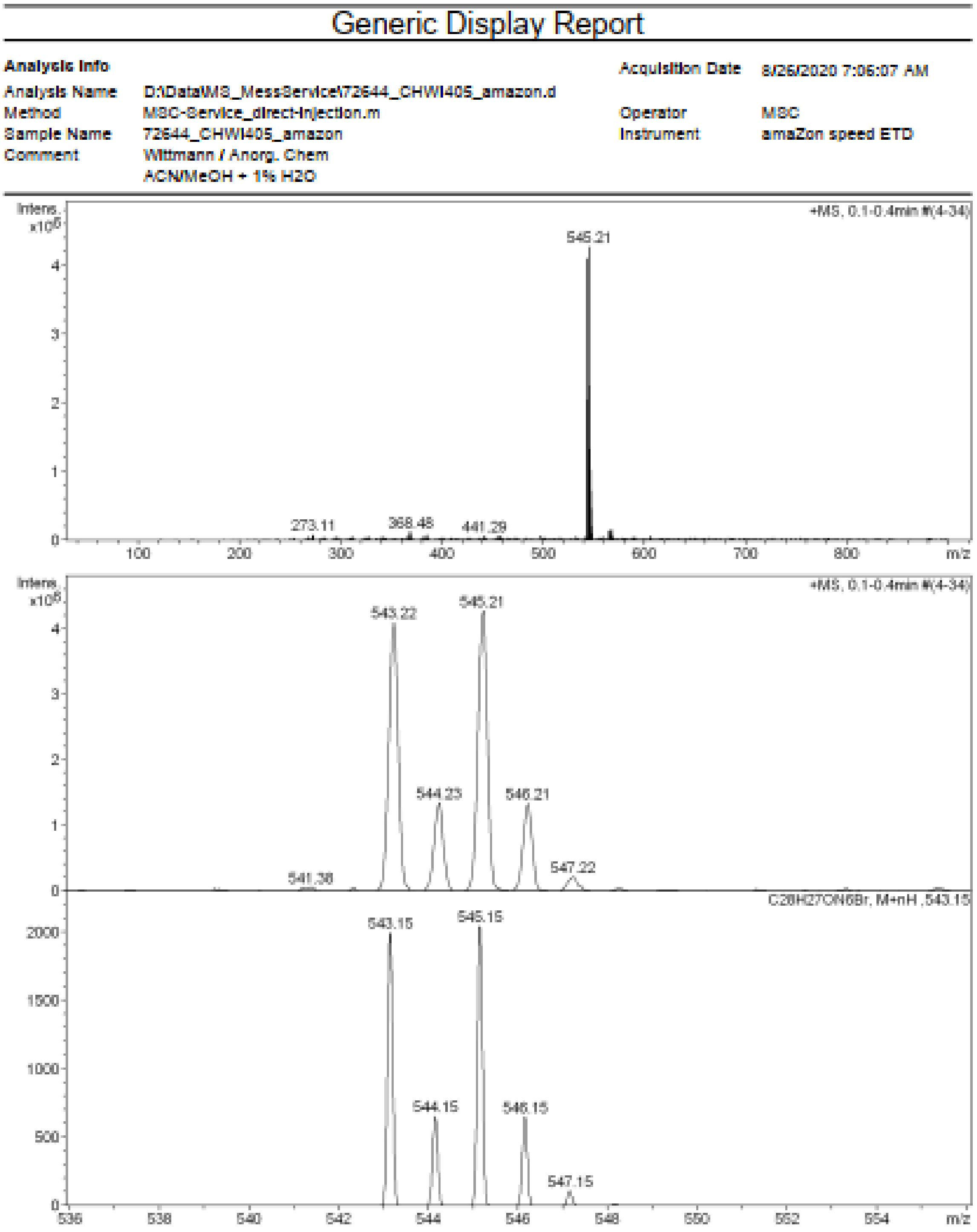

Figure S60. ESI mass spectrum of $\mathbf{HL^4}$ in positive ion mode.

Generic Display Report

Analysis Info

Analysis Name	D:\Data\MS_MessService\62048_feba594_amazon.d	Acquisition Date	05.02.2019 13:38:54
Method	MSC-Service_direct-injection.m	Operator	MSC
Sample Name	62048_feba594_amazon	Instrument	amaZon speed ETD
Comment	Bacherr / Anorg.Chem.<br>ACN/MeOH + 1% H2O		

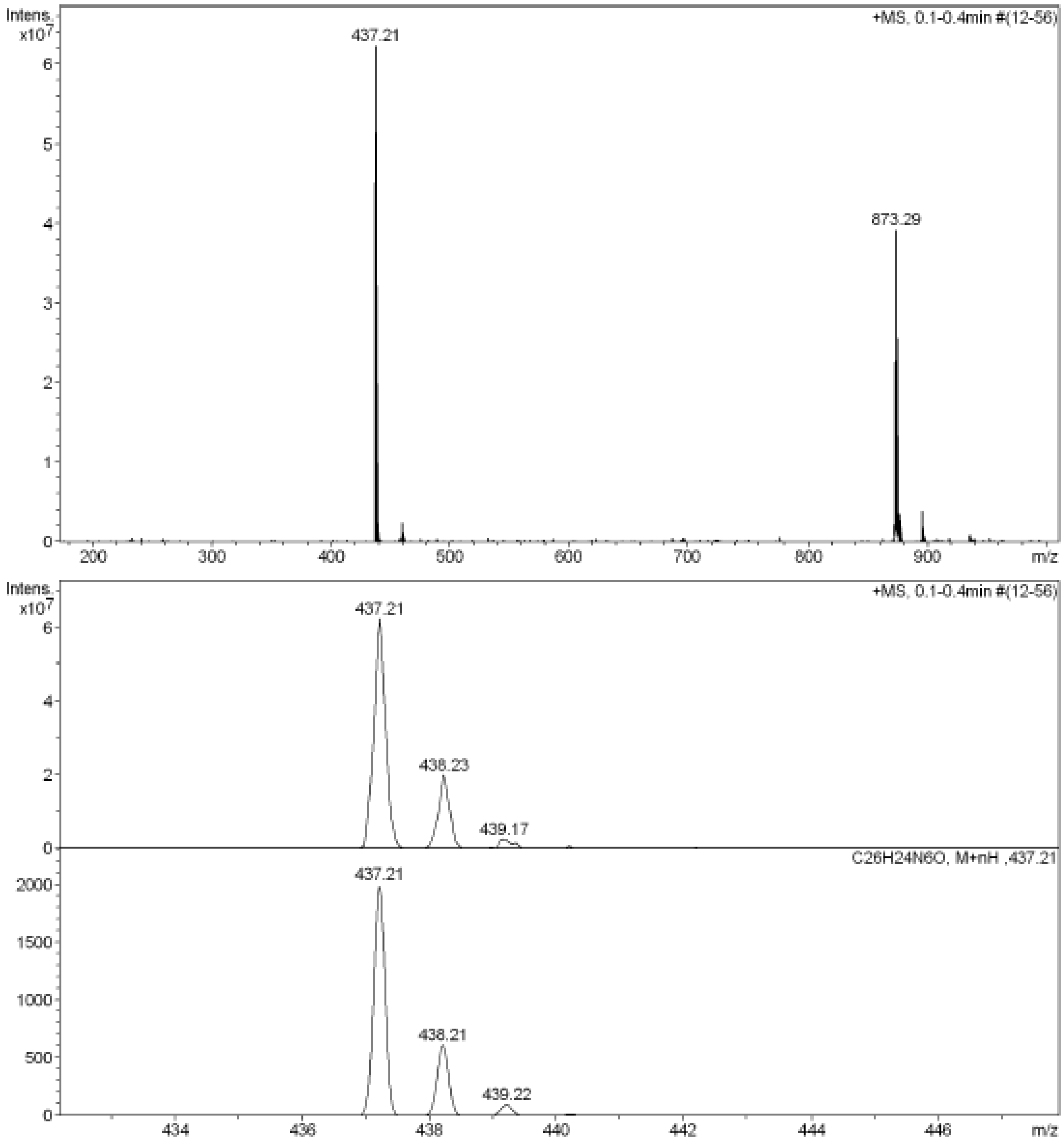

Bruker Compass DataAnalysis 4.0 printed: 05.02.2019 13:46:51 Page 1 of 1

Figure S61. ESI mass spectrum of **HL**^{5} in positive ion mode.

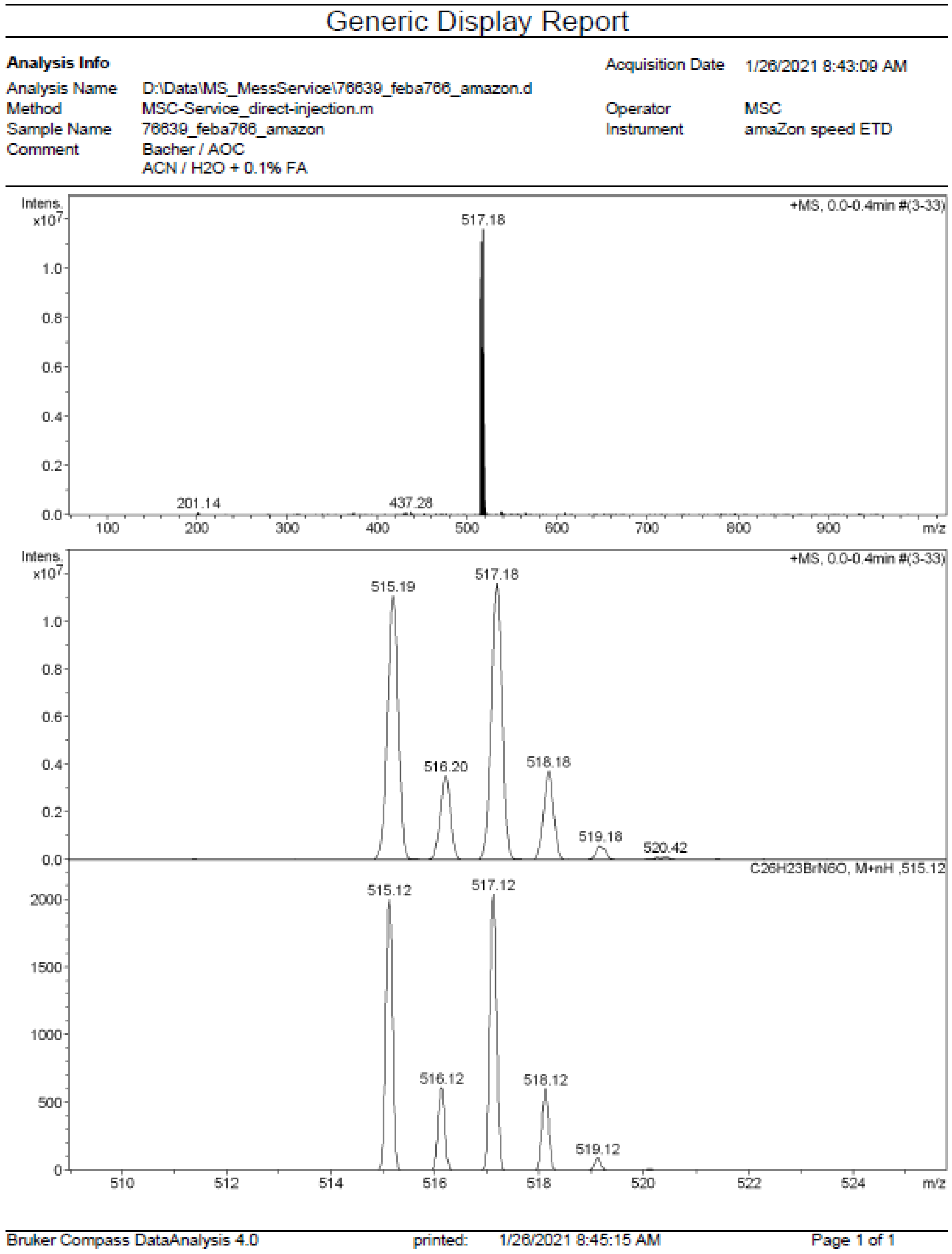

Figure S62. ESI mass spectrum of **HL⁶** in positive ion mode.

Generic Display Report

Analysis Info			
Analysis Name	D:\Data\MS_MessService\76221_febaHL5_amazon.d	Acquisition Date	1/7/2021 3:47:27 PM
Method	MSC-Service_direct-injection.m	Operator	MSC
Sample Name	76221_febaHL5_amazon	Instrument	amaZon speed ETD
Comment	Wittmann / Anorg.Chem.<br>ACN / MeOH + 1%H2O		

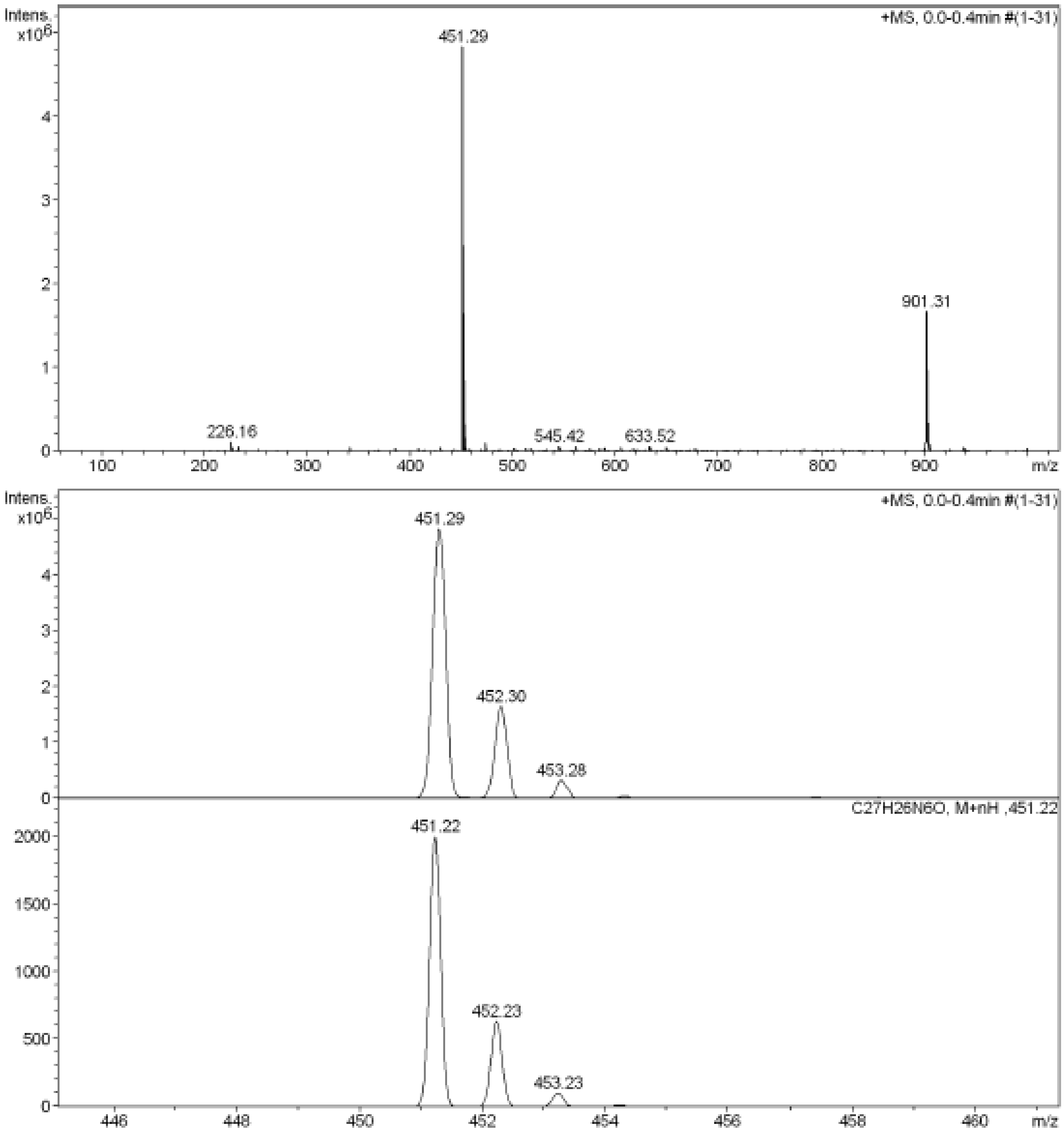

Bruker Compass DataAnalysis 4.0 printed: 1/7/2021 3:49:38 PM Page 1 of 1

Figure S63. ESI mass spectrum of **HL**[7] in positive ion mode.

Generic Display Report

Analysis Info		Acquisition Date	1/26/2021 9:32:10 AM
Analysis Name	D:\Data\MS_MessService\76640_feba767_amazon.d		
Method	MSC-Service_direct-injection.m	Operator	MSC
Sample Name	76640_feba767_amazon	Instrument	amaZon speed ETD
Comment	Bacher / AOC<br>ACN / H2O + 0.1% FA		

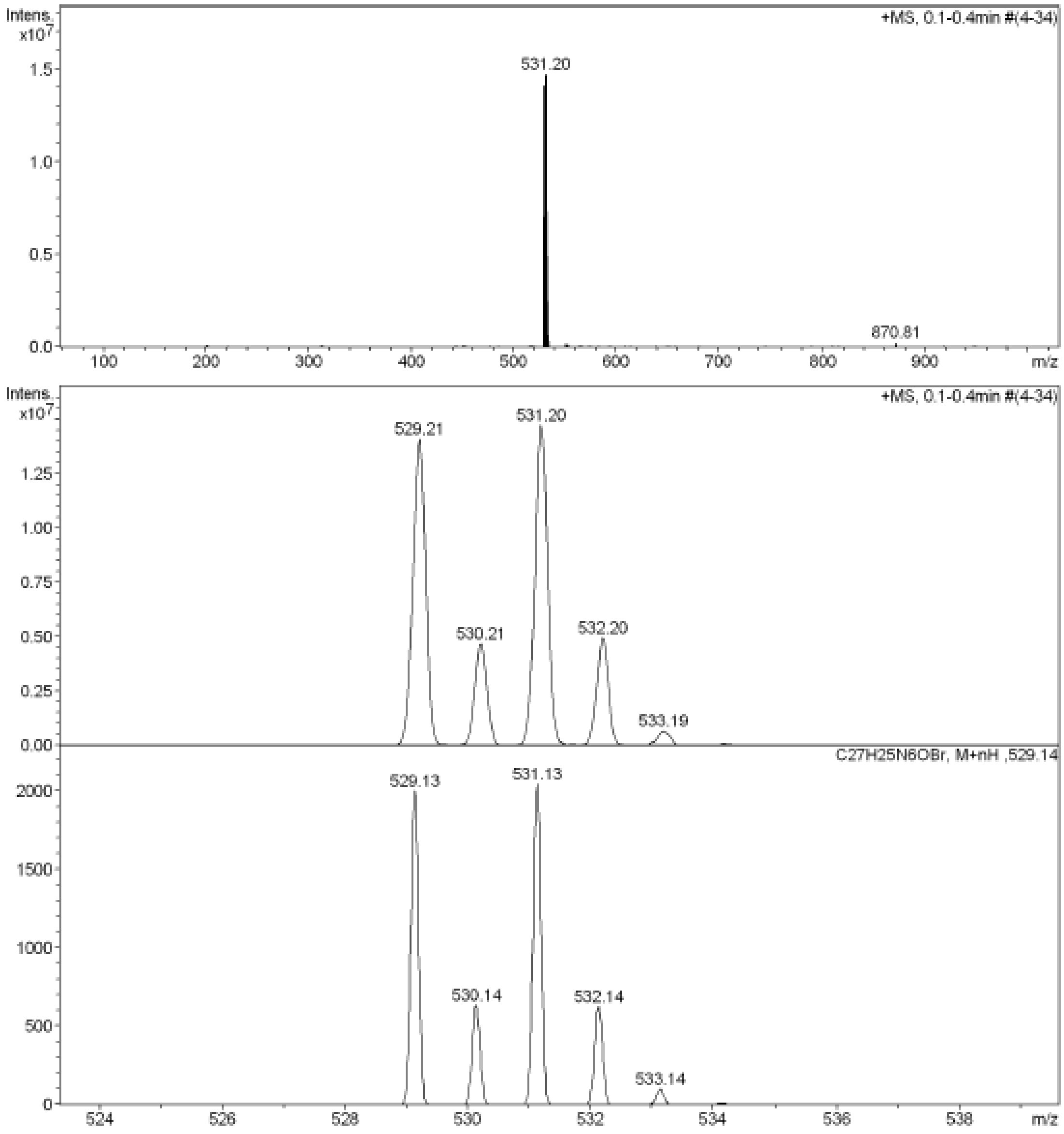

Bruker Compass DataAnalysis 4.0 printed: 1/26/2021 9:34:19 AM Page 1 of 1

Figure S64. ESI mass spectrum of **HL^8** in positive ion mode.

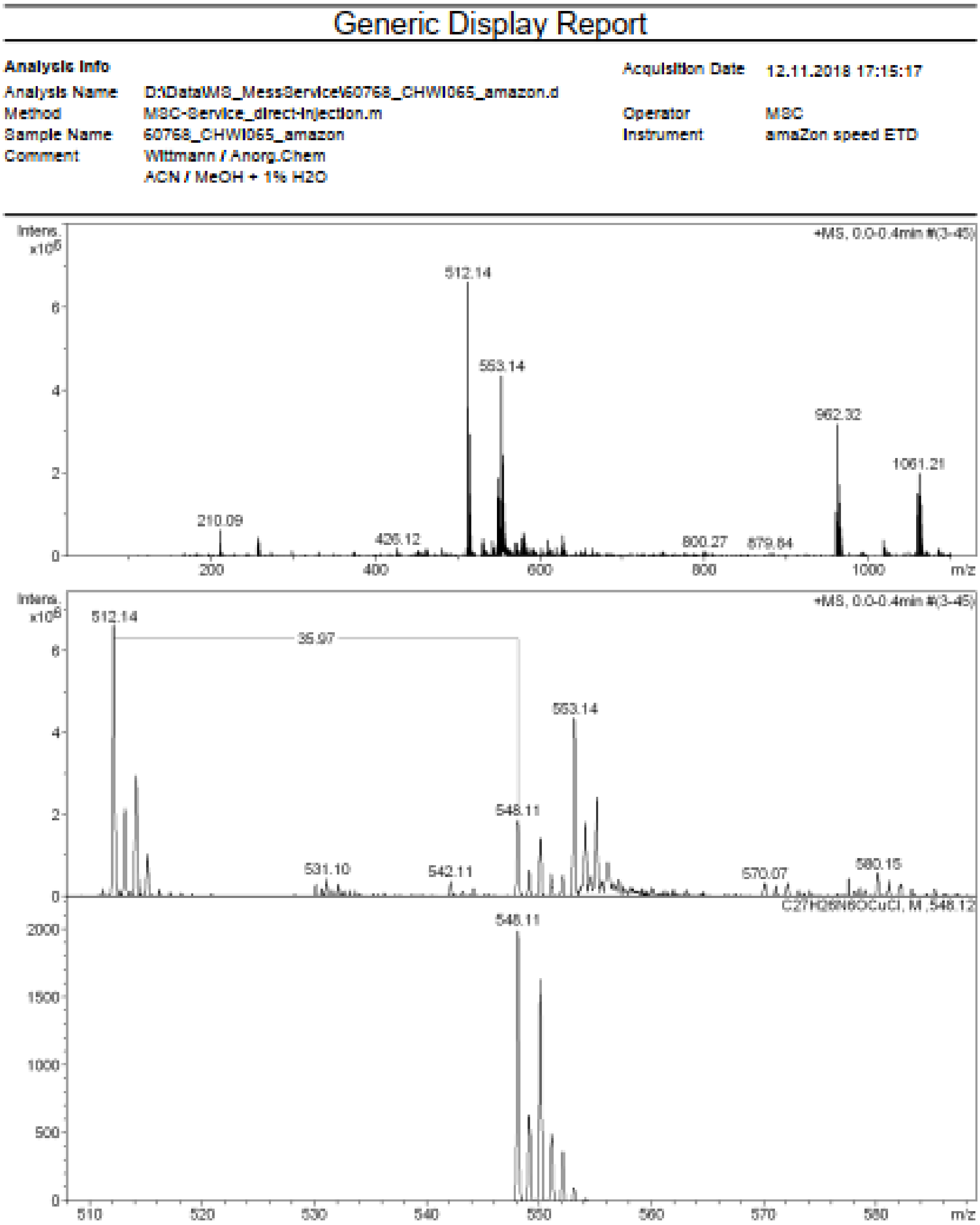

Figure S65. ESI mass spectrum of **1** in positive ion mode.

Generic Display Report

**Analysis Info**		Acquisition Date	1/24/2019 3:28:16 PM
Analysis Name	D:\Data\MS_Service_MSC_Archiv_2019_616_682\618\61843_feba589_amazon.d		
Method	MSC-Service_direct-injection.m	Operator	MSC
Sample Name	61843_feba589_amazon	Instrument	amaZon speed ETD
Comment	Bacher / AOC<br>ACN/MeOH +1%H2O		

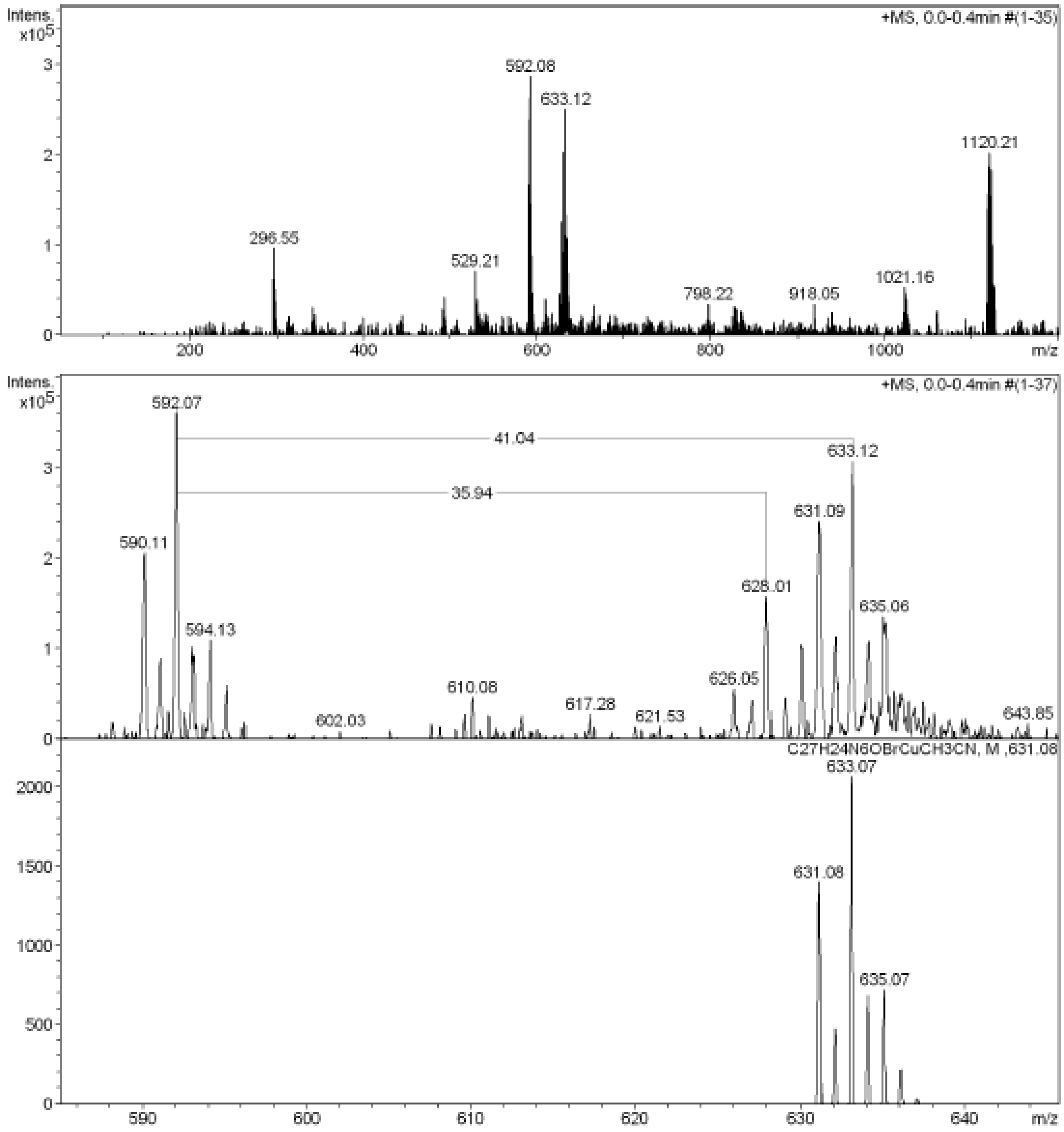

Bruker Compass DataAnalysis 4.0 printed: 9/1/2021 9:41:50 AM Page 1 of 1

Figure S66. ESI mass spectrum of **2** in positive ion mode.

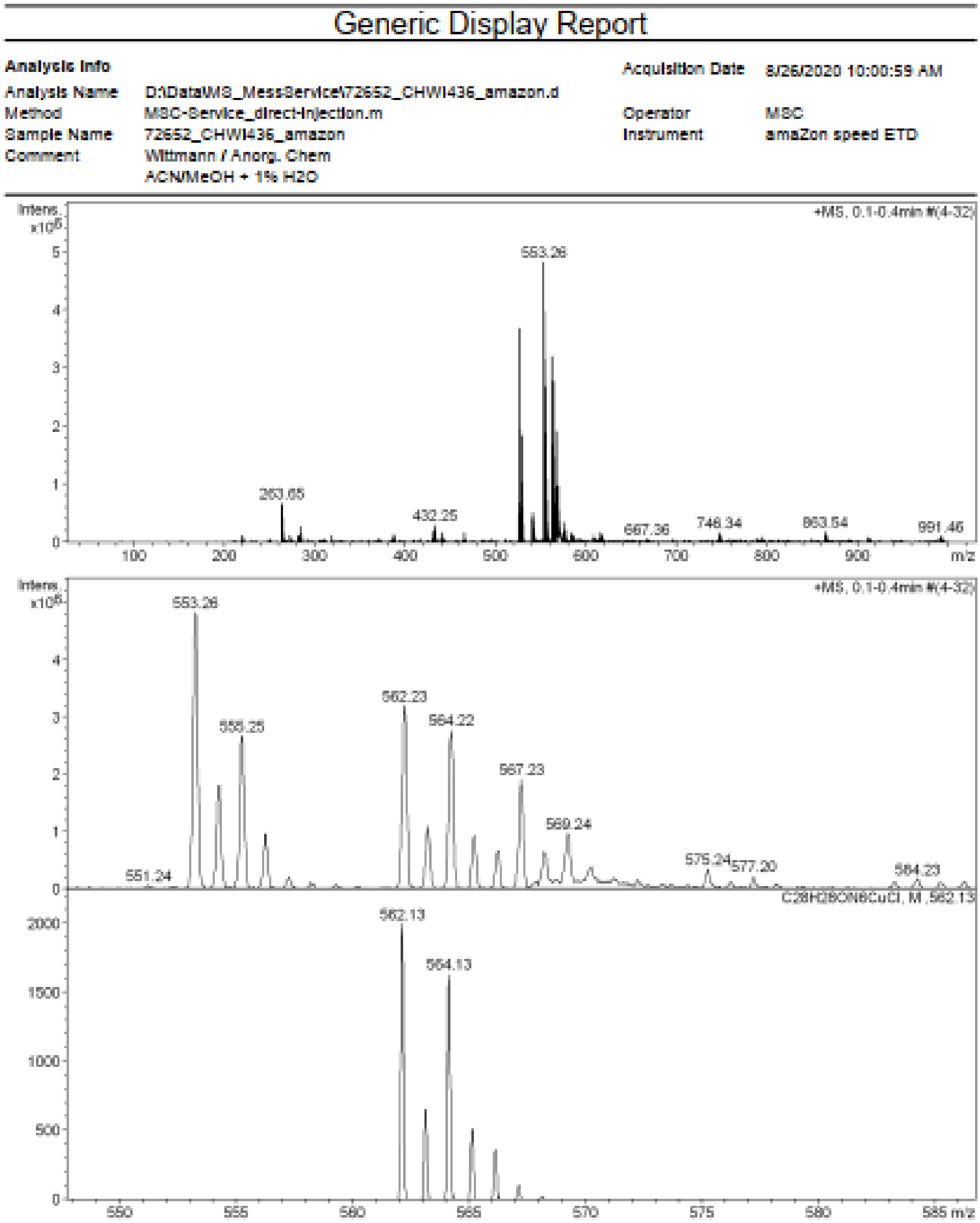

Figure S67. ESI mass spectrum of **3** in positive ion mode.

Generic Display Report

**Analysis Info**		Acquisition Date	8/26/2020 7:16:21 AM
Analysis Name	D:\Data\MS_Service_MSC_Archiv_2020_683_761\726\72645_CHWI409_amazon.d		
Method	MSC-Service_direct-injection.m	Operator	MSC
Sample Name	72645_CHWI409_amazon	Instrument	amaZon speed ETD
Comment	Wittmann / Anorg. Chem<br>ACN/MeOH + 1% H2O		

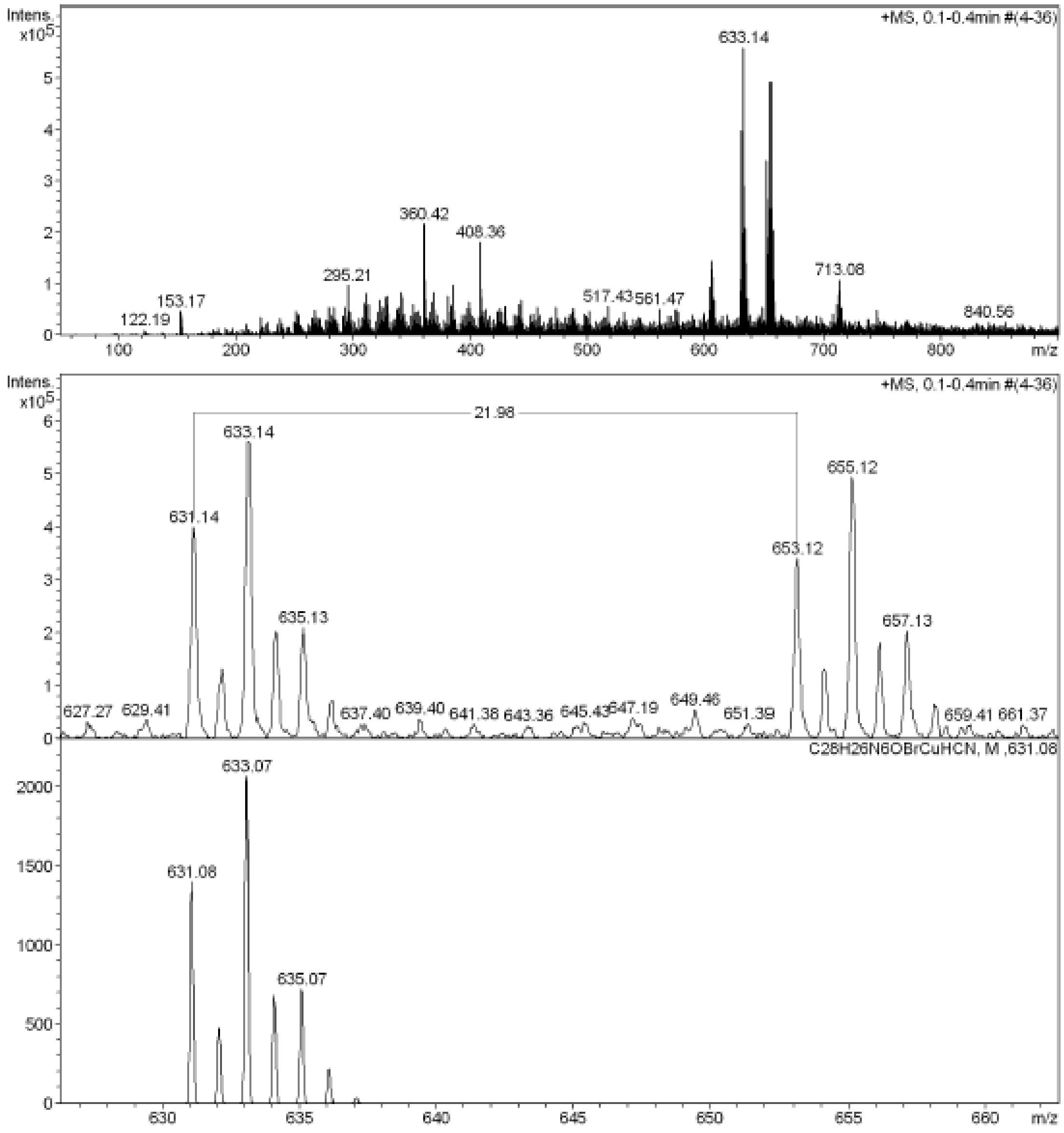

Bruker Compass DataAnalysis 4.0 printed: 8/30/2021 9:55:35 AM Page 1 of 1

Figure S68. ESI mass spectrum of **4** in positive ion mode.

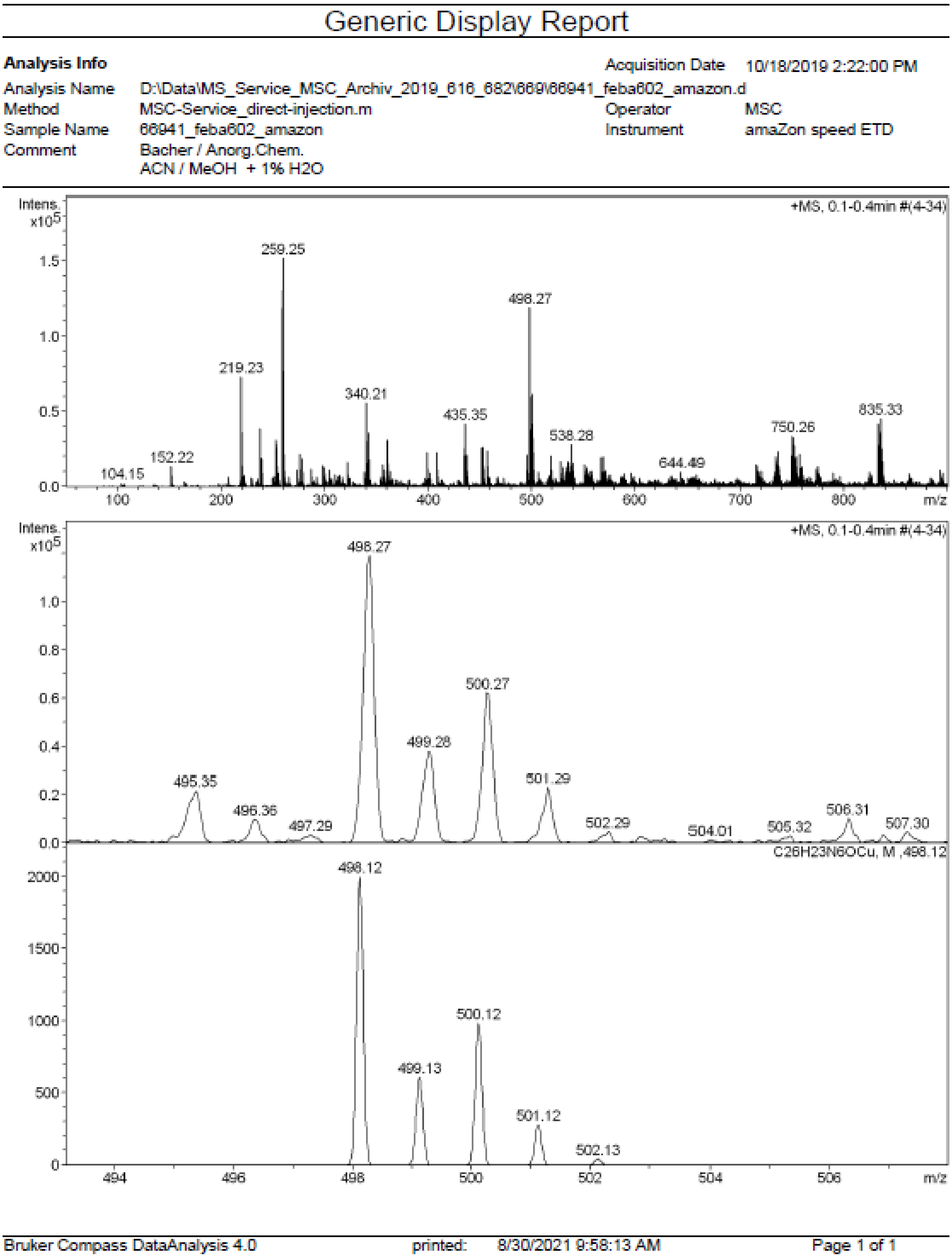

Figure S69. ESI mass spectrum of **5** in positive ion mode.

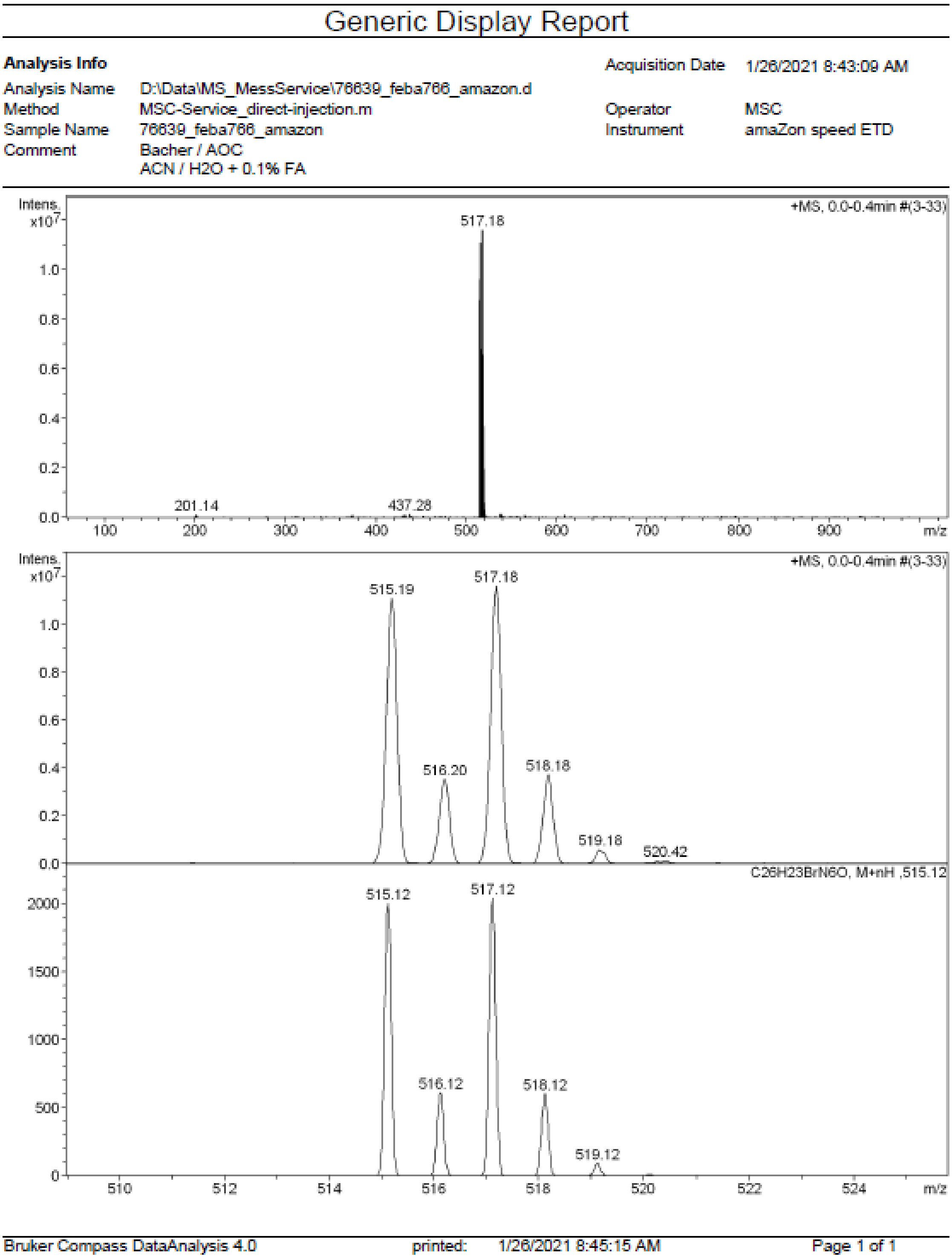

Figure S70. ESI mass spectrum of **6** in positive ion mode.

Generic Display Report

Analysis Info			
Analysis Name	D:\Data\MS_MessService\76365_feba765_amazon.d	Acquisition Date	1/14/2021 9:06:47 AM
Method	MSC-Service_direct-injection.m	Operator	MSC
Sample Name	76365_feba765_amazon	Instrument	amaZon speed ETD
Comment	Bacher / AOC<br>ACN / MeOH + 1%H2O		

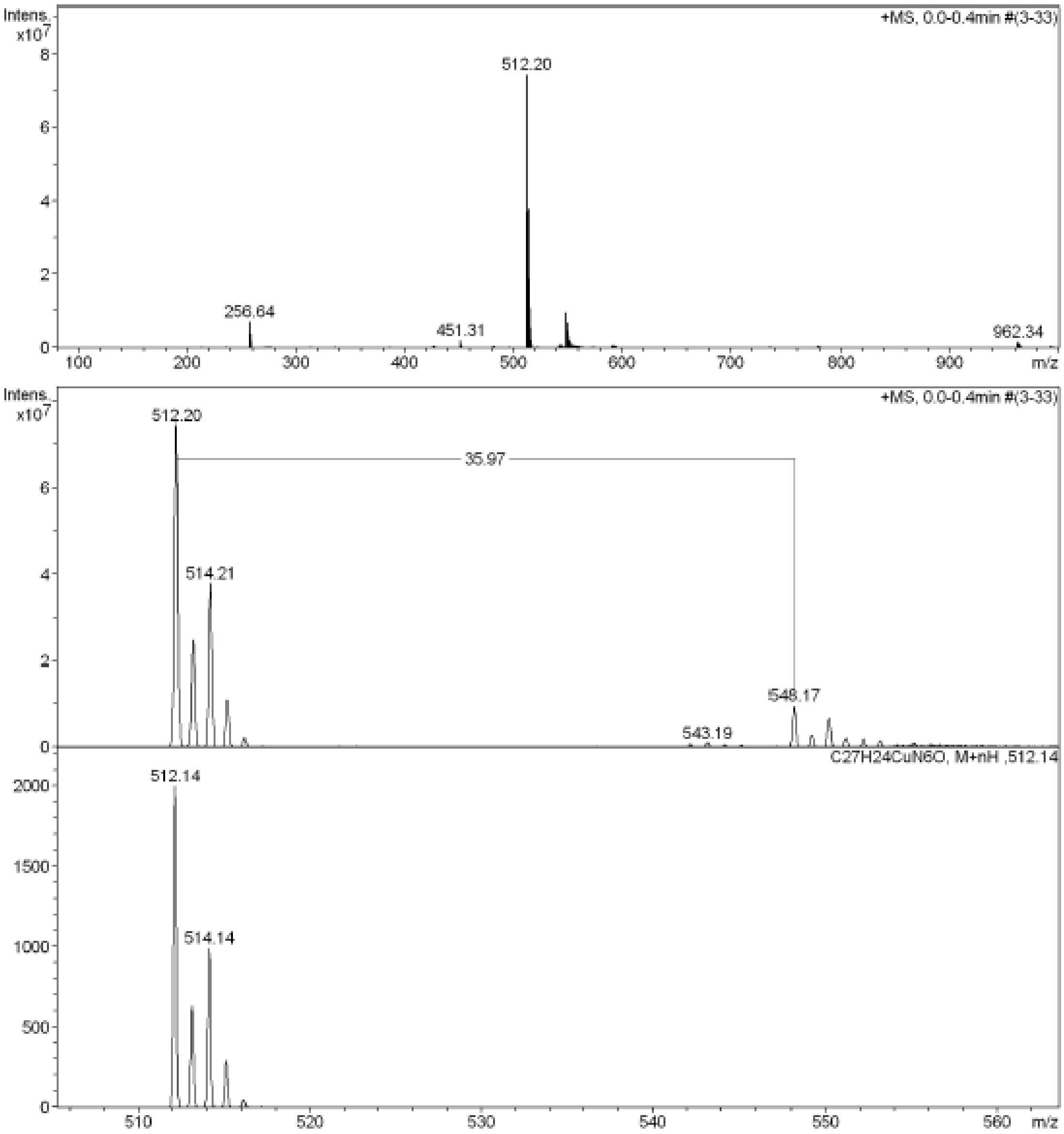

Bruker Compass DataAnalysis 4.0 printed: 1/14/2021 9:09:01 AM Page 1 of 1

Figure S71. ESI mass spectrum of **7** in positive ion mode.

Generic Display Report

Analysis Info

		Acquisition Date	1/26/2021 10:47:27 AM
Analysis Name	D:\Data\MS_Service_MSC_Archiv_2021_762_xxx\766\76642_feba769_amazon.d		
Method	MSC-Service_direct-injection.m	Operator	MSC
Sample Name	76642_feba769_amazon	Instrument	amaZon speed ETD
Comment	Bacher / AOC<br>ACN / H2O + 0.1% FA		

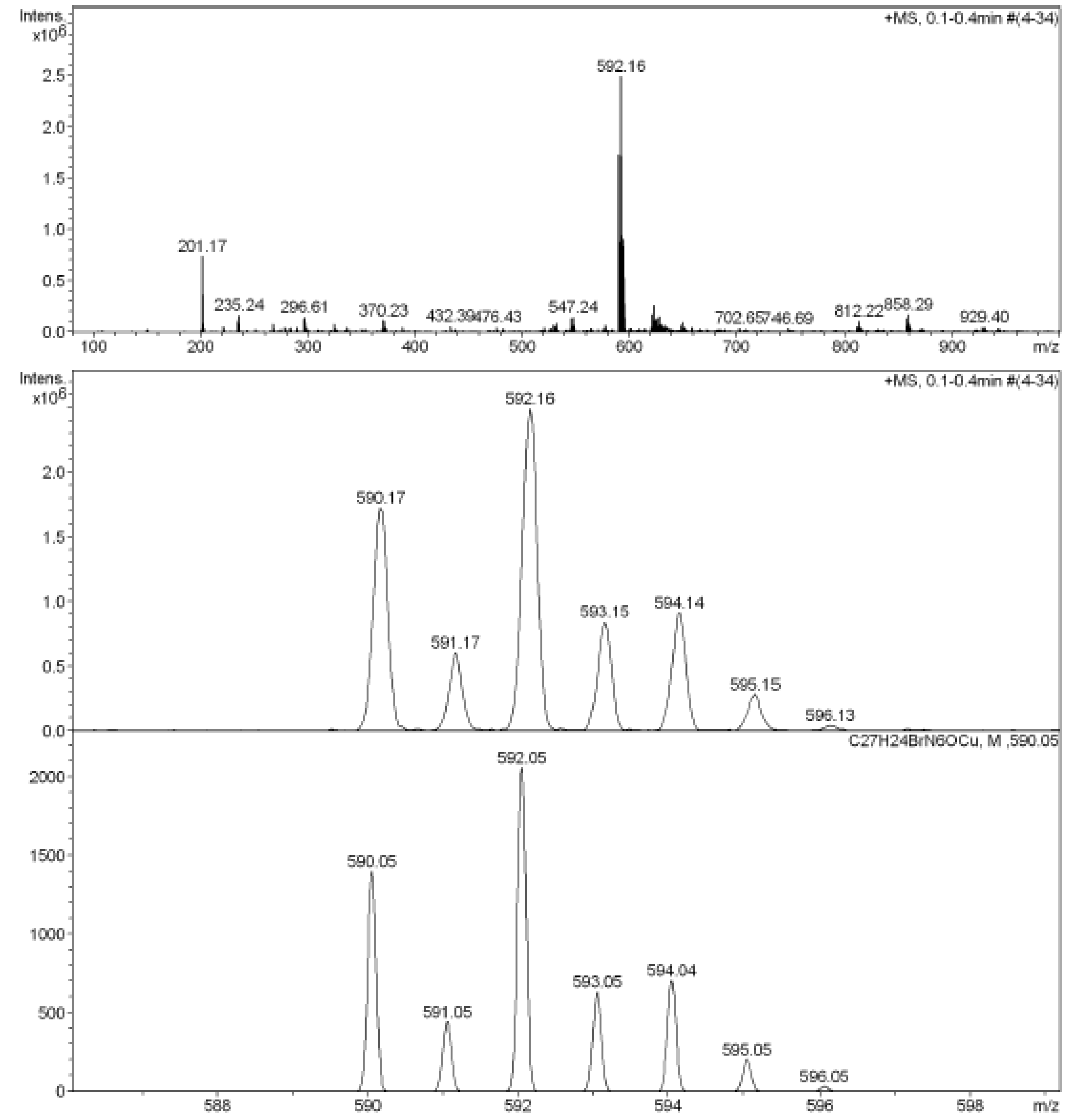

Bruker Compass DataAnalysis 4.0 printed: 8/30/2021 10:00:17 AM Page 1 of 1

Figure S72. ESI mass spectrum of **8** in positive ion mode.

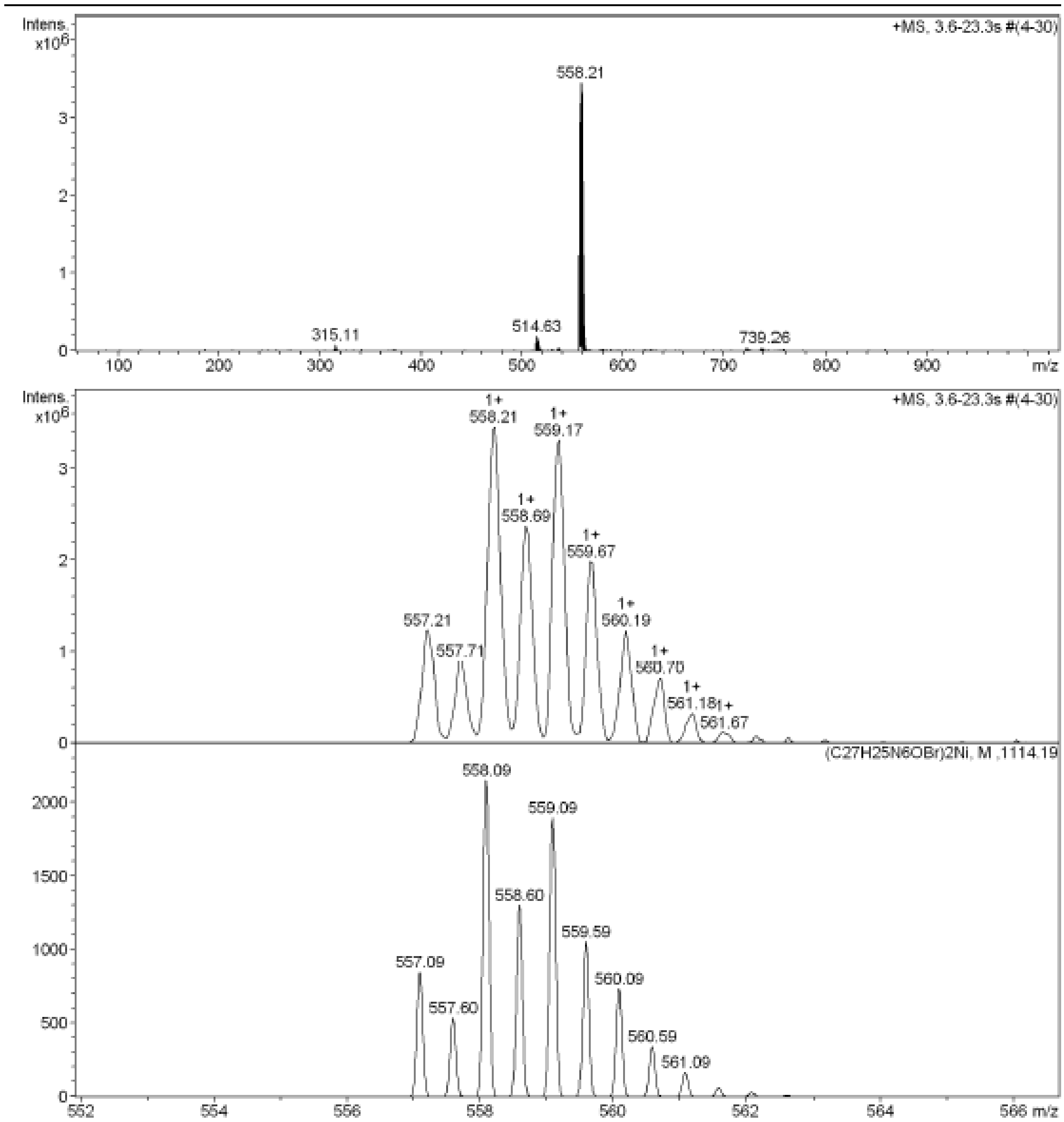

Figure S73. ESI mass spectrum of **[Ni(HL[8])$_2$]Cl$_2$** in positive ion mode.

- **HPLC-HR-mass spectra**

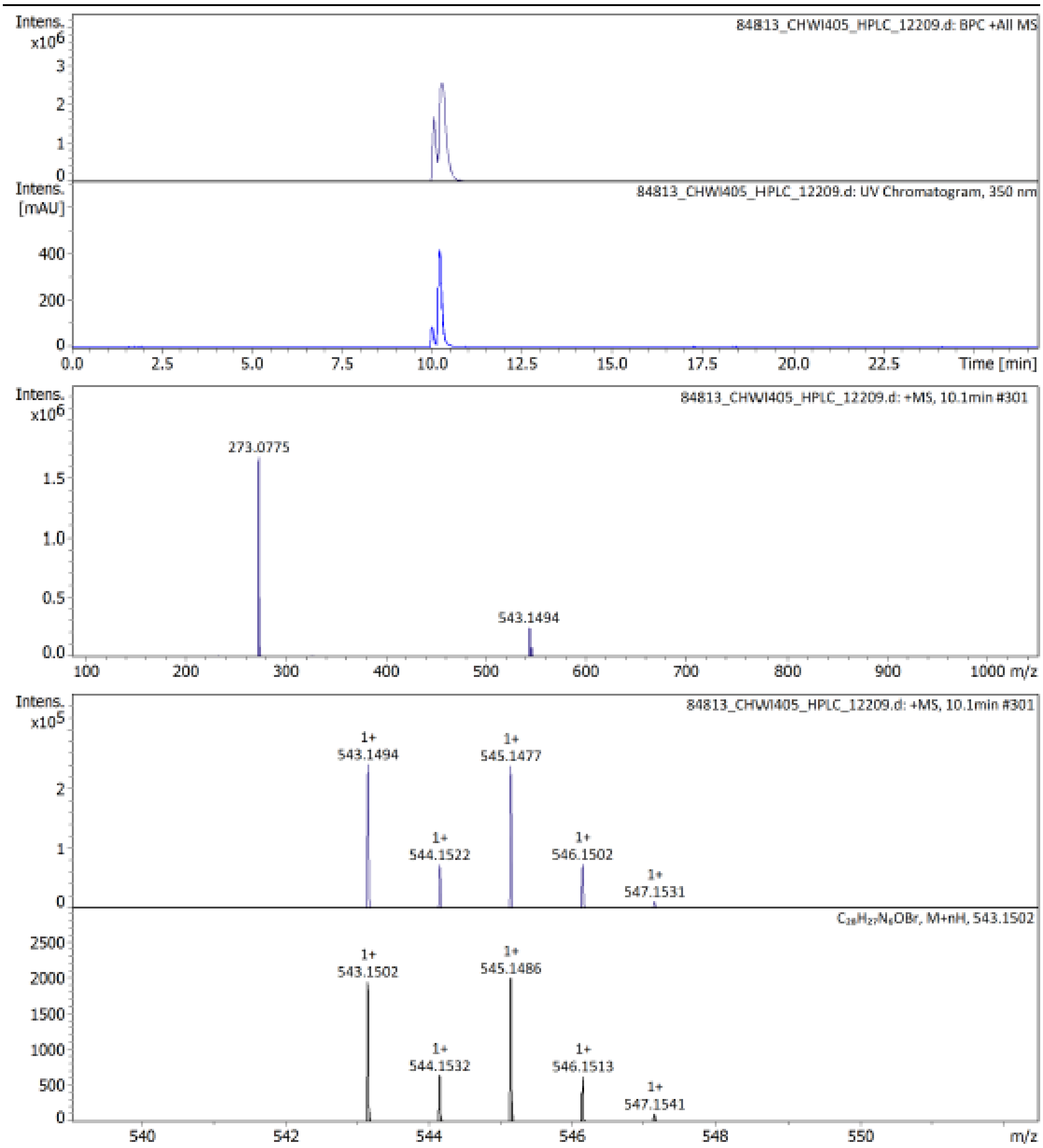

Figure S74. HPLC-HR-mass spectra of **HL⁴** at retention time 10.1 min.

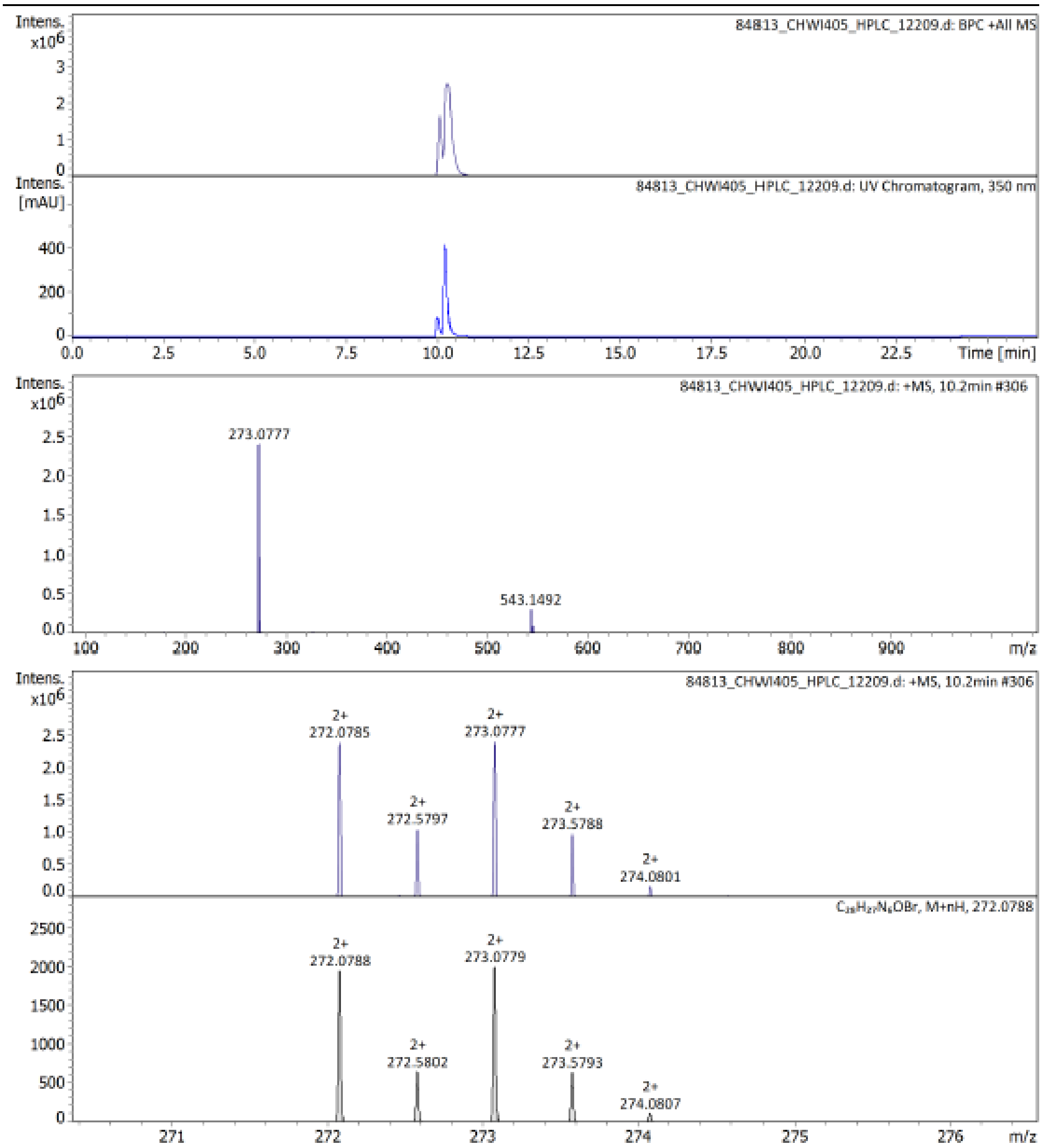

Figure S75. HPLC-HR-mass spectra of **HL⁴** at retention time 10.2 min.

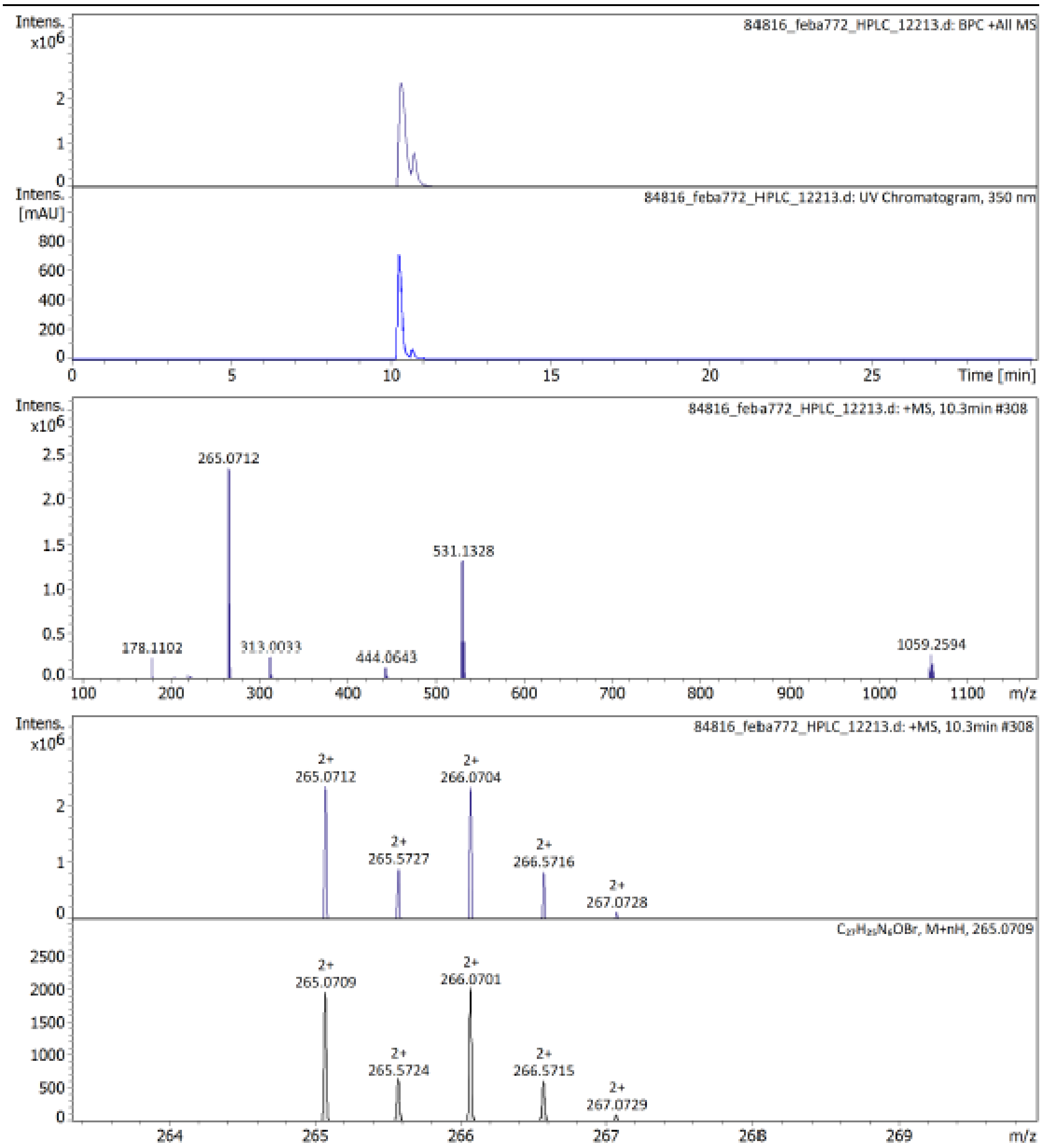

Figure S76. HPLC-HR-mass spectra of **HL⁸** at retention time 10.3 min.

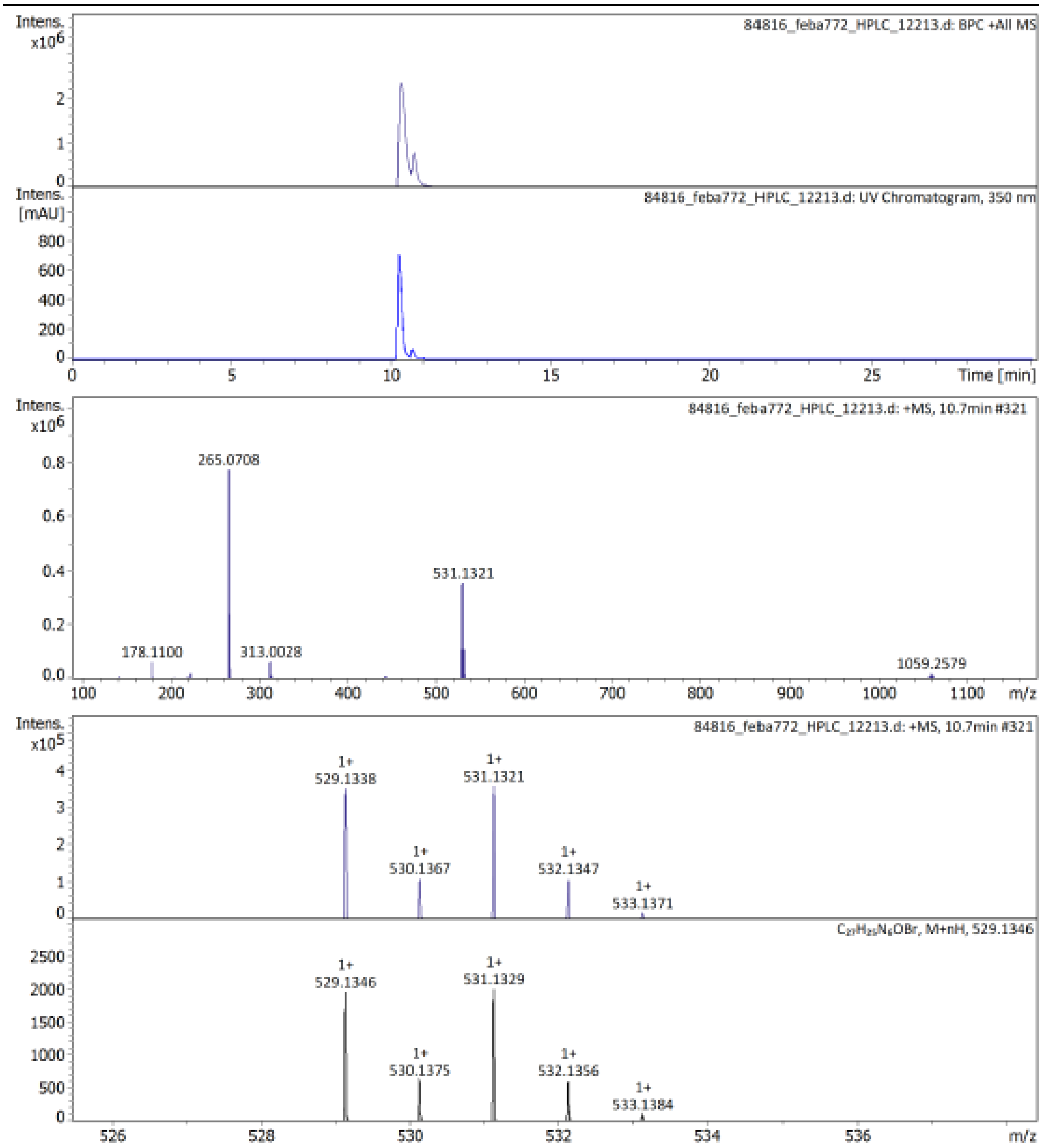

Figure S77. HPLC-HR-mass spectra of **HL⁸** at retention time 10.7 min.

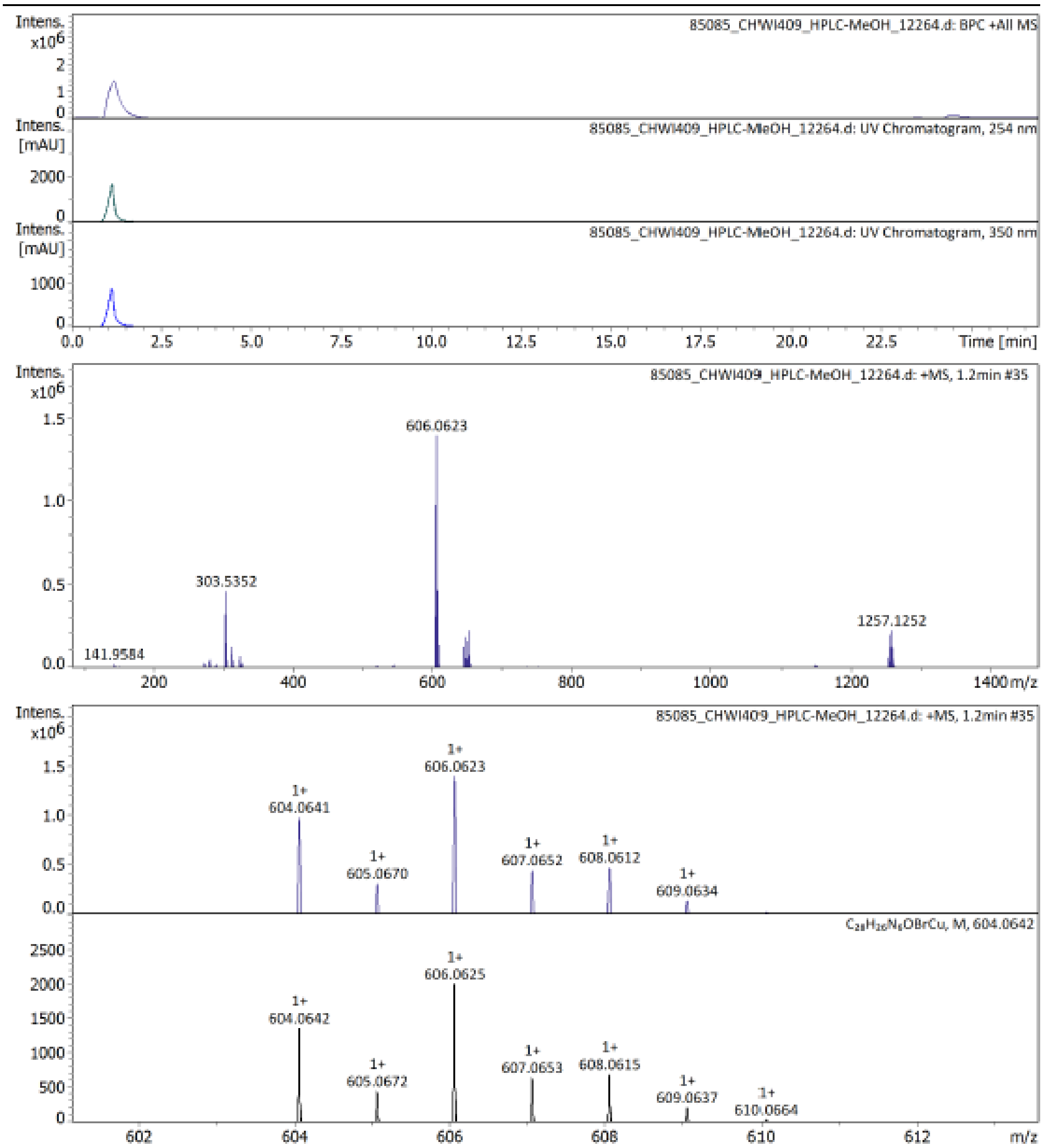

Figure S78. HPLC-HR-mass spectra of **4** at retention time 1.2 min.

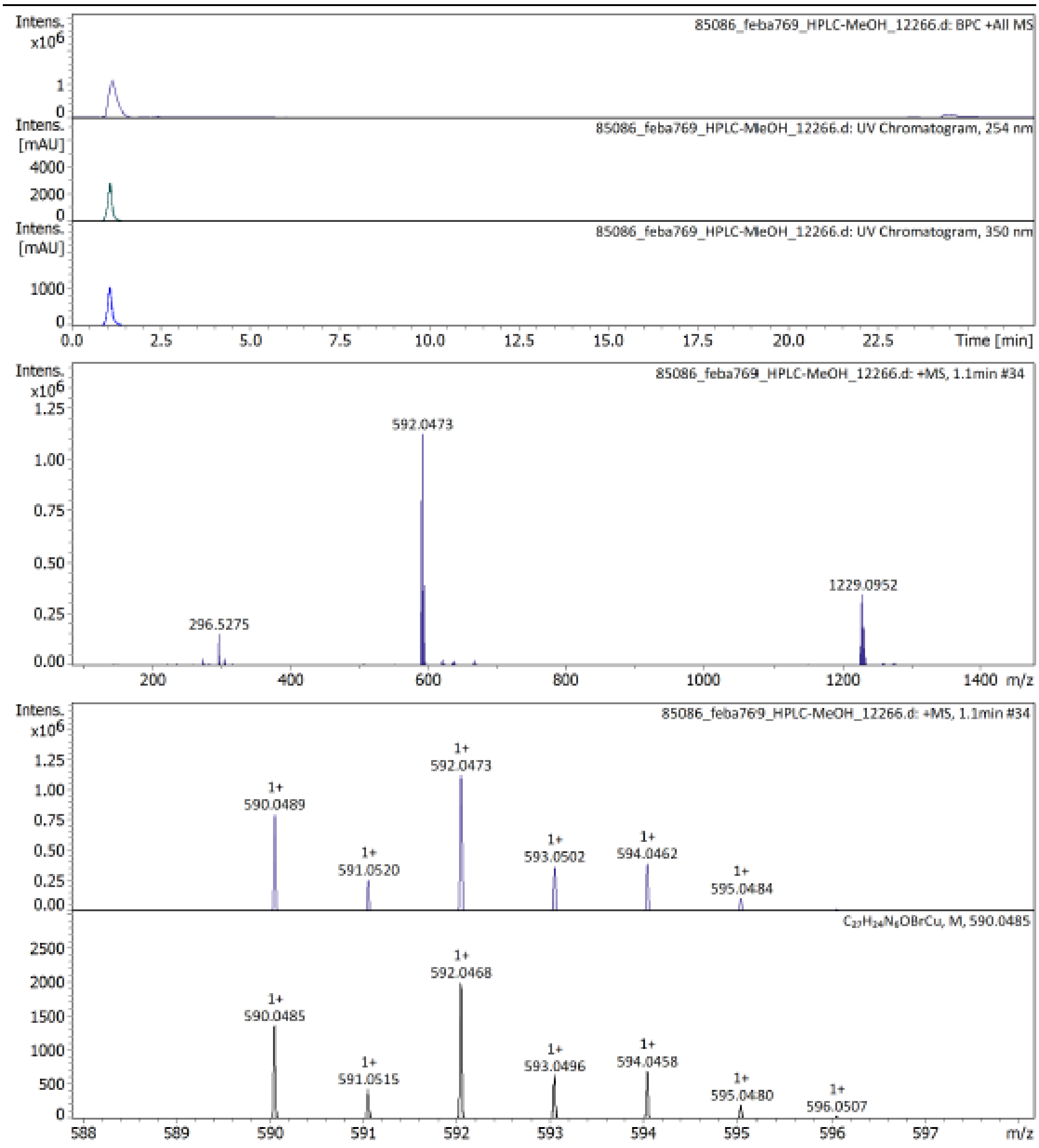

Figure S79. HPLC-HR-mass spectra of **8** at retention time 1.1 min.

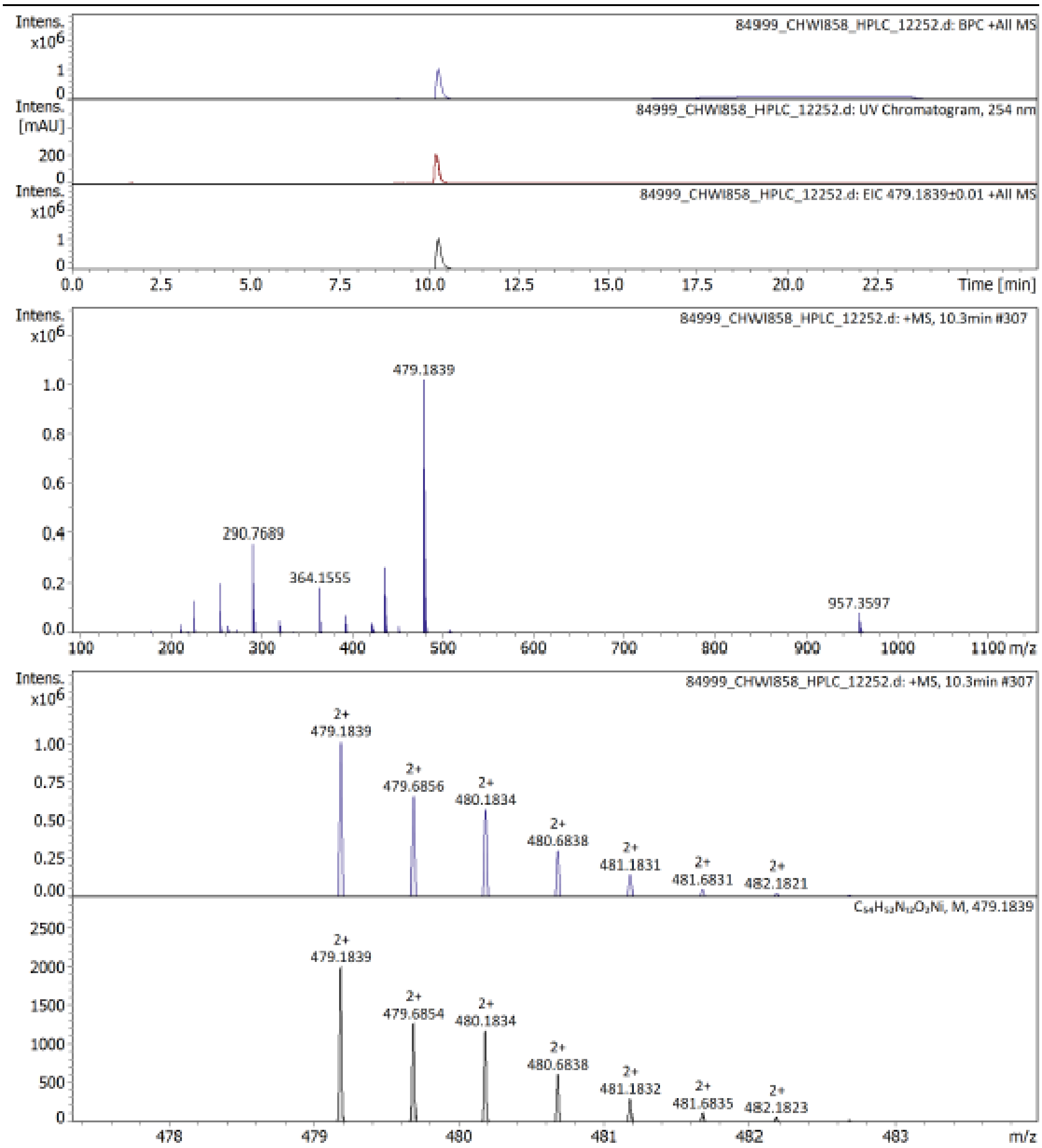

Figure S80. HPLC-HR-mass spectra of **[Ni(HL[7])$_2$]Cl$_2$** at retention time 10.3 min.

- **References**

(1) *QikProp*; 2009.

(2) Ioakimidis, L.; Thoukydidis, L.; Mirza, A.; Naeem, S.; Reynisson, J. Benchmarking the Reliability of QikProp. Correlation between Experimental and Predicted Values. *QSAR Comb. Sci.* **2008**, *27*, 445–456.

(3) *Scigress Ultra V F.J 2.6*.

(4) Eurtivong, C.; Reynisson, J. The Development of a Weighted Index to Optimise Compound Libraries for High Throughput Screening. *Mol. Inf.* **2019**, *38*, 1800068.

(5) Dennington, R.; Keith, T.; Millam, J. *GaussView*; Semichem Inc.: Shawnee Mission, KS, 2016.

(6) Becke, A. D. Density-Functional Exchange-Energy Approximation with Correct Asymptotic Behavior. *Phys. Rev. A* **1988**, *38*, 3098–3100.

(7) Becke, A. D. Density-functional Thermochemistry. III. The Role of Exact Exchange. *J. Chem. Phys.* **1993**, *98*, 5648–5652.

(8) Lee, C.; Yang, W.; Parr, R. G. Development of the Colle-Salvetti Correlation-Energy Formula into a Functional of the Electron Density. *Phys. Rev. B* **1988**, *37*, 785–789.

(9) Frisch, M. J.; Pople, J. A.; Binkley, J. S. Self-consistent Molecular Orbital Methods 25. Supplementary Functions for Gaussian Basis Sets. *J. Chem. Phys.* **1984**, *80*, 3265–3269.

(10) Hariharan, P. C.; Pople, J. A. The Influence of Polarization Functions on Molecular Orbital Hydrogenation Energies. *Theoret. Chim. Acta* **1973**, *28*, 213–222.

(11) Wong, M. W. Vibrational Frequency Prediction Using Density Functional Theory. *Chem. Phys. Lett.* **1996**, *256*, 391–399.

(12) Frisch, A.; Foresman, J. *Exploring Chemistry with Electronic Structure Methods.*, 3rd ed.; Gaussian Inc., 1996.

(13) Yu, B.; Reynisson, J. Bond Stability of the “Undesirable” Heteroatom–Heteroatom Molecular Moieties for High-Throughput Screening Libraries. *Eur. J. Med. Chem.* **2011**, *46*, 5833–5837.

(14) Tong, H.; Bell, D.; Tabei, K.; Siegel, M. M. Automated Data Massaging, Interpretation, and e-Mailing Modules for High Throughput Open Access Mass Spectrometry. *J. Am. Soc. Mass Spectrom.* **1999**, *10*, 1174–1187.
